# Red light-induced conjugation of amines through amide bond formation triggered via photooxidation of 3-acylindolizines

**DOI:** 10.1038/s42004-022-00712-5

**Published:** 2022-08-05

**Authors:** Kenji Watanabe, Asuka Kuratsu, Daisuke Hashizume, Takashi Niwa, Takamitsu Hosoya

**Affiliations:** 1 Laboratory for Chemical Biology, RIKEN Center for Biosystems Dynamics Research (BDR), Kobe, Japan; 2 RIKEN Center for Emergent Matter Science (CEMS), Wako, Saitama Japan; 3 Laboratory of Chemical Bioscience, Institute of Biomaterials and Bioengineering, Tokyo Medical and Dental University (TMDU), Tokyo, Japan

**Keywords:** Synthetic chemistry methodology, Chemical synthesis, Photocatalysis

## Abstract

The development of a conjugation method initiated by irradiation of long-wavelength light (>500 nm) to prepare densely functionalized molecules while avoiding undesired photodegradation has attracted considerable attention. Here we show an amide bond formation method based on the photoreaction of 3-acylindolizines in the presence of amines triggered via red-light irradiation. Photooxidation of 3-acylindolizines using a catalytic amount of a photosensitizer and red light-emitting diodes (660 nm) affords the corresponding conjugated amides in nearly quantitative yields within <5 min. This transformation can be performed in aqueous organic solvents and is applicable to diverse aliphatic amines with various functional groups, including the moieties responsive to short-wavelength light.

## Abstract (web summary)

Photoinduced conjugation has been broadly used for functionalization of molecules but is limited by possible photodegradation. Here, the authors report a robust amide bond formation method based on the photoreaction of 3-acylindolizines with amines triggered via red-light irradiation in nearly quantitative yields within less than 5 min.

## Introduction

Photoinduced conjugation has been used for synthesizing functional materials in a broad range of fields such as bioconjugation chemistry^1,2^ and polymer science^3^ because properties of light enable the spatiotemporal control of the chemical reactions. In fact, various reactions involving a photoinitiation process have been developed^4–10^. However, these methods require irradiation with short-wavelength light (<500 nm), which has high energy and can induce undesired photodegradation of the target molecules having absorption at these wavelengths. Therefore, to apply this technology for the modification of densely functionalized molecules, development of photoreactions triggered via long-wavelength light irradiation is urgently required.

**Fig. 1:**
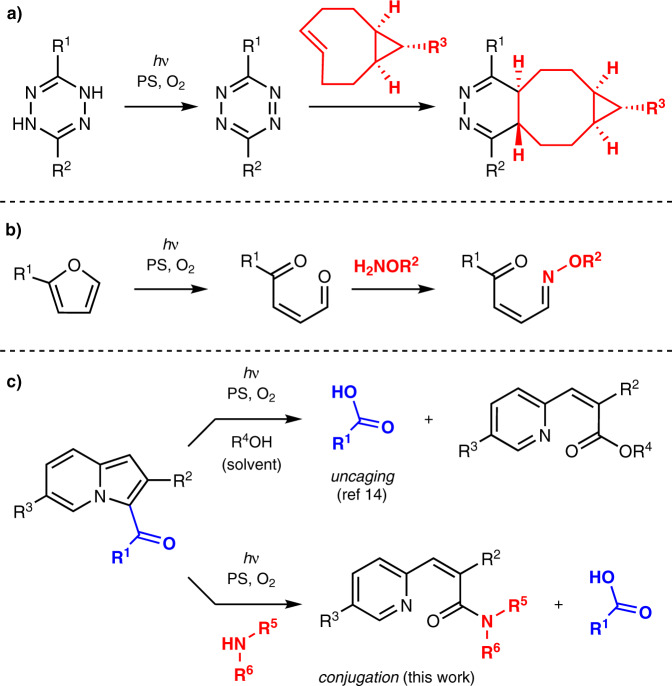
Photooxidation-initiated conjugation. **a** Inverse-electron-demand Diels–Alder reaction initiated via tetrazine formation. **b** Oxime ligation reaction via aldehyde formation. **c** This work: amide formation via photooxidative ring-opening of indolizines. PS photosensitizer.

Triggering chemical reactions via irradiation with long-wavelength light is a challenging task because the energy associated with long-wavelength light is generally insufficient to directly activate covalent bonds. In this context, using a photosensitizer, which can employ long-wavelength light for generating highly reactive singlet oxygen via energy transfer to triplet oxygen, has emerged as an attractive strategy. For instance, Fox et al. developed a red light-induced inverse-electron-demand Diels–Alder reaction involving photooxidation to provide tetrazines that smoothly react with trans-cyclooctenes (Fig. 1a)^11^. Truong and Forsythe employed red light-induced photooxidation of dihydrogen tetrazines for activation of inverse-electron-demand Diels–Alder conjugation of tetrazines and norbornenes^12^. Truong and Barner-Kowollik reported a photoinitiated oxime ligation reaction involving the generation of aldehydes by the photooxidation of furans (Fig. 1b)^13^. These works successfully applied long-wavelength light for the in situ preparation of agents used in well-established bioorthogonal reactions.

We recently reported a 3-acylindolizine-based photouncaging system that liberates carboxylic acids from indolizines upon red-light irradiation in the presence of a sensitizer such as methylene blue (Fig. 1c)^14,15^. Although the indolizine core is chemically stable, it is responsive to singlet oxygen to produce β-pyridylacrylic acids or the corresponding esters by capturing a solvent molecule such as water or alcohols, respectively, with the release of carboxylic acids^16^. The formation of these byproducts indicates that a nucleophilic agent such as an amine could bond with the pyridine moiety upon red-light irradiation (Fig. 1c). We anticipated that this approach would provide a practical conjugation method because the uncaging occurred within a few minutes. Herein, we report a red light-induced amidation reaction for the chemical conjugation of 3-acylindolizines with amines. Owing to the use of ubiquitous amino groups, this method is advantageous in terms of practical application for modifying bio/functional molecules.

## Results and discussion

To verify our hypothesis, we commenced our investigation with the photoreaction of various 3-acylindolizines **2** with benzylamine (**1a**) as the model substrate under red-light (660 nm) irradiation in the presence of 1 mol% methylene blue (**PS1**, Table 1). As expected, the reaction using 3-acetylindolizine **2a** afforded amide **3a** with an excellent yield within only 3 min of irradiation. The screening of diverse 3-acyl groups such as benzoyl (**2b**), methoxycarbonyl (**2c**), and phenoxycarbonyl (**2d**) revealed that the use of **2a** afforded amide **3a** in the highest yield. The installation of a substituent at the 1- or 5-position of indolizine was tolerated, affording the products **2e** and **2f**, respectively, whereas a 4-methyl derivative resulted in a poor yield presumably due to steric hindrance (Supplementary Table S1). The reaction with the substrates having methyl (**2g**) or phenyl (**2h**) groups at the 2-position instead of the methoxy group also occurred, although prolonged photoirradiation time was required. In the course of our investigation, we did not observe the formation of any byproduct derived from benzylamine (**1a**), such as oxygenized^17^ or oxidatively dimerized compounds^18^, probably because of the short irradiation time. The photoreaction of **2a** in the presence of water-*d*_2_ (17 v/v%) also afforded **3a** in a comparable yield, suggesting that the amidation preferentially proceeded than the carboxylic acid formation via the reaction with water due to the high nucleophilicity of the amine.

**Table 1:**
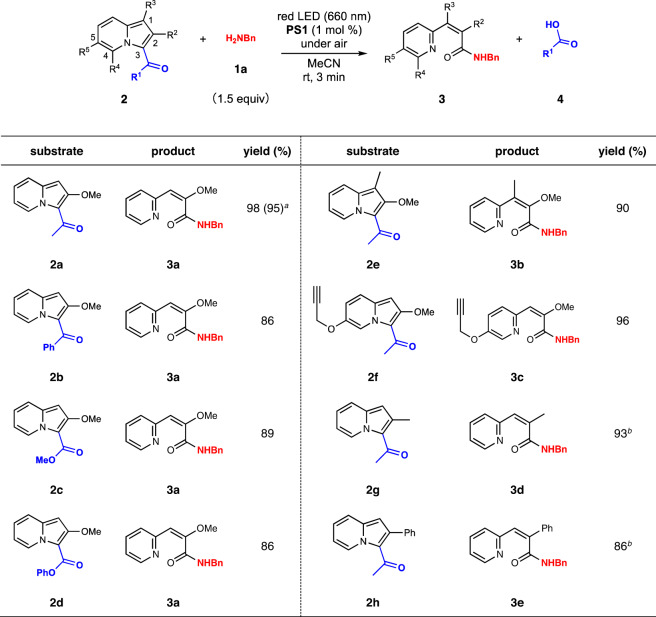
Photoreaction of 3-acylindolizines with benzylamine.

Isolated yields are shown.

^*a*1^H NMR yield of the product when the reaction was conducted in acetonitrie-*d*_3_/water-*d*_2_ (17 v/v%) is shown in the parentheses. ^*b*^Irradiated for 4 min.

**Fig. 2:**
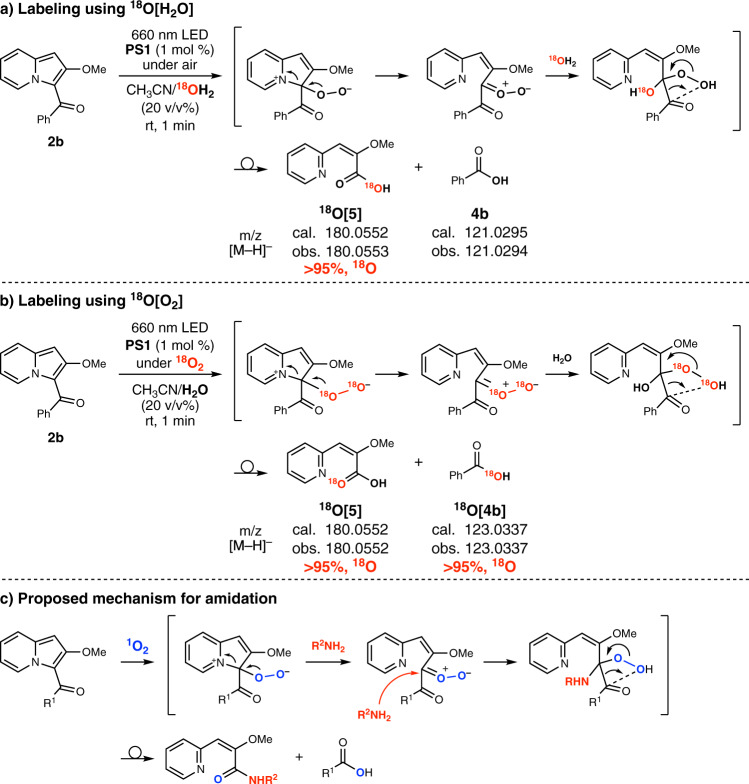
Mechanistic studies. ^18^O-Labeling using (**a**) ^18^O[H_2_O] or (**b**) ^18^O[O_2_]. **c** Proposed mechanism for amidation.

We conducted several experiments to gain insight into the mechanism of the photooxidative amide bond formation. The absorption spectra of **2a** or its mixture with **1a** showed no absorbance in the red-light region, which suggests that the red-light irradiation does not excite the substrates but **PS1** (Supplementary Fig. S1). Control experiments under dark (Table 2, entry 2), under argon (entry 3), or in the absence of **PS1** (entry 4) resulted in no reaction, indicating that the process involves photooxidation mediated by **PS1**. Singlet oxygen, generated by photoirradiation to **PS1** under air, is likely to be the reactive species as the addition of sodium azide (NaN_3_, 10 equiv), a known scavenger for singlet oxygen, shut down the process (entry 5). A similar trend was observed when a chlorin e6 derivative (**PS2**) was used as the photosensitizer instead of **PS1** (entries 6 and 7). We also conducted the reaction using ^18^O-labeled compounds in the absence of an amine nucleophile to validate the reaction with a nucleophile with 3-acylindolizines (Fig. 2a, b, and Supplementary Fig. S3). The reaction of **2b** in a mixture of acetonitrile and ^18^O-labeled water (^18^O[H_2_O]) afforded ^18^O-labeled β-pyridylacrylic acid **5** (Fig. 2a), whereas the presence of ^18^O-labeled oxygen (^18^O[O_2_]) provided a mixture of **5** and ^18^O-labeled benzoic acid (Fig. 2b). These results are consistent with the mechanism involving nucleophilic incorporation of water to provide a carboxylic acid. Overall, we consider that the amidation occurs via singlet oxygen-mediated oxidative ring-opening of an indolizine ring followed by nucleophilic addition of an amine (Fig. 2c). Interestingly, when **2a** was irradiated with 370 nm LED in the absence of **PS1**, product **3a** was obtained in 63% yield (Table 2, entry 8). This result indicates that direct activation of **2a** by 370 nm light can produce singlet oxygen.

**Table 2:** Control experiments.


Entry	Variation from standard conditions	Yield (%)^a^
1	None	>95
2	Under dark	<5
3	Under argon	6
4	Absence of **PS1**	<5
5	Addition of NaN_3_ (10 equiv) in water-*d*_2_	<5
6	Addition of water-*d*_2_, **PS2** instead of **PS1**	>95
7	Addition of NaN_3_ (10 equiv) in water-*d*_2_, **PS2** instead of **PS1**	<5
8	Absence of **PS1**, 370 nm LED, 60 min	63


^a^Yields determined by ^1^H NMR measurements.

**Fig. 3:**
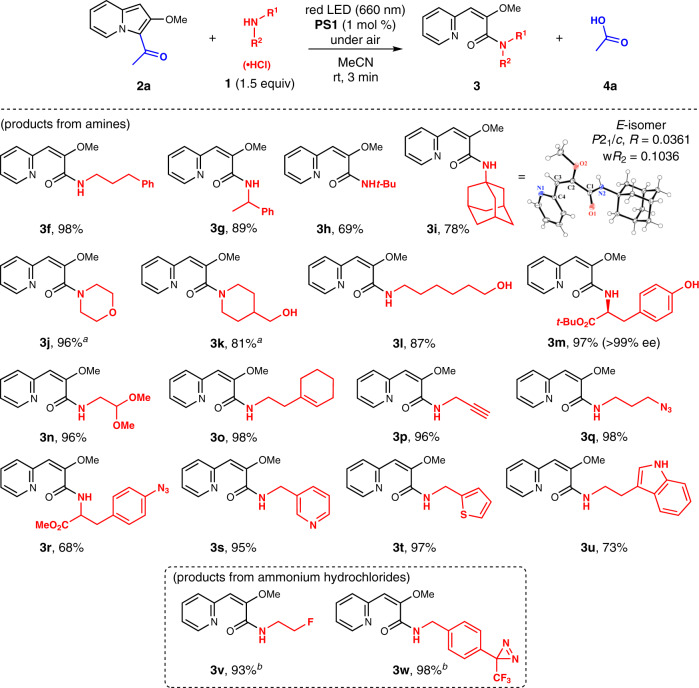
Substrate scope of amines. Isolated yields are shown. ^a^Irradiated for 4 min. ^b^Na_2_CO_3_ (1.5 equiv), MeCN/H_2_O (40/1).

We then investigated the substrate scope of the photoreaction (Fig. 3). The reactions proceeded smoothly with primary amines having linear or branched chains to afford the corresponding amides (**3f** and **3g**). *tert*-Butylamine and 1-aminoadamantane afforded the products **3h** and **3i**, respectively. We established the structure of the product **3i** as an *E*-isomer by single-crystal X-ray diffractometry. We did not observe the formation of the *Z*-isomer using ^1^H NMR spectroscopy measurements of the reaction mixtures. The reaction was applicable to cyclic secondary amines to provide morpholine and piperidine derivatives **3j** and **3k**, respectively, whereas acyclic secondary amines such as diethylamine, dibenzylamine, and *N*-methylbenzylamine did not furnish the expected products (Supplementary Table S1). The reaction with the less nucleophilic aniline was also unsuccessful (Supplementary Table S1). When using amino alcohols, amide bond formation occurred preferentially over ester formation to afford the products **3k–3m**. Tyrosine was conjugated with the pyridine moiety without the loss of enantiopurity. Further, amines having various functional groups, such as the acetal, allyl, propargyl, and aromatic azido groups, participated in the reaction to furnish the corresponding amides **3n**–**3r**. Moreover, the reaction proceeded with amines containing heteroaryl rings such as the pyridinyl, thienyl, and indolyl rings (**3s**–**3u**). Amine hydrochlorides could also be used in the reaction by adding a stoichiometric amount of sodium carbonate in aqueous acetonitrile solutions, affording the products **3v** and **3w**. Overall, these results demonstrate the excellent functional group tolerance of this conjugation method involving the photooxidation of indolizine rings.

**Fig. 4:**
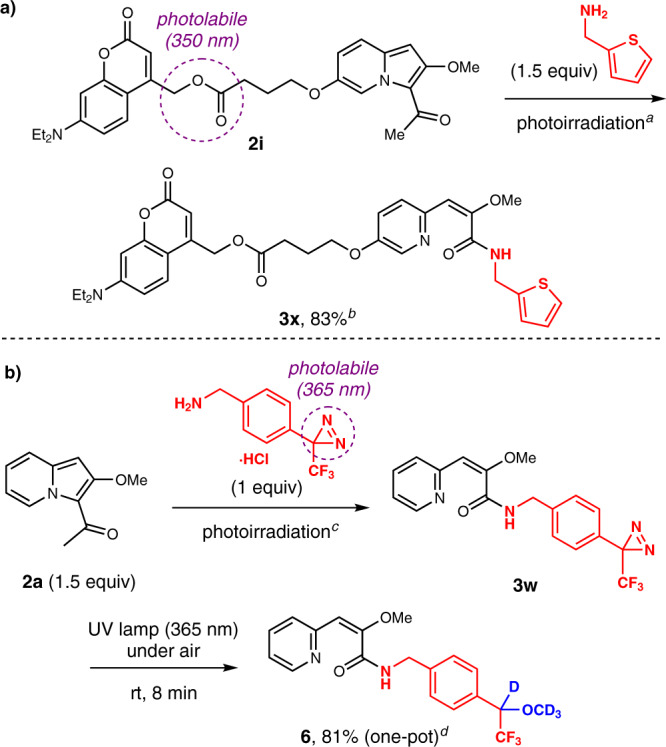
Wavelength-selective photoreactions. Photoconjugation with substrates bearing photoreactive (coumarin-4-yl)methyl ester (**a**) and diazirinyl (**b**) moieties. ^a^Red light-emitting diode (LED) (660 nm), methylene blue (1 mol %), MeCN, under air, room temperature, 3 min. ^b^Isolated yield. ^c^Red LED (660 nm), methylene blue (1 mol %), Na_2_CO_3_ (1 equiv), PhCF_3_ (internal standard, 1 equiv), methanol-*d*_4_/water-*d*_2_ (13/1), under air, room temperature, 3 min. ^d^Yield determined by ^19^F NMR measurement.

Notably, this method tolerates the substrates that are sensitive to short-wavelength light because the reaction occurs under irradiation with red light, which has long wavelength. Thus, we achieved the conjugation of the coumarin-linked indolizine **2i**, whose (coumarin-4-yl)methyl ester is cleavable upon 350 nm-light^19^. 2-Thiophenemethylamine smoothly underwent the red light-induced amide formation to furnish the functionalized amide **3x** in high yield (Fig. 4a). Moreover, an amine with a diazirinyl group, which generates carbene species under 365-nm ultraviolet-light irradiation^20,21^, was successfully conjugated to provide **3w** (Fig. 3). Emission spectrum of the red LED light source and the absorption spectrum of the substrates showed no overlap, exemplifying the wavelength selectivity (Supplementary Fig. S2). Motivated by this result, we performed a continuous photoreaction using diazirinyl amine hydrochloride. As shown in Fig. 4b, red light-induced amide formation in methanol-*d*_4_/water-*d*_2_ (13/1) followed by irradiation with 365-nm ultraviolet light for 8 min afforded the methanol adduct **6** in high yield without purification.

**Fig. 5:**
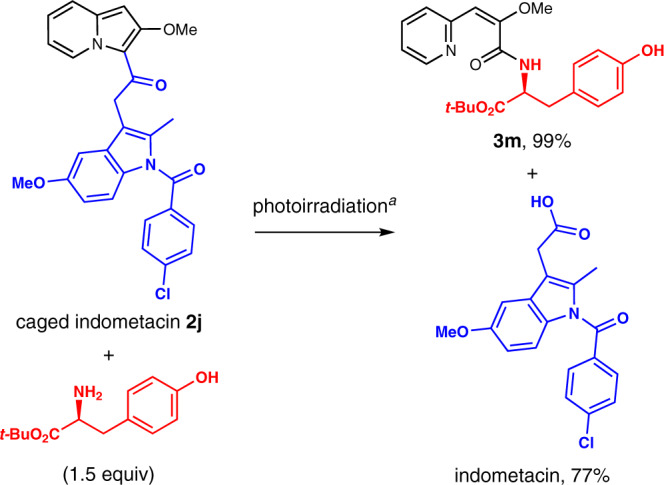
Simultaneous conjugation and release of indometacin. Yields determined by high performance liquid chromatography (HPLC) measurements. ^a^Red LED (660 nm), methylene blue (1 mol%), MeCN, under air, room temperature, 3 min.

Red-light irradiation of a mixture of indometacin–indolizine conjugate **2j** and *C*-protected tyrosine afforded free indometacin and tyrosine-derived amide **3m** in high yield (Fig. 5), indicating that the photooxidation of 3-acylindolizines enables the simultaneous amide conjugation and release of functional carboxylic acids.

The compatibility of the method in an aqueous solution (Table 1) prompted us to perform the amidation in a water medium without organic co-solvent. The reaction in water using water-soluble 3-acylindolizine **2k** and propargylamine afforded desired amide **3p** quantitatively, while increased amount of the amine (5 equiv) and **PS1** (5 mol%) and extended reaction time (5 min) was required (Table 3, entry 1). This is probably due to the reduced singlet oxygen lifetime in water (~3 μs) compared to that in acetonitrile (~80 μs)^22^. Using a nearly neutral buffered solution (pH 7.4) lowered the yield, which could be attributed to the decrease in nucleophilicity of the amine due to the protonation (p*K*_b_ = 9) (entry 2). Indeed, increasing the pH of the buffered solution to 8.5 provided amide **3p** in high yield (entry 3). These results indicated a potential applicability of this method to biofunctional molecules that are only soluble in water, although careful control of the pH is necessary.

**Table 3:** Photoreaction in water.


Entry	Solvent	Yield (%)^a^
1	Water	>95
2	Sodium phosphate buffer (pH 7.4)	35
3	Sodium phosphate buffer (pH 8.5)	93

^a^Yield determined by ^1^H NMR measurement.

## Conclusion

In summary, we developed a red light-induced conjugation method of amines with chemically stable 3-acylindolizines. The reaction, which involved photooxidation and amide bond formation, proceeded rapidly with a broad substrate scope. This method is expected to be applied to the modification of biological and functional materials, although consideration to the potential damage caused by singlet oxygen should be paid. Further studies including the application of this method for the synthesis of functional molecules are currently ongoing in our laboratory.

## Methods

### General information

See Supplementary Methods, general information (page S6).

### Chemicals

See Supplementary Methods, chemicals (page S7).

### Synthesis of substrates

See  Supplementary Methods, synthesis of substrates (pages S8–S14).

### Procedures for photoreactions

See Supplementary Methods, procedures for photoreactions (pages S15–S26).

### NMR charts

See Supplementary Data 1 for NMR charts of all synthesized compounds.

### Chiral HPLC charts

See Supplementary Methods, HPLC charts (page S27) for **3m** (Fig. 3).

### ORTEP diagram and crystallographic data

See Supplementary Methods, ORTEP diagram and crystallographic data (page S28–S29) for **3i** (Fig. 3).

### Cif file

See Supplementary Data 2 for a cif file of **3i** (Fig. 3).

## Supplementary information

Supplementary Information

Description of Additional Supplementary Files

Supplementary Data 1

Supplementary Data 2

## Data Availability

Supplementary Information includes Supplementary Methods, absorption spectra of **2a** in the absence and presence of benzylamine (Fig. S1), absorption spectra of substrates and emission spectra of red LEDs (Fig. S2), SI mass spectra for ^18^O-labeling experiments (Fig. S3), unapplicable substrates (Table S1). Supplementary Data 1 includes NMR charts of all synthesized compounds. Supplementary Data 2 includes a cif file of **3i** (Fig. 3). The X-ray crystallographic data of **3i** (Fig. 3) has been deposited at the Cambridge Crystallographic Data Center (CCDC), under deposition number CCDC 2149818. The data can be obtained free of charge from The Cambridge Crystallographic Data Center via www.ccdc.cam.ac.uk/data_request/cif. Extra data are available from the corresponding authors upon request.
