# Supplementary material for: Red light-induced conjugation of amines through amide bond formation triggered via photooxidation of 3-acylindolizines
Source: Commun Chem. 2022 Aug 5;5:91. doi: 10.1038/s42004-022-00712-5 (PMC9814406; doi:10.1038/s42004-022-00712-5)
Supplement: Supplementary file 1 — Supplementary Information [file 42004_2022_712_MOESM1_ESM.pdf]

Supplementary Information for

Red light-induced conjugation of amines through amide bond formation triggered via photooxidation of 3-acylindolizines

Kenji Watanabe,[1] Asuka Kuratsu,[1] Daisuke Hashizume,[2] Takashi Niwa,[1,3] Takamitsu Hosoya[1,3]

[1]Laboratory for Chemical Biology, RIKEN Center for Biosystems Dynamics Research (BDR), 6-7-3 Minatojima-minamimachi, Chuo-ku, Kobe 650-0047, Japan

[2]RIKEN Center for Emergent Matter Science (CEMS), 2-1 Hirosawa, Wako, Saitama 351-0198, Japan

[3]Laboratory of Chemical Bioscience, Institute of Biomaterials and Bioengineering, Tokyo Medical and Dental University (TMDU), 2-3-10 Kanda-Surugadai, Chiyoda-ku, Tokyo 101-0062, Japan

Additional Results

Absorption spectra of **2a** in the absence and presence of benzylamine (**Fig. S1**) S2

Absorption spectra of substrates and emission spectra of red LEDs (**Fig. S2**) S3

ESI mass spectra for ^{18}O-labeling experiments (**Fig. S3**) S4

Unapplicable substrates (**Table S1**) S5

Supplementary Methods

General information S6

Chemicals S7

Synthesis of substrates S8–S14

Procedures for photoreactions S15–S26

Chiral HPLC charts S27

ORTEP diagram and crystallographic data S28–S29

References S30

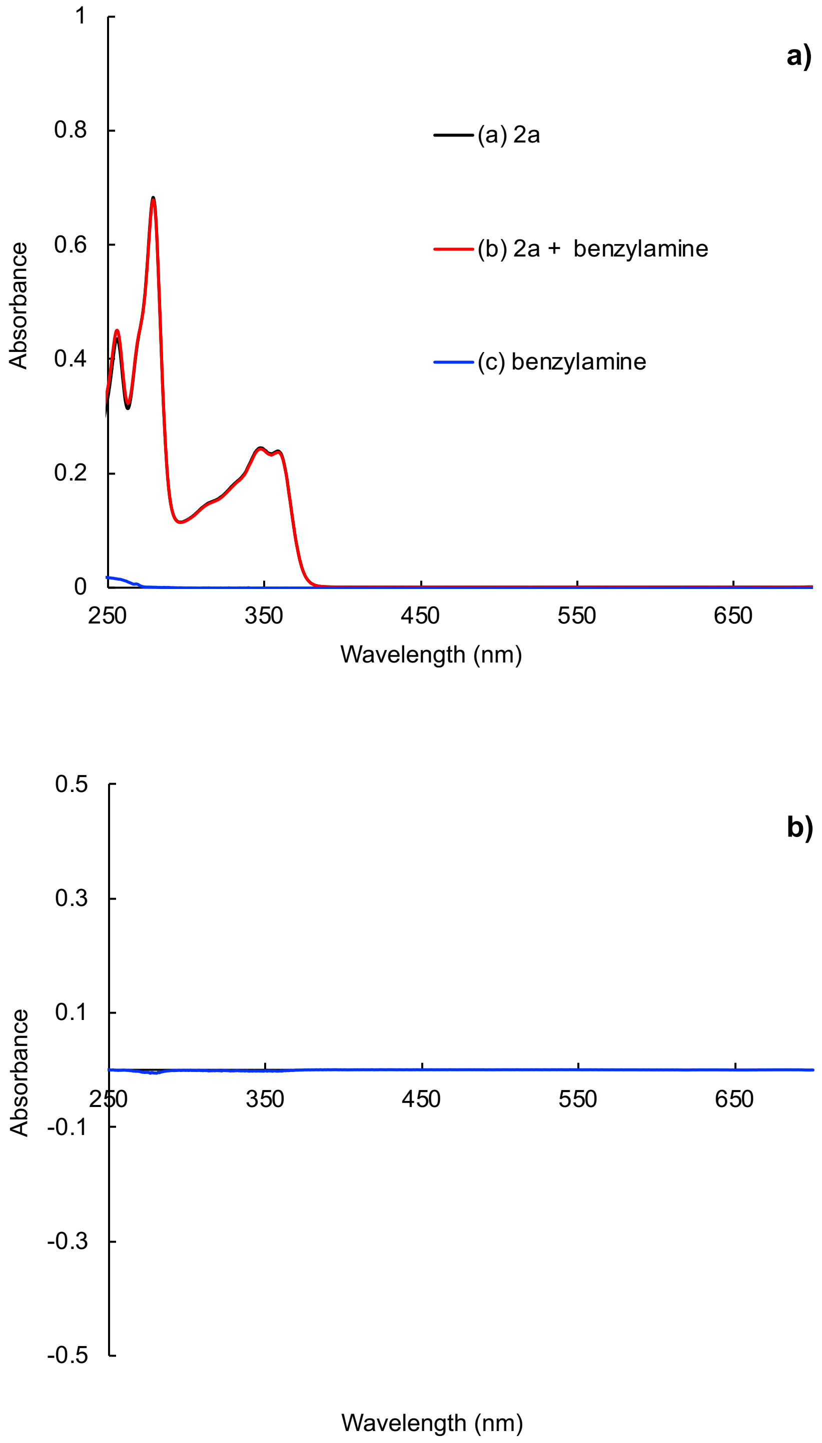

Fig. S1 Absorption spectra of 2a in the absence and presence of benzylamine.

Panel a: 2a (1 equiv, 3.0 x 10^{-5} M) in the absence (**a**) and presence (**b**) of benzylamine (1.5 equiv, 4.5 x 10^{-5} M) in acetonitrile. (**c**) Benzylamine (4.5 x 10^{-5} M). Under dark.

Panel b: Differential spectrum, (**b**) – ((**a**) + (**c**)). The differential spectrum shows almost negligible absorbance change, indicating that **2a** do not interact with benzyl amine under dark.

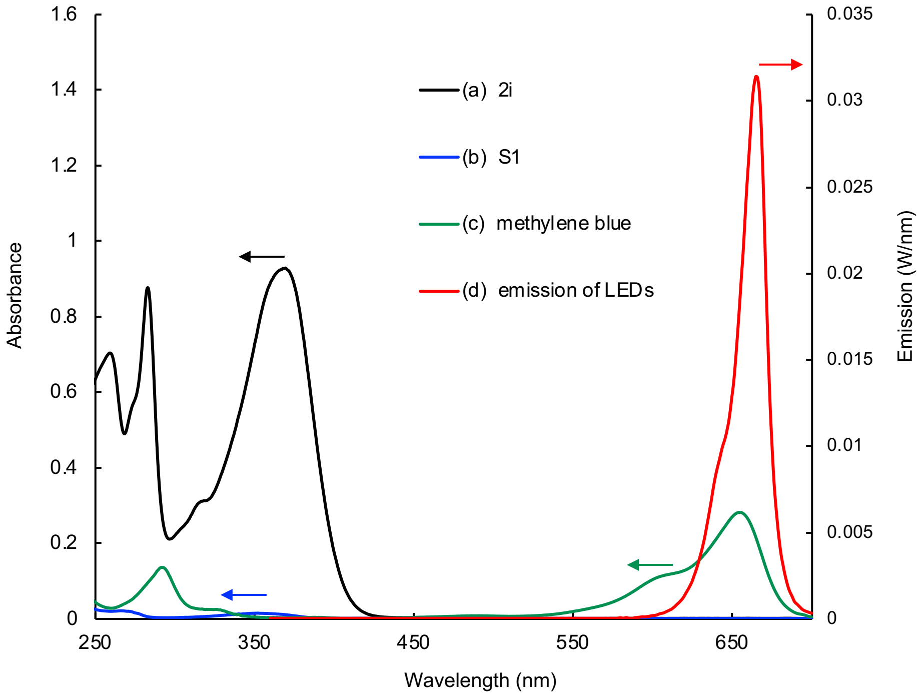

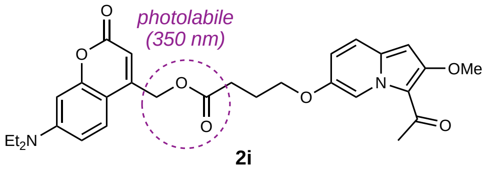

2i

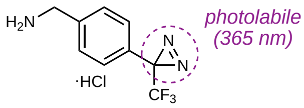

S1

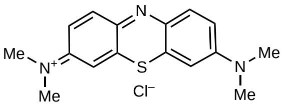

methylene blue (**PS1**)

Fig. S2 Absorption spectra of substrates and emission spectra of red LEDs.
(**a**) **2i** (3.0×10^{-5} M) in acetonitrile. (**b**) **S1** (3.0×10^{-5} M) in acetonitrile/water (39/1). (**c**) methylene blue (**PS1**, 3.0×10^{-6} M) in acetonitrile. (**d**) Emission spectra of a red LED light source (Kessil H160 Tuna Flora LED lamp, red channel) provided from Kessil®. The arrows indicate the corresponding vertical axis.

a) Labeling using $^{18}O[H_2O]$

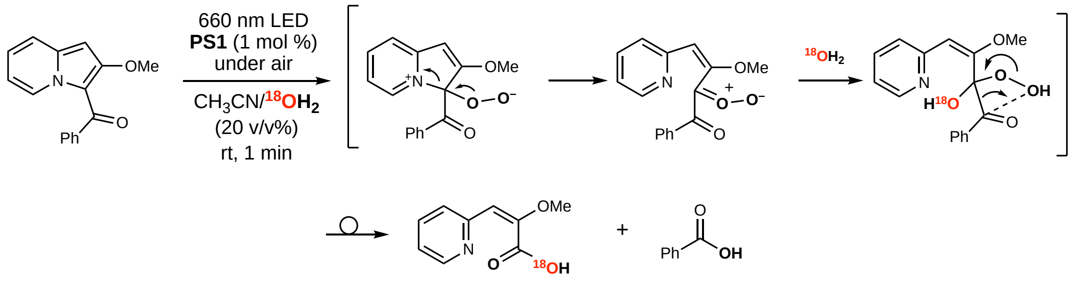

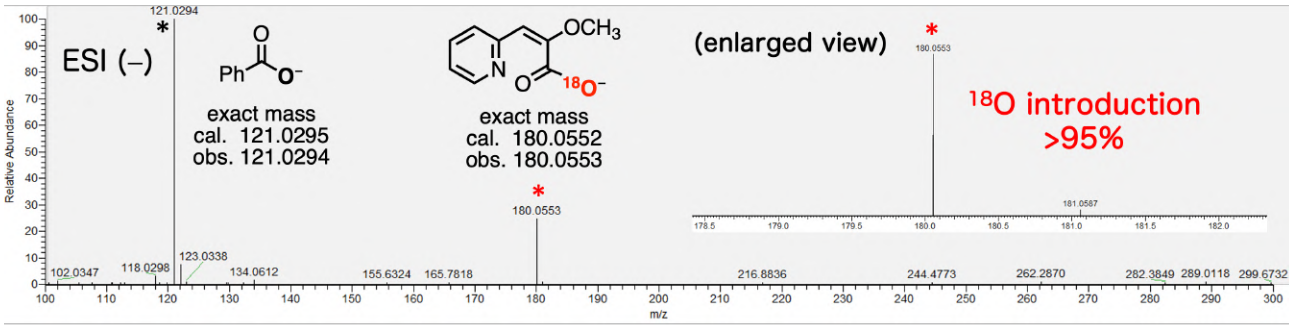

b) Labeling using $^{18}O[O_2]$

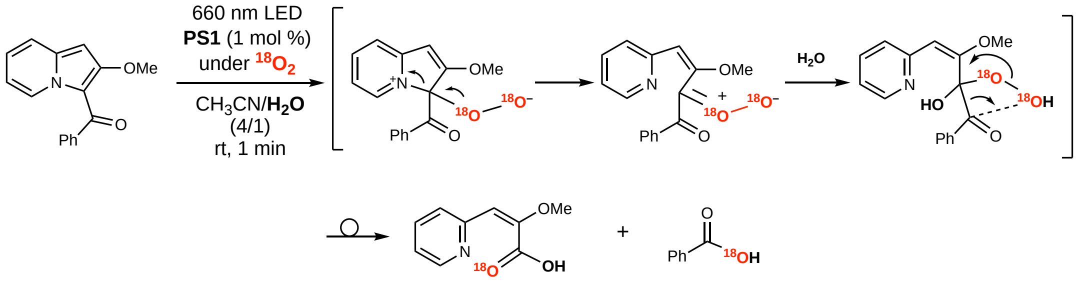

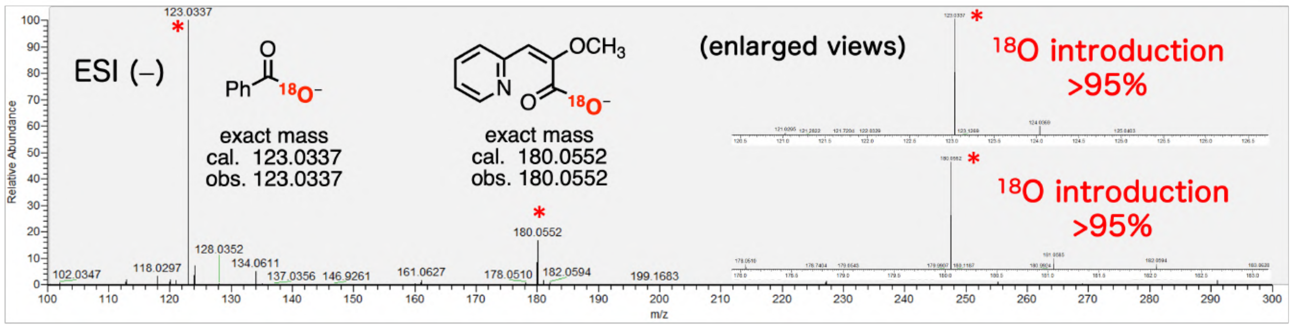

Fig. S3 ESI mass spectra for ^{18}O-labeling experiments. Labeling using $^{18}O[H_2O]$ (**a**) and $^{18}O[O_2]$ (**b**).

Table S1 Unapplicable substrates. Yield based on ^{1}H NMR measurements.

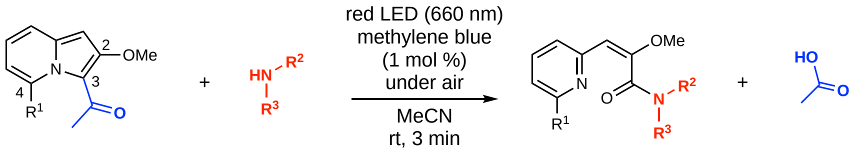

2 **1** (1.5 equiv) **3** **4a**

substrate	product	yield (%)
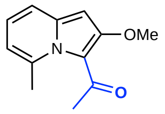	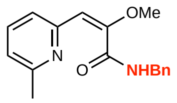	<5
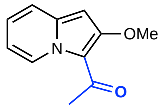	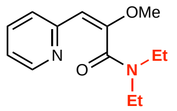	<5
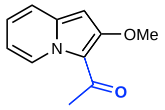	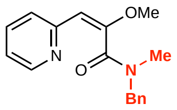	<5
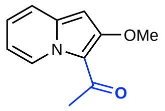	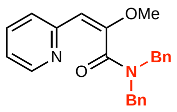	<5
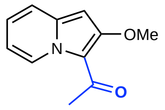	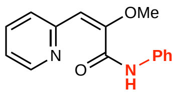	<5

Procedure

To a stirring MeCN (40 mL) solution of indolizine **2** (0.200 mmol, 1 equiv) and amine **1** (0.300 mmol, 1.5 equiv) in a 100 mL round-bottom flask was added an MeCN solution of methylene blue (1.0 mL, 2.0 mM, 2.0 μmol, 1.0 mol %) under air. The solution was vigorously stirred by a magnetic stirrer at room temperature under air and photoirradiated for 3 min. After the photoirradiation, the solvent was evaporated, and the residue was dried under vacuum. A $CDCl_3$ (2 mL) solution of CH_2Br_2 (internal standard, 13.9 μL, 0.200 mmol, 1 equiv) was added to the residue. A portion of the solution was transferred to an NMR tube, and ^{1}H NMR measurement was performed. The formation of the product was less that 5% as judged from the ^{1}H NMR measurement.

General information

All reactions for synthesis of photoreactive substrates were performed under argon atmosphere and shading from light unless otherwise indicated. The definition for room temperature (rt) is 23–27 °C. An IKA RCT basic hot plate stirrer equipped with aluminum blocks was used for heating. Analytical thin-layer chromatography (TLC) was performed on precoated (0.25 mm) silica-gel plates (Merck, Merck Silica Gel 60 F254). Column chromatography was conducted on a YAMAZEN Automated Flash Chromatography System that consists of AI-580 and Parallel Frac FR-360. Melting points (mp) were measured with an OptiMelt automated melting point apparatus (Stanford Research Systems, Inc.) and were uncorrected. ^{1}H NMR (400 MHz), ^{13}C NMR (100 MHz), and ^{19}F NMR (373 MHz) spectra were obtained from measurements at room temperature on a JEOL ECS400 spectrometer. Chloroform-d_1 ($CDCl_3$) containing 0.05% tetramethylsilane (TMS, 99.8%D, Cambridge Isotope Laboratories, Inc.), methanol-d_4 (CD_3OD, 99.8%D, Merck, Inc.), dimethyl sulfoxide-d_6 (DMSO-d_6, 99.9%D, Cambridge Isotope Laboratories), and acetonitrile-d_3 (CD_3CN, 99.8%D, Cambridge Isotope Laboratories) were used as solvents for NMR measurements. Chemical shifts (δ) for ^{1}H NMR are given in parts per million (ppm) downfield from signal of TMS (δ 0.00 ppm) for the measurements in $CDCl_3$. The residual DMSO (δ 2.50 ppm) and MeOH (δ 3.31 ppm) were used as internal standards for the ^{13}C{^{1}H} NMR measurements in DMSO-d_6 and CD_3OD, respectively. Chemical shifts (δ) for ^{19}F NMR are given in parts per million (ppm) downfield from signal of (trifluoromethyl)benzene ($PhCF_3$, δ -62.6 ppm in $CDCl_3$). The coupling constants (*J*) are given in hertz (Hz). Chemical shifts (δ) for ^{13}C NMR are given in parts per million (ppm) downfield from signal of residual $CHCl_3$ (δ 77.2 ppm), DMSO (δ 39.5 ppm), and MeOH (δ 49.0 ppm) as internal standards. The abbreviations s, d, t, q, and m signify singlet, doublet, triplet, quartet, and multiplet, respectively. IR spectra were measured by attenuated total reflection method on a Shimadzu IRPrestige-21 spectrometer with the absorption band given in cm^{-1}. Recycle gel permeation chromatography (GPC) was performed on a YMC LC-Forte/R multiple preparative HPLC system. High-resolution mass spectra (HRMS) were measured on a Thermo Fisher Scientific Exactive Plus Orbitrap mass spectrometer.

Chemicals

All purchased chemicals were used as received unless otherwise indicated. Acetonitrile (MeCN, super dehydrated), dichloromethane (CH_2Cl_2, deoxygenated), sodium chloride (NaCl), chlorotrimethylsilane, *N*,*N*-dimethylformamide (DMF, deoxygenated), tetrahydrofuran (THF, deoxygenated), isopropyl alcohol (*i*-PrOH, deoxygenated), piperidin-4-ylmethanol, and *tert*-butyl L-tyrosinate were purchased from FUJIFILM Wako Pure Chemical. Methylene blue (**PS1**), *tert*-butyl 4-bromobutanoate, dimethyl sulfate, 7-(diethylamino)-4-(hydroxymethyl)-2*H*-chromen-2-one, 3-phenylpropan-1-amine, 6-aminohexan-1-ol, 2,2-dimethoxyethan-1-amine, 2-(cyclohex-1-en-1-yl)ethan-1-amine, propargylamine, 3-azidopropan-1-amine, pyridin-3-ylmethanamine, thiophen-2-ylmethanamine, 2-(1*H*-indol-3-yl)ethan-1-amine, 2-fluoroethylamine hydrochloride, formic acid, and 4-[3-(trifluoromethyl)-3*H*-diazirin-3-yl]benzylamine hydrochloride were purchased from Tokyo Chemical Industry. Dibromomethane (CH_2Br_2), acetic acid, 4-dimethylaminopyridine (DMAP), ethyl acetate (EtOAc), sodium sulfate (Na_2SO_4), hexane, methyl chloroformate, acetic anhydride, acetyl chloride, sodium carbonate (Na_2CO_3), chloroform ($CHCl_3$), potassium carbonate (K_2CO_3), ethyl bromoacetate, cesium hydroxide monohydrate, *N*,*N*-diisopropylethylamine, sodium hydrogen carbonate ($NaHCO_3$), disodium phosphate (Na_2HPO_4), sodium dihydrogenphosphate (NaH_2PO_4), benzylamine, 1-phenylethylamine, 2-methylpropan-2-amine, (3s,5s,7s)-adamantan-1-amine, morpholine, diethylamine, aniline, sodium azide, *tert*-butyl alcohol, succinic anhydride, dibenzyl amine, *N*-methylbenzylamine, and (trifluoromethyl)benzene ($PhCF_3$) were purchased from Nacalai Tesque. 1-(3-Dimethylaminopropyl)-3-ethylcarbodiimide hydrochloride (EDC·HCl), (*S*)-2-amino-3-(4-azidophenyl)propanoic acid, and (*R*)-2-amino-3-(4-azidophenyl)propanoic acid were purchased from Watanabe Chemical Industries. *tert*-Butyl D-tyrosinate was purchased from Bachem AG. Chlorin e6 monolysine amide trisodium salt (**PS2**) was purchased from Frontier Specialty Chemicals. ^{18}O labeling reagents, $^{18}O[O_2]$ gas (>98% ^{18}O) and $^{18}O[H_2O]$ (>97% ^{18}O) were purchased from Taiyo Nippon Sanso. Oxygen gas (1%) was purchased from Fujita Oxygen. (±)-*tert*-Butyl tyrosinate and (±)-2-amino-3-(4-azidophenyl)propanoic acid were prepared by mixing their D, L isomers in 1:1 ratio.

Synthesis of substrates

2-Methoxyindolizine,[S1] (2-methoxyindolizin-3-yl)(phenyl)methanone (**2b**),[S1] phenyl 2-methoxyindolizine-3-carboxylate (**2d**),[S1] 2-(1-(4-chlorobenzoyl)-5-methoxy-2-methyl-1*H*-indol-3-yl)-1-(2-methoxyindolizin-3-yl)ethan-1-one (**2j**), 2-methoxy-1-methylindolizine,[S2] 2-methoxy-6-(prop-2-yn-1-yloxy)indolizine,[S3] 2-methylindolizine,[S3] 2-phenylindolizine,[S4] and 2-methoxy-5-methylindolizine[15] were synthesized according to the literature.

1-(2-Methoxyindolizin-3-yl)ethan-1-one (**2a**)

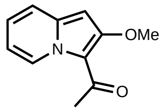

To a stirring CH_2Cl_2 (6 mL) solution of 2-methoxyindolizine (294 mg, 2.00 mmol, 1 equiv), acetic acid (180 mg, 3.00 mmol, 1.5 equiv), and DMAP (48.9 mg, 0.400 mmol, 20 mol %) in a 20 mL vial was added EDC·HCl (422 mg, 2.20 mmol, 1.1 equiv) at room temperature. The solution was stirred at 30 °C for 20 h under argon atmosphere. The reaction mixture was poured into brine (30 mL) and extracted with EtOAc (30 mL) for three times. The combined organic layer was dried with Na_2SO_4 and evaporated. The residue was purified by silica-gel column chromatography (hexane/EtOAc = 1/0 to 1/1).
Yield: 372 mg (1.97 mmol, 98.4%); Colorless solid; mp: 94.6–96.8 °C; TLC R_f = 0.41 (hexane/EtOAc = 7/3); ^{1}H NMR (400 MHz, $CDCl_3$): δ 9.93 (d, J = 7.2 Hz, 1H), 7.34 (d, J = 8.4 Hz, 1H), 7.14–7.10 (m, 1H), 6.80–6.76 (m, 1H), 6.02 (s, 1H), 3.98 (s, 3H), 2.55 (s, 3H); ^{13}C NMR (100 MHz, $CDCl_3$): δ 186.1, 159.1, 136.9, 128.8, 124.9, 116.8, 112.6, 111.8, 85.1, 58.0, 29.4; IR (ZnSe); 1597, 1504, 1450, 1420, 1252, 1022 cm^{-1}; HRMS (ESI, m/z): $[M + H]^+$ calcd. for $C_{11}H_{12}NO_2^+$, 190.0863; found 190.0863.

Methyl 2-methoxyindolizine-3-carboxylate (**2c**)

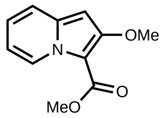

To a stirring CH_2Cl_2 (1 mL) solution of 2-methoxyindolizine (73.6 mg, 0.500 mmol, 1 equiv) in a 4 mL vial was added a CH_2Cl_2 (1 mL) solution of methyl chloroformate (70.9 mg, 0.750 mmol, 1.5 equiv) at 0 ºC under argon atmosphere. The solution was stirred at room temperature for 20 h. The reaction mixture was poured into brine (15 mL) and extracted with EtOAc (15 mL) for three times. The organic layer was dried with Na_2SO_4 and evaporated. The residue was purified by silica-gel column chromatography (hexane/EtOAc = 1/0 to 1/1).
Yield: 60.7 mg (0.296 mmol, 59.1%); Pale yellow solid; mp: 59.0–60.0 °C; TLC R_f = 0.41 (hexane/EtOAc = 7/3); ^{1}H NMR (400 MHz, $CDCl_3$): δ 9.46–9.44 (m, 1H), 7.35–7.32 (m, 1H), 7.07–7.02 (m, 1H), 6.78–6.74 (m, 1H), 6.06 (s, 1H), 3.98 (s, 3H), 3.93 (s, 3H); ^{13}C NMR (100 MHz, $CDCl_3$): δ 162.2, 157.8, 136.5, 127.8, 123.2, 117.2, 101.4, 112.0, 85.3, 58.3, 51.2 ; IR (ZnSe); 2947, 1667, 1503, 1344, 1233, 1101 cm^{-1}; HRMS (ESI, m/z): $[M + H]^+$ calcd for $C_{11}H_{12}NO_3^+$, 206.0812; found 206.0812.

1-(2-Methoxy-1-methylindolizin-3-yl)ethan-1-one (**2e**)

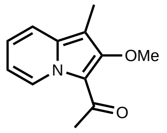

To a stirring CH_2Cl_2 (3 mL) solution of 2-methoxy-1-methylindolizine (161 mg, 0.999 mmol, 1 equiv), acetic acid (90.1 mg, 1.50 mmol, 1.5 equiv) and DMAP (24.4 mg, 0.200 mmol, 20 mol %) in a 20 mL vial was added EDC·HCl (211 mg, 1.10 mmol, 1.1 equiv) at room temperature. The solution was stirred at 30 °C for 20 h under argon atmosphere. The reaction mixture was poured into brine (15 mL) and extracted with EtOAc (15 mL) for three times. The organic layer was dried with Na_2SO_4 and evaporated. The residue was purified by silica-gel column chromatography (hexane/EtOAc = 1/0 to 2/1).

Yield: 179 mg (0.880 mmol, 88.1%); Pale yellow solid; mp: 57.1-58.7 °C; TLC R_f = 0.41 (hexane/EtOAc = 7/3); ^{1}H NMR (400 MHz, $CDCl_3$): δ 9.88–9.86 (m, 1H), 7.37–7.34 (m, 1H), 7.14–7.09 (m, 1H), 6.81–6.77 (m, 1H), 4.00 (s, 3H), 2.59 (s, 3H), 2.30 (s, 3H); ^{13}C NMR (100 MHz, $CDCl_3$): δ 186.1, 156.0, 135.6, 128.6, 123.8, 115.7, 113.5, 113.0, 100.5, 62.0, 28.4, 7.8; IR (ZnSe); 1593, 1551, 1554, 1393, 1016 cm^{-1}; HRMS (ESI, m/z): $[M + H]^+$ calcd for $C_{12}H_{14}NO_2^+$, 204.1019; found 204.1020.

1-(2-Methoxy-6-(prop-2-yn-1-yloxy)9ndolizine-3-yl)ethan-1-one (**2f**)

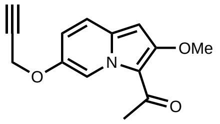

To a stirring CH_2Cl_2 (3 mL) solution of 2-methoxy-6-(prop-2-yn-1-yloxy)indolizine (201 mg, 0.999 mmol, 1 equiv), acetic acid (90.1 mg, 1.50 mmol, 1.5 equiv) and DMAP (24.4 mg, 0.200 mmol, 20 mol %) in a 20 mL vial was added EDC·HCl (211 mg, 1.10 mmol, 1.1 equiv) at room temperature. The solution was stirred at 30 °C for 20 h under argon atmosphere. The reaction mixture was poured into brine (15 mL) and extracted with EtOAc (15 mL) for three times. The organic layer was dried with Na_2SO_4 and evaporated. The residue was purified by silica-gel column chromatography (hexane/EtOAc = 1/0 to 2/1).

Yield: 226 mg (0.929 mmol, 93.0%); Colorless solid; mp: 148.3–149.5°C; TLC R_f = 0.39 (hexane/EtOAc = 2/1); ^{1}H NMR (400 MHz, $CDCl_3$): δ 9.89 (d, J = 2.4 Hz, 1H), 7.28–7.25 (m, 1H), 7.00 (dd, J = 9.6, 2.4 Hz, 1H), 5.98 (s, 1H), 4.72 (d, J = 2.4 Hz, 2H), 3.96 (s, 3H), 2.59 (t, J = 2.4 Hz, 1H), 2.54 (s, 3H); ^{13}C NMR (100 MHz, $CDCl_3$): δ 186.3, 158.6, 147.4, 133.5, 119.4, 117.1, 113.8, 112.7, 85.0, 78.1, 76.4, 57.9, 57.4, 29.3; IR (ZnSe); 1584, 1454, 1418, 1240, 1030, 802 cm^{-1}; HRMS (ESI, m/z): $[M + H]^+$ calcd for $C_{14}H_{14}NO_3^+$, 244.0968; found 244.0970.

1-(2-Methylindolizin-3-yl)ethan-1-one (**2g**)

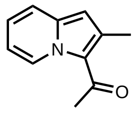

To a stirring CH_2Cl_2 (5 mL) solution of 2-methylindolizine (131 mg, 0.999 mmol, 1 equiv) in a 20 mL vial was added a CH_2Cl_2 (1 mL) solution of acetic anhydride (123 mg, 1.20 mmol, 1.2 equiv) at 0 ºC under argon atmosphere. The solution was stirred at 30 °C for 24 h. The reaction mixture was poured into brine (15 mL)

and extracted with EtOAc (15 mL) for three times. The organic layer was dried with Na_2SO_4 and evaporated. The residue was purified by silica-gel column chromatography (hexane/EtOAc = 1/0 to 2/1).

Yield: 123 mg (0.710 mmol, 71.1%); Pale yellow solid; mp: 81.5–82.8 °C; TLC R_f = 0.51 (hexane/EtOAc = 7/3); ^{1}H NMR (400 MHz, $CDCl_3$): δ 9.98 (d, J = 7.2 Hz, 1H), 7.40 (d, J = 8.8 Hz, 1H), 7.11–7.07 (m, 1H), 6.81–6.77 (m, 1H), 6.34 (s, 1H), 2.61 (s, 3H), 2.57 (s, 3H); ^{13}C NMR (100 MHz, $CDCl_3$): δ 187.0, 137.6, 134.6, 129.1, 124.1, 122.1, 117.5, 113.1, 105.4, 30.6, 17.0; IR (ZnSe); 1599, 1425, 1402, 1327, 1248, 968 cm^{-1}; HRMS (ESI, m/z): $[M + H]^+$ calcd for $C_{11}H_{12}NO^+$, 174.0913; found 174.0914.

1-(2-Phenylindolizin-3-yl)ethan-1-one (**2h**)

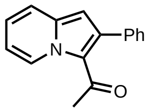

To a stirring THF (15 mL) solution of 2-phenylindolizine (193 mg, 0.999 mmol, 1 equiv) in a 20 mL vial was added a THF (1 mL) solution of acetyl chloride (118 mg, 1.50 mmol, 1.5 equiv) at 0 ºC under argon atmosphere. The solution was stirred at room temperature for 20 h. The reaction mixture was poured into brine (15 mL) and extracted with EtOAc (15 mL) for three times. The organic layer was dried with Na_2SO_4 and evaporated. The residue was purified by silica-gel column chromatography (hexane/EtOAc = 1/0 to 1/1).

Yield: 167 mg (0.710 mmol, 71.1%); Pale yellow oil; TLC R_f = 0.57 (hexane/EtOAc = 7/3); ^{1}H NMR (400 MHz, $CDCl_3$): δ 10.00–9.98 (m, 1H), 7.52–7.49 (m, 1H), 7.47–7.39 (m, 5H), 7.19–7.14 (m, 1H), 6.90–6.86 (m, 1H), 6.48 (s, 1H), 2.05 (s, 3H); ^{13}C NMR (100 MHz, $CDCl_3$): δ 188.5, 139.7, 137.5, 137.1, 129.9, 129.0, 128.4, 127.9, 124.4, 121.3, 118.2, 113.8, 105.1, 30.3; IR (ZnSe); 1612, 1601, 1539, 1501, 1402 cm^{-1}; HRMS (ESI, m/z): $[M + H]^+$ calcd for $C_{16}H_{14}NO^+$, 236.1070; found 236.1071.

1-(2-Methoxy-5-methylindolizin-3-yl)ethan-1-one

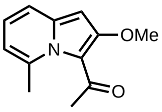

To a stirring CH_2Cl_2 (3 mL) solution of 2-methoxy-5-methylindolizine (161 mg, 0.999 mmol, 1 equiv), acetic acid (90.1 mg, 1.50 mmol, 1.5 equiv) and DMAP (24.4 mg, 0.200 mmol, 20 mol %) in a 20 mL vial was added EDC·HCl (211 mg, 1.10 mmol, 1.1 equiv) at room temperature. The solution was stirred at 30 °C for 20 h under argon atmosphere. The reaction mixture was poured into brine (15 mL) and extracted with EtOAc (15 mL) for three times. The organic layer was dried with Na_2SO_4 and evaporated. The residue was purified by silica-gel column chromatography (hexane/EtOAc = 1/0 to 2/1).

Yield: 178 mg (0.878 mmol, 87.9%); Pale yellow solid; mp: 60.3–61.9 °C; TLC R_f = 0.60 (hexane/EtOAc = 7/3); ^{1}H NMR (400 MHz, CD_3OD): δ 7.39 (d, J = 8.8 Hz, 1H), 7.25 (dd, J = 8.8, 7.2 Hz, 1H), 6.79–6.77 (m, 1H), 6.28 (s, 1H), 4.02 (s, 3H), 2.54 (s, 3H), 2.43 (s, 3H); ^{13}C NMR (100 MHz, CD_3OD): δ 186.0, 162.0, 142.0, 141.5, 127.2, 116.0, 115.8, 114.8, 86.8, 58.6, 29.1, 23.3; IR (ZnSe); 1620, 1537, 1516, 1447, 1358, 1323 cm^{-1}; HRMS (ESI, m/z): $[M + H]^+$ calcd for $C_{12}H_{14}NO_2^+$, 204.1019; found 204.1018.

Methyl 2-amino-3-(4-azidophenyl)propanoate

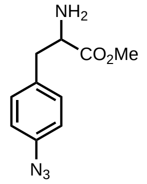

To a stirring MeOH (15 mL) solution of 2-amino-3-(4-azidophenyl)propanoic acid (309 mg, 1.50 mmol, 1 equiv) in a 20 mL vial was added chlorotrimethylsilane (1.63 g, 15.0 mmol, 10 equiv) at room temperature. The solution was stirred at room temperature for 20 h under argon atmosphere. The solution was evaporated. The residue was extracted from saturated Na_2CO_3 aqueous solution (60 mL) and $CHCl_3$ (60 mL) for three times. The organic layer was dried with Na_2SO_4 and evaporated.
Yield: 269 mg (1.22 mmol, 81.5%); Pale yellow oil; ^{1}H NMR (400 MHz, $CDCl_3$): δ 7.20–7.16 (AA′BB′, 2H), 6.99–6.96 (AA′BB′, 2H), 3.72–3.69 (m, 4H), 3.08–3.03 (dd, J = 13.6, 5.2 Hz, 1H), 2.89–2.83 (dd, J = 13.8, 8.0 Hz, 1H); ^{13}C NMR (100 MHz, $CDCl_3$): δ 175.5, 138.8, 134.1, 130.8, 119.3, 56.0, 52.2, 40.5; IR (ZnSe); 2951, 2112, 1732, 1605, 1504, 1285 cm^{-1}; HRMS (ESI, m/z): [M + H]$^+$ calcd for $C_{10}H_{13}N_4O_2^+$, 221.1033; found 221.1029.

tert-Butyl 4-((6-methylpyridin-3-yl)oxy)butanoate

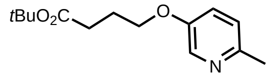

To a stirring DMF (20 mL) solution of 6-methylpyridin-3-ol (2.18 mg, 20.0 mmol, 1 equiv) in a 100 mL round-bottom flask was added K_2CO_3 (2.76 g, 20.0 mmol, 1 equiv) at room temperature. The solution was stirred at room temperature for 30 min under argon atmosphere. The solution was cooled to 0 °C, and *tert*-butyl 4-bromobutanoate (4.46 g, 20.0 mmol, 1 equiv) was added to the solution. The solution was stirred at room temperature for 22 h under argon atmosphere. The reaction mixture was poured into brine (100 mL) and extracted with EtOAc (100 mL) for three times. The organic layer was dried with Na_2SO_4 and evaporated. The residue was purified by silica-gel column chromatography (hexane/EtOAc = 1/0 to 1/4).
Yield: 3.85 g (15.3 mmol, 76.7%); Colorless oil; TLC R_f = 0.59 (hexane/EtOAc = 1/1); ^{1}H NMR (400 MHz, $CDCl_3$): δ 8.17 (d, J = 2.8 Hz, 1H), 7.11–6.99 (m, 2H), 4.01 (t, J = 6.0 Hz, 2H), 2.48 (s, 3H), 2.42 (t, J = 7.2 Hz, 2H), 2.10–2.03 (m, 2H), 1.45 (s, 9H); ^{13}C NMR (100 MHz, $CDCl_3$): δ 172.6, 153.2, 150.6, 137.0, 123.5, 122.1, 80.7, 67.5, 32.0, 28.3, 24.9, 23.5; IR (ZnSe); 2976, 1726, 1485, 1366, 1263, 1150 cm^{-1}; HRMS (ESI, m/z): [M + H]$^+$ calcd for $C_{14}H_{22}NO_3^+$, 252.1594; found 252.1594.

5-(4-(tert-Butoxy)-4-oxobutoxy)-1-(2-ethoxy-2-oxoethyl)-2-methylpyridin-1-ium bromide

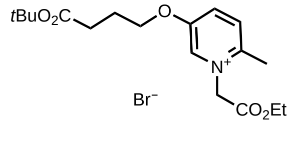

To a stirring THF (10 mL) solution of *tert*-Butyl 4-((6-methylpyridin-3-yl)oxy)butanoate (2.67 g, 10.6 mmol, 1 equiv) was added ethyl bromoacetate (1.77 g, 10.6 mmol, 1 equiv) at room temperature. The solution was stirred at 80 °C for 21 h under argon atmosphere. After cooling to room temperature, the reaction mixture was poured into hexane (30 mL) to form a precipitate. The precipitate was collected by filtration, washed with hexane (30 mL × 3), and dried in vacuum.

Yield: 3.32 g (7.93 mmol, 74.8%); White solid; mp: 141.0–142.1 °C; ^{1}H NMR (400 MHz, $CDCl_3$): δ 9.80 (d, J = 2.8 Hz, 1H), 7.85–7.82 (m, 1H), 7.62–7.60 (m, 1H), 6.17 (s, 2H), 4.45 (t, J = 6.4 Hz, 2H), 4.33 (q, J = 7.2 Hz, 2H), 2.69 (s, 3H), 2.44 (t, J = 7.6 Hz, 2H), 2.17–2.10 (m, 2H), 1.45 (s, 9H), 1.36 (t, J = 7.6 Hz, 3H); ^{13}C NMR (100 MHz, $CDCl_3$): δ 172.3, 165.7, 156.5, 147.8, 134.2, 134.1, 129.4, 80.8, 71.0, 63.6, 59.3, 31.9, 28.3, 24.4, 20.0, 14.3; IR (ZnSe); 3001, 2852, 1740, 1715, 1368, 1225, 1159 cm^{-1}; HRMS (ESI, m/z): $[M + H]^+$ calcd for $C_{18}H_{28}NO_5^+$, 338.1962; found 338.1960.

tert-Butyl 4-((2-methoxyindolizin-6-yl)oxy)butanoate

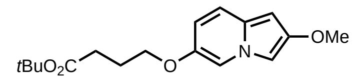

5-(4-(*tert*-Butoxy)-4-oxobutoxy)-1-(2-ethoxy-2-oxoethyl)-2-methylpyridin-1-ium bromide (4.18 g, 9.99 mmol, 1 equiv), DMF (50 mL), and *i*PrOH (50 mL) were added into a 300 mL round-bottom flask. The flask was filled with argon and set to an ice water bath. Cesium hydroxide monohydrate (3.36 g, 20.0 mmol, 2 equiv) was added to the solution in one portion. The resulting mixture was stirred at room temperature for 30 min under argon atmosphere. Dimethyl sulfate (1.26 g, 9.99 mmol, 1 equiv) was added dropwise to the mixture for 5 min at 0 °C. The resulting mixture was stirred at room temperature for 2 h under argon atmosphere. The mixture was then poured into a solution of hexane (300 mL)/EtOAc (150 mL), and washed with brine (300 mL × 3). The organic layer was dried with Na_2SO_4 and evaporated. The residue was purified by silica-gel column chromatography (hexane/EtOAc = 1/0 to 4/1).

Yield: 468 mg (1.53 mmol, 15.3%); Brown solid; mp: 48.4–50.0 °C; TLC R_f = 0.34 (hexane/EtOAc = 4/1); ^{1}H NMR (400 MHz, $CDCl_3$): δ 7.43–7.41 (m, 1H), 7.12–7.10 (m, 1H), 6.93–6.92 (m, 1H), 6.51–6.48 (m, 1H), 6.01–6.00 (m, 1H), 3.90 (t, J = 6.4 Hz, 2H), 3.82 (s, 3H), 2.43 (t, J = 7.2 Hz, 2H), 2.09–2.01 (m, 2H), 1.45 (s, 9H); ^{13}C NMR (100 MHz, $CDCl_3$): δ 172.7, 152.7, 146.8, 128.3, 118.3, 113.1, 109.0, 98.0, 85.6, 80.6, 68.3, 57.9, 32.2, 28.3, 25.0; IR (ZnSe); 2932, 1730, 1557, 1368, 1319, 1246, 1150, 793 cm^{-1}; HRMS (ESI, m/z): $[M + H]^+$ calcd for $C_{17}H_{24}NO_4^+$, 306.1700; found 306.1698.

tert-Butyl 4-((3-acetyl-2-methoxyindolizin-6-yl)oxy)butanoate

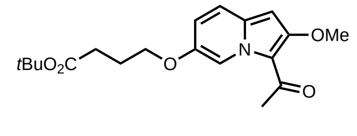

To a stirring CH_2Cl_2 (1.2 mL) solution of *tert*-butyl 4-((2-methoxyindolizin-6-yl)oxy)butanoate (122 mg, 0.400 mmol, 1 equiv), acetic acid (36.0 mg, 0.600 mmol, 1.5 equiv) and DMAP (9.8 mg, 0.080 mmol, 20 mol %) in a 4 mL vial was added EDC·HCl (84.3 mg, 0.440 mmol, 1.1 equiv) at room temperature. The solution was stirred at 30 °C for 20 h under argon atmosphere. The reaction mixture was poured into brine (15 mL) and extracted with EtOAc (15 mL) for three times. The organic layer was dried with Na_2SO_4 and evaporated. The residue was purified by silica-gel column chromatography (hexane/EtOAc = 1/0 to 1/1).

Yield: 126 mg (0.363 mmol, 90.8%); Pale brown solid; mp: 95.2–96.2 °C; TLC R_f = 0.32 (hexane/EtOAc = 7/3); ^{1}H NMR (400 MHz, $CDCl_3$): δ 9.72–7.71 (m, 1H), 7.25–7.22 (m, 1H), 6.95–6.92 (m, 1H), 5.94 (s, 1H), 4.01 (t, J = 6.4 Hz, 2H), 3.95 (s, 3H), 2.53 (s, 3H), 2.43 (t, J = 7.2 Hz, 2H), 2.12–2.05 (m, 2H), 1.45 (s, 9H); ^{13}C NMR (100 MHz, $CDCl_3$): δ 186.1, 172.6, 158.4, 148.7, 133.2, 119.5, 117.0, 112.7, 112.6, 84.7, 80.6, 68.1,

57.9, 32.3, 29.3, 28.3, 24.9; IR (ZnSe); 2932, 1713, 1595, 1510, 1454, 1418, 1238 cm^{-1}; HRMS (ESI, m/z): $[M + H]^+$ calcd for $C_{19}H_{26}NO_5^+$, 348.1805; found 348.1803.

(7-(diethylamino)-2-oxo-2H-chromen-4-yl)methyl 4-((3-acetyl-2-methoxyindolizin-6-yl)oxy)butanoate (**2i**)

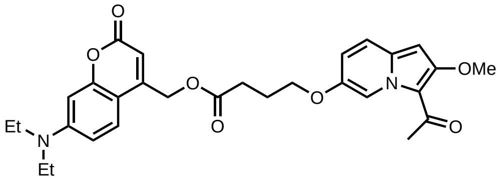

To a stirring CH_2Cl_2 (2.0 mL) solution of *tert*-butyl 4-((3-acetyl-2-methoxyindolizin-6-yl)oxy)butanoate (41.7 mg, 0.120 mmol, 1 equiv) in a 20 mL vial was added trifluoracetic acid (0.5 mL) at 0 °C. The solution was stirred at room temperature for 2 h under argon atmosphere. The solution was evaporated, and the residue was dried under vacuum. Then, CH_2Cl_2 (1.0 mL), *N*,*N*-diisopropylethylamine (105 µL, 0.603 mmol, 5 equiv), DMAP (14.7 mg, 0.120 mmol, 1 equiv), 7-(diethylamino)-4-(hydroxymethyl)-2*H*-chromen-2-one (44.5 mg, 0.180 mmol, 1.5 equiv) and EDC·HCl (27.6 mg, 0.144 mmol, 1.2 equiv) were added to the residue. The resulting solution was stirred at 30 °C for 20 h under argon atmosphere. The reaction mixture was poured into brine (15 mL) and extracted with EtOAc (15 mL) for three times. The organic layer was dried with Na_2SO_4 and evaporated. The residue was purified by silica-gel column chromatography (hexane/EtOAc = 1/0 to 0/1). The product was further purified by recycle gel permeation chromatography (GPC, solvent $CHCl_3$).

Yield: 58.2 mg (0.0112 mmol, 93.1%); Yellow solid; mp: 55.1–58.4 °C; TLC R_f = 0.55 (hexane/EtOAc = 1:2); ^{1}H NMR (400 MHz, $CDCl_3$): δ 9.73 (s, 1H), 7.31–7.23 (m, 2H), 6.95–6.92 (m, 1H), 6.58–6.56 (m, 1H), 6.51 (d, J = 2.4 Hz, 1H), 6.13 (s, 1H), 5.95 (s, 1H), 5.24 (d, J = 1.6 Hz, 2H), 4.07 (t, J = 6.0 Hz, 2H), 3.95 (s, 3H), 3.41 (q, J = 6.8 Hz, 4H), 2.68 (t, J = 7.2 Hz, 2H), 2.53 (s, 3H), 2.23–2.19 (m, 2H), 1.20 (t, J = 6.8 Hz, 6H); ^{13}C NMR (100 MHz, $CDCl_3$): δ 186.1, 172.5, 162.0, 158.4, 156.5, 150.8, 149.5, 148.6, 133.3, 124.7, 119.4, 117.1, 112.7, 112.6, 109.0, 107.0, 106.4, 98.1, 84.9, 67.8, 61.7, 57.9, 45.0, 30.9, 29.3, 24.7, 12.6; IR (ZnSe); 2971, 1712, 1599, 1146, 1271, 1140, 989 cm^{-1}; HRMS (ESI, m/z): $[M + H]^+$ calcd for $C_{29}H_{33}N_2O_7^+$, 521.2282; found 521.2281.

4-(2-methoxyindolizin-3-yl)-4-oxobutanoic acid (**2k**)

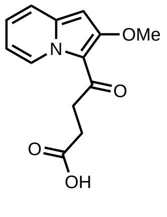

To a stirring CH_2Cl_2 (2.0 mL) solution of 2-methoxyindolizine (88.3 mg, 0.600 mmol, 1.5 equiv), succinic anhydride (40.0 mg, 0.400 mmol, 1 equiv) in a 4 mL vial was added DMAP (9.8 mg, 0.080 mmol, 20 mol %) at room temperature. The solution was stirred at 40 °C for 24 h under argon atmosphere. The reaction mixture was evaporated. The residue was purified by silica-gel column chromatography ($CHCl_3$/MeOH + 1 v/v% formic acid = 1/0 to 4/1).

Yield: 73.1 mg (0.297 mmol, 74.0%); Pale yellow solid; mp: 144.0–147.4 °C); TLC R_f = 0.37 ($CHCl_3$/MeOH + 1 v/v% formic acid = 10/1); ^{1}H NMR (400 MHz, DMSO-d_6): δ 12.02 (s, 1H), 9.77 (d, J = 8.0 Hz, 1H), 7.57–

7.55 (m, 1H), 7.27–7.22 (m, 1H), 6.94–6.90 (m, 1H), 6.33 (s, 1H), 3.98 (s, 3H), 3.07 (t, J = 6.8 Hz, 2H), 2.56 (t, J = 6.8 Hz, 2H); ^{13}C NMR (100 MHz, DMSO-d_6): δ 185.4, 174.3, 158.3, 136.3, 127.3, 124.9, 117.3, 112.8, 110.2, 85.9, 58.2, 34.9, 27.9; IR (ZnSe); 2941, 1697, 1595, 1470, 1423, 1250, 1082 cm^{-1}; HRMS (ESI, m/z): [M + H]$^+$ calcd for $C_{13}H_{14}NO_4^+$, 248.0917; found 248.0912.

Procedures for photoreactions

General photoreaction procedures

To a stirring MeCN (40 mL) solution of an indolizine (0.200 mmol, 1 equiv) and an amine (0.300 mmol, 1.5 equiv) in a 100 mL round-bottom flask was added an MeCN (1 mL) solution of methylene blue (2.0 mM, 2.0 mmol, 1.0 mol %) under air. The solution was vigorously stirred by a magnetic stirrer at room temperature under air and photoirradiated for 3 min. The photoreactions were performed by using a Kessil H160 Tuna Flora LED lamp (40 W, maximum irradiation of a red channel) as a light source without filter. The distance from the light source to the irradiation reaction flask was ca. 1 cm. The photoreaction setup is shown in Fig. S4. After the photoirradiation, the solvent was evaporated, and the residue was dried under vacuum. The residue was purified by silica-gel column chromatography ($CHCl_3$/MeOH = 1/0 to 95/5) to afford an amide.

Fig. S4 Photoreaction setup. (Caution!!) Protective eyewear for red light should be use.

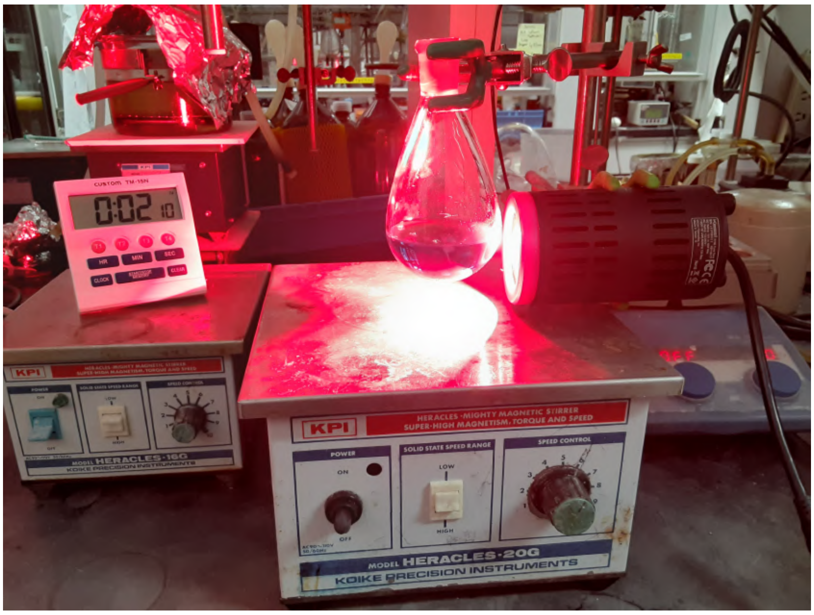

Procedure for control experiments (**Table 2**)

To an acetonitrile-d_3 (700 μL) solution of **2a** (5.0 mM, 1 equiv), benzylamine (7.5 mM, 1 equiv) and *tert*-butyl alcohol (5.0 mM, 1 equiv, internal standard) in a 4 mL vial were added an acetonitrile-d_3 (7.0 μL) solution of a photosensitizer (5.0 mM, 1.0 mol %; methylene blue (**PS1**) for entries 1, 2, 3, and 5; chlorin e6 derivative (**PS2**) for entries 6 and 7), additives (water-d_2 (140 μL) for entry 6; sodium azide (0.25 M,10 equiv) in water-d_2 (140 μL) for entries 5 and 7) and a magnetic stir bar under air. The solution was vigorously stirred by a magnetic stirrer at room temperature and photoirradiated for 3 min (660 nm LED for entries 1, 3, 4, 5, 6 and 7; 370 nm LED for entry 8). The photoreaction setup is shown in Fig. S5. After the photoirradiation, the resulting solution was subjected to ^{1}H NMR measurement. The reaction yield was determined by comparing the integration of the ^{1}H NMR peaks of the product (**3a**) and that of *tert*-butyl alcohol. For entry 3, acetonitrile-d_3 degassed by argon purging (30 min) was used as a solvent and the photoreaction was performed under argon atmosphere.

Fig. S5 Photoreaction setup for <1 mL scale. The photoreactions were performed in EvoluChem™ PhotoRedOx Box (HepatoChem).

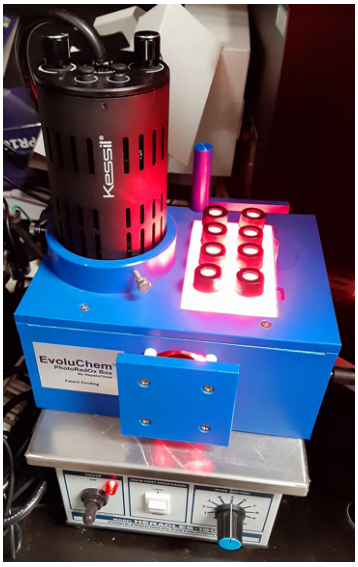

Procedure for photoreactions in aqueous solutions (**Table 3**)

To a water (280 μL) solution of **2k** (5.0 mM, 1 equiv), propargylamine (25 mM, 1 equiv) in a 4 mL vial were added a buffer (420 μL; water for entry 1, pH 7.4 sodium phosphate (1.0 M) for entry 2, pH 8.5 sodium phosphate (1.0 M) for entry 3), a water (35 μL) solution of methylene blue (**PS1**, 2.0 mM, 5.0 mol %) and a magnetic stir bar under air. The solution was vigorously stirred by a magnetic stirrer at room temperature and photoirradiated with 660 nm LED for 5 min. The photoreaction setup is shown in Fig. S5. After the photoirradiation, the resulting solution was extracted with a $CDCl_3$ (1.0 mL) solution of CH_2Br_2 (1.4 mM, 1 equiv). The organic layer was subjected to ^{1}H NMR measurement. The reaction yield was determined by comparing the integration of the ^{1}H NMR peaks of the product (**3p**) and that of CH_2Br_2.

Procedure for conjugation with photolabile diazirine ring (**Fig. 4b**)

A CD_3OD solution of **2a** (500 μL, 7.50 mM, 3.75 μmol, 1.5 equiv), a CD_3OD solution of 4-[3-(trifluoromethyl)-3*H*-diazirin-3-yl]benzylamine hydrochloride (50.0 μL, 50.0 mM, 2.50 μmol, 1 equiv), a D_2O solution of Na_2CO_3 (50.0 μL, 50.0 mM, 2.50 μmol, 1.5 equiv), a CD_3OD solution of methylene blue (50.0 μL, 5.0 mM, 25 nmol, 1.0 mol %) and a CD_3OD solution of $PhCF_3$ (internal standard, 50.0 μL, 50.0 mM, 2.50 μmol, 1 equiv) were combined. The solution was vigorously stirred by a magnetic stirrer at room temperature under air and photoirradiated for 3 min with 660 nm LED. The photoreaction setup is shown Fig. S4. Subsequently, the solution was photoirradiated for 8 min with 365 nm UV lamp (UVP UVGL-58, 6 W). After the photoirradiation, a portion of the solution was transferred to an NMR tube, and ^{19}F NMR measurement was performed. The reaction yield (%) was determined to be 81% by comparison of the integral values of the peaks corresponding to the product **6** and that of $PhCF_3$ as the internal standard. The authentic product was prepared according to the following procedure.

(E)-2-Methoxy-3-(pyridin-2-yl)-N-(4-(2,2,2-trifluoro-1-(methoxy-d₃)ethyl-1-d)benzyl)acrylamide (**6**)

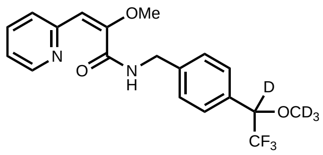

A CD_3OD (2.0 mL) solution of (*E*)-2-Methoxy-3-(pyridin-2-yl)-*N*-(4-(3-(trifluoromethyl)-3*H*-diazirin-3-yl)benzyl)acrylamide (**3w**, 37.6 mg, 0.999 mmol, 1 equiv) was stirred at 80 °C for 48 h under argon atmosphere. The solution was evaporated. The residue was purified by silica-gel column chromatography ($CHCl_3$/MeOH = 1/0 to 95/5).

Yield: 35.0 mg (91.1 μmol, 91.1%); Pale yellow solid; mp: 95.3–96.8 °C; TLC R_f = 0.53 ($CHCl_3$/MeOH = 10/1); ^{1}H NMR (400 MHz, $CDCl_3$): δ 9.01 (s, 1H), 8.25–8.23 (m, 1H), 7.63–7.58 (m, 1H), 7.42–7.34 (m, 5H), 7.09–7.05 (m, 1H), 6.05 (s, 1H), 4.54 (d, J = 5.6 Hz, 2H), 3.80 (s, 3H); ^{13}C NMR (100 MHz, $CDCl_3$): δ 162.8, 153.7, 152.5, 147.8, 139.7, 136.8, 131.7, 128.6, 128.5, 125.2, 123.9 (q, $^1J_{C\text{-}F}$ = 282.7 Hz), 121.7, 106.6, 56.0, 43.5, 29.9–29.3 (m), carbon of OCD_3 group was not observed; ^{19}F NMR (373 MHz, $CDCl_3$): δ –76.7; IR (ZnSe); 1665, 1628, 1564, 1435, 1302, 1163 cm^{-1}; HRMS (ESI, m/z): [M + H]$^+$ calcd for $C_{19}H_{16}D_4F_3N_2O_2^+$, 385.1672; found 385.1664.

Procedure for simultaneous amide conjugation and release of indomethacin (**Fig. 5**)

To an MeCN (4.0 mL) solution of **2j** (5.0 mM, 1 equiv) and *tert*-butyl L-tyrosinate (7.5 mM, 1.5 equiv) in a 30 mL round-bottom flask were added an MeCN (0.10 mL) solution of methylene blue (2.0 mM, 1.0 mol %) and a magnetic stir bar under air. The solution was vigorously stirred by a magnetic stirrer at room temperature and photoirradiated for 3 min. After the photoirradiation, a DMSO (4.0 mL) solution of 1-naphthol (5.0 mM, 1 equiv) was added, and a portion of the resulting solution was subjected to HPLC analysis. The peak areas of the products (**3m** and indomethacin) were calibrated against that of 1-naphthol as an internal standard (R^2 >0.99). The reaction yields (%) were determined by comparison of the peak areas of **3m**, indomethacin and 1-naphthol. The conditions for HPLC analysis are as below: Column: COSMOSIL C18-MS-II, 4.6 × 100 mm (Nacalai Tesque); Mobile phase: A = MeCN, B = aqueous HCO_2H (40 mM); Gradient method: A/B = 5/95 to 95/5 (0–30 min); Flow rate: 1.0 mL/min.

Procedure for ^{18}O-labeling using [^{18}O]H_2O (**Fig. S3A**)

To a solution of **2b** in MeCN (2.5 mM, 400 μL) in a 4 mL vial were added ^{18}O water (100 μL), an MeCN solution of methylene blue (1.0 mM, 10 μL, 1.0 mol %), and a magnetic stir bar under air. The solution was vigorously stirred by a magnetic stirrer at room temperature and photoirradiated for 60 sec. The photoreaction setup is shown in Fig. S5. After the photoirradiation, a portion of the resulting solution was subjected to ESI mass analysis (negative mode).

Procedure for ^{18}O-labeling using [^{18}O]H_2O (**Fig. S3B**)

To a solution of **2b** in MeCN (2.5 mM, 800 μL) in a 4 mL vial were added ultrapure water (200 μL), an MeCN solution of methylene blue (1.0 mM, 20 μL, 1.0 mol %), and a magnetic stir. The solution was degassed by 3 cycles of freeze-pump-thaw. The solution was bubbled with ^{18}O oxygen gas for 10 min. The solution was

vigorously stirred by a magnetic stirrer at room temperature and photoirradiated for 60 sec. The photoreaction setup is shown in Fig. S5. After the photoirradiation, a portion of the resulting solution was subjected to ESI mass analysis (negative mode).

(E)-N-Benzyl-2-methoxy-3-(pyridin-2-yl)acrylamide (**3a**)

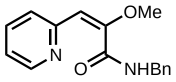

The tittle compound (**3a**) was synthesized from **2a** (37.8 mg, 0.200 mmol) and benzylamine (32.1 mg, 0.300 mmol) according to the general procedure.

Yield: 52.7 mg (0.196 mmol, 98.3%); Pale yellow solid; mp: 68.1–69.6 °C; TLC R_f = 0.53 ($CHCl_3$/MeOH = 9/1); ^{1}H NMR (400 MHz, $CDCl_3$): δ 8.75 (s, 1H), 8.30 (d, J = 4.0 Hz, 1H), 7.63–7.58 (m, 1H), 7.41 (d, J = 8.4 Hz, 1H), 7.35–7.28 (m, 5H), 7.10–7.06 (m, 1H), 6.05 (s, 1H), 4.52 (d, J = 5.6 Hz, 2H), 3.79 (s, 3H); ^{13}C NMR (100 MHz, $CDCl_3$): δ 162.8, 154.0, 152.4, 148.2, 138.0, 136.5, 128.8, 128.3, 127.6, 125.0, 121.5, 106.9, 56.0, 43.9; IR (ZnSe); 2932, 1991, 1624, 1557, 1192, 742, 698 cm^{-1}; HRMS (ESI, m/z): [M + H]$^+$ calcd for $C_{16}H_{17}N_2O_2^+$, 269.1285; found 269.1284.

The tittle compound (**3a**) was also synthesized from **2b** (50.3 mg, 0.200 mmol), **2c** (41.0 mg, 0.200 mmol) and **2d** (53.5 mg, 0.200 mmol) in 46.2 mg (0.172 mmol, 86.0%), 47.7 mg (0.178 mmol, 88.8%), 46.2 mg (0.172 mmol, 86.0%) yields, respectively. The ^{1}H NMR spectra of the products were in accordance.

(E)-N-Benzyl-2-methoxy-3-(pyridin-2-yl)but-2-enamide (**3b**)

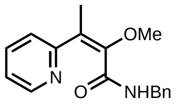

The tittle compound (**3b**) was synthesized from **2e** (40.6 mg, 0.200 mmol, 1 equiv) and benzylamine (32.1 mg, 0.300 mmol, 1.5 equiv) according to the general procedure.

Yield: 50.6 mg (0.179 mmol, 89.7%); Pale yellow solid; mp: 79.9–82.0°C; TLC R_f = 0.60 ($CHCl_3$/MeOH = 9/1); ^{1}H NMR (400 MHz, $CDCl_3$): δ 8.50–8.48 (m, 1H), 7.60–7.55 (m, 1H), 7.29–7.20 (m, 4H), 7.14–7.08 (m, 3H), 6.51 (s, 1H), 4.33 (d, J = 6.0 Hz, 2H), 3.70 (s, 3H), 2.13 (s, 3H); ^{13}C NMR (100 MHz, $CDCl_3$): δ 163.6, 159.1, 149.2, 147.7, 137.8, 136.4, 128.8, 128.3, 128.1, 127.7, 123.3, 122.1, 58.9, 43.6, 17.5; IR (ZnSe); 2936, 1632, 1585, 1514, 1454, 1219, 1084 cm^{-1}; HRMS (ESI, m/z): [M + H]$^+$ calcd for $C_{17}H_{19}N_2O_2^+$, 283.1441; found 283.1441.

(E)-N-Benzyl-2-methoxy-3-(5-(prop-2-yn-1-yloxy)pyridin-2-yl)acrylamide (**3c**)

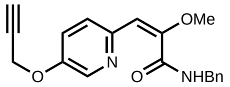

The tittle compound (**3c**) was synthesized from **2f** (48.7 mg, 0.200 mmol, 1 equiv) and benzylamine (32.1 mg, 0.300 mmol, 1.5 equiv) according to the general procedure.

Yield: 62.0 mg (0.192 mmol, 96.1%); Pale yellow solid; mp: 79.9–82.4 °C; TLC R_f = 0.50 ($CHCl_3$/MeOH = 9/1); ^{1}H NMR (400 MHz, $CDCl_3$): δ 8.37 (s, 1H), 8.08 (d, J = 2.8 Hz, 1H), 7.45 (d, J = 8.8 Hz, 1H), 7.36–7.28

(m, 5H), 7.25–7.22 (m, 1H), 6.04 (s, 1H), 4.70 (d, J = 2.4 Hz, 2H), 4.51 (d, J = 5.6 Hz, 2H), 3.77 (s, 3H), 2.56–2.55 (m, 1H); ^{13}C NMR (100 MHz, $CDCl_3$): δ 162.9, 152.2, 151.1, 147.2, 138.0, 136.4, 128.8, 128.3, 127.7, 125.6, 122.5, 107.1, 77.8, 76.6, 56.4, 55.9, 43.8; IR (ZnSe); 2932, 1665, 1634, 1568, 1481, 1221, 1028 cm^{-1}; HRMS (ESI, m/z): [M + H]$^+$ calcd for $C_{19}H_{19}N_2O_3^+$, 323.1390; found 323.1388.

(Z)-N-Benzyl-2-methyl-3-(pyridin-2-yl)acrylamide (**3d**)

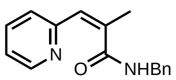

The tittle compound (**3d**) was synthesized from **2g** (34.6 mg, 0.200 mmol, 1 equiv) and benzylamine (32.1 mg, 0.300 mmol, 1.5 equiv) according to the general procedure. The photoirradiation time was 4 min.
Yield: 46.7 mg (0.185 mmol, 92.7%); Pale yellow solid; mp: 58.6–60.0 °C; TLC R_f = 0.59 ($CHCl_3$/MeOH = 9/1); ^{1}H NMR (400 MHz, $CDCl_3$): δ 9.02 (s, 1H), 8.25–8.23 (m, 1H), 7.61–7.57 (m, 1H), 7.32–7.27 (m, 3H), 7.24–7.22 (m, 3H), 7.11–7.08 (m, 1H), 6.65 (s, 1H), 4.48 (d, J = 5.6 Hz, 2H), 2.22 (d, J = 2.0 Hz, 3H); ^{13}C NMR (100 MHz, $CDCl_3$): δ 168.8, 154.3, 148.2, 138.8, 138.3, 137.0, 129.3, 128.7, 128.3, 127.4, 124.9, 122.4, 44.2, 23.7; IR (ZnSe); 3028, 1651, 1632, 1553, 1468, 1433 cm^{-1}; HRMS (ESI, m/z): [M + H]$^+$ calcd for $C_{16}H_{17}N_2O^+$, 253.1335; found 253.1335.

(Z)-N-Benzyl-2-phenyl-3-(pyridin-2-yl)acrylamide (**3e**)

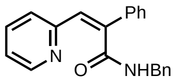

The tittle compound (**3e**) was synthesized from **2h** (47.1 mg, 0.200 mmol, 1 equiv) and benzylamine (32.1 mg, 0.300 mmol, 1.5 equiv) according to the general procedure. The photoirradiation time was 4 min.
Yield: 54.2 mg (0.172 mmol, 86.1%); Pale yellow solid; mp: 123.6–125.6 °C; TLC R_f = 0.41 (hexane/EtOAc = 1/1); ^{1}H NMR (400 MHz, $CDCl_3$): δ 8.53–8.52 (m, 1H), 7.63–7.57 (m, 3H), 7.44–7.35 (m, 4H), 7.29–7.27 (m, 4H), 7.25–7.24 (m, 1H), 7.16–7.13 (m, 1H), 7.03 (s, 1H), 6.27 (s, 1H), 4.58 (d, J = 5.6 Hz, 2H); ^{13}C NMR (100 MHz, $CDCl_3$): δ 169.4, 154.3 149.6, 141.4, 137.8, 137.0, 136.6, 129.0, 128.8, 128.4, 128.2, 127.7, 126.7, 124.0, 122.6, 44.3; IR (ZnSe); 3001, 1663, 1585, 1420, 1258, 999 cm^{-1}; HRMS (ESI, m/z): [M + H]$^+$ calcd for $C_{21}H_{19}N_2O^+$, 315.1492; found 314.1491.

(E)-2-Methoxy-N-(3-phenylpropyl)-3-(pyridin-2-yl)acrylamide (**3f**)

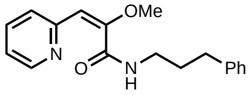

The tittle compound (**3f**) was synthesized from **2a** (37.8 mg, 0.200 mmol, 1 equiv) and 3-phenylpropan-1-amine (40.6 mg, 0.300 mmol, 1.5 equiv) according to the general procedure.
Yield: 58.1mg (0.196 mmol, 98.1%); Colorless oil; TLC R_f = 0.38 (EtOAc/MeOH = 10/1); ^{1}H NMR (400 MHz, DMSO-d_6): δ 8.40–8.37 (m, 2H), 7.65–7.60 (m, 1H), 7.29–7.26 (m, 3H), 7.19–7.15 (m, 3H), 7.10–7.07 (m, 1H), 5.87 (s, 1H), 3.69 (s, 3H), 3.13–3.08 (m, 2H), 2.55 (t, J = 7.6 Hz, 2H), 1.75–1.67 (m, 2H); ^{13}C NMR (100 MHz, DMSO-d_6): δ 164.0, 155.7, 154.3, 148.6, 141.7, 136.0, 128.3, 125.7, 122.1, 120.6, 102.2, 55.5, 38.2, 32.5, 30.2; IR (ZnSe); 2932, 1632, 1163, 1094, 770, 700 cm^{-1}; HRMS (ESI, m/z): [M + H]$^+$ calcd for

$C_{18}H_{21}N_2O_2^+$, 297.1598; found 297.1597.

(E)-2-Methoxy-N-(1-phenylethyl)-3-(pyridin-2-yl)acrylamide (**3g**)

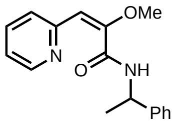

The tittle compound (**3g**) was synthesized from **2a** (37.8 mg, 0.200 mmol, 1 equiv) and 1-phenylethan-1-amine (36.4 mg, 0.300 mmol, 1.5 equiv) according to the general procedure.
Yield: 54.6 mg (0.193 mmol, 96.7%); Pale yellow solid; mp: 122.2–123.6 °C; TLC R_f = 0.48 ($CHCl_3$/MeOH = 9/1); ^{1}H NMR (400 MHz, $CDCl_3$): δ 8.73–8.71 (d, J = 6.0 Hz, 1H), 8.34–8.32 (m, 1H), 7.60–7.55 (m, 1H), 7.37 (d, J = 8.0 Hz, 1H), 7.33–7.30 (m, 4H), 7.28–7.23 (m, 1H), 7.09–7.06 (m, 1H), 6.01 (s, 1H), 5.21–5.14 (m, 1H), 3.78 (s, 3H), 1.52 (d, J = 7.2 Hz, 3H); ^{13}C NMR (100 MHz, $CDCl_3$): δ 162.0, 154.0, 152.8, 148.1, 143.1, 136.5, 128.8, 127.5, 126.6, 125.0, 121.5, 106.6, 56.0, 49.1, 21.8; IR (ZnSe); 2922, 1809, 1735, 1463, 1207 cm^{-1}; HRMS (ESI, m/z): [M + H]$^+$ calcd for $C_{17}H_{19}N_2O_2^+$, 283.1441; found 283.1439.

(E)-N-(tert-Butyl)-2-methoxy-3-(pyridin-2-yl)acrylamide (**3h**)

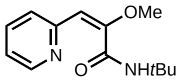

The tittle compound (**3h**) was synthesized from **2a** (37.8 mg, 0.200 mmol, 1 equiv) and 2-methylpropan-2-amine (21.9 mg, 0.299 mmol, 1.5 equiv) according to the general procedure.
Yield: 32.1 mg (0.137 mmol, 68.5%); Pale yellow solid; mp: 108.2–110.8 °C; TLC R_f = 0.46 ($CHCl_3$/MeOH = 9/1); ^{1}H NMR (400 MHz, $CDCl_3$): δ 8.49 (d, J = 4.4 Hz, 1H), 7.64–7.60 (m, 1H), 7.55 (s, 1H), 7.41 (d, J = 8.0 Hz, 1H), 7.13–7.10 (m, 1H), 5.94 (s, 1H), 3.77 (s, 3H), 1.36 (s, 9H); ^{13}C NMR (100 MHz, $CDCl_3$): δ 162.4, 154.5, 154.0, 148.4, 136.4, 124.6, 121.4, 105.3, 55.9, 51.5, 28.6; IR (ZnSe); 2963, 1667, 1626, 1196, 1167 , cm^{-1}; HRMS (ESI, m/z): [M + H]$^+$ calcd for $C_{13}H_{19}N_2O_2^+$, 235.1441; found 235.1443.

(E)-N-((3s,5s,7s)-Adamantan-1-yl)-2-methoxy-3-(pyridin-2-yl)acrylamide (**3i**)

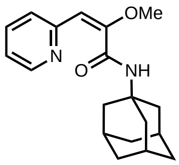

The tittle compound (**3i**) was synthesized from **2a** (37.8 mg, 0.200 mmol, 1 equiv) and (3*s*,5*s*,7*s*)-adamantan-1-amine (45.3 mg, 0.300 mmol, 1.5 equiv) according to the general procedure.
Yield: 56.3 mg (0.180 mmol, 90.2%); Colorless solid; mp: 150.5–152.0 °C; TLC R_f = 0.54 (EtOAc/MeOH = 10/1); ^{1}H NMR (400 MHz, $CDCl_3$): δ 8.51–8.49 (m, 1H), 7.63–7.59 (m, 1H), 7.43 (d, J = 8.0 Hz, 1H), 7.15 (s, 1H), 7.12–7.09 (m, 1H), 5.93 (s, 1H), 3.76 (s, 3H), 2.06 (s, 3H), 2.01 (d, J = 8.0 Hz, 6H), 1.67 (s, 6H); ^{13}C NMR (100 MHz, $CDCl_3$): δ 162.1, 154.6, 153.9, 148.5, 136.3, 124.6, 121.4, 105.5, 55.9, 52.3, 41.4, 36.5, 29.6; IR (ZnSe); 2909, 2882, 1661, 1632, 1416, 1013 cm^{-1}; HRMS (ESI, m/z): [M + H]$^+$ calcd for $C_{19}H_{25}N_2O_2^+$, 313.1911; found 313.1910.

(E)-2-Methoxy-1-morpholino-3-(pyridin-2-yl)prop-2-en-1-one (**3j**)

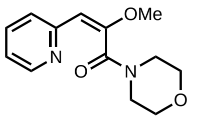

The tittle compound (**3j**) was synthesized from **2a** (37.8 mg, 0.200 mmol, 1 equiv) and morpholine (26.1 mg, 0.300 mmol, 1.5 equiv) according to the general procedure.

Yield: 45.5 mg (0.183 mmol, 91.7%); Pale yellow solid; mp: 100.0–102.5 °C; TLC R_f = 0.54 (EtOAc/MeOH = 9/1); ^{1}H NMR (400 MHz, $CDCl_3$): δ 8.46 (d, J = 4.0 Hz, 1H), 7.58–7.54 (m, 1H), 7.15 (d, J = 8.0 Hz, 1H), 7.06–7.03 (m, 1H), 5.83 (s, 1H), 3.80 (s, 3H), 3.71 (s, 4H), 3.49 (t, J = 4.8 Hz, 2H), 3.36 (t, J = 4.8 Hz, 2H); ^{13}C NMR (100 MHz, $CDCl_3$): δ 165.5, 154.3, 153.9, 149.5, 136.5, 122.3, 121.1, 102.2, 66.5, 66.4, 56.1, 46.7, 41.9; IR (ZnSe); 2860, 1557, 1425, 1152, 1113 cm^{-1}; HRMS (ESI, m/z): [M + H]$^+$ calcd for $C_{13}H_{17}N_2O_3^+$, 249.1234; found 249.1237.

(E)-1-(4-(Hydroxymethyl)piperidin-1-yl)-2-methoxy-3-(pyridin-2-yl)prop-2-en-1-one (**3k**)

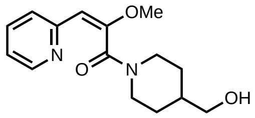

The tittle compound (**3k**) was synthesized from **2a** (37.8 mg, 0.200 mmol, 1 equiv) and piperidin-4-ylmethanol (34.6 mg, 0.300 mmol, 1.5 equiv) according to the general procedure.

Yield: 44.8 mg (0.162 mmol, 81.2%); Colorless oil; TLC R_f = 0.43 ($CHCl_3$/MeOH = 5/1); ^{1}H NMR (400 MHz, $CDCl_3$): δ 8.46–8.44 (m, 1H), 7.56–7.52 (m, 1H), 7.20 (d, J = 8.0 Hz, 1H), 7.04–7.00 (m, 1H), 5.84 (s, 1H), 4.72–4.66 (m, 1H), 3.83–3.79 (m, 4H), 3.47–3.38 (m, 2H), 2.94–2.87 (m, 1H), 2.70–2.63 (m, 1H), 1.80–1.56 (m, 4H), 1.26–1.15 (m, 1H), 0.93– 0.82 (m, 1H); ^{13}C NMR (100 MHz, $CDCl_3$): δ 164.9, 155.0, 154.2, 149.3, 136.4, 122.2, 120.9, 102.0, 67.5, 56.0, 46.6, 41.3, 38.9, 28.6, 27.9; IR (ZnSe); 2918, 2857, 1620, 1587, 1471, 1263 cm^{-1}; HRMS (ESI, m/z): [M + H]$^+$ calcd for $C_{15}H_{21}N_2O_3^+$, 277.1547; found 277.1547.

(E)-N-(6-Hydroxyhexyl)-2-methoxy-3-(pyridin-2-yl)acrylamide (**3l**)

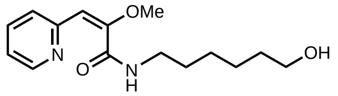

The tittle compound (**3l**) was synthesized from **2a** (37.8 mg, 0.200 mmol, 1 equiv) and 6-aminohexan-1-ol (35.2 mg, 0.300 mmol, 1.5 equiv) according to the general procedure.

Yield: 48.6 mg (0.175 mmol, 87.4%); Colorless solid; mp: 64.7–66.5°C; TLC R_f = 0.56 (hexane/EtOAc = 85/15); ^{1}H NMR (400 MHz, $CDCl_3$): δ 8.50–8.48 (m, 1H), 7.94 (s, 1H), 7.65–7.60 (m, 1H), 7.43 (d, J = 7.6 Hz, 1H), 7.13–7.10 (m, 1H), 6.02 (s, 1H), 3.78 (s, 3H), 3.62 (t, J = 6,4 Hz, 2H), 3.33–3.28 (m, 2H), 1.67 (s, 1H), 1.58–1.50 (m, 4H), 1.41–1.28 (m, 4H); ^{13}C NMR (100 MHz, $CDCl_3$): δ 163.0, 154.3, 152.6, 148.4, 136.4, 125.0, 121.5, 106.6, 62.9, 55.9, 39.5, 32.7, 29.3, 26.8, 25.5; IR (ZnSe); 2930, 2855, 1624, 1566, 1431, 1167 cm^{-1}; HRMS (ESI, m/z): [M + H]$^+$ calcd for $C_{15}H_{23}N_2O_3^+$, 279.1703; found .279.1703.

tert-Butyl (E)-(2-methoxy-3-(pyridin-2-yl)acryloyl)-L-tyrosinate (**3m**)

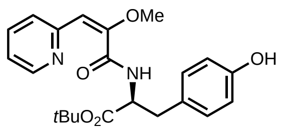

The tittle compound (**3m**) was synthesized from **2a** (37.8 mg, 0.200 mmol, 1 equiv) and *tert*-butyl L-tyrosinate (71.2 mg, 0.300 mmol, 1.5 equiv) according to the general procedure.

Yield: 77.1 mg (0.193 mmol, 96.9%, 99.6% ee); Pale yellow solid; mp: 68.3–74.4 °C; $[\alpha]_D^{23}$ +60.3 (c 0.500, $CHCl_3$); TLC R_f = 0.40 ($CHCl_3$/MeOH = 15/1); ^{1}H NMR (400 MHz, $CDCl_3$): δ 8.67 (d, *J* = 7.6 Hz, 1H), 8.43–8.41 (m, 1H), 7.64–7.60 (m, 1H), 7.41 (d, *J* = 8.4 Hz, 1H), 7.13–7.10 (m, 1H), 6.96–6.94 (AA′ BB′ , 2H), 6.72–6.69 (AA′ BB′ , 2H), 6.04 (s, 1H), 4.79–4.74 (m, 1H), 3.68 (s, 3H), 3.03 (d, *J* = 6.0 Hz, 2H), 1.39 (s, 9H); ^{13}C NMR (100 MHz, $CDCl_3$): δ 170.7, 162.2, 155.5, 153.8, 151.6, 148.0, 136.6, 130.8, 127.7, 125.2, 121.7, 115.5, 107.4, 82.4, 55.9, 54.3, 37.3, 28.1; IR (ZnSe); 2978, 1728, 1631, 1612, 1593, 1514, 1149 cm^{-1}; HRMS (ESI, m/z): [M + H]$^+$ calcd for $C_{22}H_{27}N_2O_5^+$, 399.1914; found 399.1912.

tert-Butyl (E)-(2-methoxy-3-(pyridin-2-yl)acryloyl)tyrosinate (*rac*-**3m**)

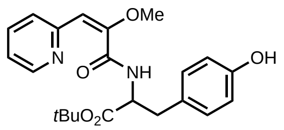

The tittle compound (**3m**) was synthesized from **2a** (37.8 mg, 0.200 mmol, 1 equiv) and *tert*-butyl tyrosinate (71.2 mg, 0.300 mmol, 1.5 equiv) according to the general procedure.

Yield: 77.7 mg (0.195 mmol, 97.6%); Pale yellow solid; mp: 68.3–74.4 °C; TLC R_f = 0.40 ($CHCl_3$/MeOH = 15/1); ^{1}H NMR (400 MHz, $CDCl_3$): δ 8.79 (d, *J* = 7.2 Hz, 1H), 8.41–8.40 (m, 1H), 7.64–7.59 (m, 1H), 7.39 (d, *J* = 7.6 Hz, 1H), 7.12–7.09 (m, 1H), 6.98–6.96 (AA′BB′, 2H), 6.69–6.67 (AA′BB′, 2H), 6.04 (s, 1H), 4.81–4.76 (m, 1H), 3.72 (s, 3H), 3.04 (d, *J* = 6.4 Hz, 2H), 1.39 (s, 9H); ^{13}C NMR (100 MHz, $CDCl_3$): δ 170.7, 162.2, 155.2, 153.8, 151.6, 148.1, 136.5, 130.9, 128.1, 125.1, 121.7, 115.4, 107.5, 82.4, 56.0, 54.3, 37.3, 28.2; IR (ZnSe); 2978, 1728, 1634, 1612, 1593, 1514, 1227 cm^{-1}; HRMS (ESI, m/z): [M + H]$^+$ calcd for $C_{22}H_{27}N_2O_5^+$, 399.1914; found 399.1913.

(*E*)-*N*-(2,2-Dimethoxyethyl)-2-methoxy-3-(pyridin-2-yl)acrylamide (**3n**)

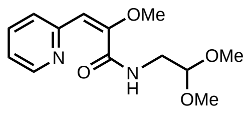

The tittle compound (**3n**) was synthesized from **2a** (37.8 mg, 0.200 mmol, 1 equiv) and 2,2-dimethoxyethan-1-amine (31.5 mg, 0.300 mmol, 1.5 equiv) according to the general procedure.

Yield: 51.1 mg (0.192 mmol, 96.1%); Pale yellow solid; mp: 45.9–47.4 °C; TLC R_f = 0.34 ($CHCl_3$/MeOH = 9/1); ^{1}H NMR (400 MHz, $CDCl_3$): δ 8.53–8.51 (m, 1H), 8.30 (s, 1H), 7.65–7.61 (m, 1H), 7.42 (d, *J* = 7.6 Hz, 1H), 7.14–7.11 (m, 1H), 6.05 (s, 1H), 4.41 (t, *J* = 5.6 Hz, 1H), 3.79 (s, 3H), 3.48 (t, *J* = 5.6 Hz, 2H), 3.378(s, 6H); ^{13}C NMR (100 MHz, $CDCl_3$): δ 163.1, 154.1, 152.1, 148.4, 136.4, 124.9, 121.5, 107.1, 102.6, 56.0, 54.4, 41.1; IR (ZnSe); 2938, 2833, 1667, 1632, 1518, 1192 cm^{-1}; HRMS (ESI, m/z): [M + H]$^+$ calcd for $C_{13}H_{19}N_2O_4^+$, 267.1339; found 267.1339.

(*E*)-*N*-(2-(Cyclohex-1-en-1-yl)ethyl)-2-methoxy-3-(pyridin-2-yl)acrylamide (**3o**)

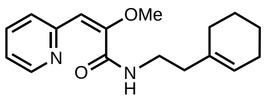

The tittle compound (**3o**) was synthesized from **2a** (37.8 mg, 0.200 mmol, 1 equiv) and 2-(cyclohex-1-en-1-yl)ethan-1-amine (37.6 mg, 0.300 mmol, 1.5 equiv) according to the general procedure.
Yield: 56.1 mg (0.196 mmol, 98.1%); Pale yellow oil; TLC R_f = 0.50 ($CHCl_3$/MeOH = 9/1); ^{1}H NMR (400 MHz, $CDCl_3$): δ 8.51–8.50 (m, 1H), 7.64–7.59 (m, 1H), 7.53–7.43 (m, 2H), 7.13–7.09 (m, 1H), 6.02 (s, 1H), 5.38–5.37 (m, 1H), 3.78 (s, 3H), 3.40–3.35 (m, 2H), 2.13 (t, J = 7.2 Hz, 2H), 1.97–1.87 (m, 4H), 1.64–1.48 (m, 4H); ^{13}C NMR (100 MHz, $CDCl_3$): δ 163.0, 154.4, 152.7, 148.6, 136.3, 134.7, 124.7, 123.5, 121.5, 106.6, 55.9, 37.5, 37.3, 28.1, 25.4, 23.0, 22.5; IR (ZnSe); 2924, 1659, 1632, 1587, 1433, 1248, 1163 cm^{-1}; HRMS (ESI, m/z): [M + H]$^+$ calcd for $C_{17}H_{23}N_2O_2^+$, 287.1754; found 287.1751.

(*E*)-2-Methoxy-*N*-(prop-2-yn-1-yl)-3-(pyridin-2-yl)acrylamide (**3p**)

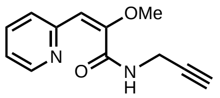

The tittle compound (**3p**) was synthesized from **2a** (37.8 mg, 0.200 mmol, 1 equiv) and propargylamine (16.5, 0.300 mmol, 1.5 equiv) according to the general procedure.
Yield: 41.6 mg (0.192 mmol, 96.3%); Pale yellow oil; TLC R_f = 0.38 ($CHCl_3$/MeOH = 9/1); ^{1}H NMR (400 MHz, $CDCl_3$): δ 9.46 (s, 1H), 8.52–8.51 (m, 1H), 7.68–7.64 (m, 1H), 7.40 (d, J = 8.4 Hz, 1H), 7.16–7.13 (m, 1H), 6.06 (s, 1H), 4.13–4.11 (m, 2H), 3.80 (s, 3H), 2.24 (t, J = 2.8 Hz, 1H); ^{13}C NMR (100 MHz, $CDCl_3$): δ 162.4, 153.6, 151.8, 148.0, 136.8, 125.3, 121.7, 107.2, 79.6, 71.7, 56.0, 29.4; IR (ZnSe); 2934, 2835, 1661, 1632, 1587, 1163 cm^{-1}; HRMS (ESI, m/z): [M + H]$^+$ calcd for $C_{12}H_{13}N_2O_2^+$ 217.0972; found 217.0972.

(*E*)-*N*-(3-Azidopropyl)-2-methoxy-3-(pyridin-2-yl)acrylamide (**3q**)

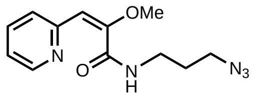

The tittle compound (**3q**) was synthesized from **2a** (37.8 mg, 0.200 mmol, 1 equiv) and 3-azidopropan-1-amine (30.0, 0.300 mmol, 1.5 equiv) according to the general procedure.
Yield: 51.3 mg (0.196 mmol, 98.3%); Colorless solid; mp: 43.4–45.6 °C; TLC R_f = 0.36 ($CHCl_3$/MeOH = 9/1); ^{1}H NMR (400 MHz, $CDCl_3$): δ 8.52–8.50 (m, 2H), 7.67–7.63 (m, 1H), 7.40 (d, J = 8.0 Hz, 1H), 7.16–7.13 (m, 1H), 6.03 (s, 1H), 3.80 (s, 3H), 3.42 (q, J = 6.0 Hz, 2H), 3.34 (t, J = 6.8 Hz, 2H), 1.87–1.81 (m, 2H); ^{13}C NMR (100 MHz, $CDCl_3$): δ 163.2, 154.1, 152.4, 148.3, 136.6, 125.1, 121.7, 106.7, 56.0, 49.4, 37.0, 28.6; IR (ZnSe); 2940, 2095, 1661, 1634,1566, 1476, 1252 cm^{-1}; HRMS (ESI, m/z): [M + H]$^+$ calcd for $C_{12}H_{16}N_5O_2^+$, 262.1299; found 262.1299.

Methyl (E)-3-(4-azidophenyl)-2-(2-methoxy-3-(pyridin-2-yl)acrylamido)propanoate (**3r**)

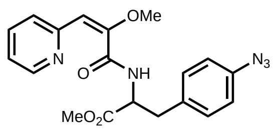

The tittle compound (**3r**) was synthesized from **2a** (37.8 mg, 0.200 mmol, 1 equiv) and methyl 2-amino-3-(4-azidophenyl)propanoate (66.1, 0.300 mmol, 1.5 equiv) according to the general procedure.

Yield: 51.9 mg (0.136 mmol, 68.1%); Colorless solid; mp: 113.5–114.9°C; TLC R_f = 0.41 ($CHCl_3$/MeOH = 20/1); ^{1}H NMR (400 MHz, $CDCl_3$): δ 9.85–9.84 (m, 1H), 8.29 (d, J = 4.4 Hz, 1H), 7.65–7.61 (m, 1H), 7.35–7.33 (m, 1H), 7.11–7.09 (m, 1H), 7.05–7.03 (m, 2H), 6.84–6.82 (m, 2H), 6.05 (s, 1H), 4.99–4.98 (m, 1H), 3.79 (s, 3H), 3.71 (s, 3H), 3.17–3.13(m, 2H); ^{13}C NMR (100 MHz, $CDCl_3$): δ 171.8, 162.3, 153.5, 151.9, 148.1, 138.9, 136.6, 133.2, 130.9, 125.0, 121.6, 119.1, 107.6, 56.1, 54.0, 52.4, 37.5; IR (ZnSe); 2112, 1738, 1655, 1641, 1537, 1161 cm^{-1}; HRMS (ESI, m/z): [M + H]$^+$ calcd for $C_{19}H_{20}N_5O_4^+$, 382.1510; found 382.1508.

(E)-2-Methoxy-3-(pyridin-2-yl)-N-(pyridin-3-ylmethyl)acrylamide (**3s**)

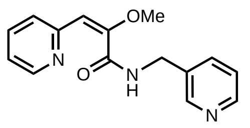

The tittle compound (**3s**) was synthesized from **2a** (37.8 mg, 0.200 mmol, 1 equiv) and pyridin-3-ylmethanamine (32.4 mg, 0.300 mmol, 1.5 equiv) according to the general procedure.

Yield: 51.3 mg (0.190 mmol, 95.4%); Pale yellow oil; TLC R_f = 0.59 ($CHCl_3$/MeOH = 4/1); ^{1}H NMR (400 MHz, $CDCl_3$): δ 9.367(s, 1H), 8.57 (d, J = 2.0 Hz, 1H), 8.54–8.53 (dd, J = 17.0, 1.6 Hz, 1H), 8.31–8.30 (m, 1H), 7.69–7.60 (m, 2H), 7.37 (d, J = 8.4 Hz, 1H), 7.28–7.25 (m, 1H), 7.12–7.09 (m, 1H), 6.05 (s, 1H), 4.55 (d, J = 6.0 Hz, 2H), 3.80 (s, 3H); ^{13}C NMR (100 MHz, $CDCl_3$): δ 163.0, 153.7, 152.3, 149.6, 149.0, 148.0, 136.8, 136.1, 133.9, 125.2, 123.7, 121.7, 106.9, 56.0, 41.3; IR (ZnSe); 2935, 1660, 1587, 1557, 1427, 1277, 1146 cm^{-1}; HRMS (ESI, m/z): [M + H]$^+$ calcd for $C_{15}H_{16}N_3O_2^+$, 270.1237; found 270.1236.

(E)-2-Methoxy-3-(pyridin-2-yl)-N-(thiophen-2-ylmethyl)acrylamide (**3t**)

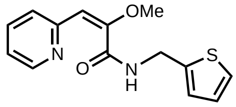

The tittle compound (**3t**) was synthesized from **2a** (37.8 mg, 0.200 mmol, 1 equiv) and thiophen-2-ylmethanamine (34.0 mg, 0.300 mmol, 1.5 equiv) according to the general procedure.

Yield: 56.1 mg (0.195 mmol, 97.4%); Pale yellow solid; mp: 81.9–90.2 °C; TLC R_f = 0.54 ($CHCl_3$/MeOH = 4/1); ^{1}H NMR (400 MHz, $CDCl_3$): δ 9.08 (s, 1H), 8.35–8.33 (m, 1H), 7.63–7.59 (m, 1H), 7.39 (d, J = 8.0 Hz, 1H), 7.23 (dd, J = 4.8, 1.6 Hz, 1H), 7.11–7.08 (m, 1H), 7.00–6.99 (m, 1H), 6.97–6.95 (m, 1H), 6.04 (s, 1H), 4.69 (d, J = 5.6 Hz, 2H), 3.79 (s, 3H); ^{13}C NMR (100 MHz, $CDCl_3$): δ 162.5, 153.8, 152.2, 148.1, 140.4, 136.6, 127.1, 126.6, 125.4, 125.1, 121.6, 107.1, 56.0, 38.5; IR (ZnSe); 2932, 2905, 1663, 1626, 1566, 1377, 1234 cm^{-1}; HRMS (ESI, m/z): [M + H]$^+$ calcd for $C_{14}H_{15}NO_2S^+$, 275.0849; found 275.0847.

(*E*)-*N*-(2-(1*H*-Indol-3-yl)ethyl)-2-methoxy-3-(pyridin-2-yl)acrylamide (**3u**)

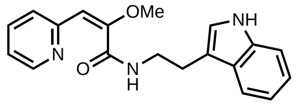

The tittle compound (**3u**) was synthesized from **2a** (37.8 mg, 0.200 mmol, 1 equiv) and 2-(1*H*-indol-3-yl)ethan-1-amine (48.1 mg, 0.300 mmol, 1.5 equiv) according to the general procedure.
Yield: 46.6 mg (0.145 mmol, 72.6%); Colorless solid; mp: 40.0–44.4 °C; TLC R_f = 0.59 ($CHCl_3$/MeOH = 4/1); ^{1}H NMR (400 MHz, $CDCl_3$): δ 8.28 (d, J = 4.4 Hz, 1H), 8.06 (s, 1H), 7.89 (s, 1H), 7.60–7.54 (m, 2H), 7.41–7.35 (m, 2H), 7.22–7.18 (m, 1H), 7.13–7.09 (m, 1H), 7.05–7.01 (m, 1H), 6.95 (d, J = 4.4 Hz, 1H), 5.99 (s, 1H), 3.74 (s, 3H), 3.67 (q, J = 6.8 Hz, 2H), 2.99 (t, J = 6.8 Hz, 2H); ^{13}C NMR (100 MHz, $CDCl_3$): δ 163.1, 154.2, 152.6, 148.4, 136.5, 136.3, 127.5, 124.8, 122.3, 122.2, 121.4, 119.6, 119.0, 113.2, 111.4, 106.6, 55.9, 39.7, 25.2; IR (ZnSe); 2928, 1632, 1587, 1518, 1229 cm^{-1}; HRMS (ESI, m/z): [M + H]$^+$ calcd for $C_{19}H_{20}N_3O_2^+$, 322.1550; found 322.1549.

(E)-N-(2-Fluoroethyl)-2-methoxy-3-(pyridin-2-yl)acrylamide (**3v**)

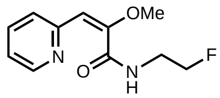

To an MeCN solution (39 mL) of **2a** (37.8 mg, 0.200 mmol, 1 equiv) in a 100 mL round-bottom flask were added an aqueous (1 mL) solution of 2-fluoroethylamine hydrochloride (29.9 mg, 0.300 mmol, 1.5 equiv) and Na_2CO_3 (31.8 mg, 0.300 mmol, 1.5 equiv), an MeCN solution of methylene blue (1.0 mL, 2.0 mM, 2.0 μmol, 1.0 mol %) under air. The solution was vigorously stirred by a magnetic stirrer at room temperature under air and photoirradiated for 3 min. After the photoirradiation, the solvent was evaporated. The residue was extracted from EtOAc (15 mL) and water (15 mL) for three times. The combined organic layer was dried with Na_2SO_4 and evaporated. The residue was purified by silica-gel column chromatography ($CHCl_3$/MeOH = 1/0 to 95/5).
Yield: 41.9 mg (0.187 mmol, 93.4%); Colorless solid; mp: 79.7–80.3 °C; TLC R_f = 0.44 ($CHCl_3$/MeOH = 19/1); ^{1}H NMR (400 MHz, $CDCl_3$): δ 9.14 (s, 1H), 8.52–8.50 (m, 1H), 7.67–7.62 (m, 1H), 7.39 (d, J = 7.6 Hz, 1H), 7.15–7.12 (m, 1H), 6.05 (s, 1H), 4.60 (t, J = 4.8 Hz, 1H), 4.48 (t, J = 4.8 Hz, 1H), 3.80 (s, 3H), 3.69 (q, J = 4.4 Hz, 1H), 3.62 (q, J = 4.4 Hz, 1H); ^{13}C NMR (100 MHz, $CDCl_3$): δ 163.1, 153.8, 152.1, 148.3, 136.6, 125.1, 121.7, 107.0, 82.8 (d, $^1J_{C\text{-}F}$ = 167.7 Hz), 56.0, 40.1 (d, $^2J_{C\text{-}F}$ = 20.2 Hz); IR (ZnSe); 2926, 1661, 1628, 1566, 1435, 1260 cm^{-1}; ^{19}F NMR (373 MHz, $CDCl_3$): δ –223.1; HRMS (ESI, m/z): [M + H]$^+$ calcd for $C_{11}H_{14}FN_2O_2^+$, 225.1034; found 225.1033.

(E)-2-Methoxy-3-(pyridin-2-yl)-N-(4-(3-(trifluoromethyl)-3H-diazirin-3-yl)benzyl)acrylamide (**3w**)

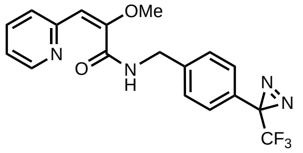

An MeCN (1 mL) solution of 4-[3-(trifluoromethyl)-3*H*-diazirin-3-yl]benzylamine hydrochloride (75.5 mg, 0.300 mmol, 1.5 equiv) and an aqueous (1 mL) solution of Na_2CO_3 (31.8 mg, 0.300 mmol, 1.5 equiv) were combined in a 4 mL vial. The resulting solution was added to a stirring MeCN (39 mL) solution of **2a** (37.8

mg, 0.200 mmol, 1 equiv) in a 100 mL round-bottom flask under air. An MeCN (1 mL) solution of methylene blue (2.0 mM, 2.0 μmol, 1.0 mol %) was added into the flask under air. The solution was vigorously stirred by a magnetic stirrer at room temperature under air and photoirradiated for 3 min. After the photoirradiation, the solvent was evaporated. The residue was extracted from EtOAc (15 mL) and water (15 mL) for three times. The combined organic layer was dried with Na_2SO_4 and evaporated. The residue was purified by silica-gel column chromatography ($CHCl_3$/MeOH = 1/0 to 95/5).

Yield: 73.8 mg (0.196 mmol, 98.2%); Colorless solid; mp: 94.8–95.7 °C; TLC R_f = 0.45 ($CHCl_3$/MeOH = 10/1); ^{1}H NMR (400 MHz, $CDCl_3$): δ 9.11 (s, 1H), 8.29 (d, J = 4.4 Hz, 1H), 7.63–7.59 (m, 1H), 7.39–7.32 (m, 3H), 7.15 (d, J = 8.4 Hz, 2H), 7.11–7.08 (m, 1H), 6.05 (s, 1H), 4.53 (d, J = 5.2 Hz, 2H), 3.79 (s, 3H); ^{13}C NMR (100 MHz, $CDCl_3$): δ 162.9, 153.8, 152.2, 148.0, 140.1, 136.7, 128.6, 128.3, 126.9, 125.2, 122.3 (q, $^1J_{C\text{-}F}$=275.9 Hz), 121.7, 106.9, 56.0, 43.2, 28.5 (q, $^2J_{C\text{-}F}$ = 40.2 Hz), carbon of OCD_3 group was not observed; ^{19}F NMR (373 MHz, $CDCl_3$): δ −65.2; IR (ZnSe); 3065, 1641, 1566, 1518, 1342, 1145 cm^{-1}; HRMS (ESI, m/z): [M + H]$^+$ calcd for $C_{18}H_{16}F_3N_4O_2^+$, 377.1220; found 377.1215.

(7-(diethylamino)-2-oxo-2H-chromen-4-yl)methyl (E)-4-((6-(2-methoxy-3-oxo-3-((thiophen-2-ylmethyl)-amino)prop-1-en-1-yl)pyridin-3-yl)oxy)butanoate (**3x**)

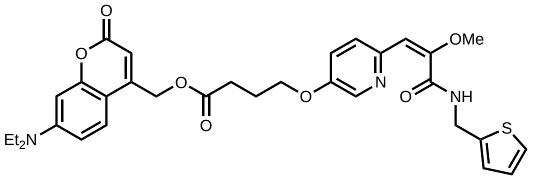

The tittle compound (**3x**) was synthesized from **2i** (52.1 mg, 0.100 mmol, 1 equiv) and thiophen-2-ylmethanamine (17.0 mg, 0.150 mmol, 1.5 equiv) according to the general procedure (0.100 mmol scale).

Yield: 50.2 mg (0.0829 mmol, 82.8%); Yellow solid; mp: 45.0–48.4 °C; TLC R_f = 0.50 ($CHCl_3$/MeOH = 10/1); ^{1}H NMR (400 MHz, $CDCl_3$): δ 8.83 (s, 1H), 8.03 (d, J = 2.8 Hz, 1H), 7.39 (d, J = 8.4 Hz, 1H), 7.29 (d, J = 9.2 Hz, 1H), 7.24–7.23 (m, 1H), 7.14–7.11 (m, 1H), 7.00–6.99 (m, 1H), 6.97–6.95 (m, 1H), 6.59–6.56 (m, 1H), 6.51 (d, J = 2.0 Hz, 1H), 6.11 (s, 1H), 6.03 (s, 1H), 5.25 (s, 2H), 4.68 (d, J = 5.6 Hz, 2H), 4.06 (t, J = 6.4 Hz, 2H), 3.76 (s, 3H), 3.41 (q, J = 7.6 Hz, 4H), 2.68 (t, J = 7.6 Hz, 2H), 2.23–2.16 (m, 2H), 1.21 (t, J = 7.6 Hz, 6H); ^{13}C NMR (100 MHz, $CDCl_3$): δ 172.5, 162.6, 162.1, 156.4, 153.4, 150.8, 150.7, 149.5, 146.3, 140.5, 135.8, 127.0, 126.6, 125.7, 125.4, 124.5, 121.8, 108.8, 107.3, 106.6, 106.1, 98.0, 67.1, 61.6, 55.9, 44.9, 38.4, 30.6, 24.6, 12.6; IR (ZnSe); 2968, 1913, 1599, 1526, 1418, 1229, 1159, 826 cm^{-1}; HRMS (ESI, m/z): [M + H]$^+$ calcd for $C_{32}H_{36}N_3O_7S^+$, 606.2268; found 606.2264.

Chiral HPLC charts

Chiral HPLC charts of **3m** and *rac*-**3m** (column, IA; mobile phase, hexane/*i*PrOH = 4/1, 1 mL/min)

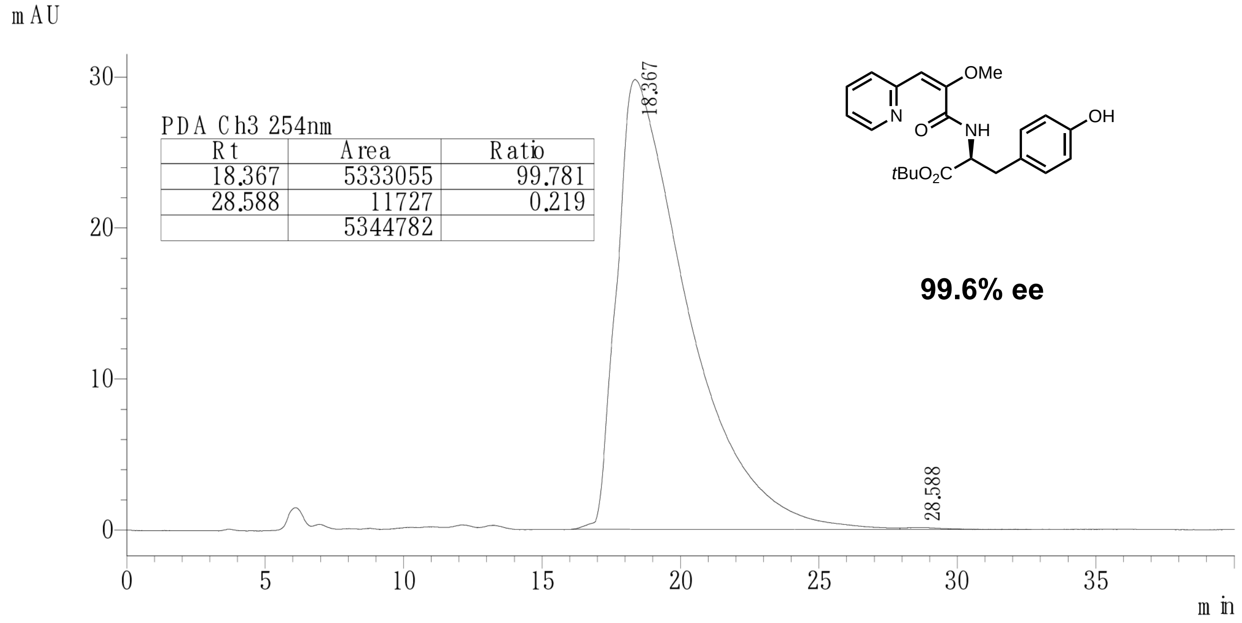

Rt	Area	Ratio
18.367	5333055	99.781
28.588	11727	0.219
	5344782	

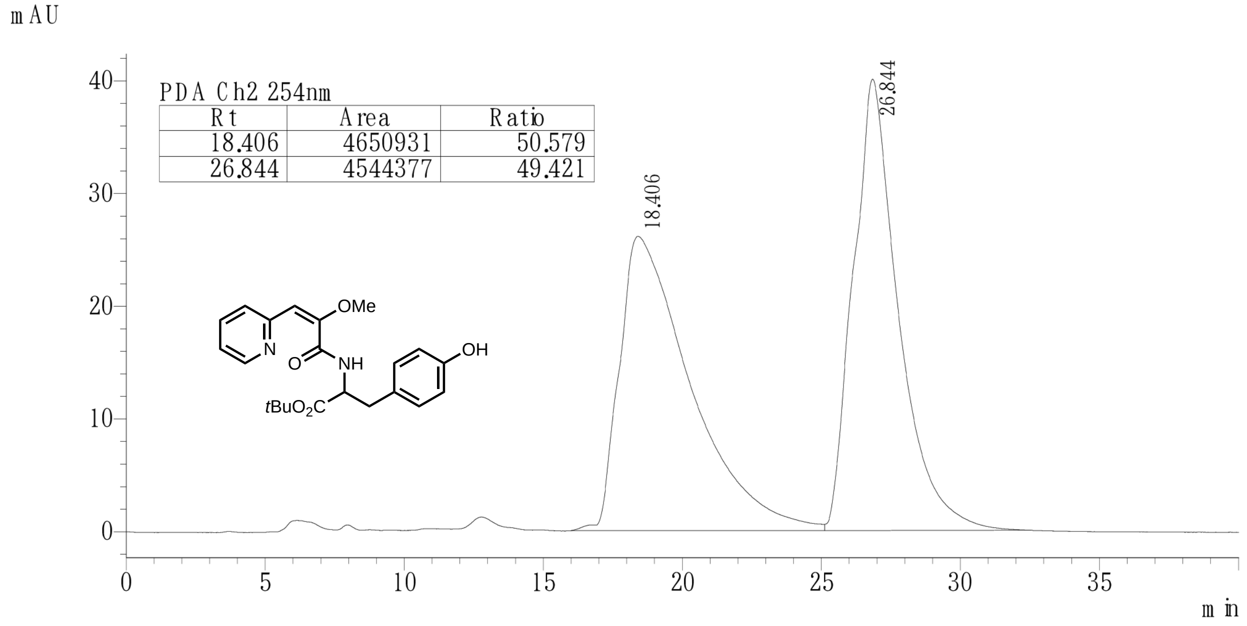

Rt	Area	Ratio
18.406	4650931	50.579
26.844	4544377	49.421

ORTEP diagram and crystallographic data

Single crystal X-ray structural analysis was performed on Rigaku AFC-11 equipped with Hypix-6000HE X-ray detector. Single crystals suitable for X-ray diffraction measurement were prepared by recrystallization of **3i** from hexane.

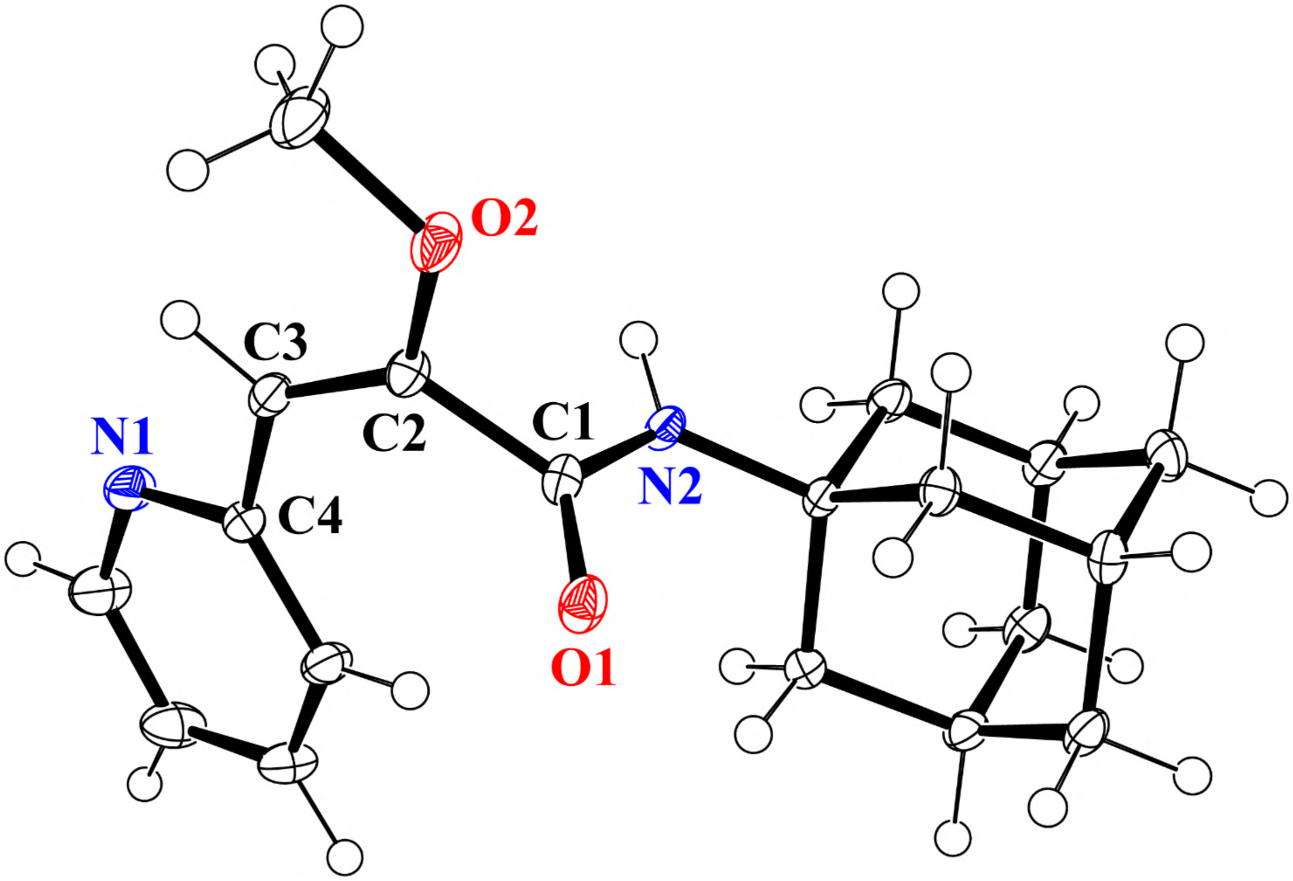

Fig. S6 ORTEP drawing of 3i with thermal ellipsoids at the 50% probability level. CCDC number 2149818.

Table S2. Crystallographic data of 3i.

Empirical formula	$C_{19}H_{24}N_2O_2$
Formula weight	312.40
Color, description	Colorless, block
Temperature, K	90.00(10)
Crystal system	Monoclinic
Space group	$P2_1/c$
a, Å	12.47992(17)
b, Å	10.84447(14)
c, Å	12.67358(19)
β , º	100.4319(14)
V, Å^3	1686.87(4)
Z	4
Calculated density, Mg m^{-3}	1.230
Limiting indices	$-17 \leq h \leq 17, -15 \leq k \leq 15, -17 \leq l \leq 17$
Linear absorption coefficient, mm^{-1}	0.080
F(000)	672
Crystal size, mm	0.14 × 0.11 × 0.10
Goodness-of-fit on F^2	1.053
R_1, wR_2 for 4381 reflections with $I > 2\sigma(I)$	0.0361, 0.1036
$\Delta\rho_{min}$, $\Delta\rho_{max}$, e Å^{-3}	−0.204, 0.413

References

S1. Watanabe, K. et al. Indolizines enabling rapid uncaging of alcohols and carboxylic acids by red light-induced photooxidation. *Org. Lett.* **22**, 5434-5438 (2020).
S2. Watanabe, K., Terao, N., Niwa, T., Hosoya, T. Direct 3-acylation of indolizines by carboxylic acids for the practical synthesis of red light-releasable caged carboxylic acids. *J. Org. Chem.* **86**, 11822-11834 (2021).
S3. Hynd, G. et al. Indolizine derivatives as CRTH2 receptor modulators, their preparation, pharmaceutical compositions. *PCT Int. Appl.* WO2007031747 (2007).
S4. Amaral, M. F. Z. J., Deliberto, L. A., de Souza, C. R. & Naal, R. M. Z. G. Synthesis, photophysical, and electrochemical properties of 2,5-diaryl-indolizines. *Tetrahedron* **70**, 3249-3258 (2014).
