# Supplementary material for: Red light-induced conjugation of amines through amide bond formation triggered via photooxidation of 3-acylindolizines
Source: Commun Chem. 2022 Aug 5;5:91. doi: 10.1038/s42004-022-00712-5 (PMC9814406; doi:10.1038/s42004-022-00712-5)
Supplement: Supplementary file 2 — Description of Additional Supplementary Files [file 42004_2022_712_MOESM2_ESM.docx]

Description of Additional Supplementary Files

**File name:** Supplementary Data 1

**Description:** NMR charts of all synthesized compounds

**File name:** Supplementary Data 2

**Description:** Cif file of 3i
