# Supplementary material for: Red light-induced conjugation of amines through amide bond formation triggered via photooxidation of 3-acylindolizines
Source: Commun Chem. 2022 Aug 5;5:91. doi: 10.1038/s42004-022-00712-5 (PMC9814406; doi:10.1038/s42004-022-00712-5)
Supplement: Supplementary file 3 — Supplementary Data 1 [file 42004_2022_712_MOESM3_ESM.pdf]

NMR charts

^{1}H NMR (400 MHz) and ^{13}C NMR (100 MHz) spectra of **2a** ($CDCl_3$)

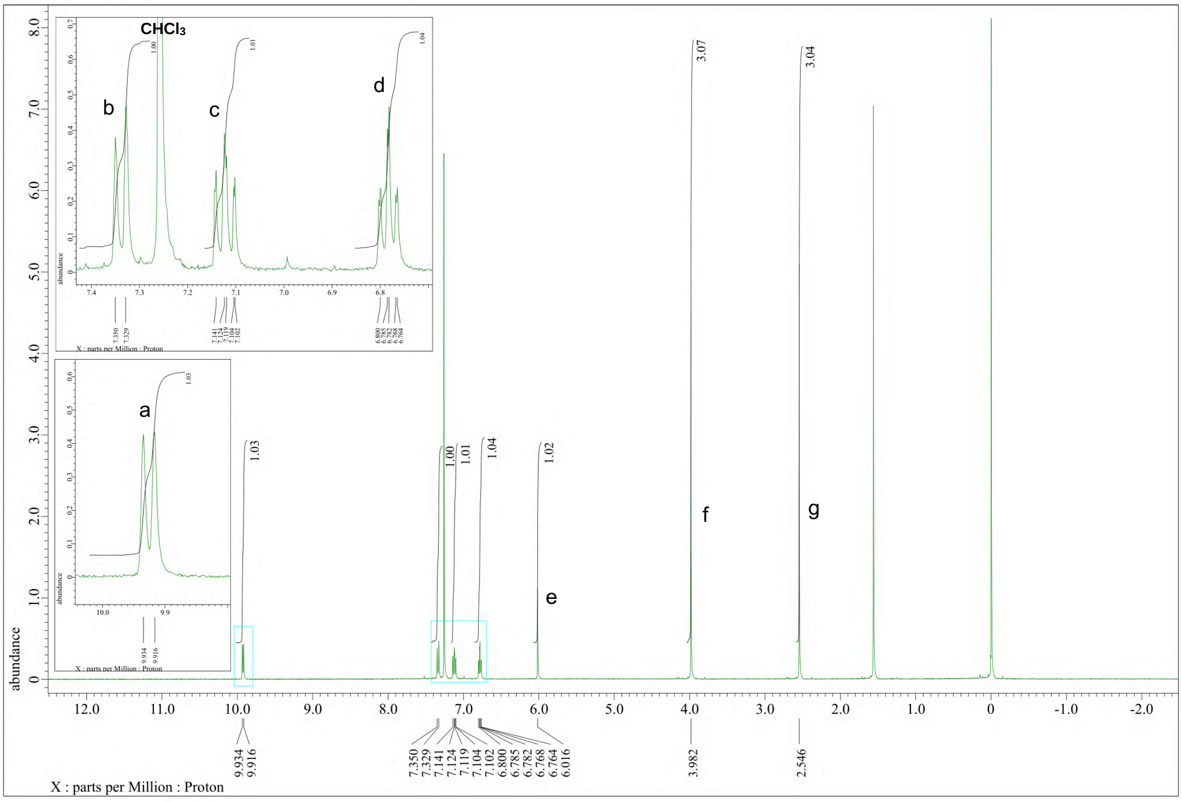

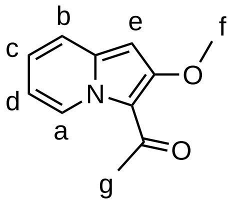

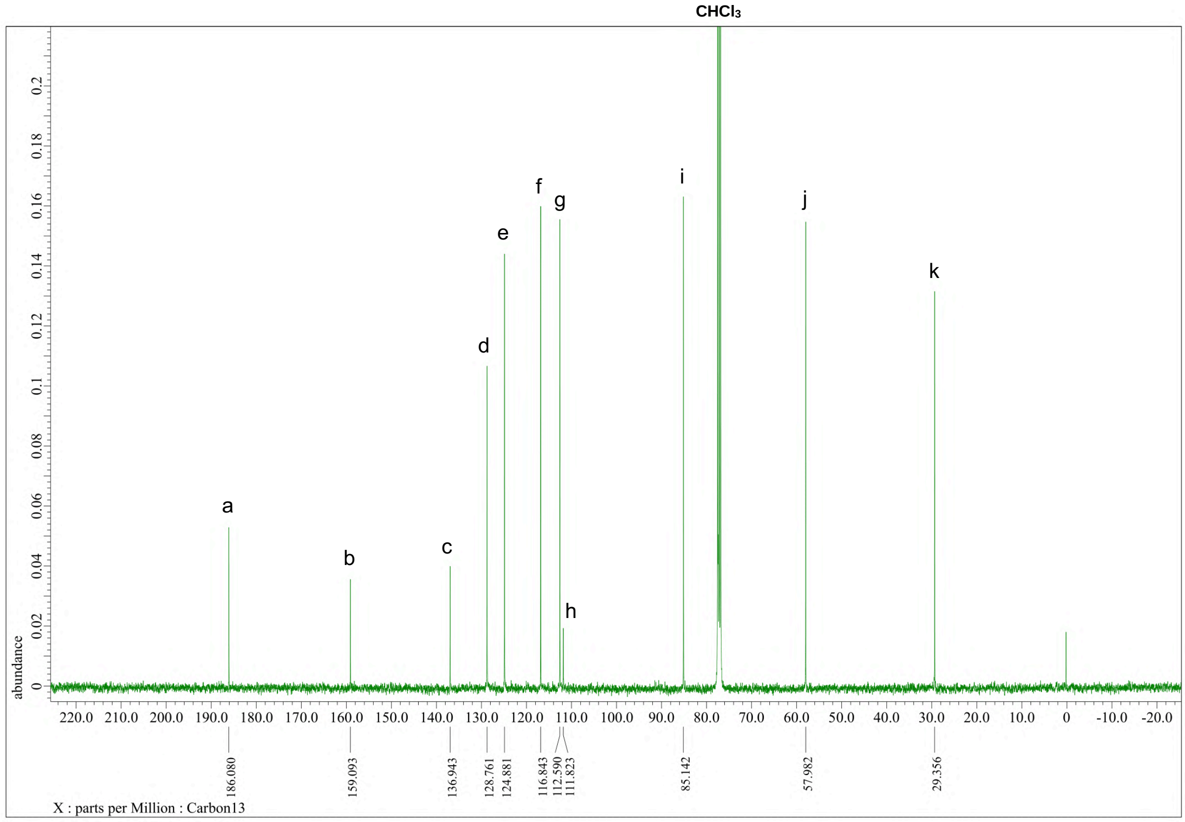
CHCl3
a
b
c
d
e
f
g
h
i
j
k
abundance
186.080
159.093
136.943
128.761
124.881
116.843
112.590
111.823
85.142
57.982
29.356
X : parts per Million : Carbon13

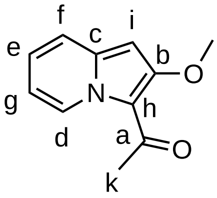
f
i
j
e
c
b
O
N
g
h
d
a
O
k

^{1}H NMR (400 MHz) and ^{13}C NMR (100 MHz) spectra of **2c** ($CDCl_3$)

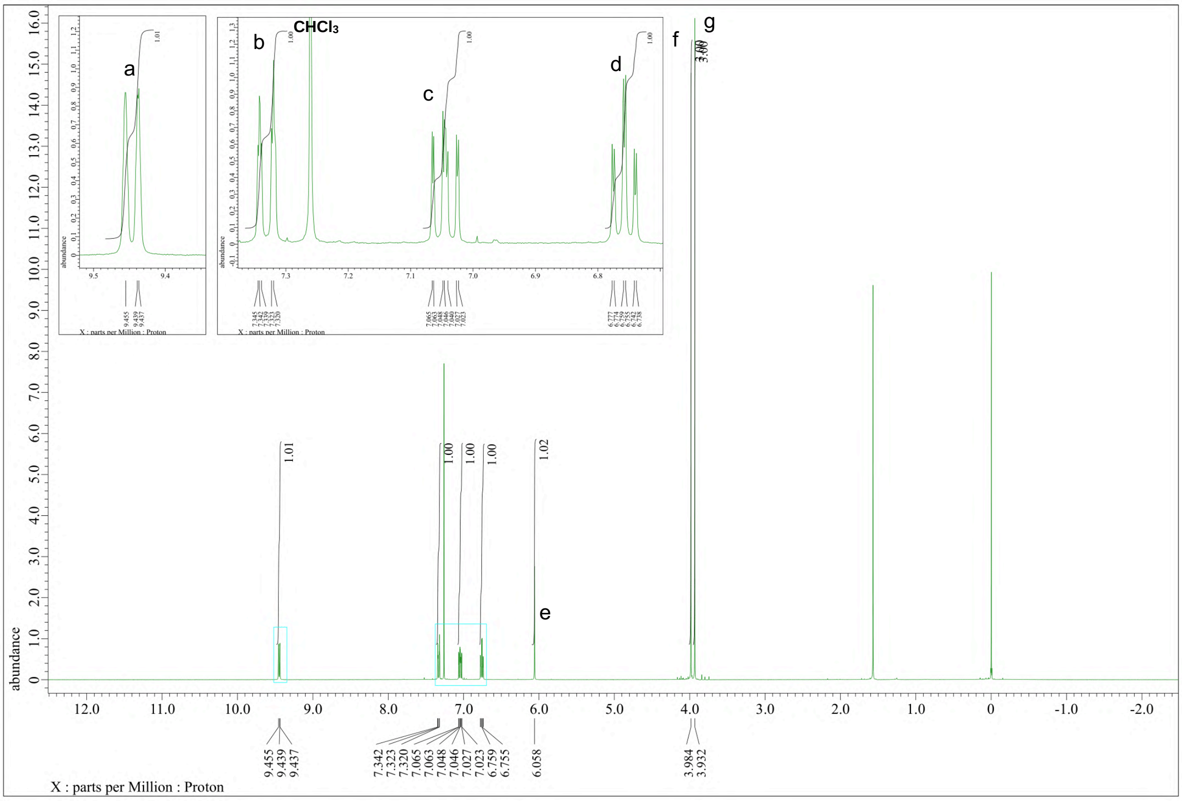

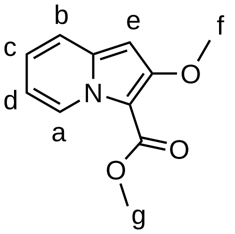

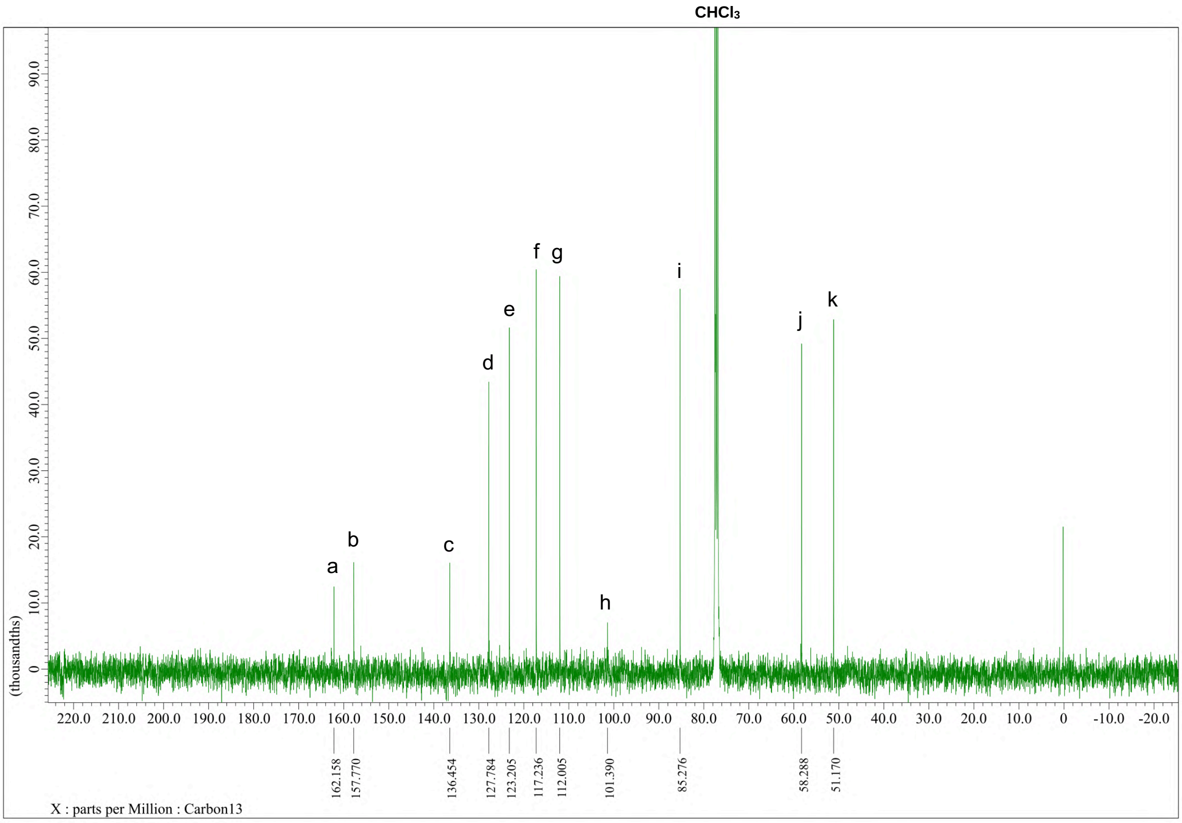
CHCl3
a
b
c
d
e
f
g
h
i
j
k
(thousandths)
162.158
157.770
136.454
127.784
123.205
117.236
112.005
101.390
85.276
58.288
51.170
X : parts per Million : Carbon13

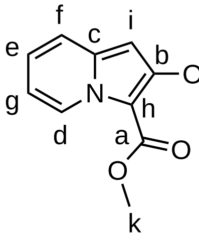
f
i
j
c
e
b
O
N
g
h
d
a
O
O
k

^{1}H NMR (400 MHz) and ^{13}C NMR (100 MHz) spectra of **2e** ($CDCl_3$)

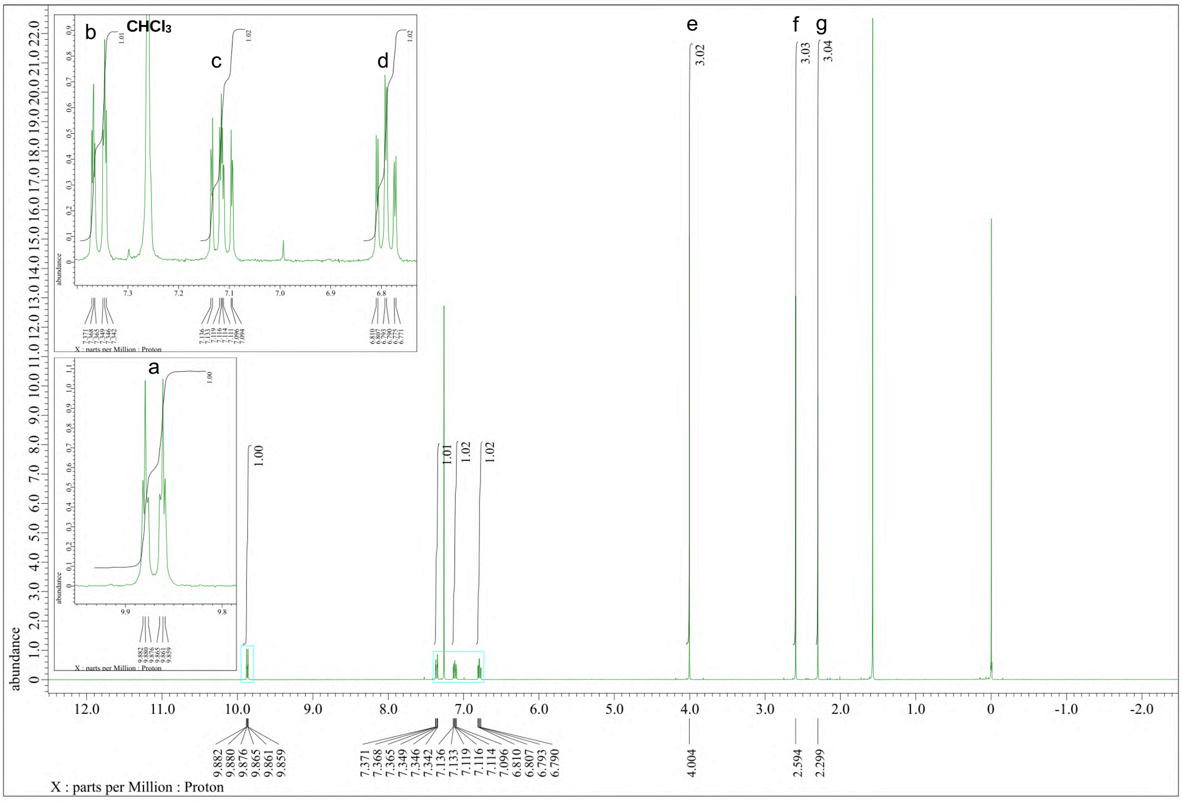

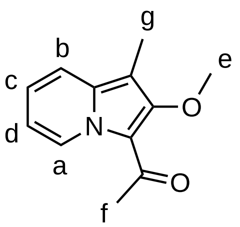

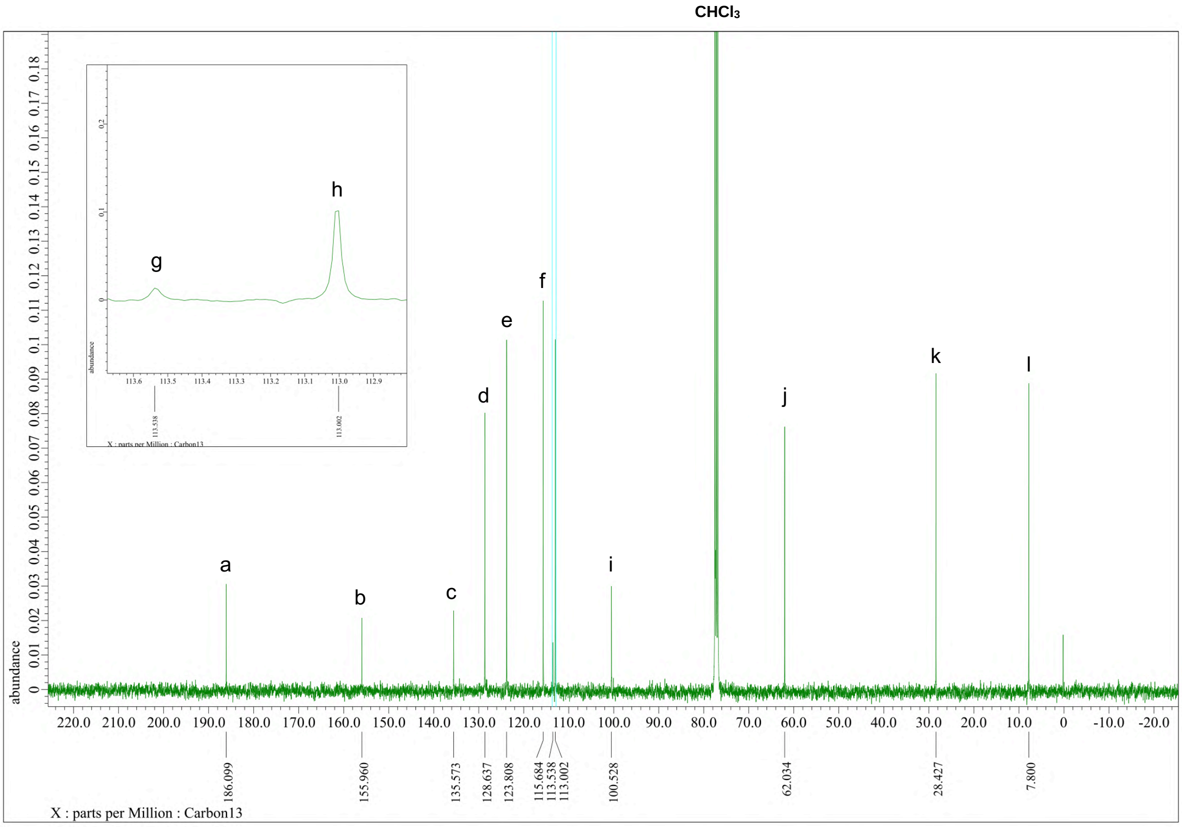
CHCl3
a
b
c
d
e
f
g
h
i
j
k
l
186.099
155.960
135.573
128.637
123.808
115.684
113.538
113.002
100.528
62.034
28.427
7.800
abundance
X : parts per Million : Carbon13

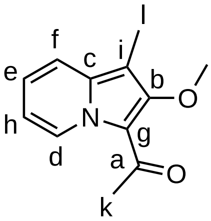
N
O
O
a
b
c
d
e
f
g
h
i
j
k
l

^{1}H NMR (400 MHz) and ^{13}C NMR (100 MHz) spectra of **2f** ($CDCl_3$)

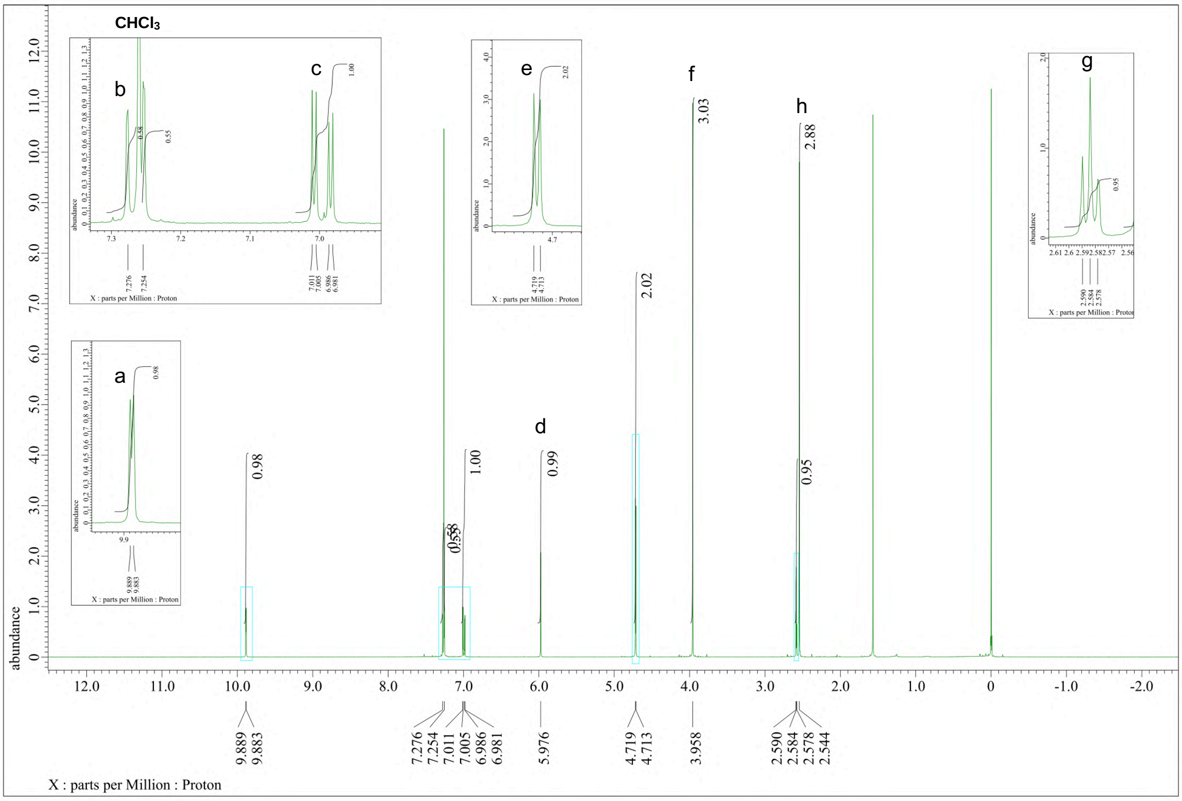

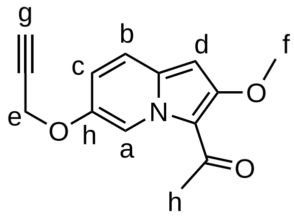

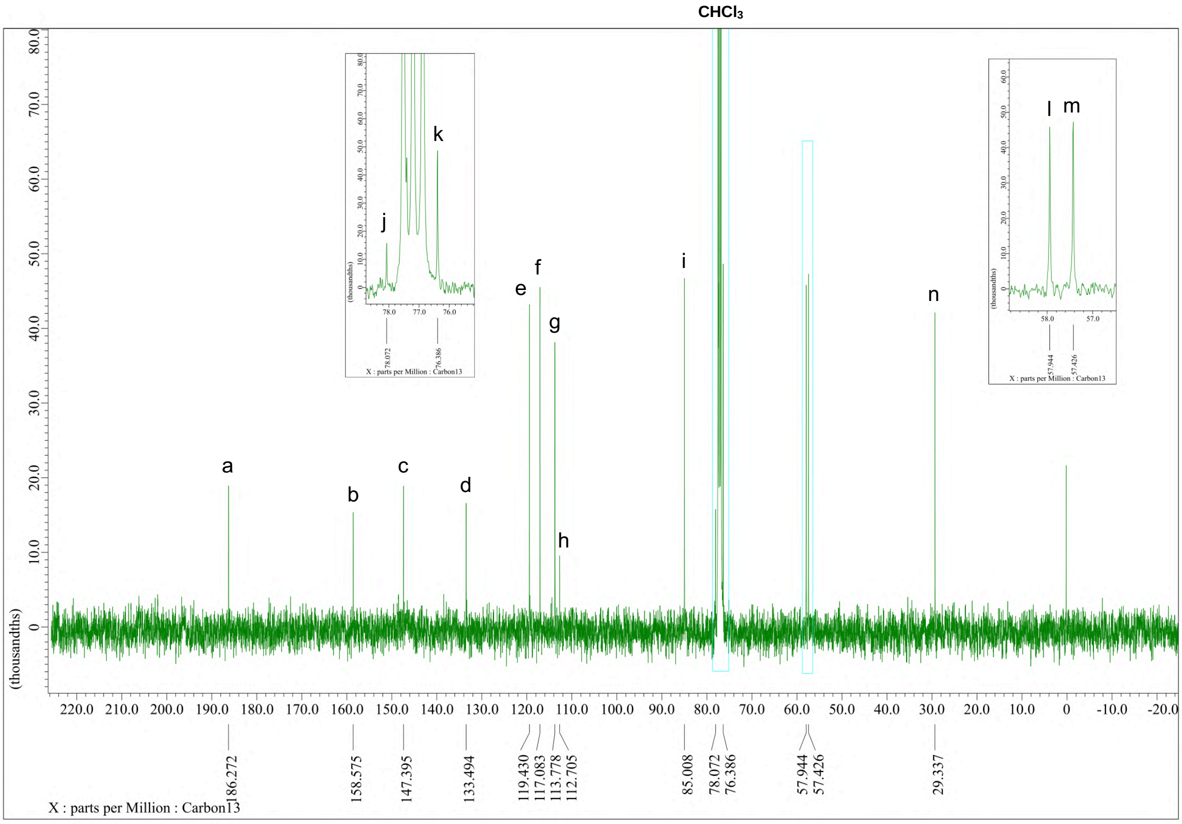
CHCl3
a
b
c
d
e
f
g
h
i
j
k
l
m
n
186.272
158.575
147.395
133.494
119.430
117.083
113.778
112.705
85.008
78.072
76.386
57.944
57.426
29.337
X : parts per Million : Carbon13
(thousandths)

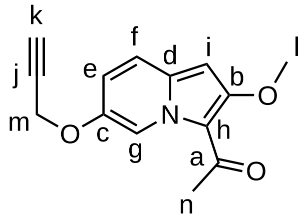
k
j
m
O
c
g
e
f
d
i
b
O
l
N
h
a
O
n

^{1}H NMR (400 MHz) and ^{13}C NMR (100 MHz) spectra of **2g** ($CDCl_3$)

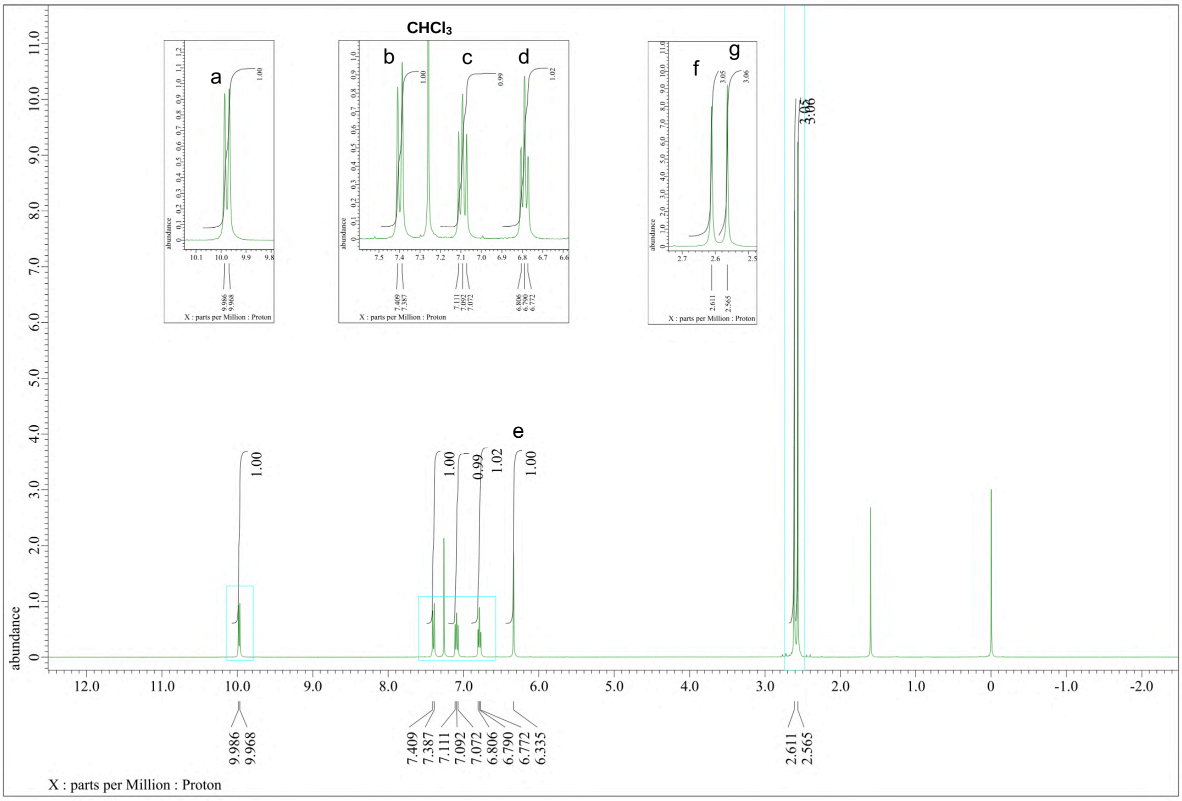

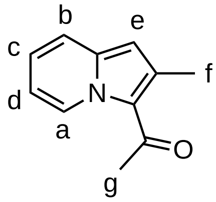

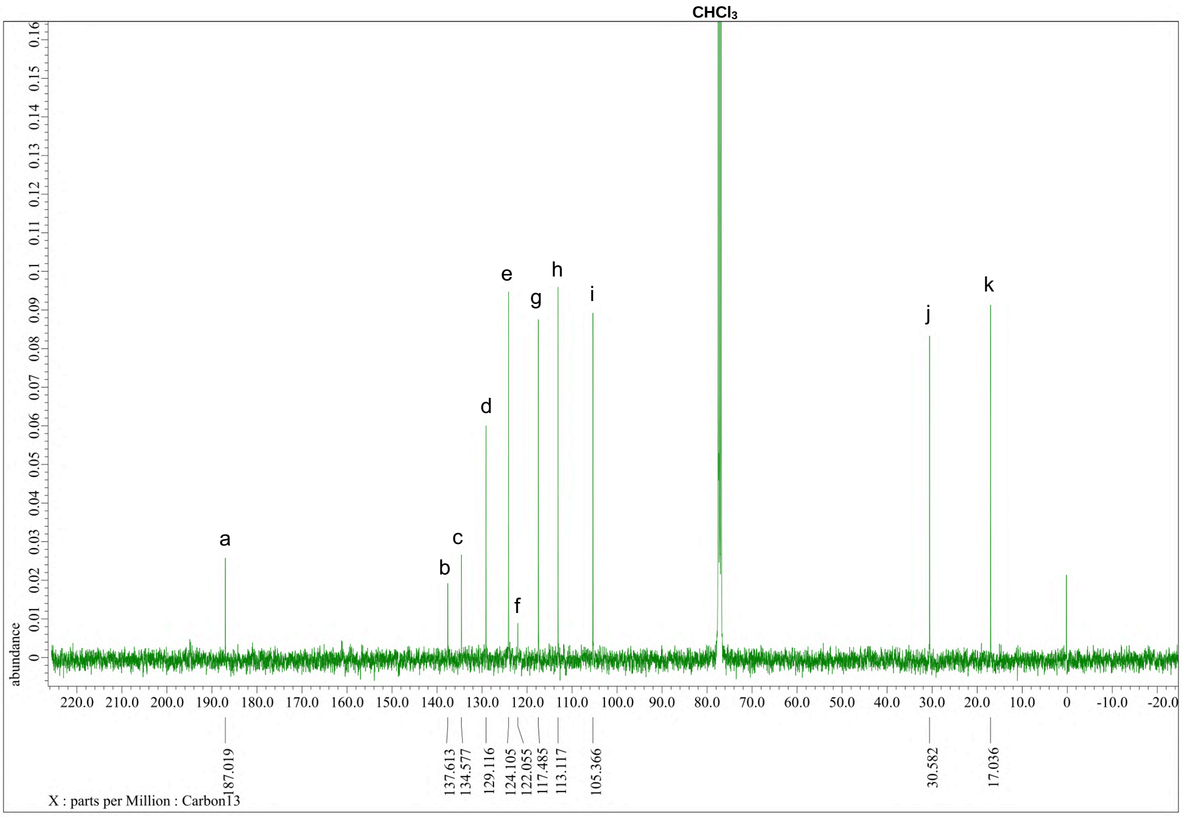
CHCl3
a
b
c
d
e
f
g
h
i
j
k
abundance
0 0.01 0.02 0.03 0.04 0.05 0.06 0.07 0.08 0.09 0.1 0.11 0.12 0.13 0.14 0.15 0.16
220.0 210.0 200.0 190.0 180.0 170.0 160.0 150.0 140.0 130.0 120.0 110.0 100.0 90.0 80.0 70.0 60.0 50.0 40.0 30.0 20.0 10.0 0 -10.0 -20.0
187.019
137.613
134.577
129.116
124.105
122.055
117.485
113.117
105.366
30.582
17.036
X : parts per Million : Carbon13

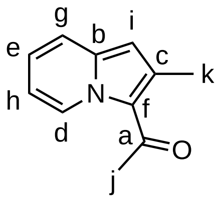
g
i
e
b
c
k
h
N
f
d
a
O
j

^{1}H NMR (400 MHz) and ^{13}C NMR (100 MHz) spectra of **2h** ($CDCl_3$)

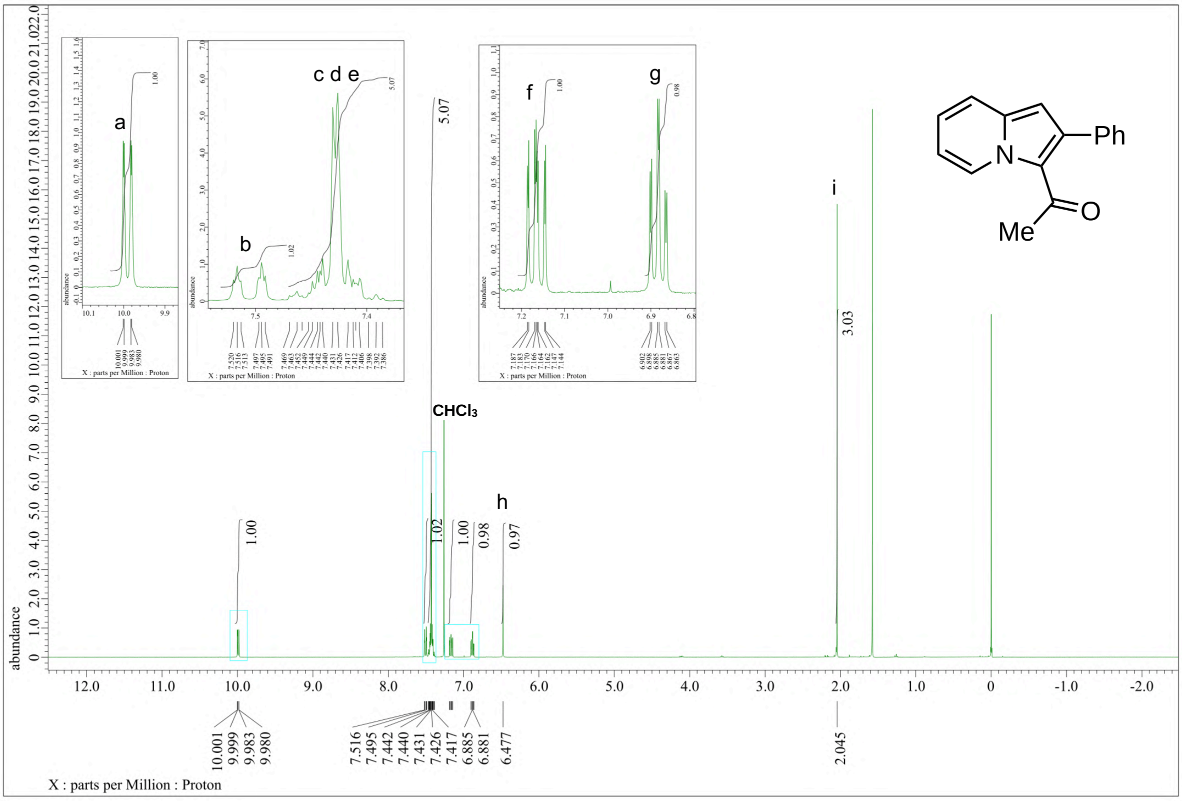

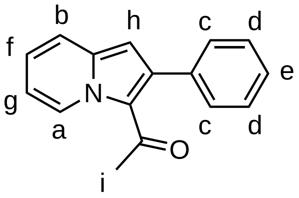

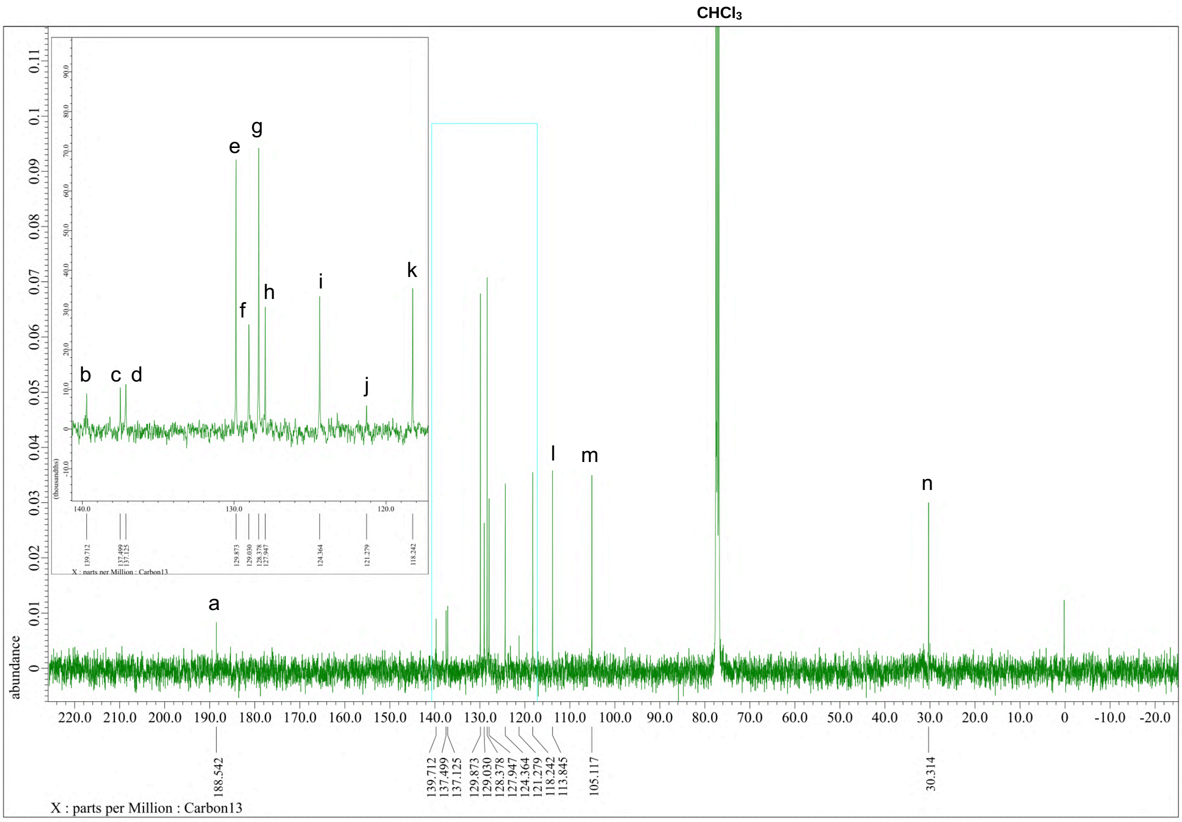
CHCl3
abundance
X : parts per Million : Carbon13
188.542
139.712
137.499
137.125
129.873
129.030
128.378
127.947
124.364
121.279
118.242
113.845
105.117
30.314
a
b
c
d
e
f
g
h
i
j
k
l
m
n

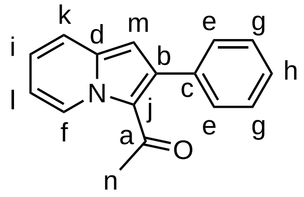
k
i
l
f
d
m
b
c
e
g
h
j
a
n
N
O

^{1}H NMR (400 MHz) and ^{13}C NMR (100 MHz) spectra of 1-(2-methoxy-5-methylindolizin-3-yl)ethan-1-one (CD_3OD)

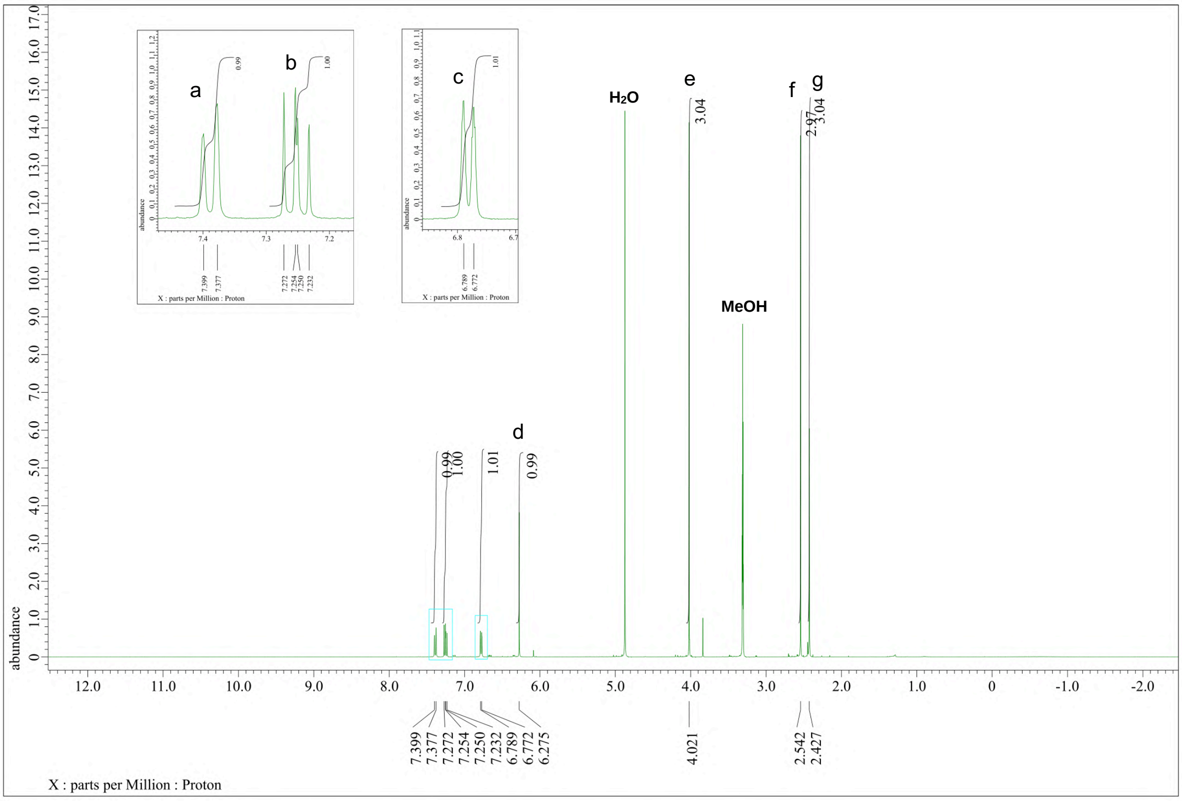

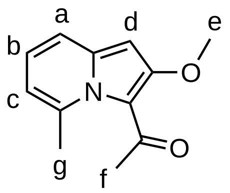

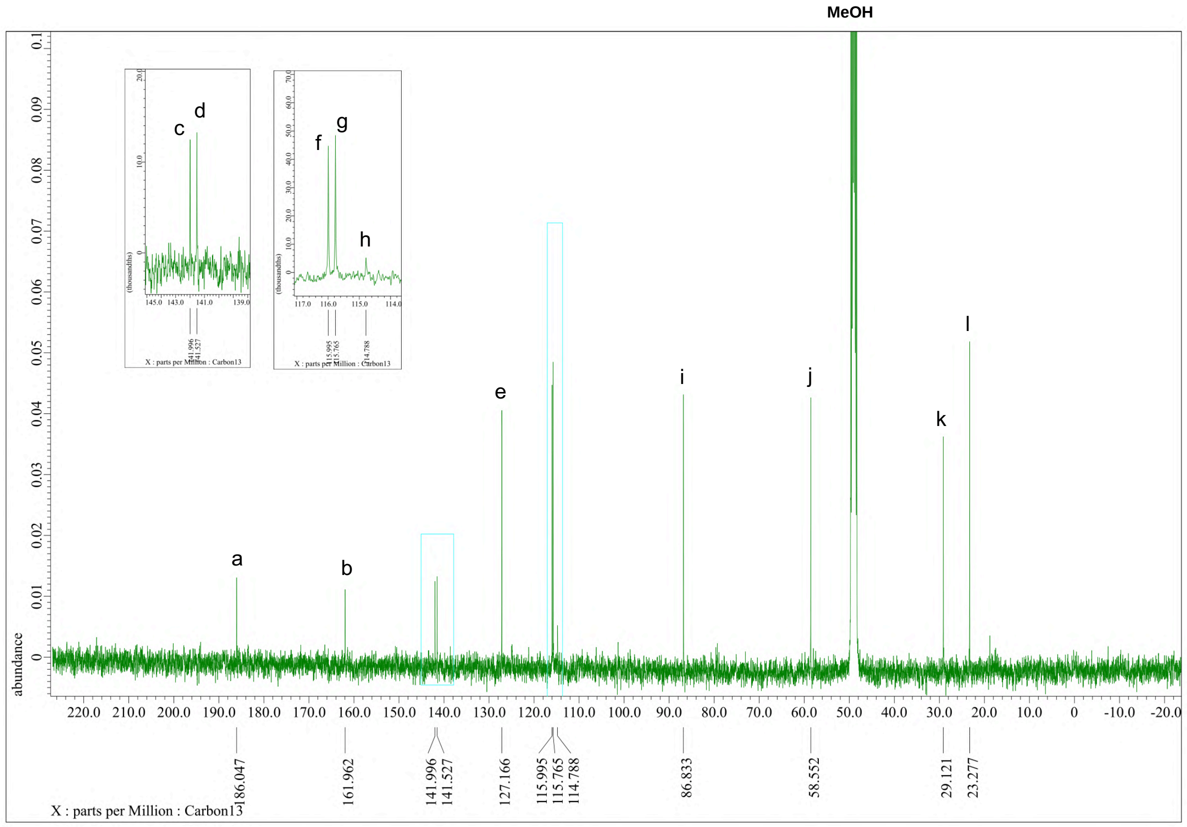
MeOH
abundance
X : parts per Million : Carbon13
a
b
e
i
j
k
l
c
d
f
g
h
186.047
161.962
141.996
141.527
127.166
115.995
115.765
114.788
86.833
58.552
29.121
23.277

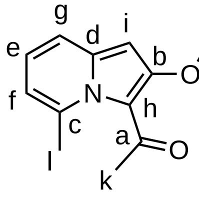
g
d
i
j
e
b
O
f
N
h
c
a
O
l
k

^{1}H NMR (400 MHz) and ^{13}C NMR (100 MHz) spectra of methyl 2-amino-3-(4-azidophenyl)propanoate ($CDCl_3$)

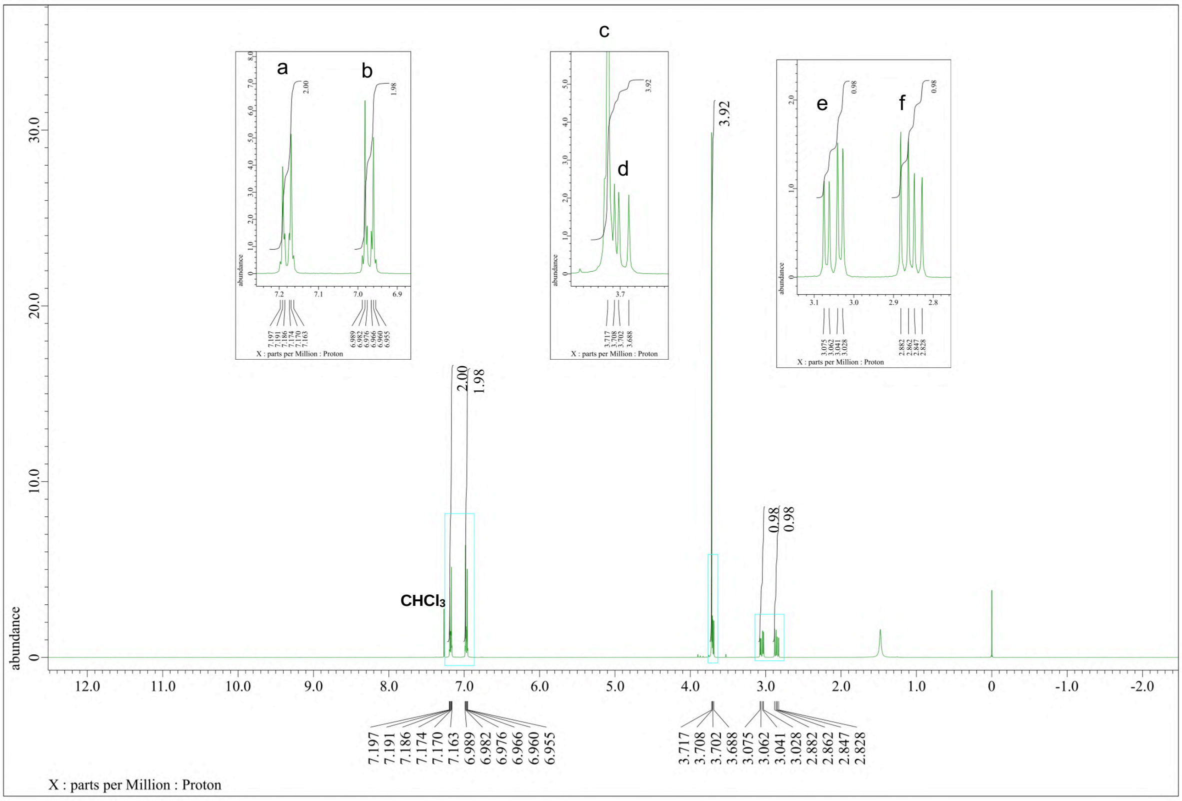

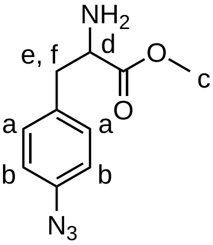

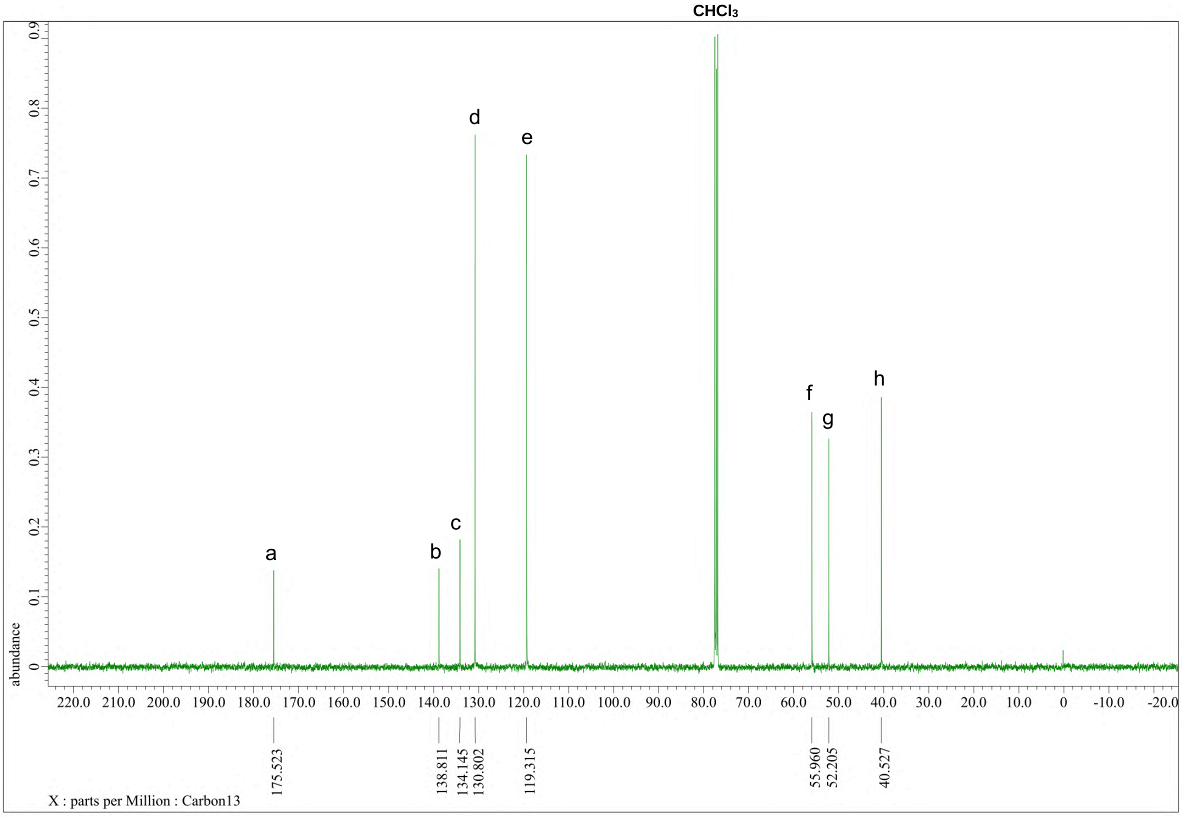
CHCl3
a
b
c
d
e
f
g
h
abundance
0 0.1 0.2 0.3 0.4 0.5 0.6 0.7 0.8 0.9
220.0 210.0 200.0 190.0 180.0 170.0 160.0 150.0 140.0 130.0 120.0 110.0 100.0 90.0 80.0 70.0 60.0 50.0 40.0 30.0 20.0 10.0 0 -10.0 -20.0
175.523
138.811
134.145
130.802
119.315
55.960
52.205
40.527
X : parts per Million : Carbon13

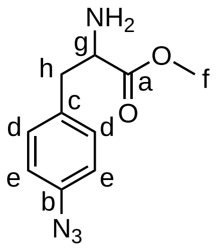
NH2
g
h
O
a
f
c
O
d
d
e
e
b
N3

^{1}H NMR (400 MHz) and ^{13}C NMR (100 MHz) spectra of *tert*-butyl 4-((6-methylpyridin-3-yl)oxy)butanoate ($CDCl_3$)

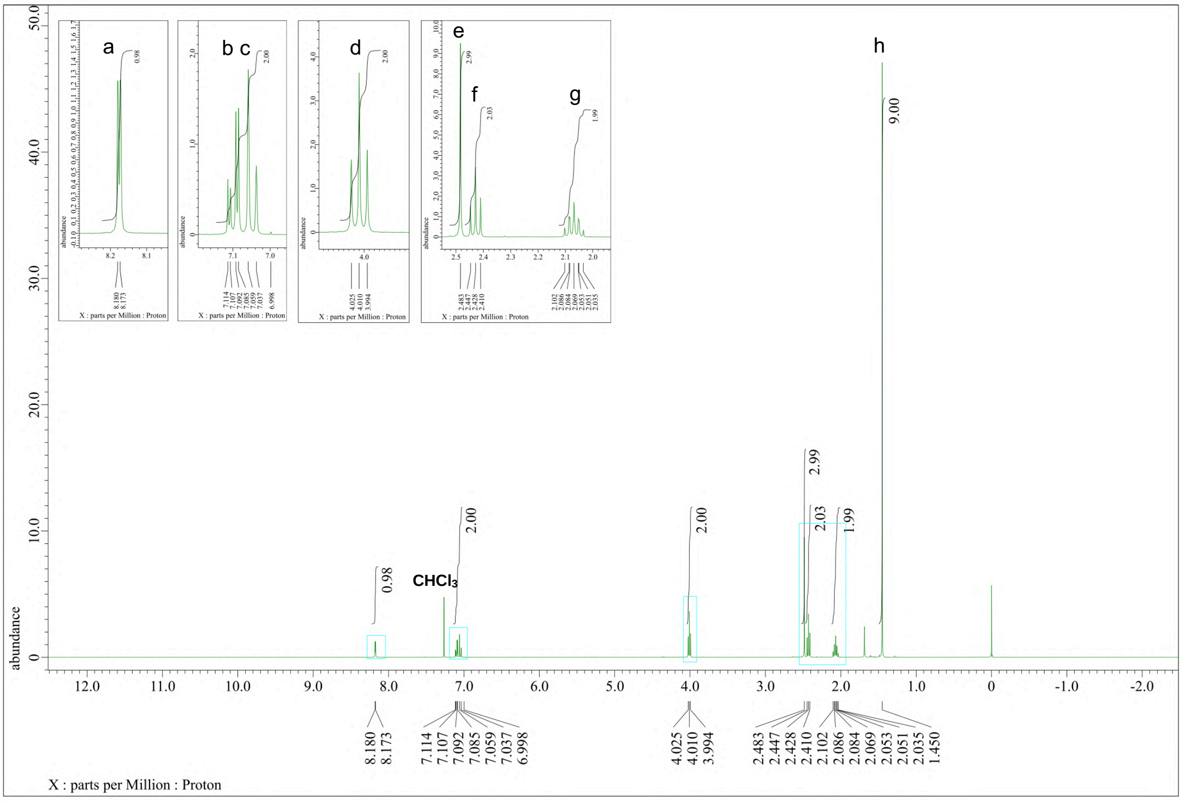

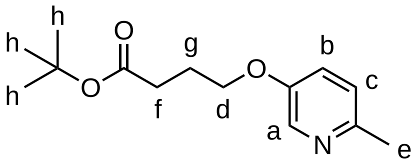

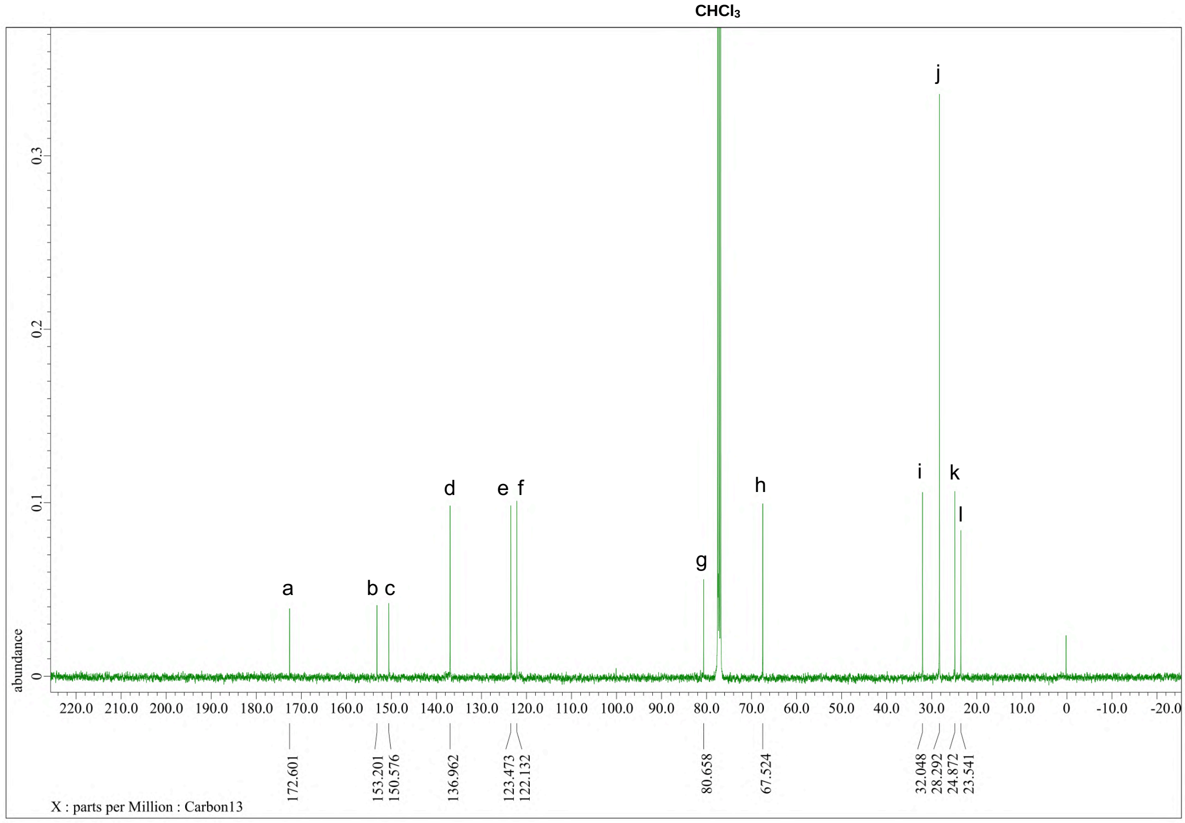
CHCl3
j
i
k
l
h
g
d
e f
a
b c
abundance
0.3
0.2
0.1
0
220.0 210.0 200.0 190.0 180.0 170.0 160.0 150.0 140.0 130.0 120.0 110.0 100.0 90.0 80.0 70.0 60.0 50.0 40.0 30.0 20.0 10.0 0 -10.0 -20.0
172.601
153.201
150.576
136.962
123.473
122.132
80.658
67.524
32.048
28.292
24.872
23.541
X : parts per Million : Carbon13

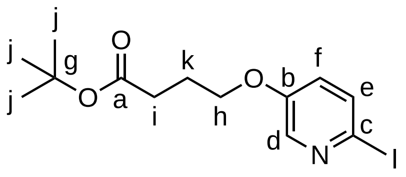
j
j
j
g
O
a
O
k
i
h
O
b
f
e
c
d
N
I

^{1}H NMR (400 MHz) and ^{13}C NMR (100 MHz) spectra of 5-(4-(*tert*-butoxy)-4-oxobutoxy)-1-(2-ethoxy-2-oxoethyl)-2-methylpyridin-1-ium bromide ($CDCl_3$)

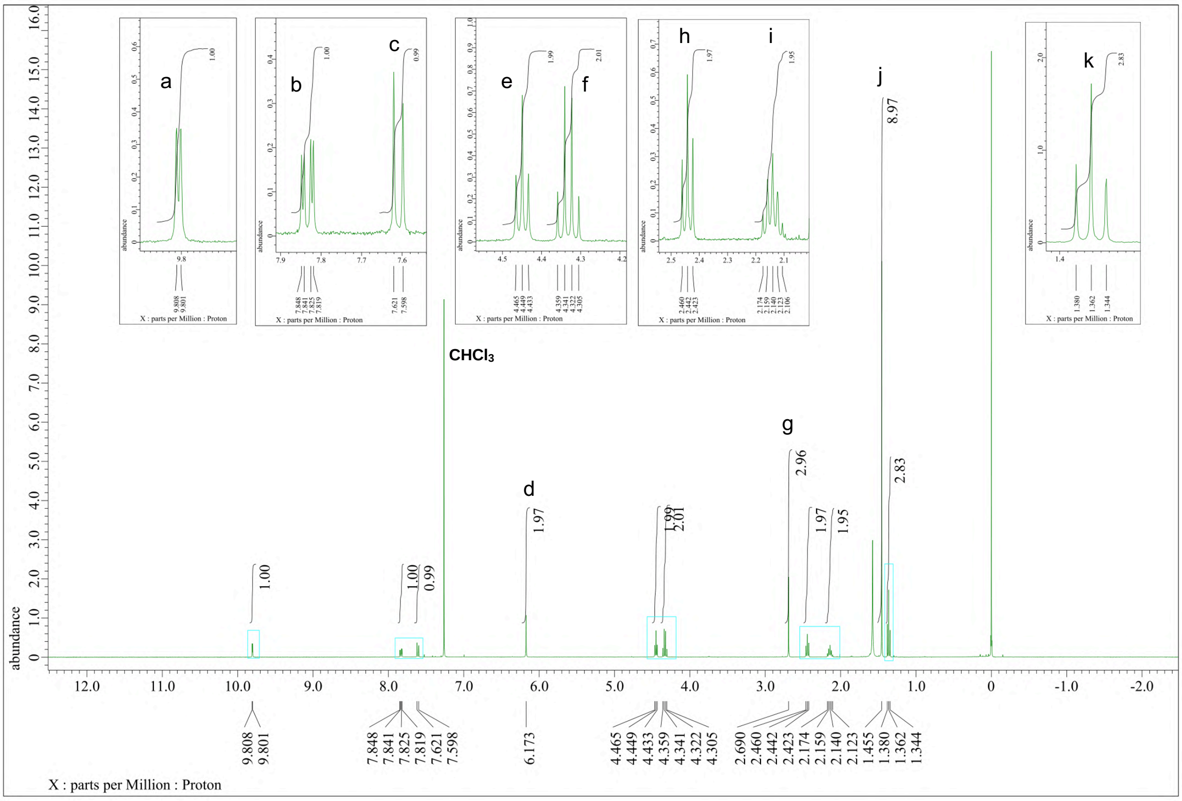

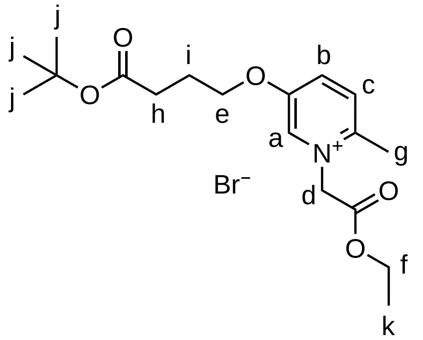

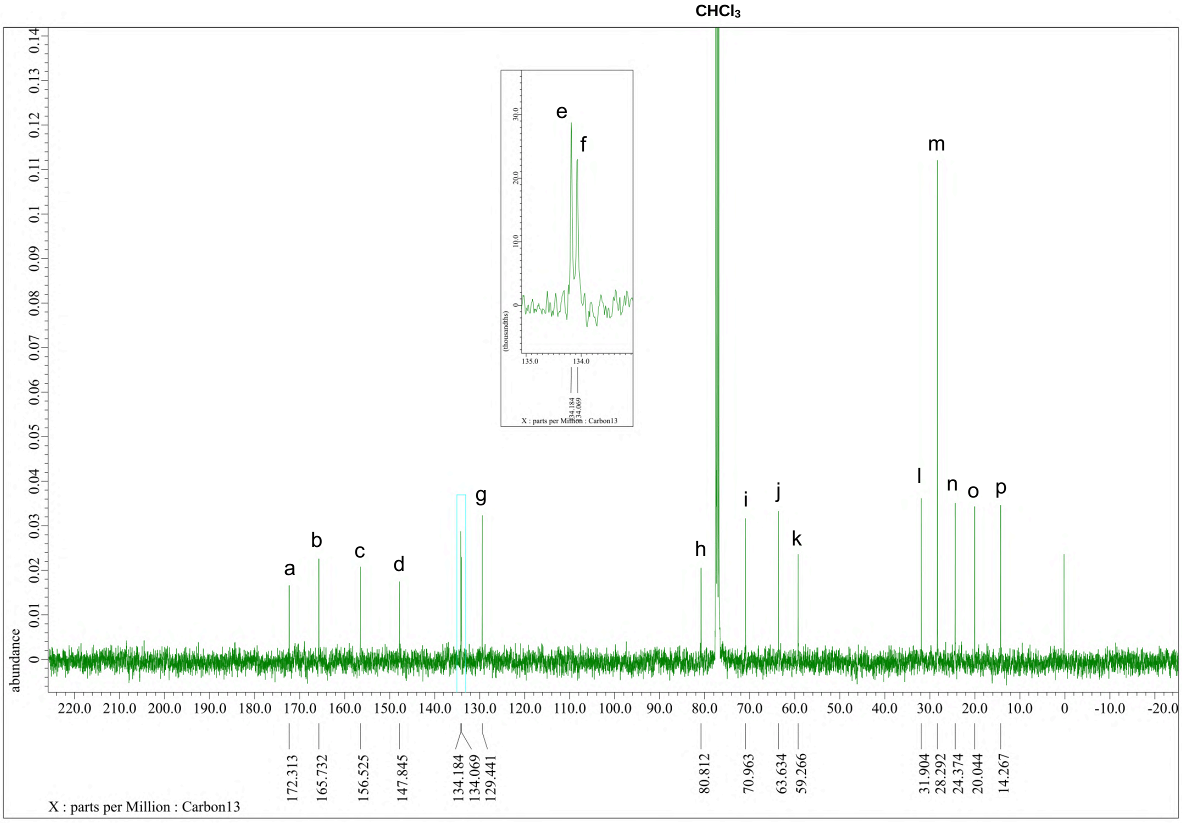
CHCl3
abundance
X : parts per Million : Carbon13
172.313
165.732
156.525
147.845
134.184
134.069
129.441
80.812
70.963
63.634
59.266
31.904
28.292
24.374
20.044
14.267
a
b
c
d
e
f
g
h
i
j
k
l
m
n
o
p

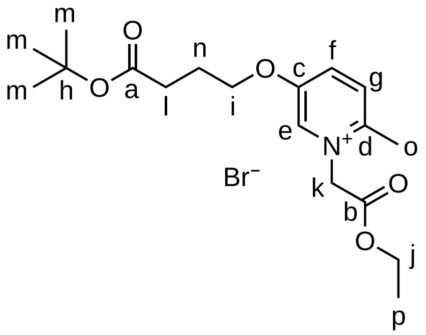
Br⁻
N⁺
O
a
b
c
d
e
f
g
h
i
j
k
l
m
n
o
p

^{1}H NMR (400 MHz) and ^{13}C NMR (100 MHz) spectra of *tert*-butyl 4-((2-methoxyindolizin-6-yl)oxy)butanoate ($CDCl_3$)

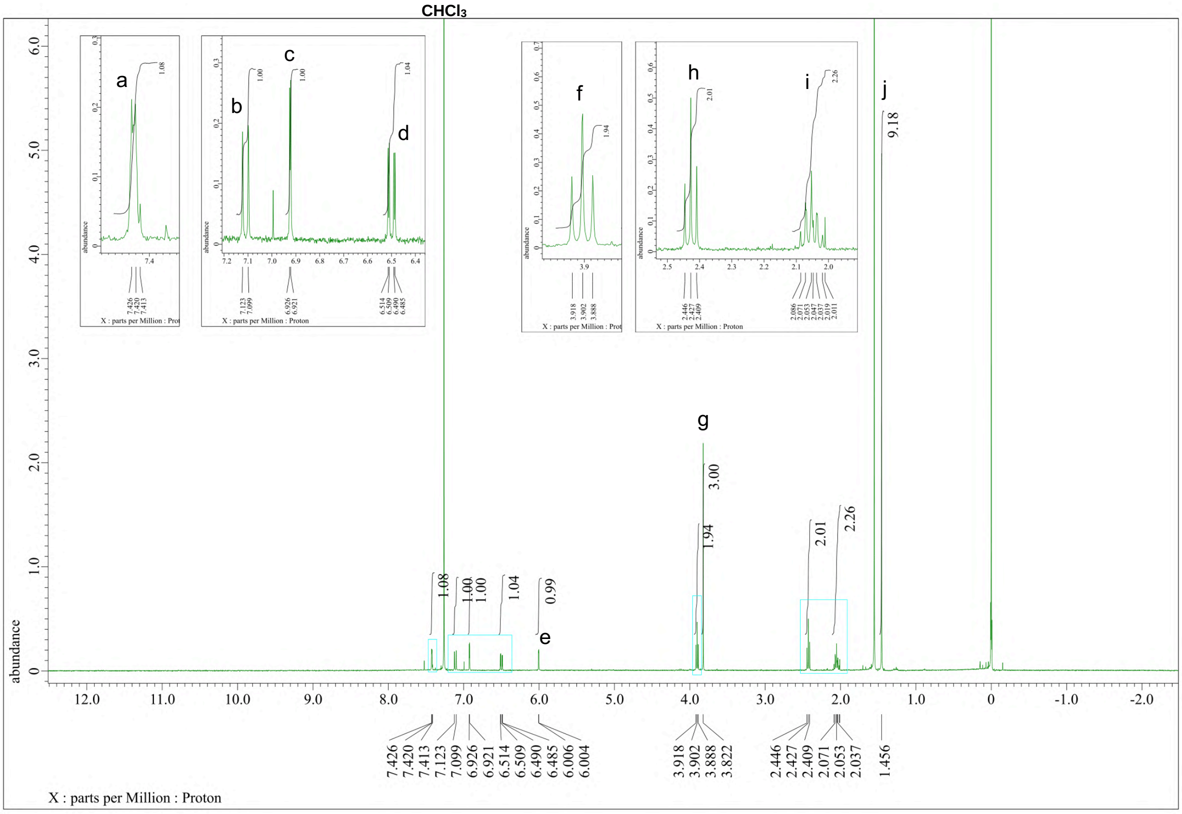

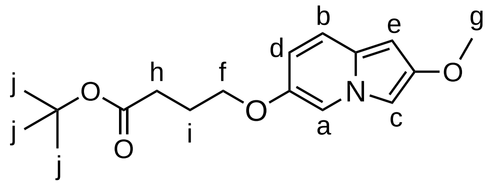

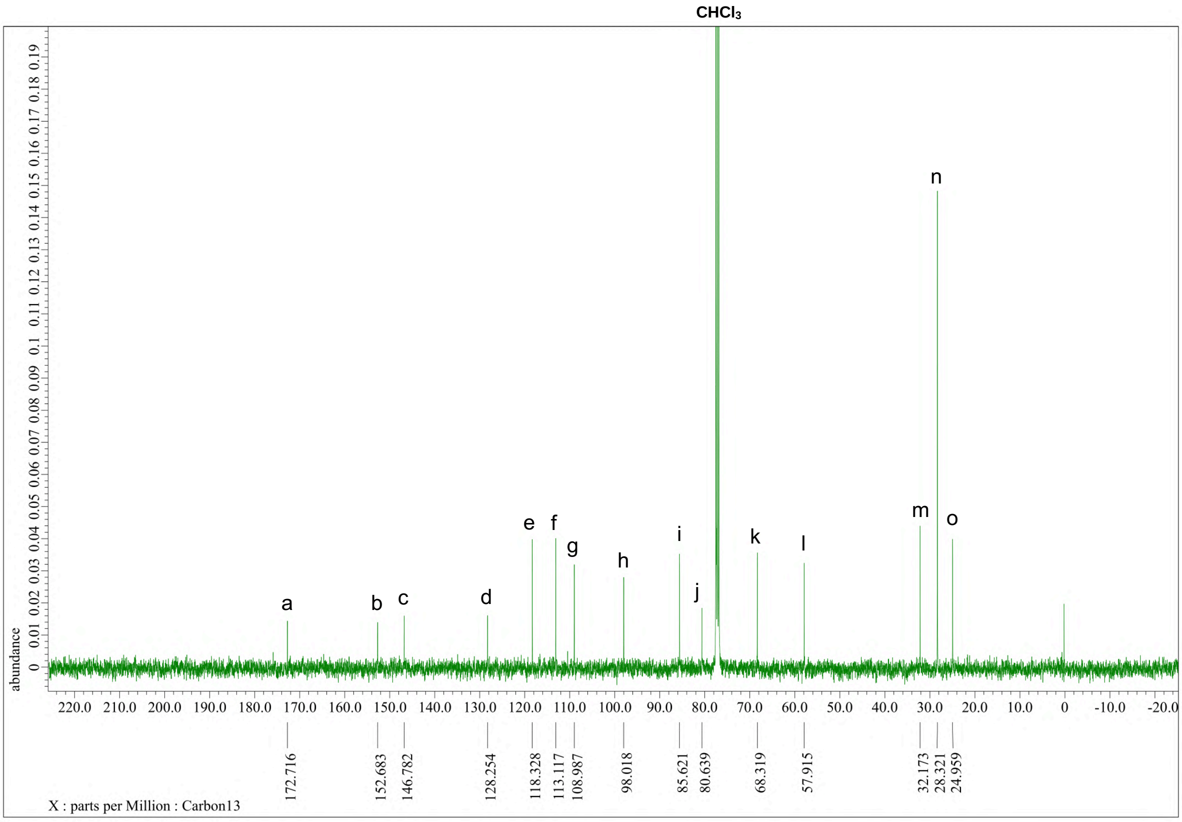
CHCl3
n
m
o
e
f
g
h
i
j
k
l
a
b
c
d
abundance
0 0.01 0.02 0.03 0.04 0.05 0.06 0.07 0.08 0.09 0.1 0.11 0.12 0.13 0.14 0.15 0.16 0.17 0.18 0.19
220.0 210.0 200.0 190.0 180.0 170.0 160.0 150.0 140.0 130.0 120.0 110.0 100.0 90.0 80.0 70.0 60.0 50.0 40.0 30.0 20.0 10.0 0 -10.0 -20.0
172.716
152.683
146.782
128.254
118.328
113.117
108.987
98.018
85.621
80.639
68.319
57.915
32.173
28.321
24.959
X : parts per Million : Carbon13

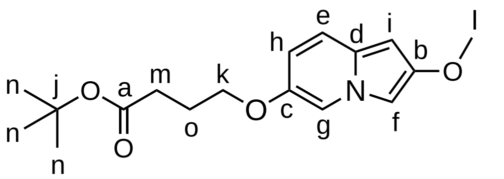
n
j
O
a
m
k
e
d
i
l
h
b
O
n
O
c
N
n
O
o
g
f

^{1}H NMR (400 MHz) and ^{13}C NMR (100 MHz) spectra of *tert*-butyl 4-((3-acetyl-2-methoxyindolizin-6-yl)oxy)butanoate ($CDCl_3$)

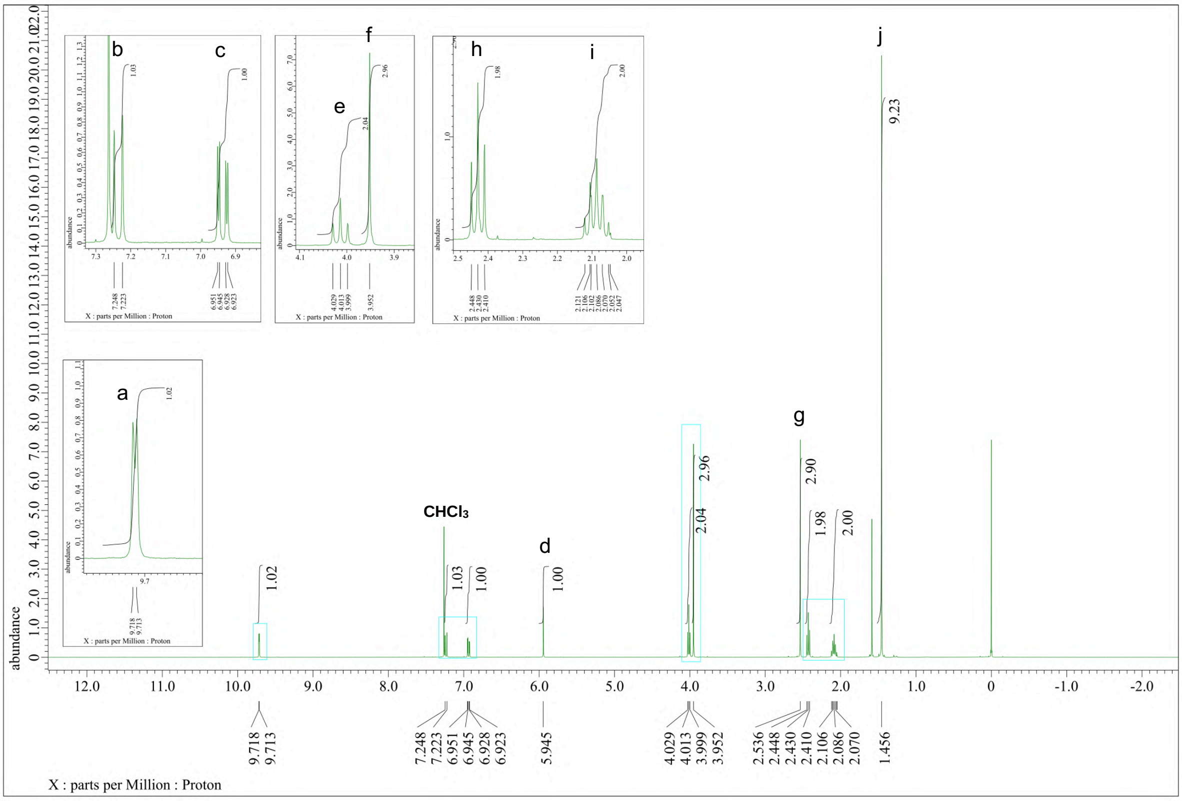

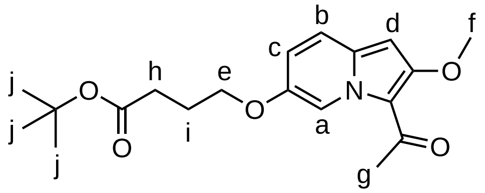

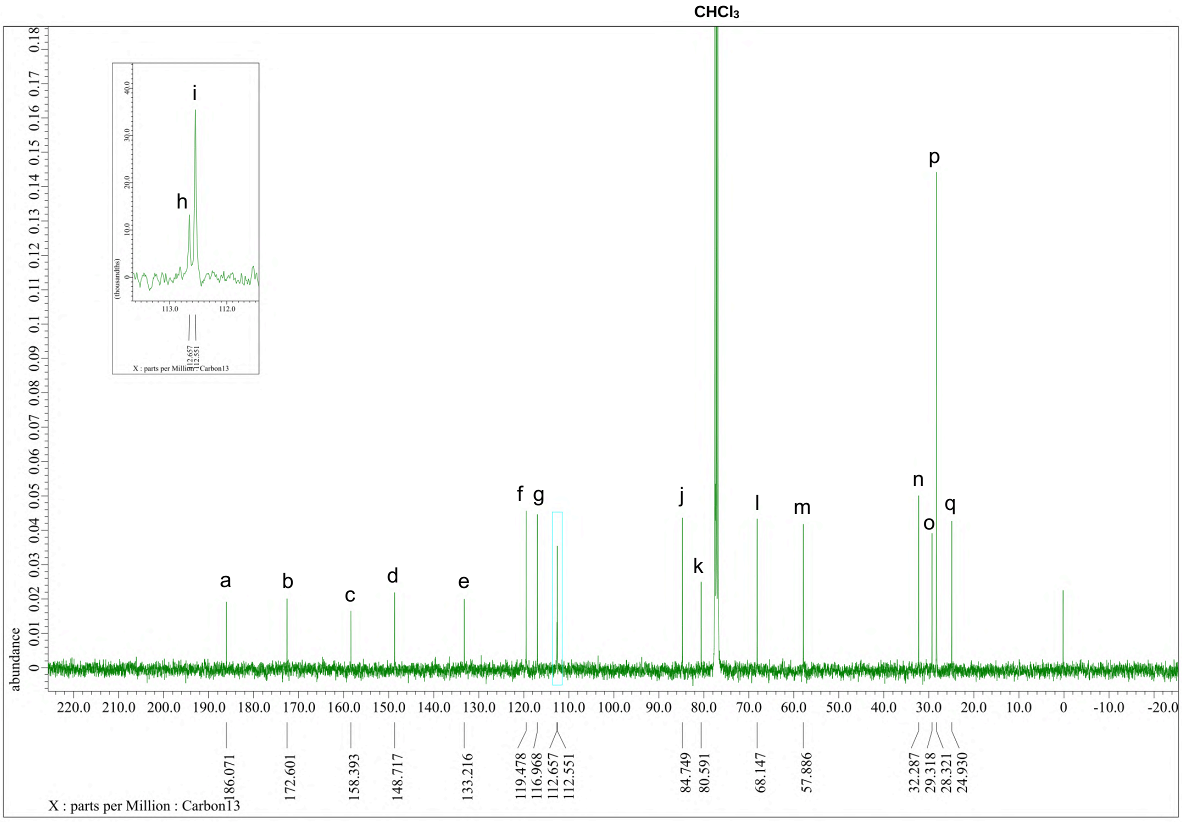
CHCl3
abundance
a
b
c
d
e
f g
h
i
j
k
l
m
n
o
p
q
186.071
172.601
158.393
148.717
133.216
119.478
116.968
112.657
112.551
84.749
80.591
68.147
57.886
32.287
29.318
28.321
24.930
X : parts per Million : Carbon13

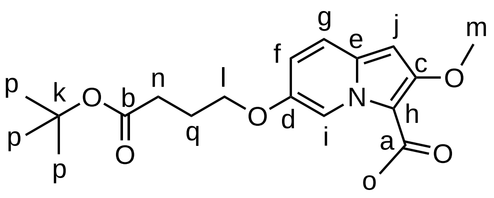

^{1}H NMR (400 MHz) and ^{13}C NMR (100 MHz) spectra of **2i** ($CDCl_3$)

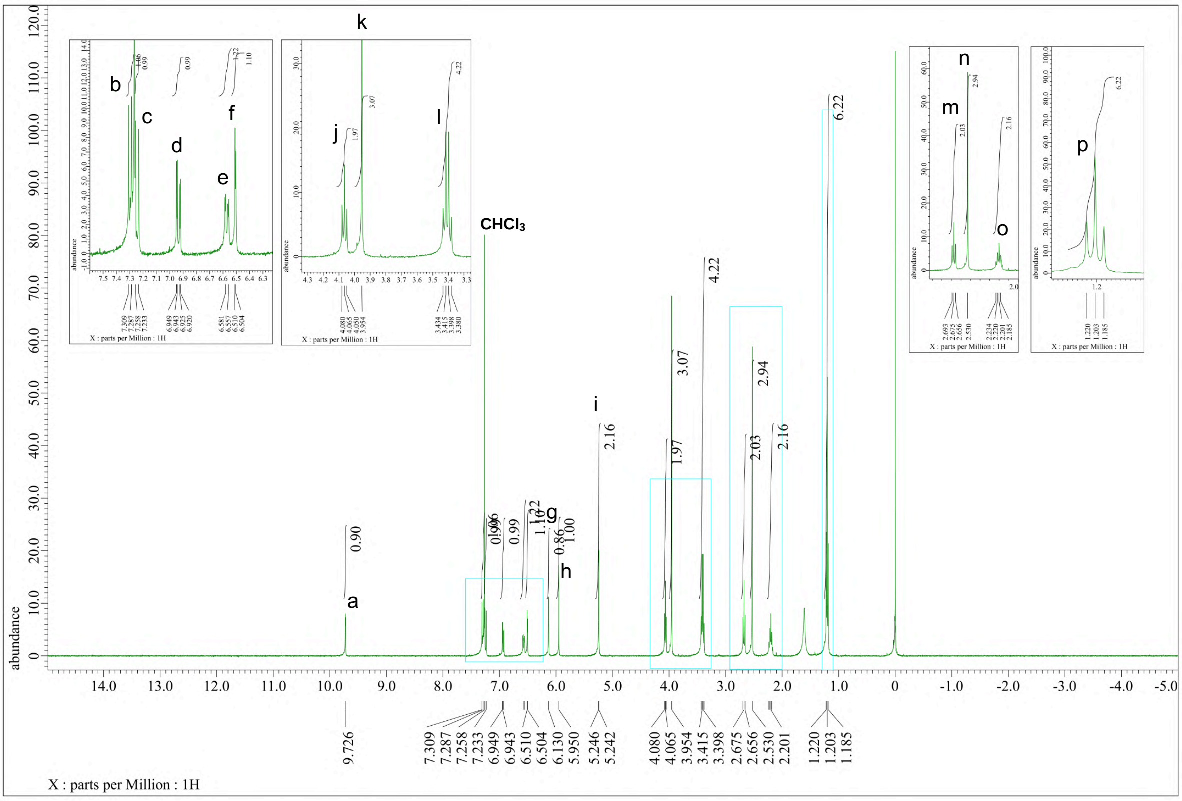

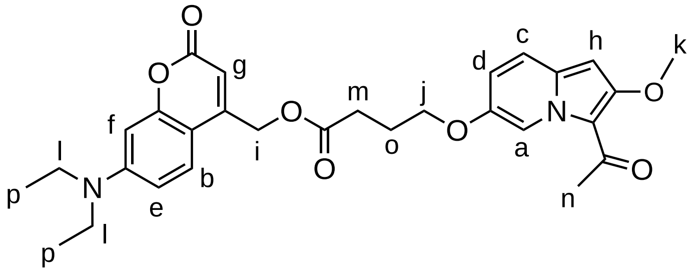

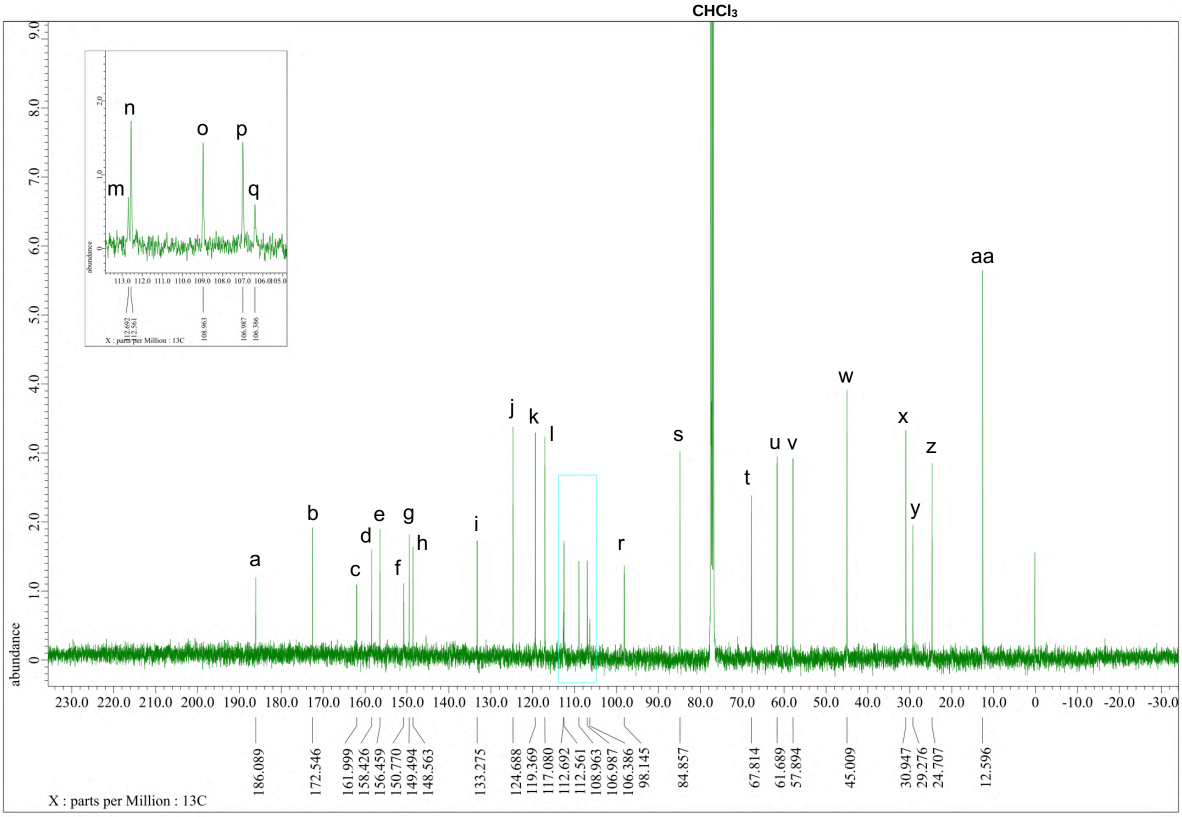
CHCl3
abundance
X : parts per Million : 13C
a
b
c
d
e
f
g
h
i
j
k
l
r
s
t
u v
w
x
y
z
aa
186.089
172.546
161.999
158.426
156.459
150.770
149.494
148.563
133.275
124.688
119.369
117.080
112.692
112.561
108.963
106.987
106.386
98.145
84.857
67.814
61.689
57.894
45.009
30.947
29.276
24.707
12.596
m
n
o
p
q

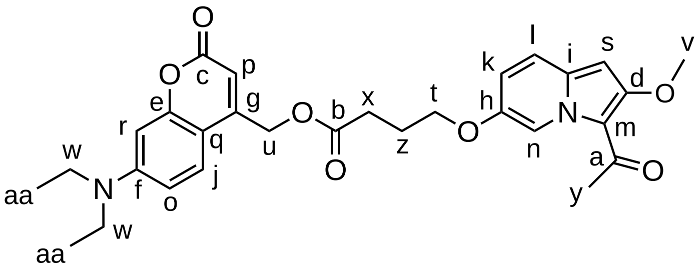

^{1}H NMR (400 MHz) and ^{13}C NMR (100 MHz) spectra of **2k** (DMSO-d_6)

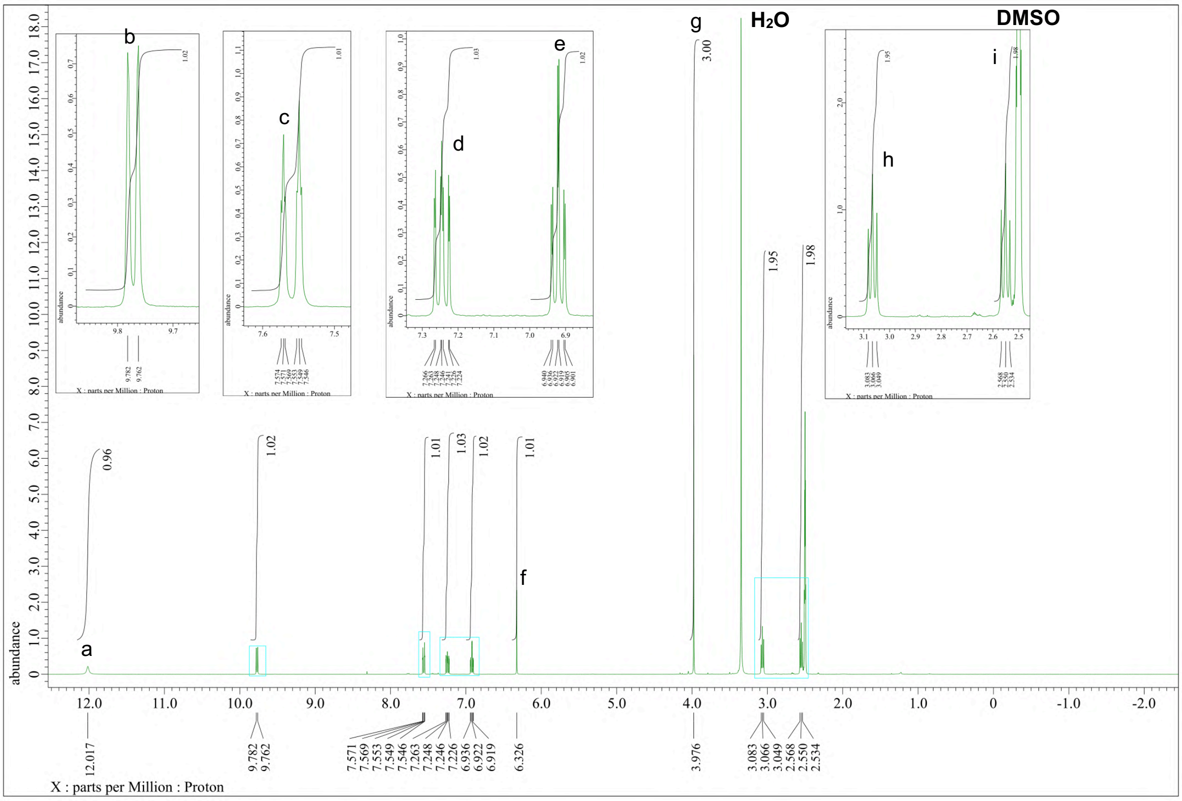

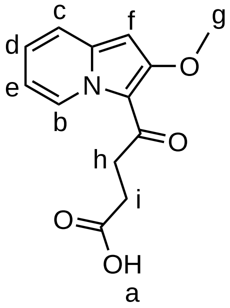

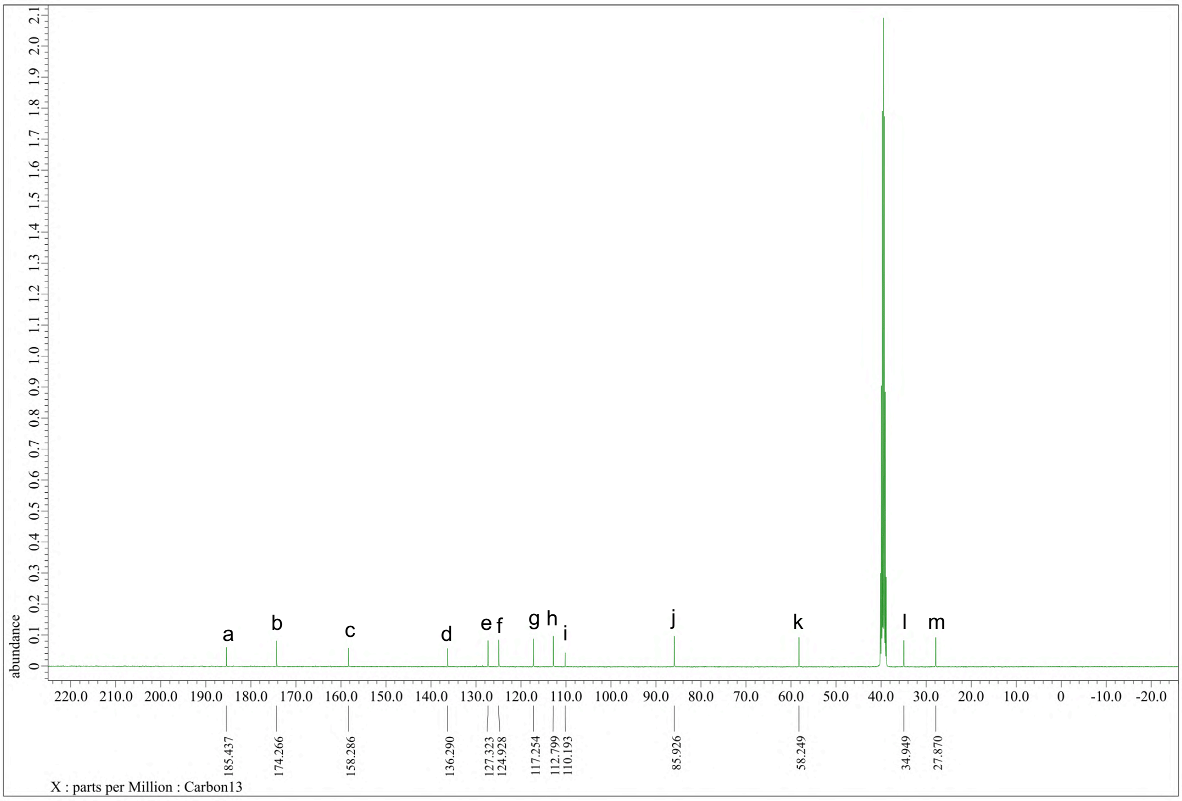
abundance
X : parts per Million : Carbon13
a
b
c
d
e
f
g
h
i
j
k
l
m
185.437
174.266
158.286
136.290
127.323
124.928
117.254
112.799
110.193
85.926
58.249
34.949
27.870

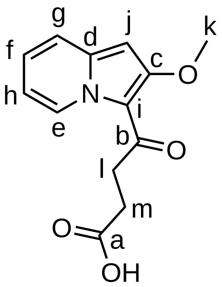
g
j
k
f
d
c
O
h
N
i
e
b
O
l
m
O
a
OH

^{1}H NMR (400 MHz) and ^{13}C NMR (100 MHz) spectra of **3a** ($CDCl_3$)

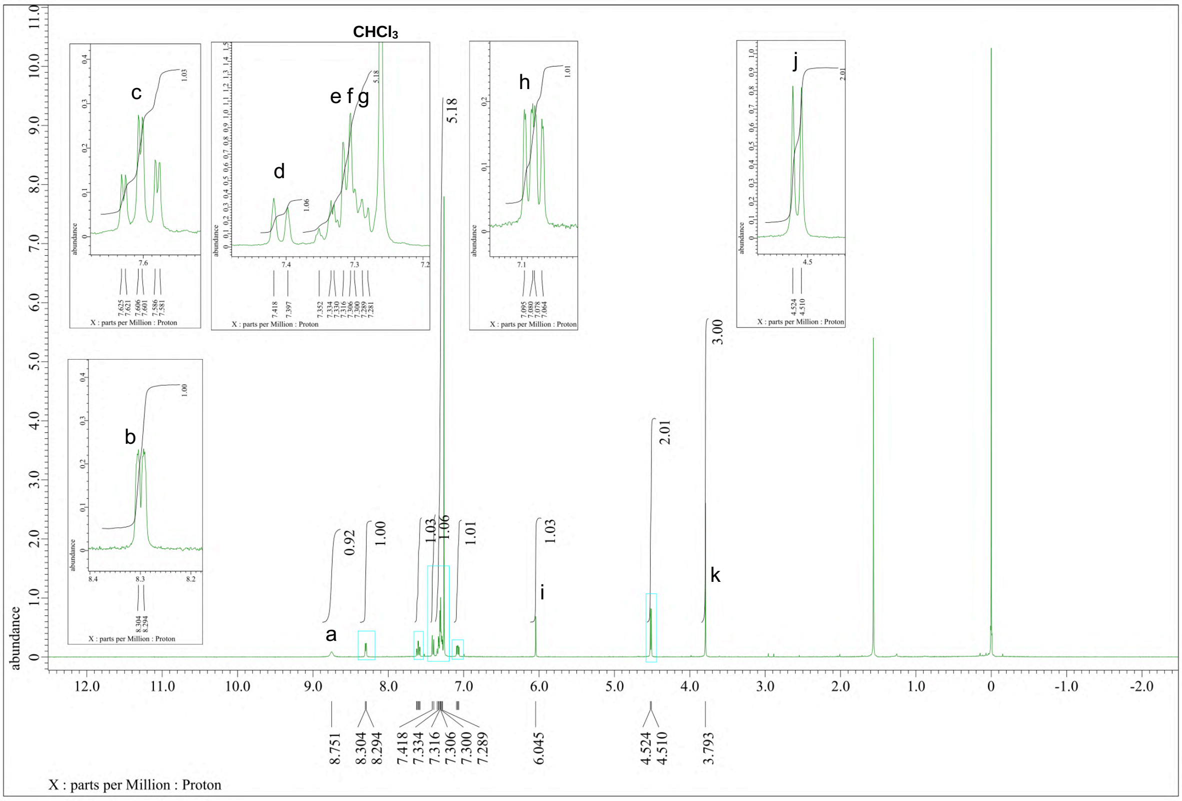

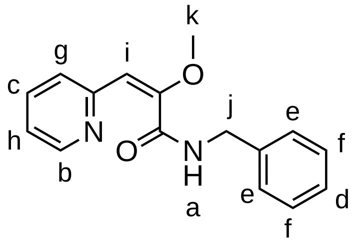

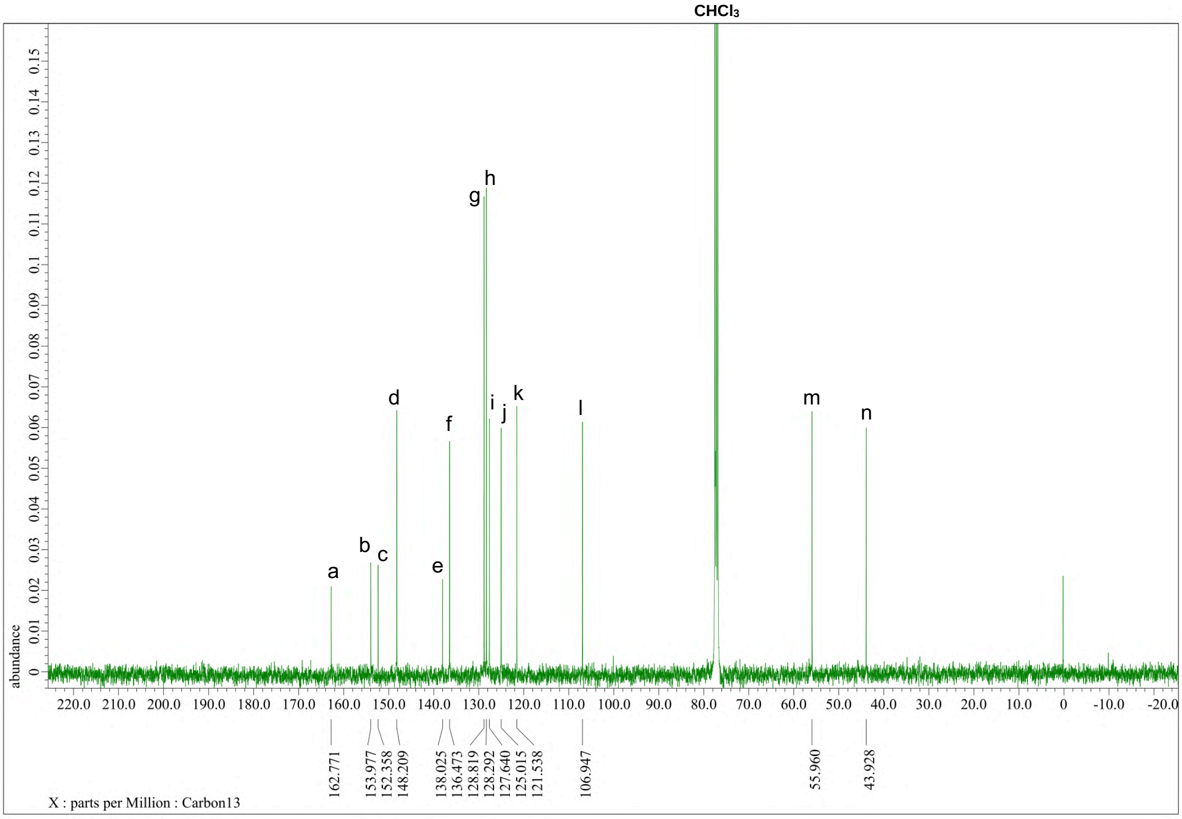
CHCl3
abundance
X : parts per Million : Carbon13
a
b
c
d
e
f
g
h
i
j
k
l
m
n
162.771
153.977
152.358
148.209
138.025
136.473
128.819
128.292
127.640
125.015
121.538
106.947
55.960
43.928

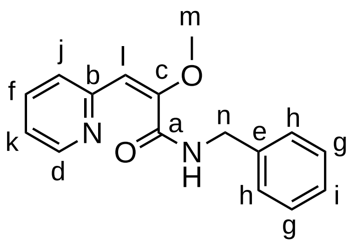
m
j
l
b
c
f
N
O
k
d
O
a
N
H
n
e
h
g
i
h
g

^{1}H NMR (400 MHz) and ^{13}C NMR (100 MHz) spectra of **3b** ($CDCl_3$)

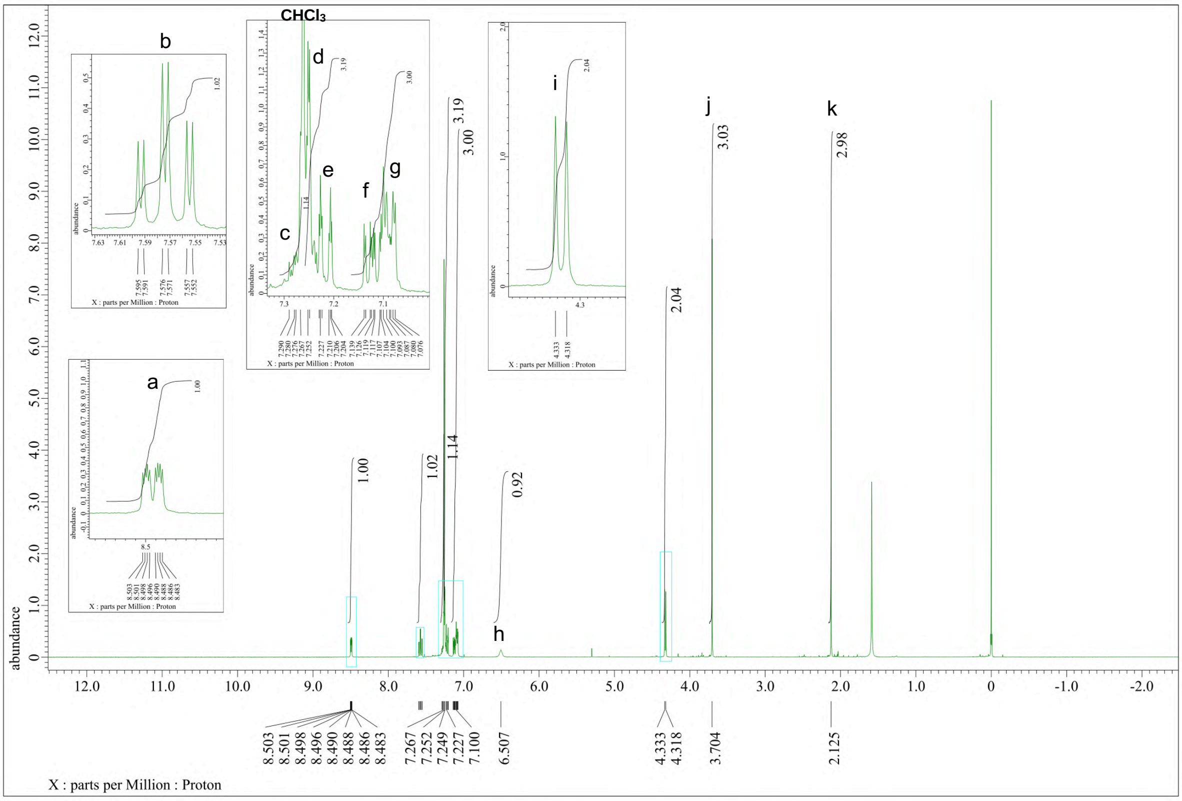

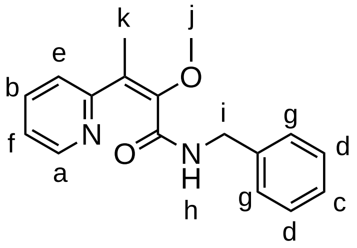

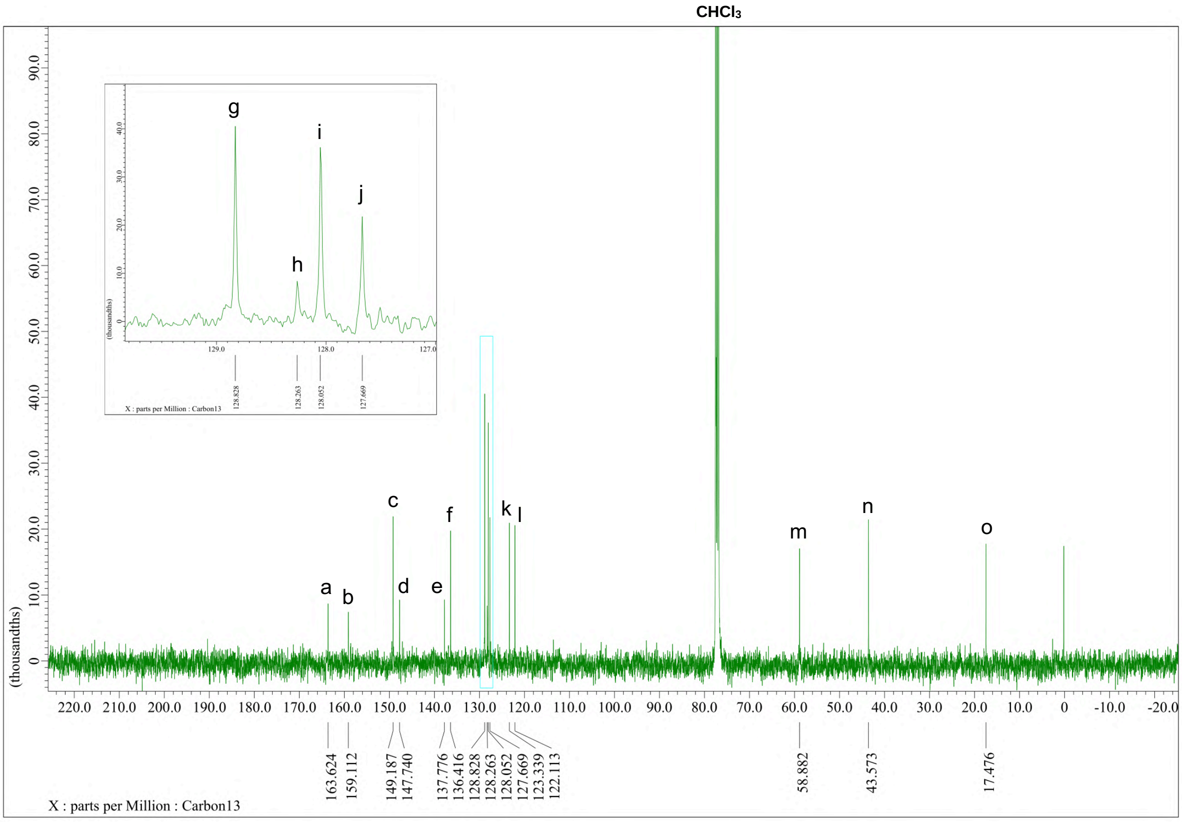

CHCl3
a
b
c
d
e
f
k
l
m
n
o
g
h
i
j
163.624
159.112
149.187
147.740
137.776
136.416
128.828
128.263
128.052
127.669
123.339
122.113
58.882
43.573
17.476
X : parts per Million : Carbon13
(thousandths)

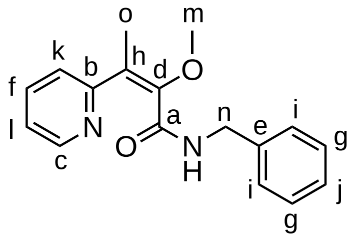

^{1}H NMR (400 MHz) and ^{13}C NMR (100 MHz) spectra of **3c** ($CDCl_3$)

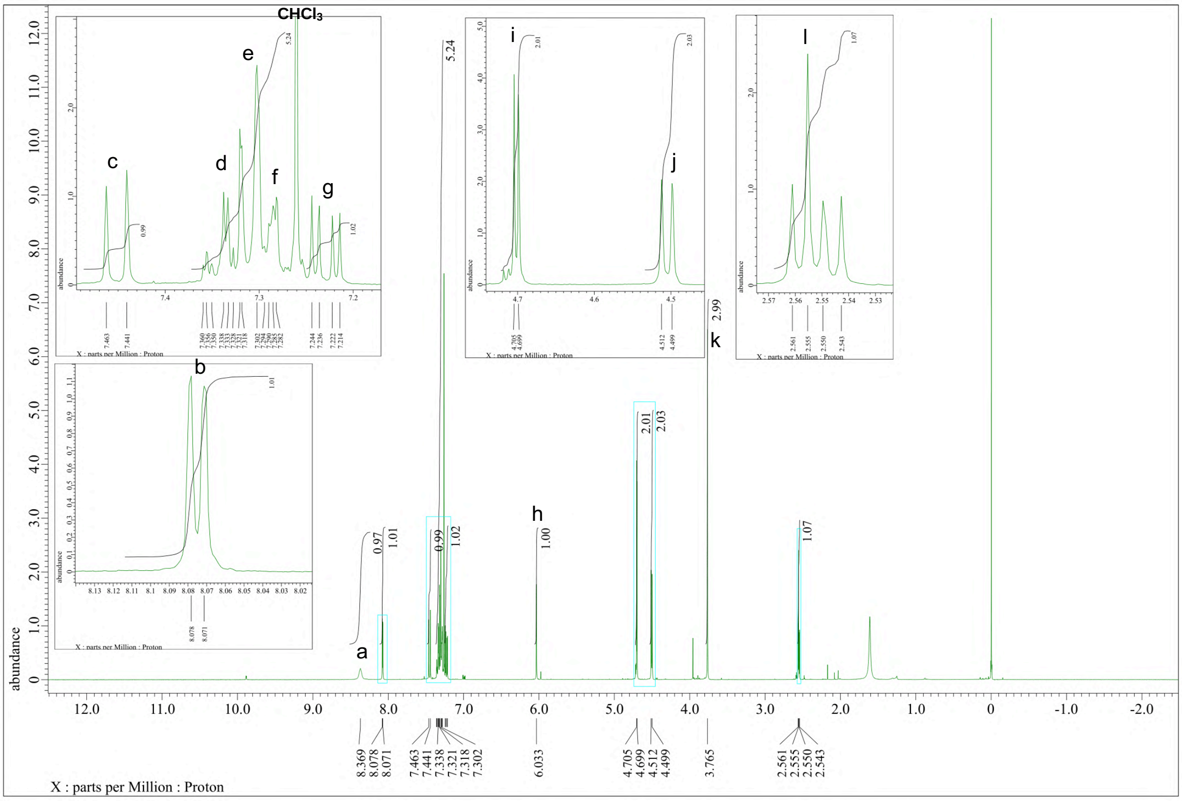

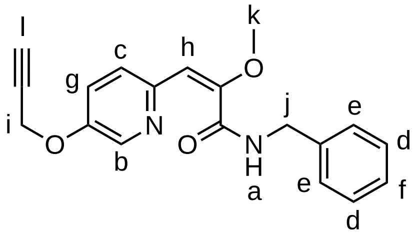

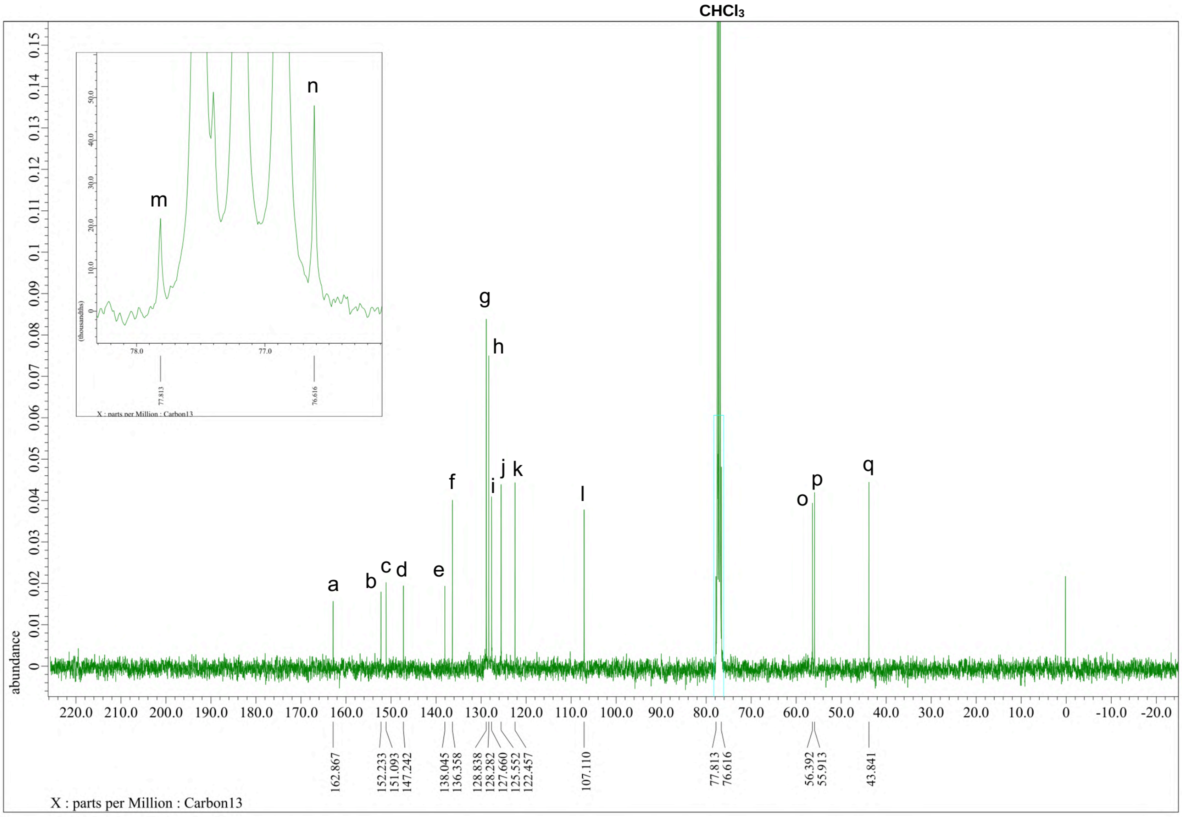
CHCl3
abundance
X : parts per Million : Carbon13
162.867
152.233
151.093
147.242
138.045
136.358
128.838
128.282
127.660
125.552
122.457
107.110
77.813
76.616
56.392
55.913
43.841
a
b
c
d
e
f
g
h
i
j
k
l
m
n
o
p
q

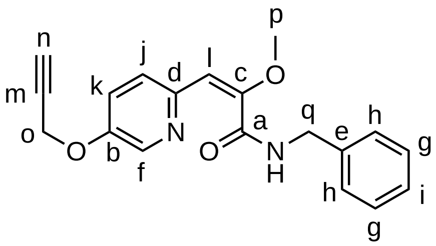
N
O
H

^{1}H NMR (400 MHz) and ^{13}C NMR (100 MHz) spectra of **3d** ($CDCl_3$)

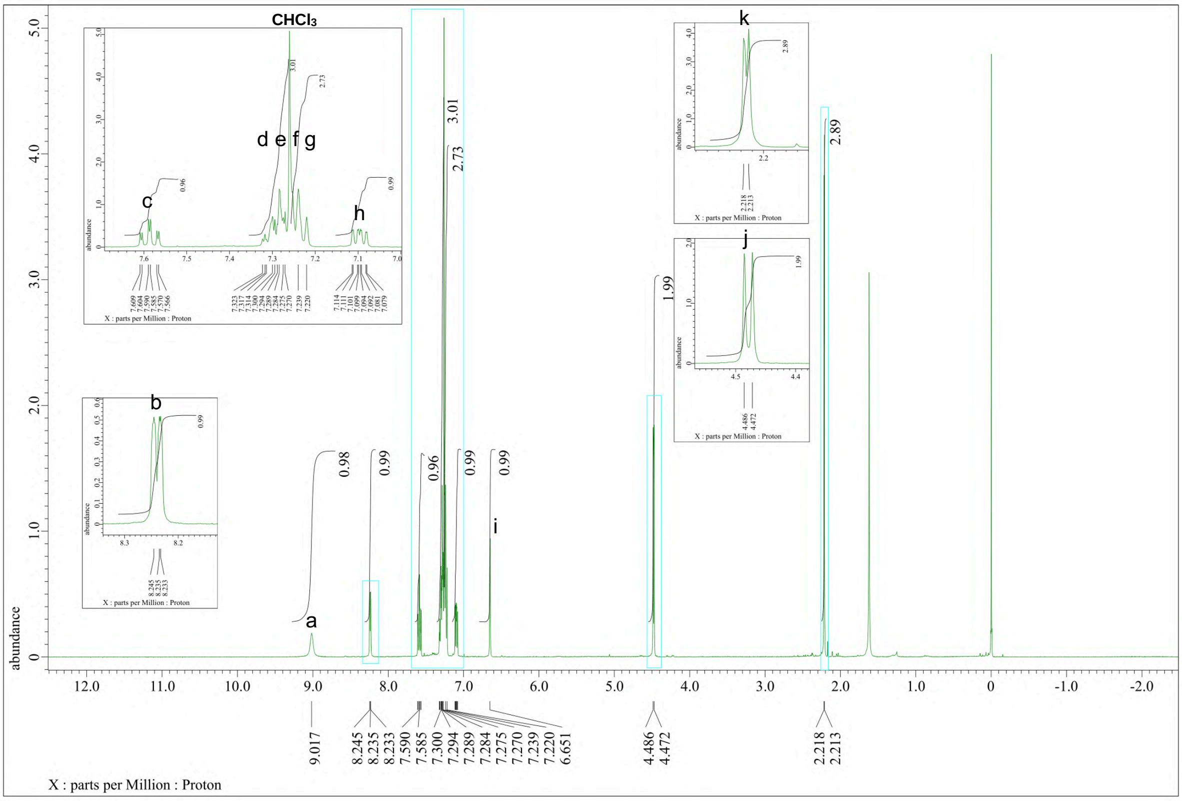

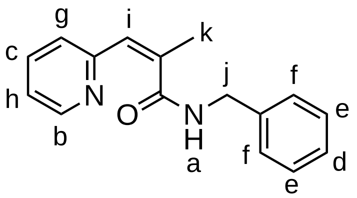

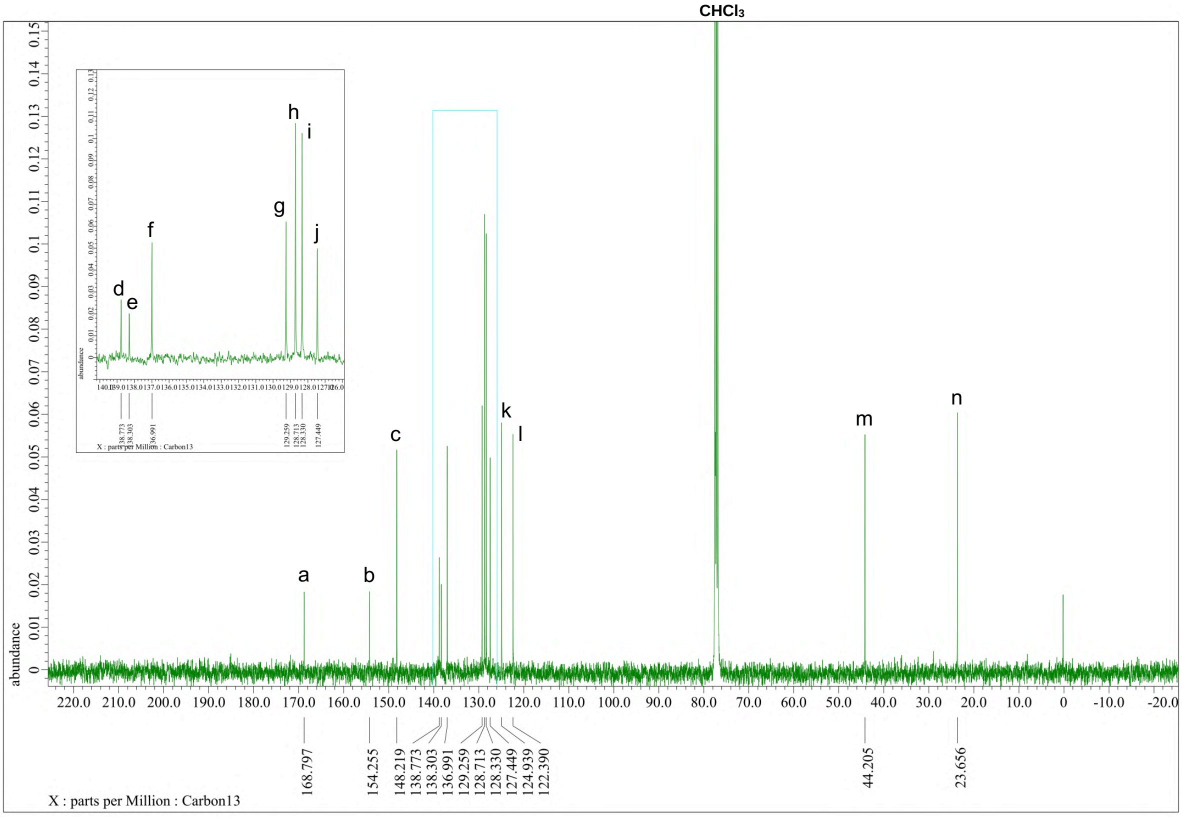
CHCl3
abundance
X : parts per Million : Carbon13
a
b
c
k
l
m
n
d
e
f
g
h
i
j
168.797
154.255
148.219
138.773
138.303
136.991
129.259
128.713
128.330
127.449
124.939
122.390
44.205
23.656

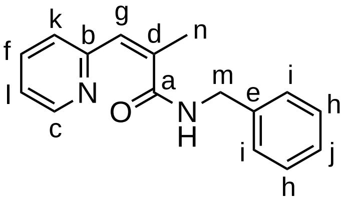
k
g
b
d
n
f
l
N
c
a
O
N
H
m
e
i
h
i
j
h

^{1}H NMR (400 MHz) and ^{13}C NMR (100 MHz) spectra of **3e** ($CDCl_3$)

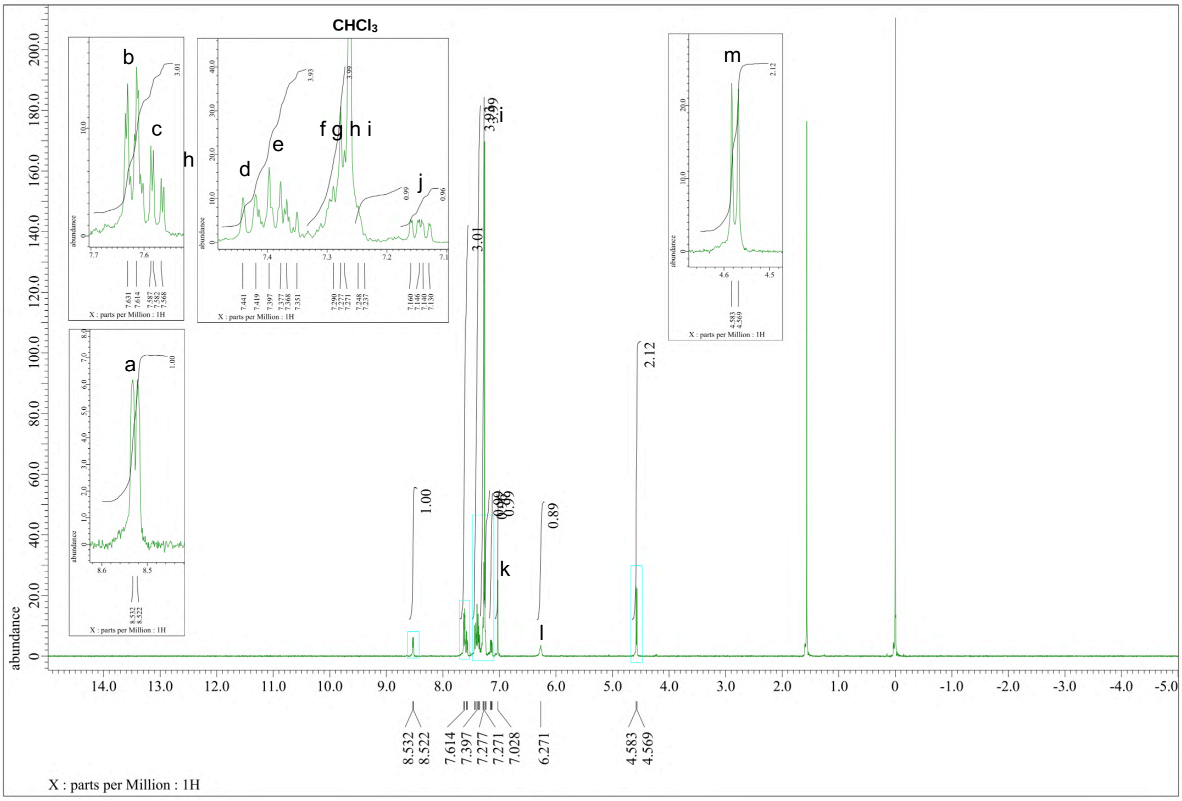

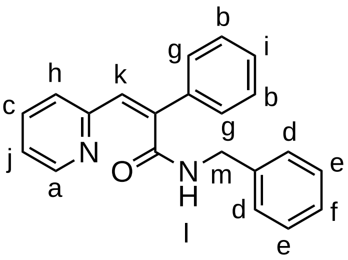

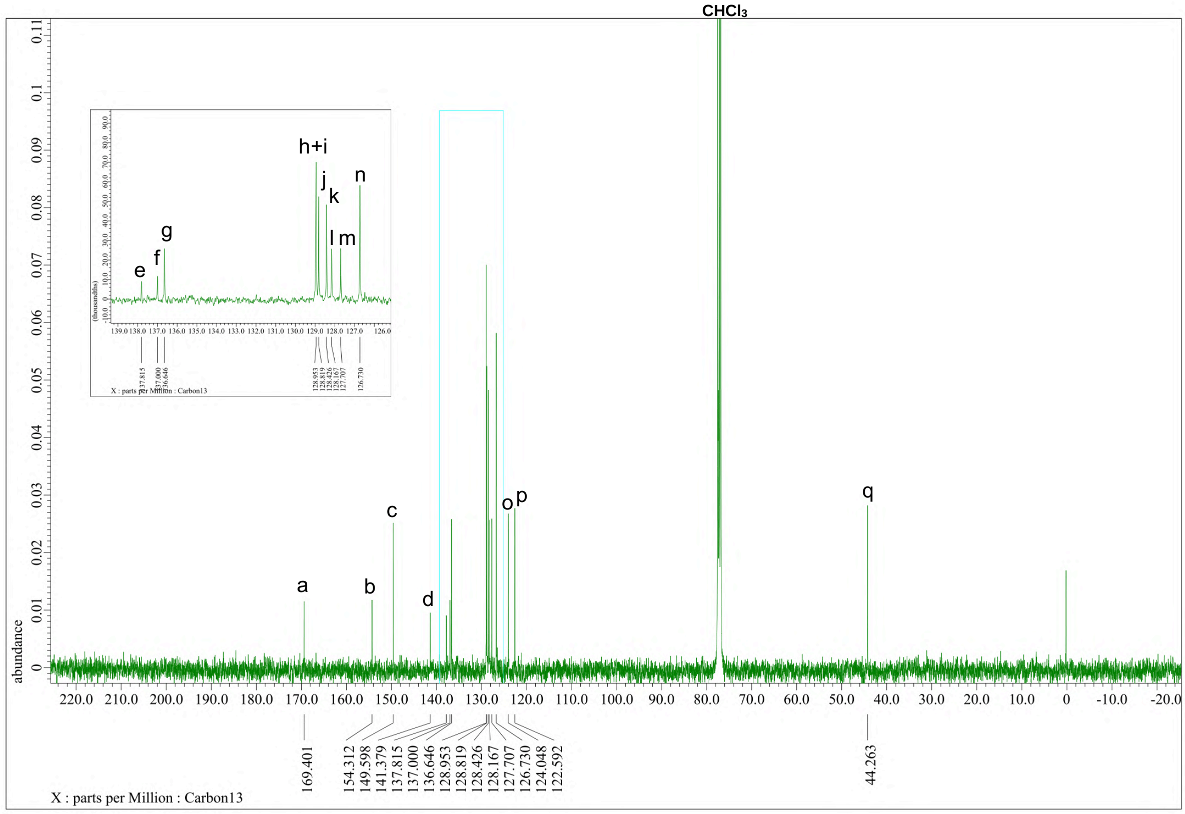
CHCl3
abundance
X : parts per Million : Carbon13
a
b
c
d
o p
q
h+i
j
k
l m
n
e
f
g
169.401
154.312
149.598
141.379
137.815
137.000
136.646
128.953
128.819
128.426
128.167
127.707
126.730
124.048
122.592
44.263

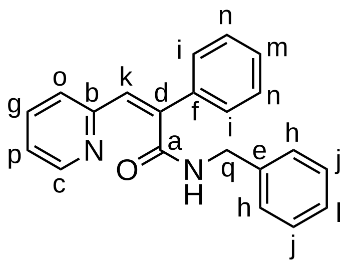
n
i
m
o
k
d
b
g
f
n
i
a
h
e
j
p
N
O
N
H
q
c
h
l
j

^{1}H NMR (400 MHz) and ^{13}C NMR (100 MHz) spectra of **3f** (DMSO-d_6)

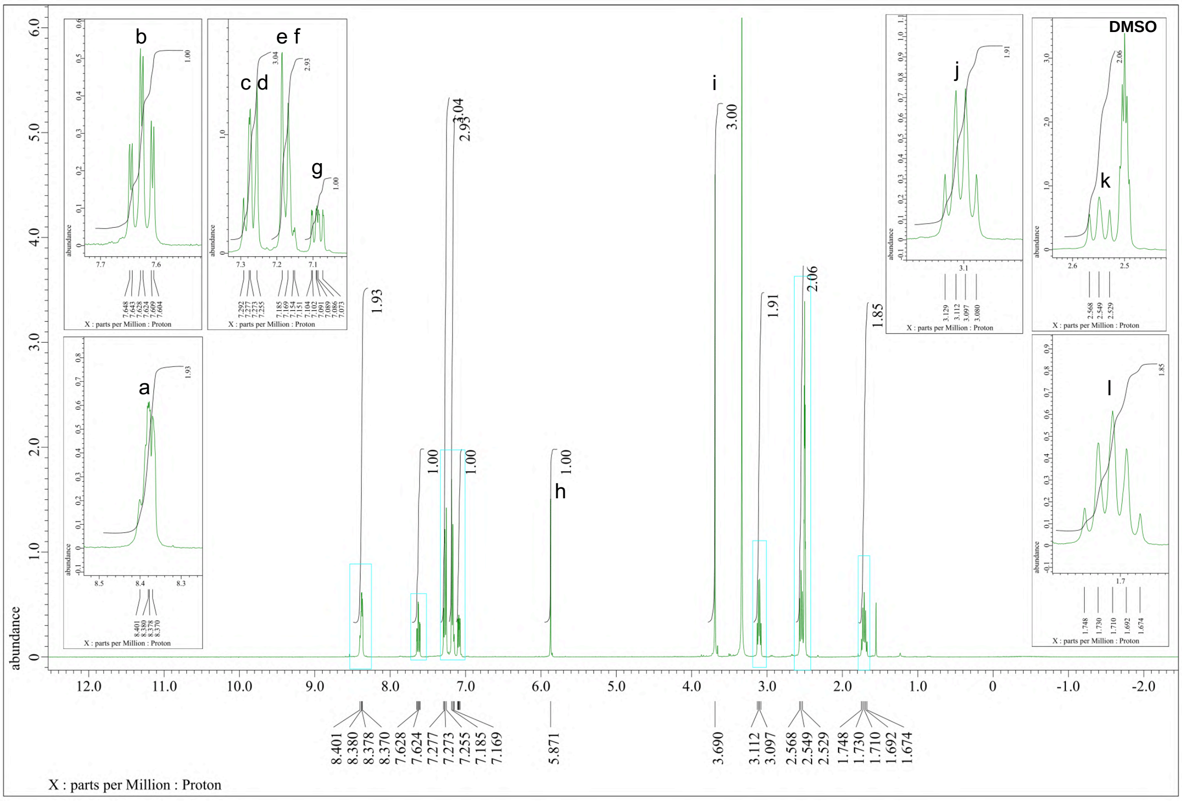

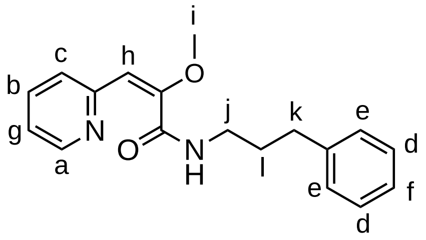

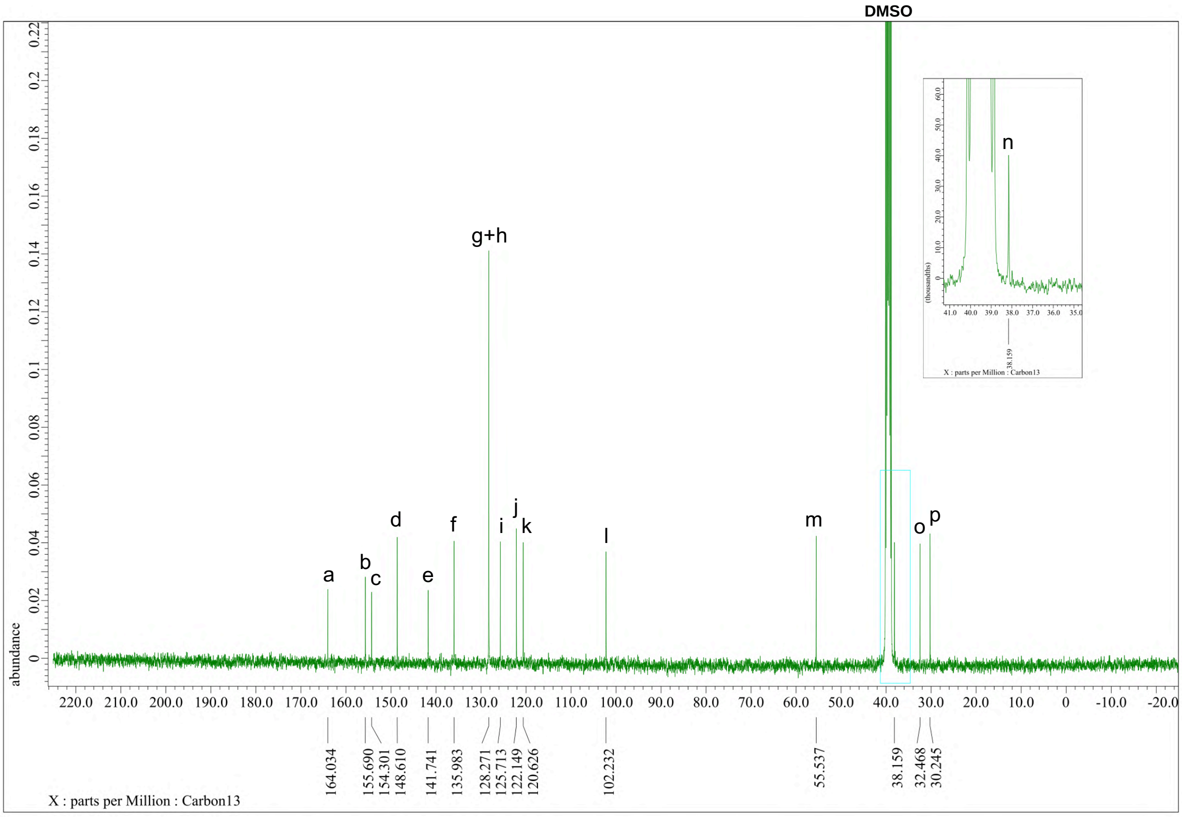
DMSO
abundance
X : parts per Million : Carbon13
a
b
c
d
e
f
g+h
i
j
k
l
m
o
p
n
164.034
155.690
154.301
148.610
141.741
135.983
128.271
125.713
122.149
120.626
102.232
55.537
38.159
32.468
30.245

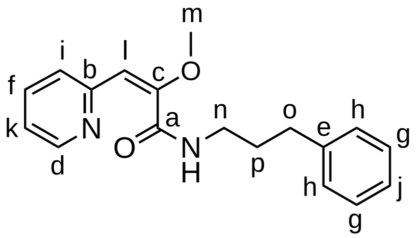
m
l
i
b
c
O
f
N
k
d
O
a
N
H
n
p
o
e
h
g
j
h
g

^{1}H NMR (400 MHz) and ^{13}C NMR (100 MHz) spectra of **3g** ($CDCl_3$)

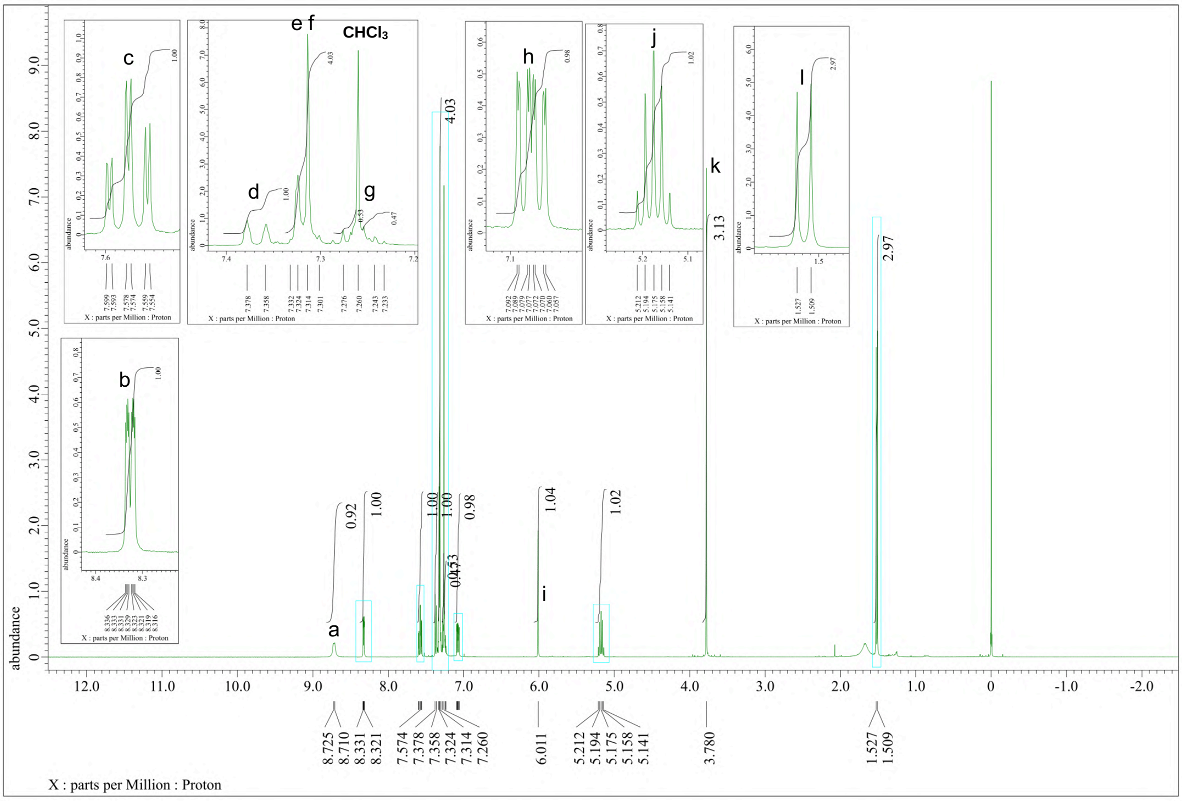

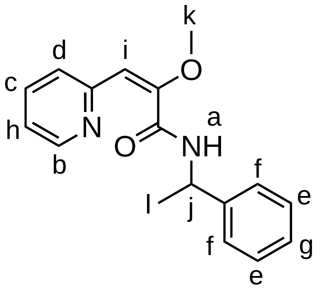

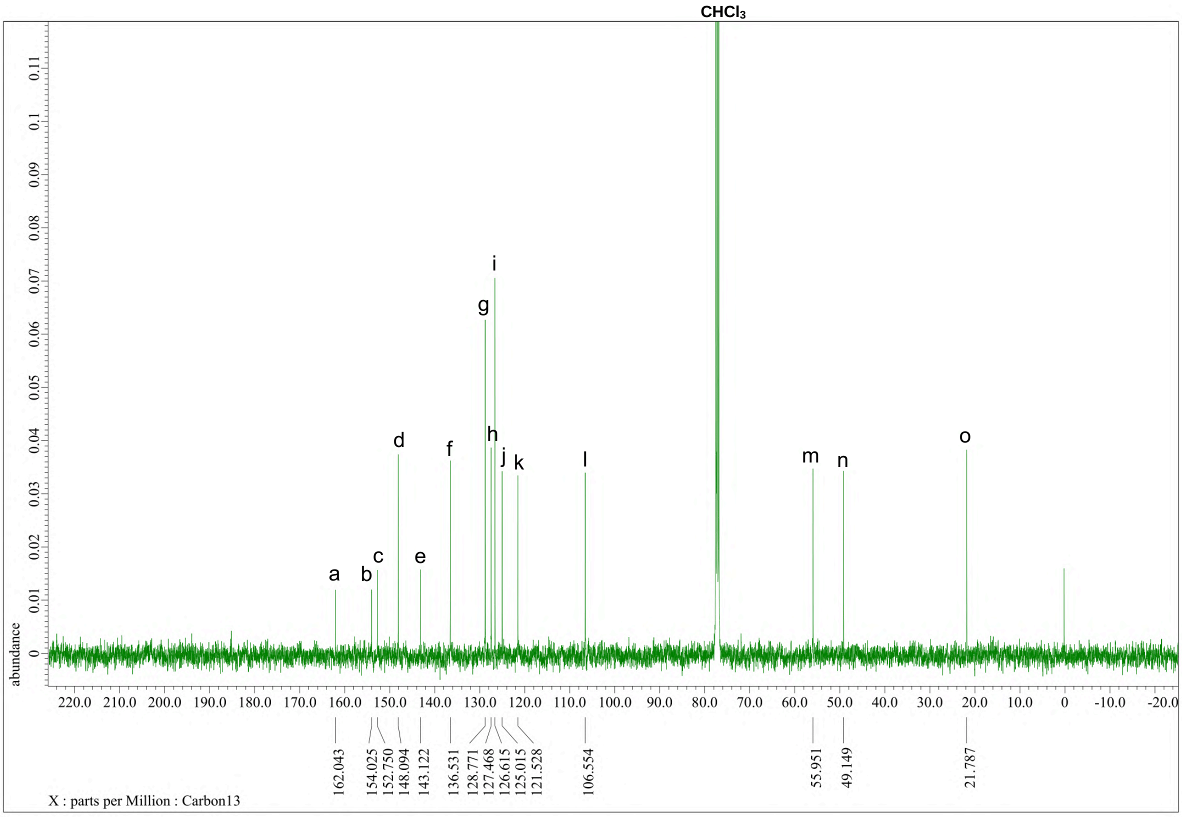
CHCl3
abundance
0.11
0.1
0.09
0.08
0.07
0.06
0.05
0.04
0.03
0.02
0.01
0
a
b
c
d
e
f
g
h
i
j
k
l
m
n
o
220.0 210.0 200.0 190.0 180.0 170.0 160.0 150.0 140.0 130.0 120.0 110.0 100.0 90.0 80.0 70.0 60.0 50.0 40.0 30.0 20.0 10.0 0 -10.0 -20.0
162.043
154.025
152.750
148.094
143.122
136.531
128.771
127.468
126.615
125.015
121.528
106.554
55.951
49.149
21.787
X : parts per Million : Carbon13

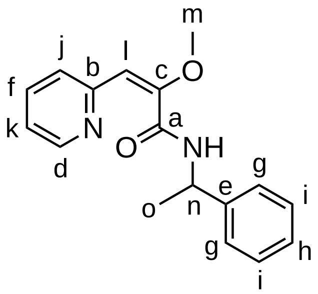
m
j
b
l
c
O
f
k
N
a
O
NH
d
g
e
i
o
n
g
h
i

^{1}H NMR (400 MHz) and ^{13}C NMR (100 MHz) spectra of **3h** ($CDCl_3$)

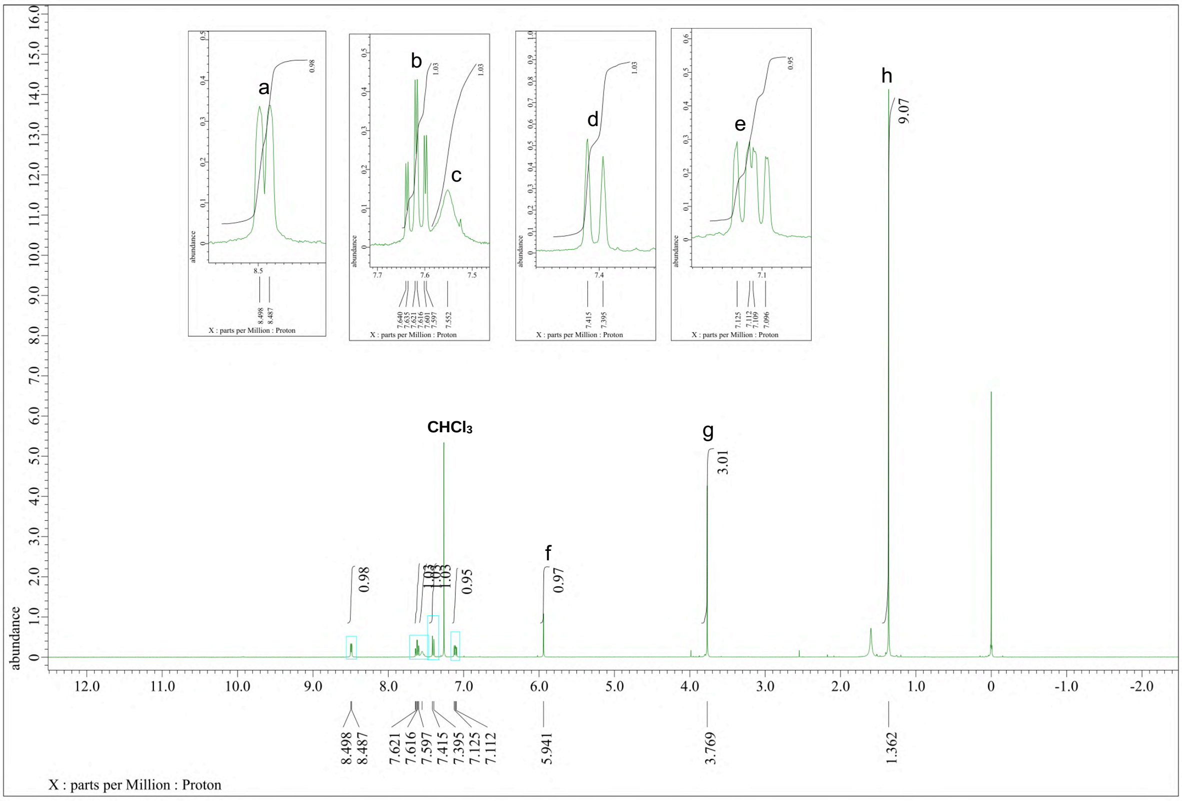

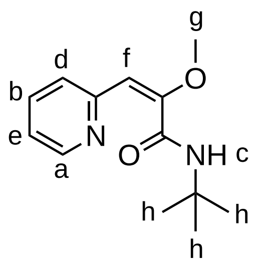

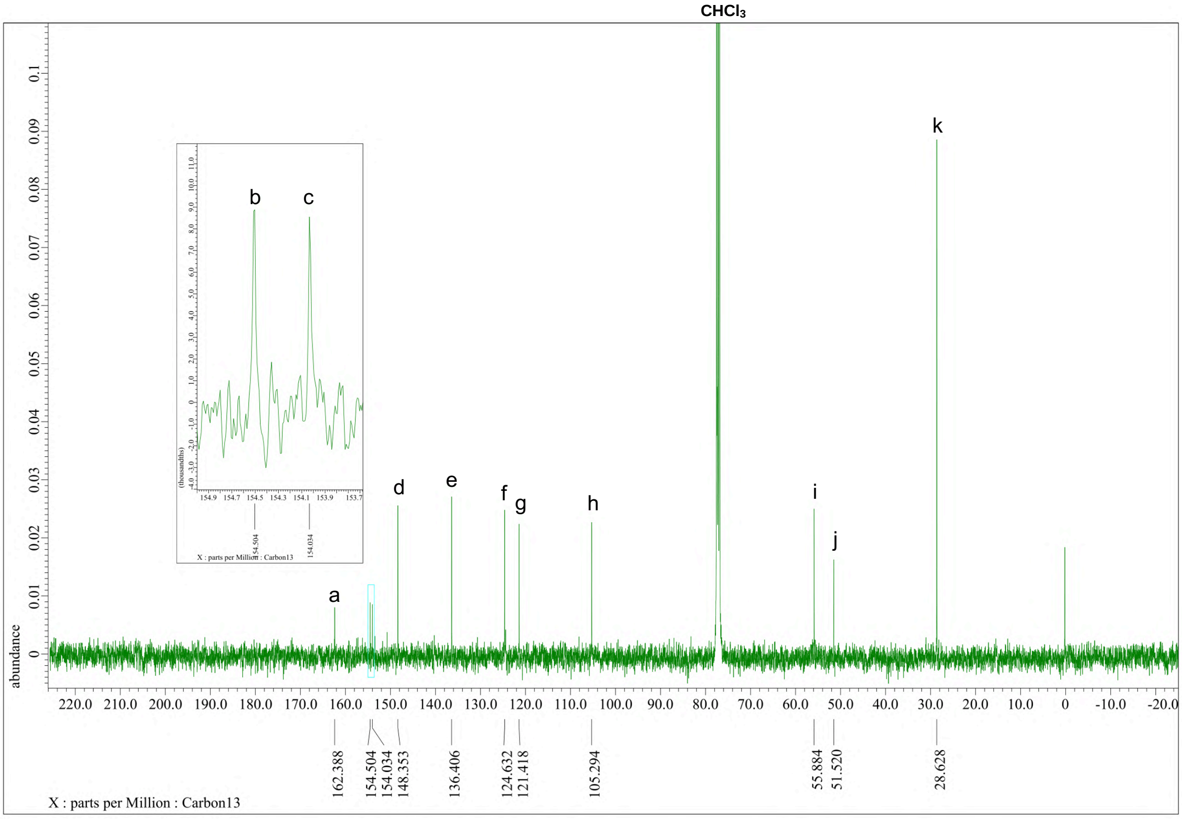
CHCl3
abundance
b
c
X : parts per Million : Carbon13
a
d
e
f
g
h
i
j
k
162.388
154.504
154.034
148.353
136.406
124.632
121.418
105.294
55.884
51.520
28.628
X : parts per Million : Carbon13

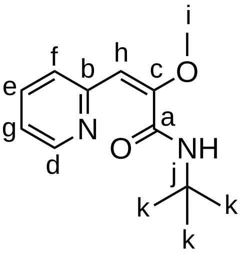
i
O
h
c
f
b
e
g
N
d
a
O
NH
j
k
k
k

^{1}H NMR (400 MHz) and ^{13}C NMR (100 MHz) spectra of **3i** ($CDCl_3$)

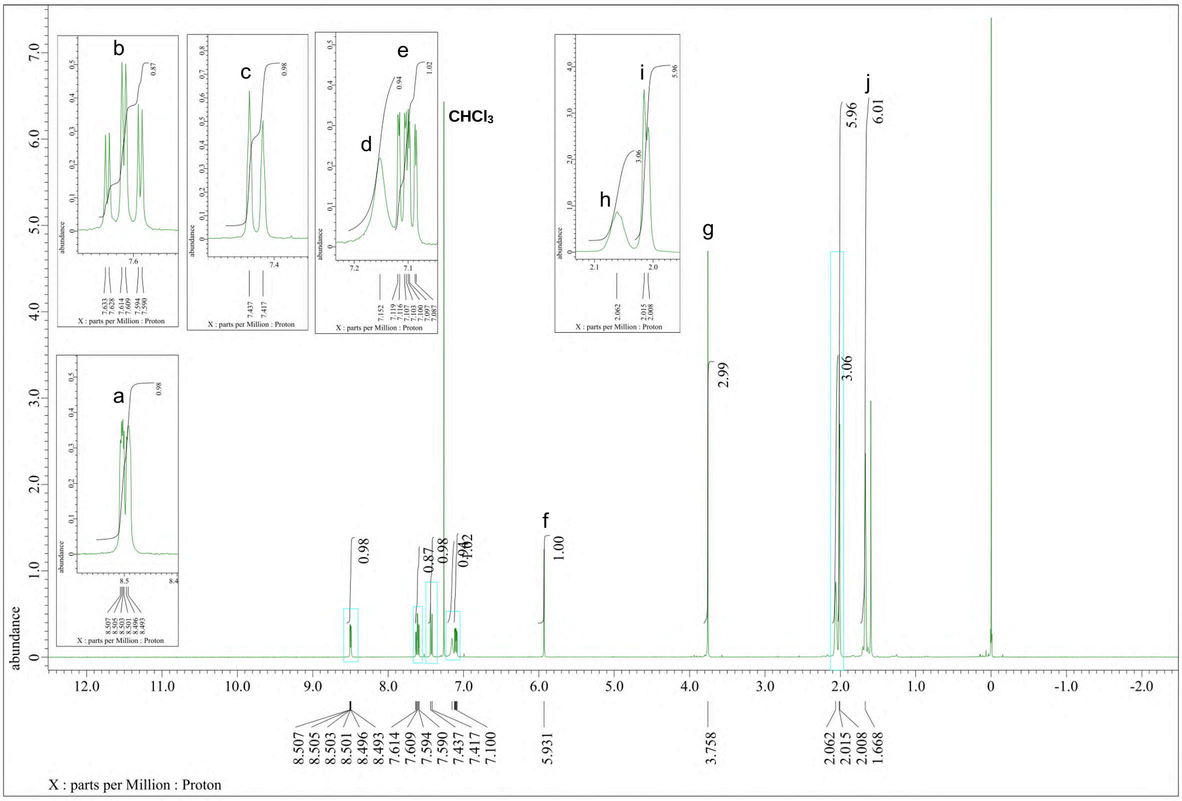

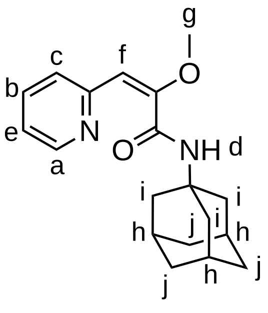

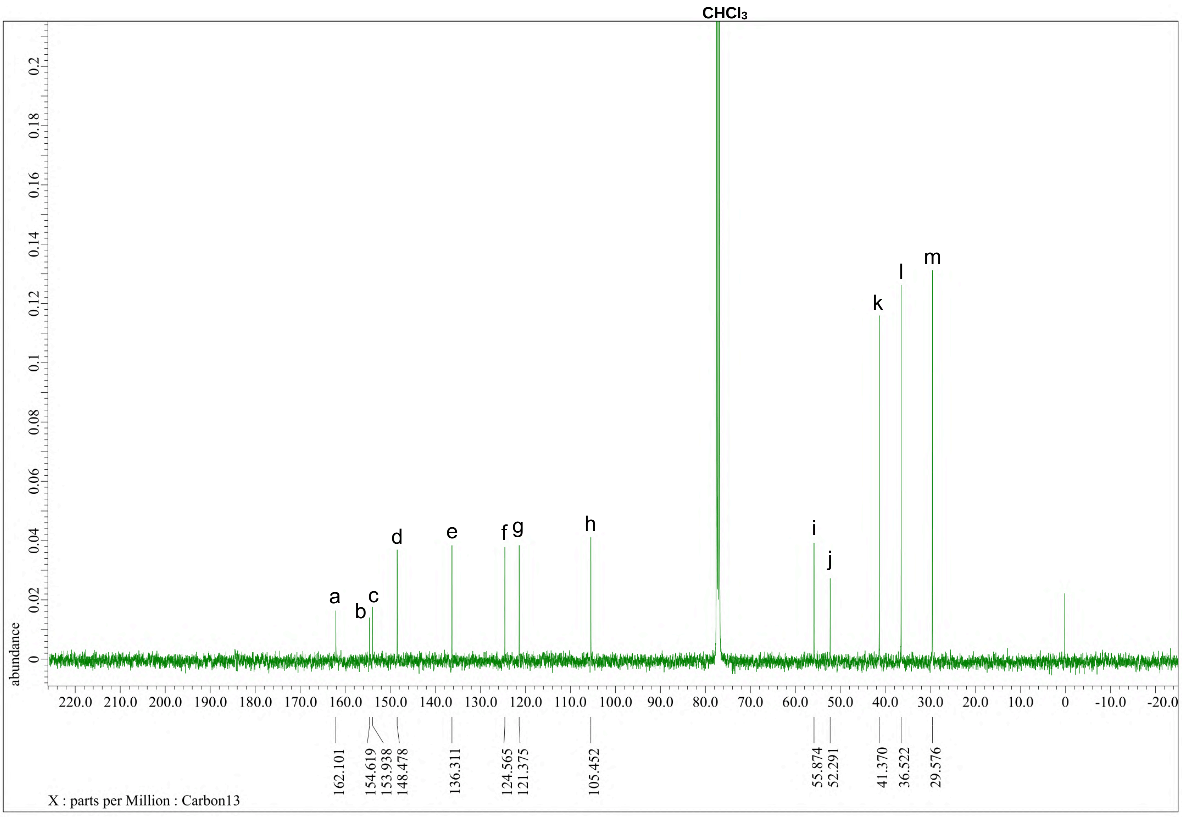
CHCl3
abundance
X : parts per Million : Carbon13
162.101
154.619
153.938
148.478
136.311
124.565
121.375
105.452
55.874
52.291
41.370
36.522
29.576

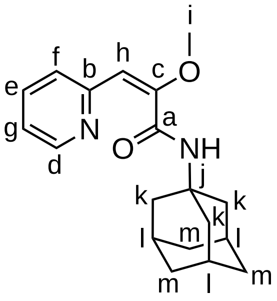

^{1}H NMR (400 MHz) and ^{13}C NMR (100 MHz) spectra of **3j** ($CDCl_3$)

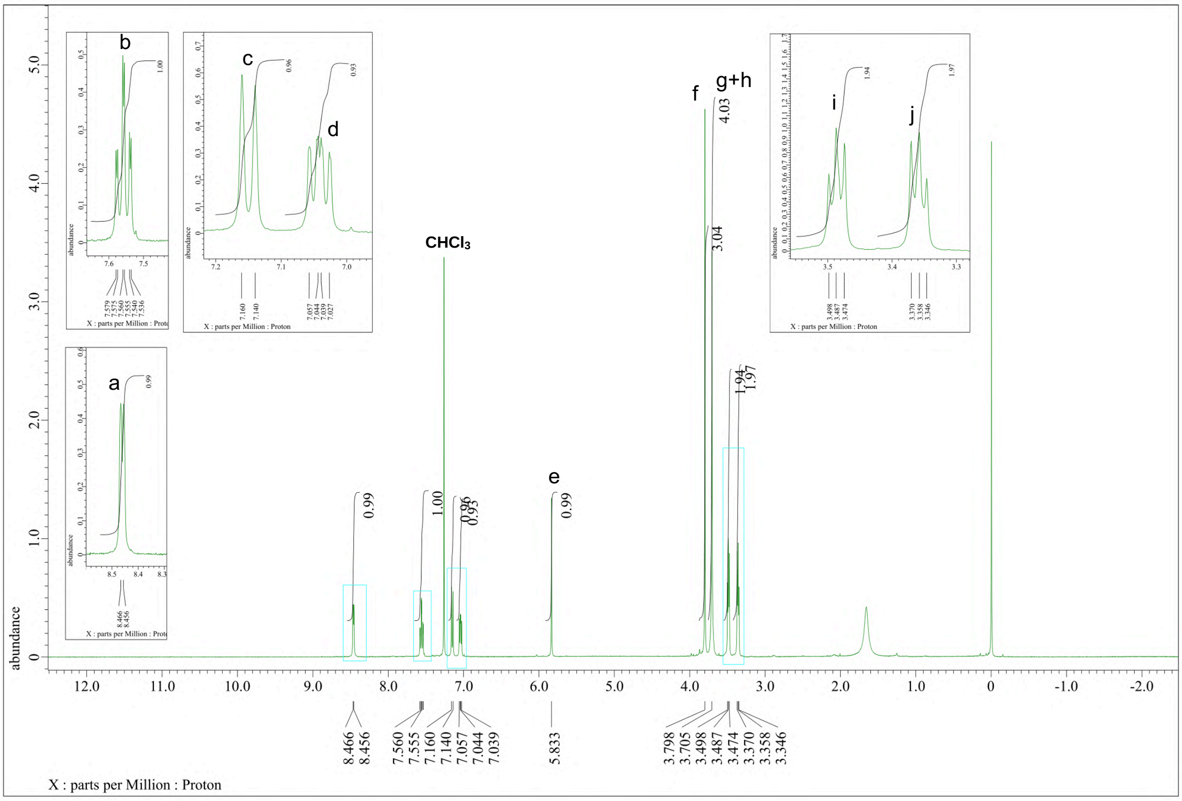

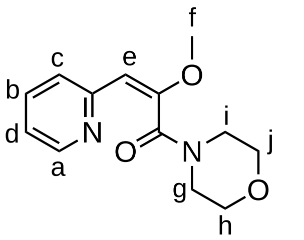

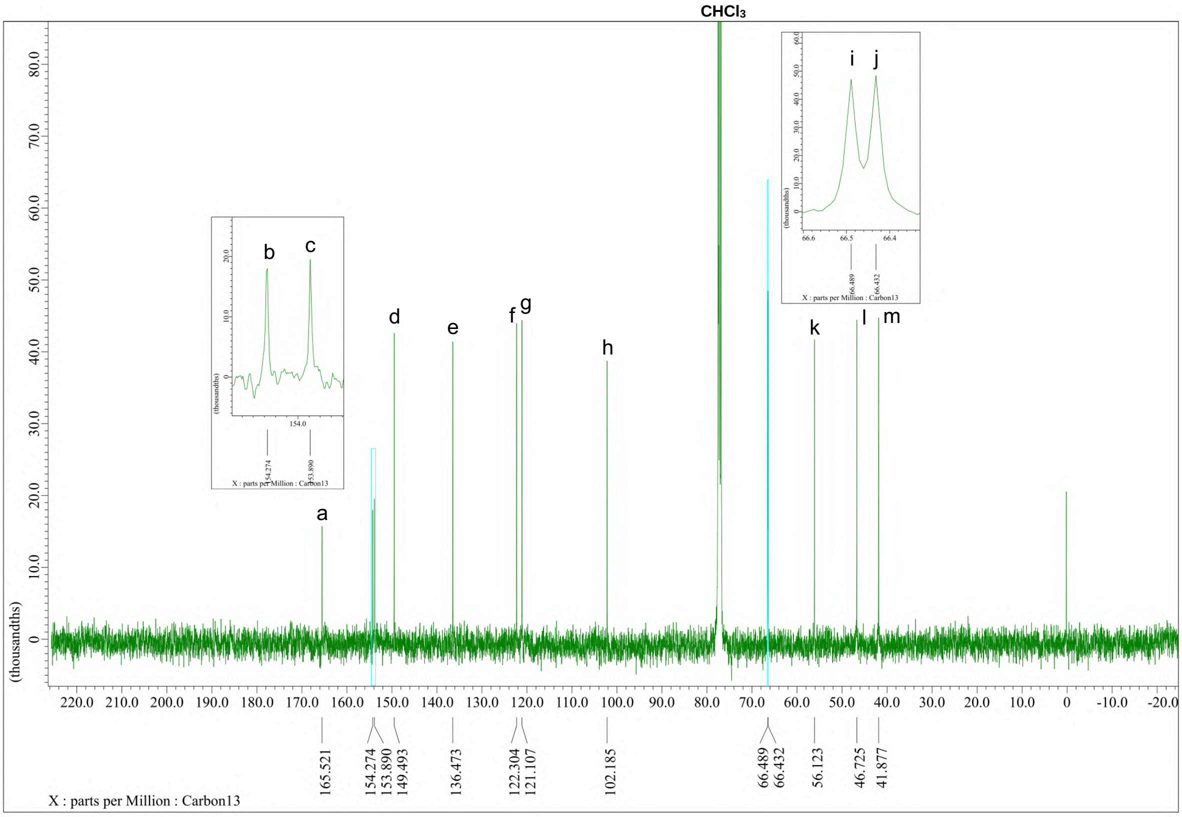
CHCl3
a
b
c
d
e
f
g
h
i
j
k
l
m
(thousandths)
X : parts per Million : Carbon13
165.521
154.274
153.890
149.493
136.473
122.304
121.107
102.185
66.489
66.432
56.123
46.725
41.877

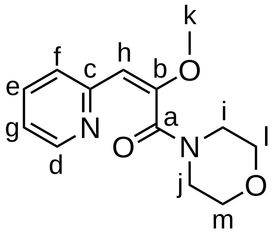
k
f
h
c
b
O
e
N
a
i
g
O
N
l
d
j
O
m

^{1}H NMR (400 MHz) and ^{13}C NMR (100 MHz) spectra of **3k** ($CDCl_3$)

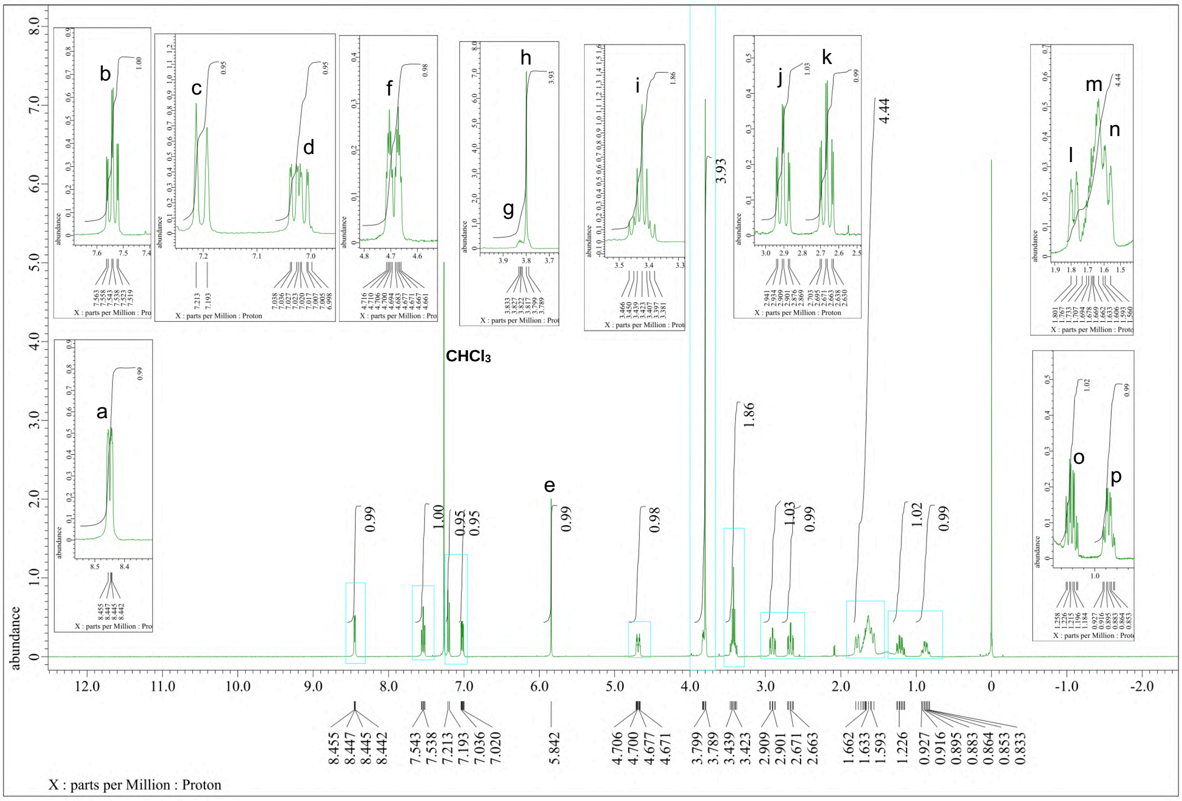

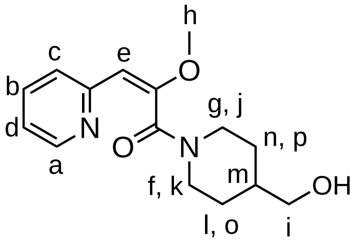

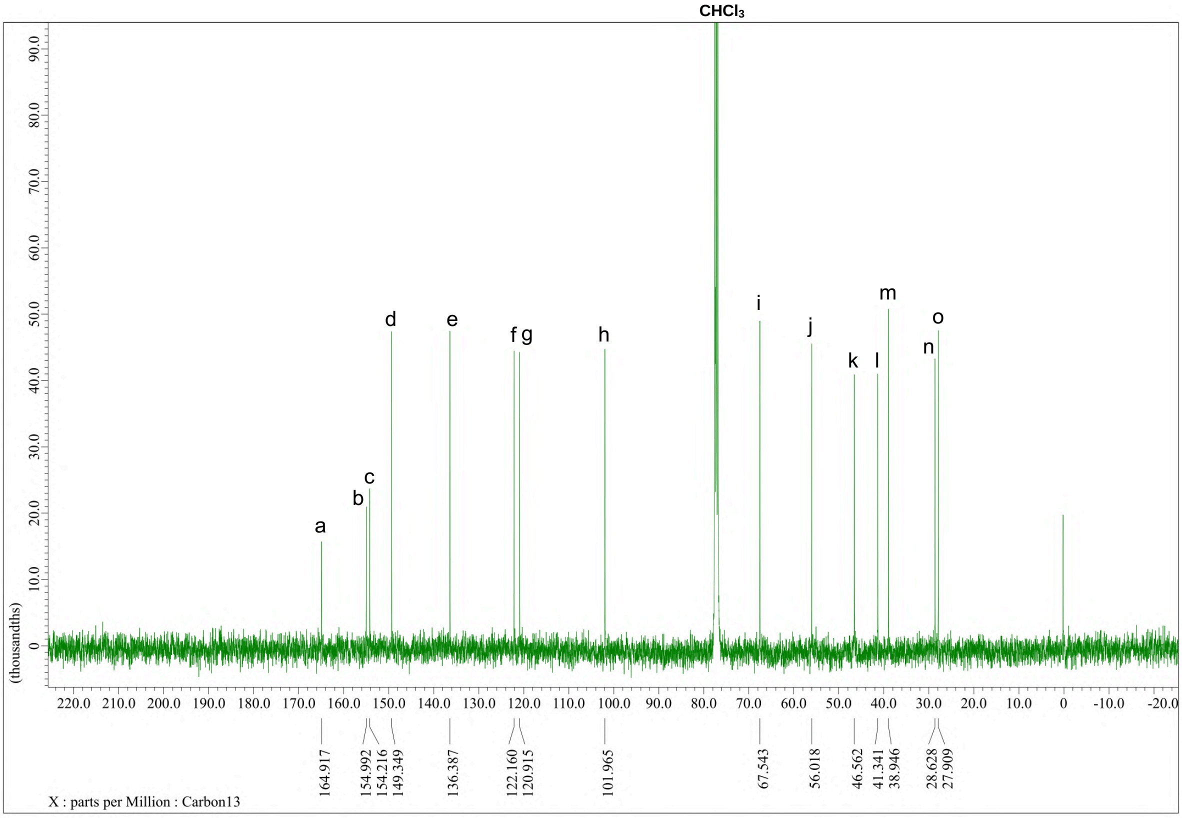
CHCl3
a
b
c
d
e
f g
h
i
j
k
l
m
n
o
(thousandths)
164.917
154.992
154.216
149.349
136.387
122.160
120.915
101.965
67.543
56.018
46.562
41.341
38.946
28.628
27.909
X : parts per Million : Carbon13

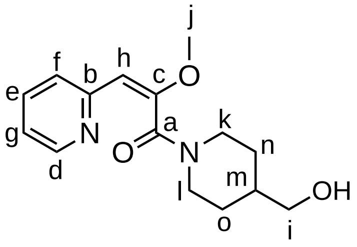
j
f
b
h
c
e
g
d
N
O
a
O
N
k
n
m
l
o
OH
i

^{1}H NMR (400 MHz) and ^{13}C NMR (100 MHz) spectra of **3l** ($CDCl_3$)

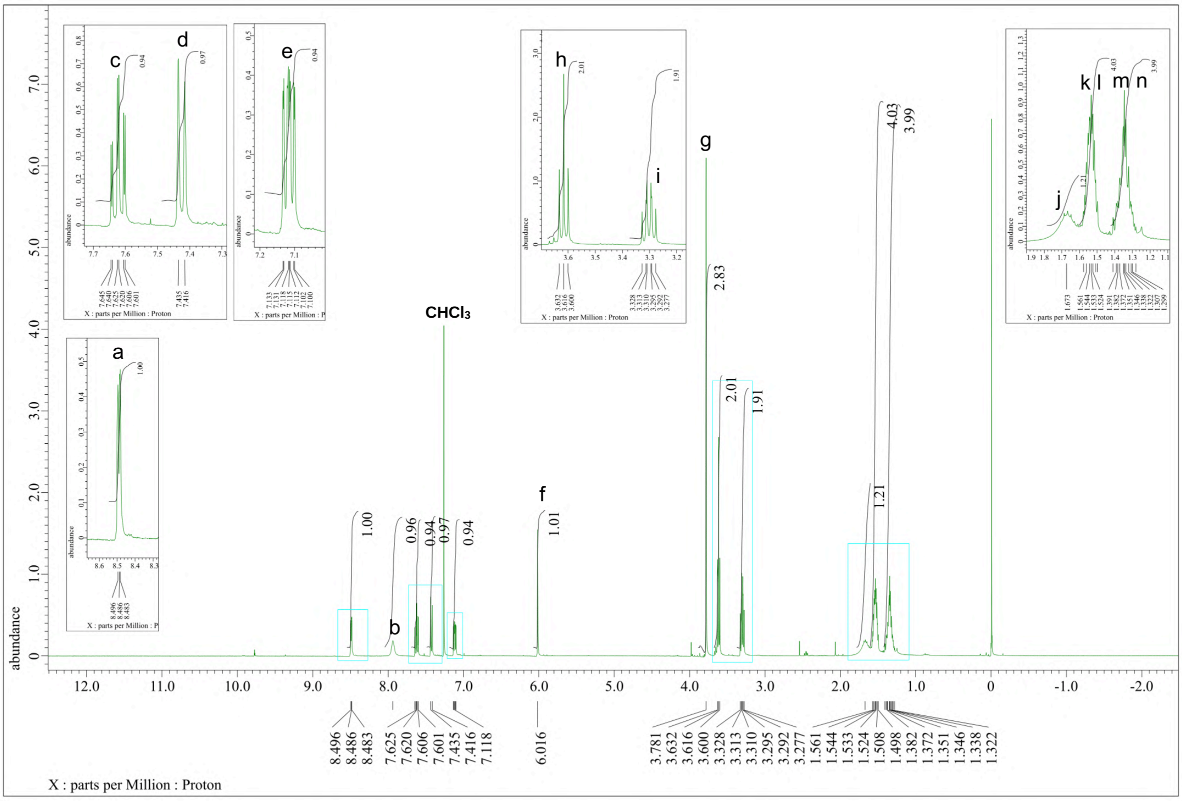

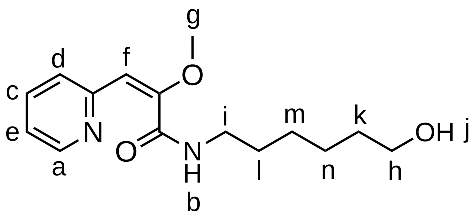

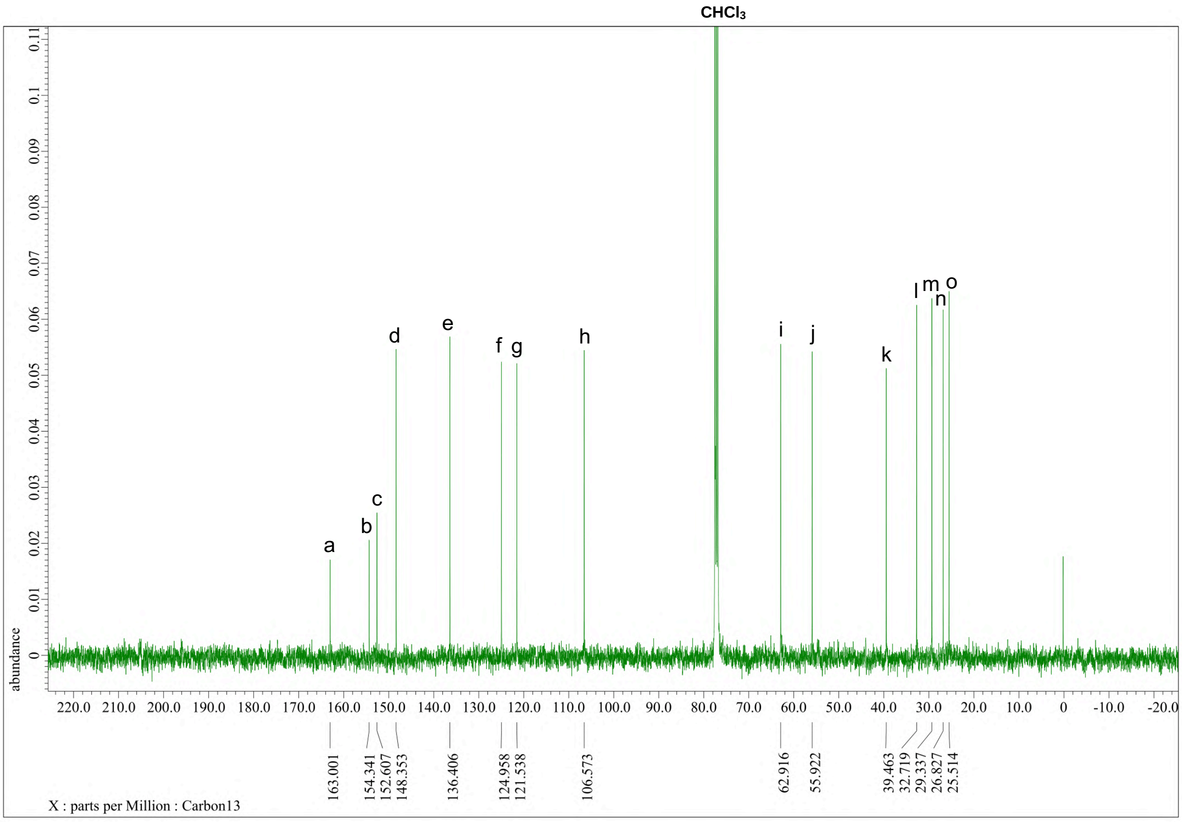
CHCl3
abundance
X : parts per Million : Carbon13
a
b
c
d
e
f g
h
i
j
k
l m o
n
163.001
154.341
152.607
148.353
136.406
124.958
121.538
106.573
62.916
55.922
39.463
32.719
29.337
26.827
25.514

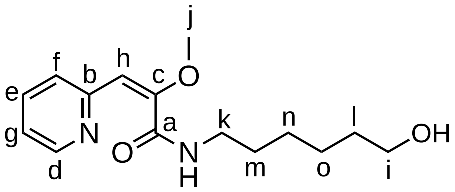
j
f
b
h
c
O
e
a
k
n
l
OH
g
N
O
N
H
d
m
o
i

^{1}H NMR (400 MHz) and ^{13}C NMR (100 MHz) spectra of **3m** ($CDCl_3$)

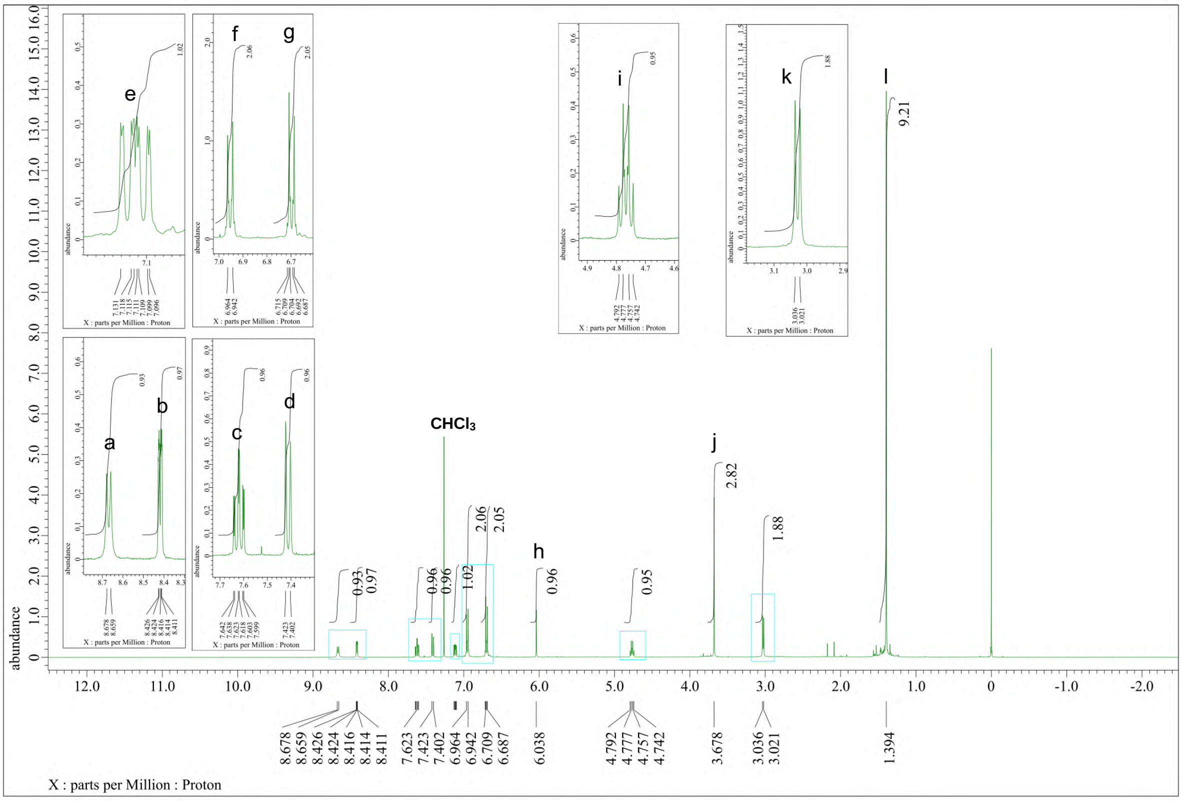

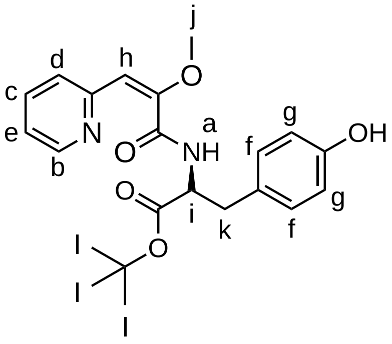

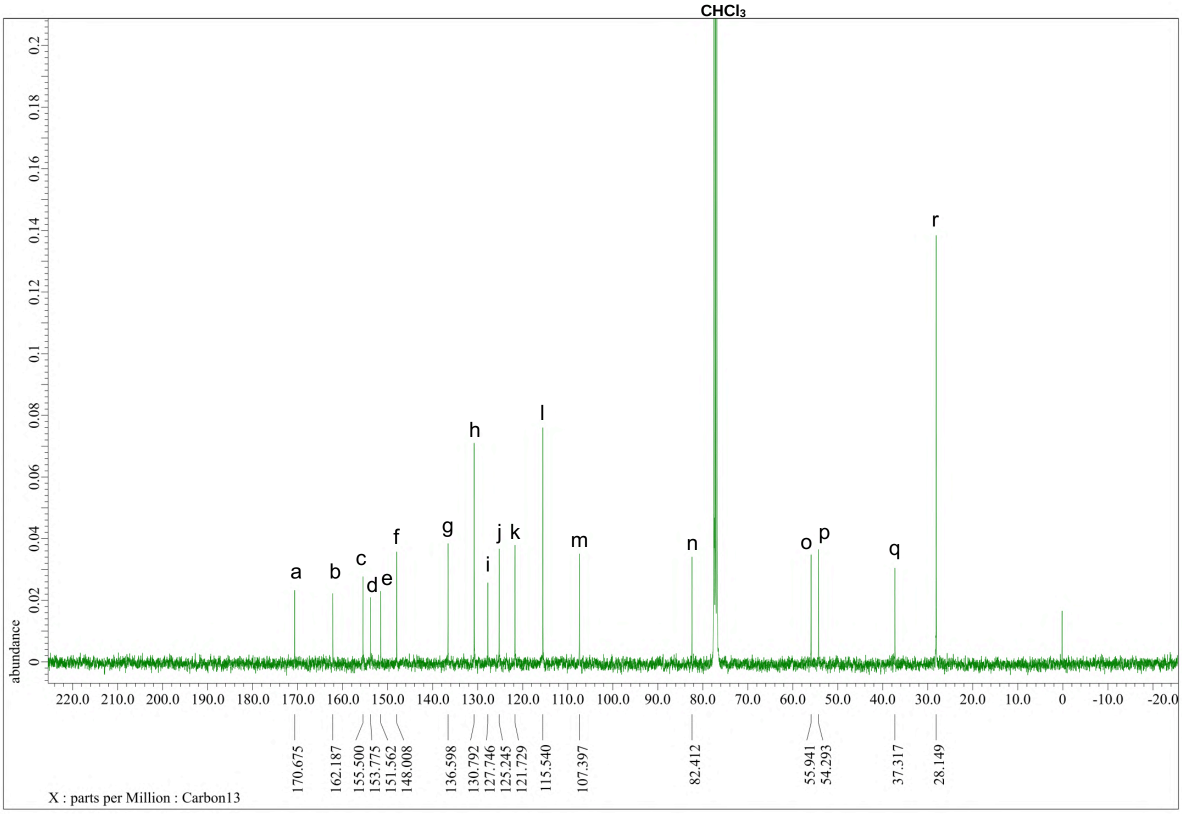
CHCl3
abundance
X : parts per Million : Carbon13
a
b
c
d
e
f
g
h
i
j
k
l
m
n
o
p
q
r
170.675
162.187
155.500
153.775
151.562
148.008
136.598
130.792
127.746
125.245
121.729
115.540
107.397
82.412
55.941
54.293
37.317
28.149

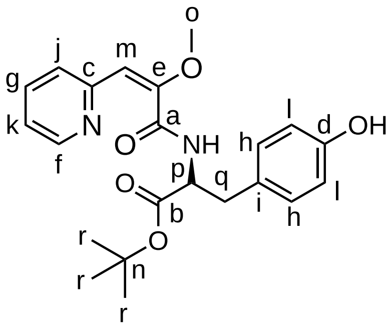

^{1}H NMR (400 MHz) and ^{13}C NMR (100 MHz) spectra of *rac*-**3m** ($CDCl_3$)

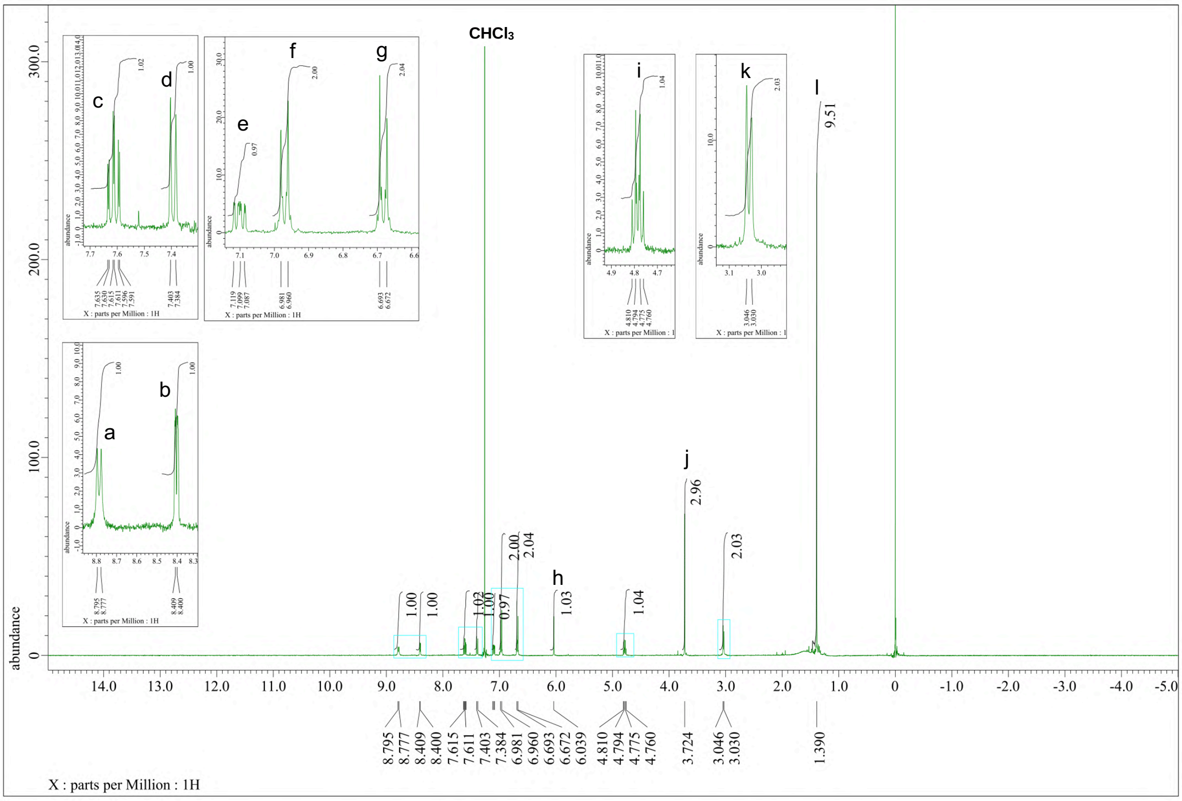

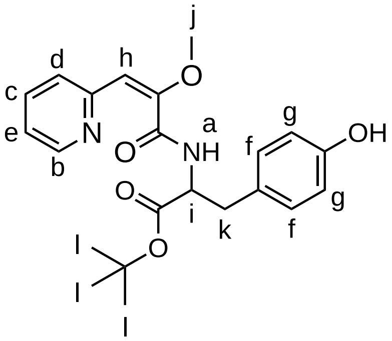

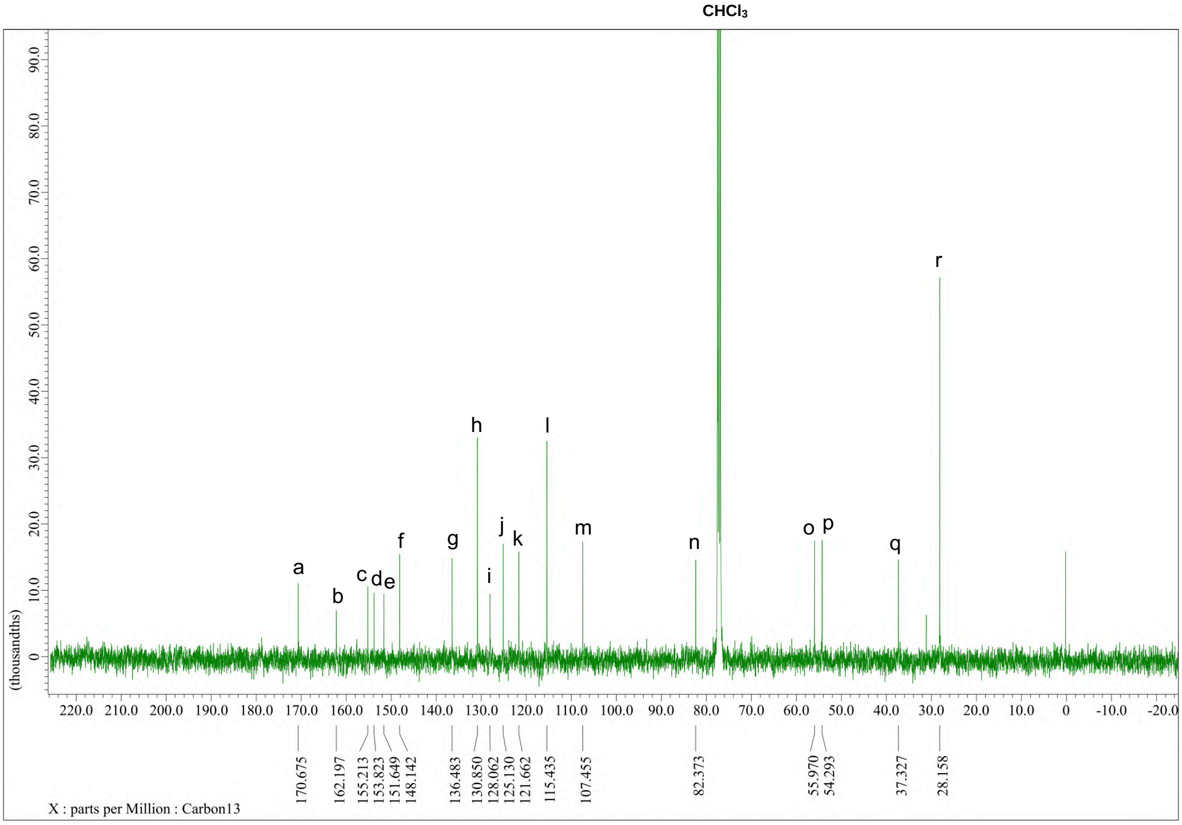

CHCl3
a
b
c
d
e
f
g
h
i
j
k
l
m
n
o
p
q
r
(thousandths)
X : parts per Million : Carbon13
170.675
162.197
155.213
153.823
151.649
148.142
136.483
130.850
128.062
125.130
121.662
115.435
107.455
82.373
55.970
54.293
37.327
28.158

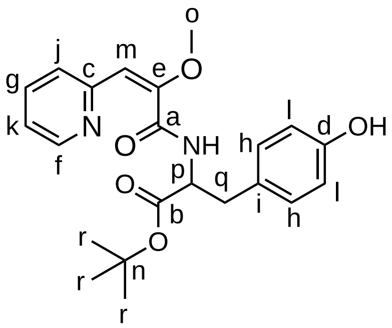

^{1}H NMR (400 MHz) and ^{13}C NMR (100 MHz) spectra of **3n** ($CDCl_3$)

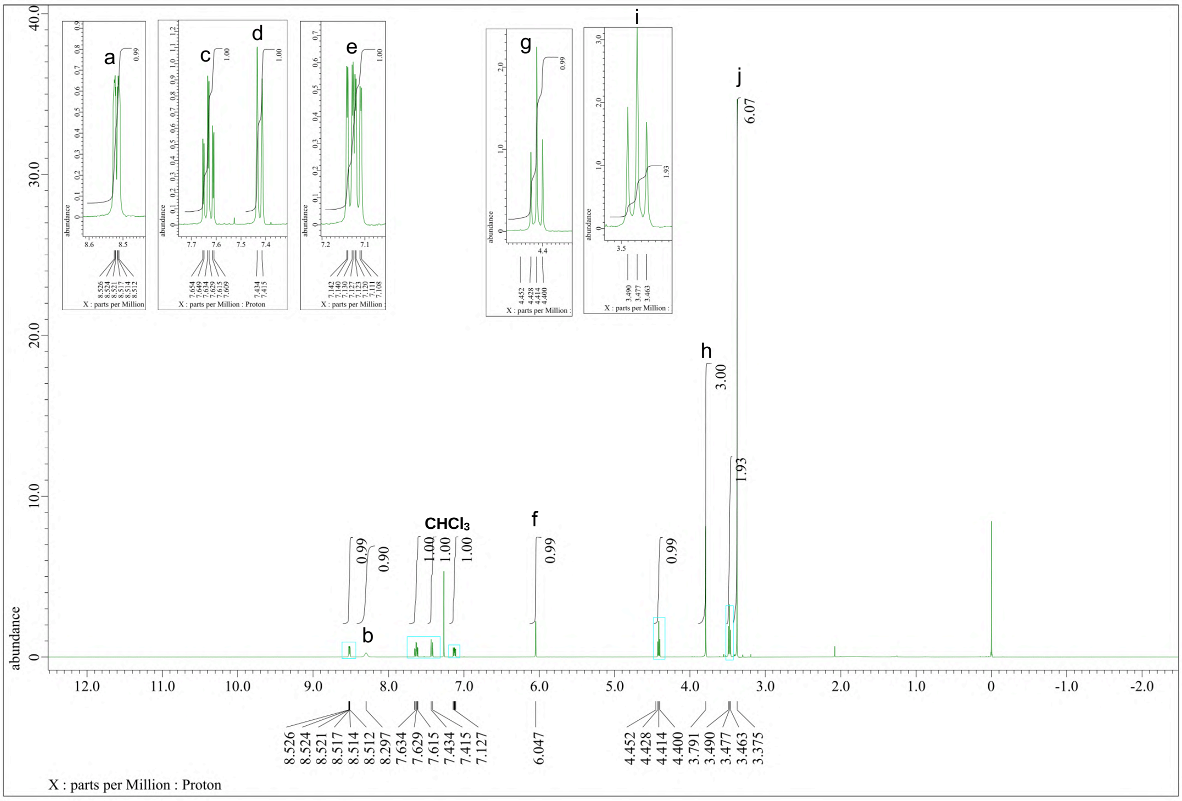

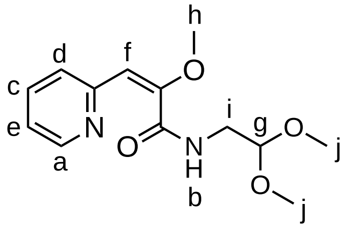

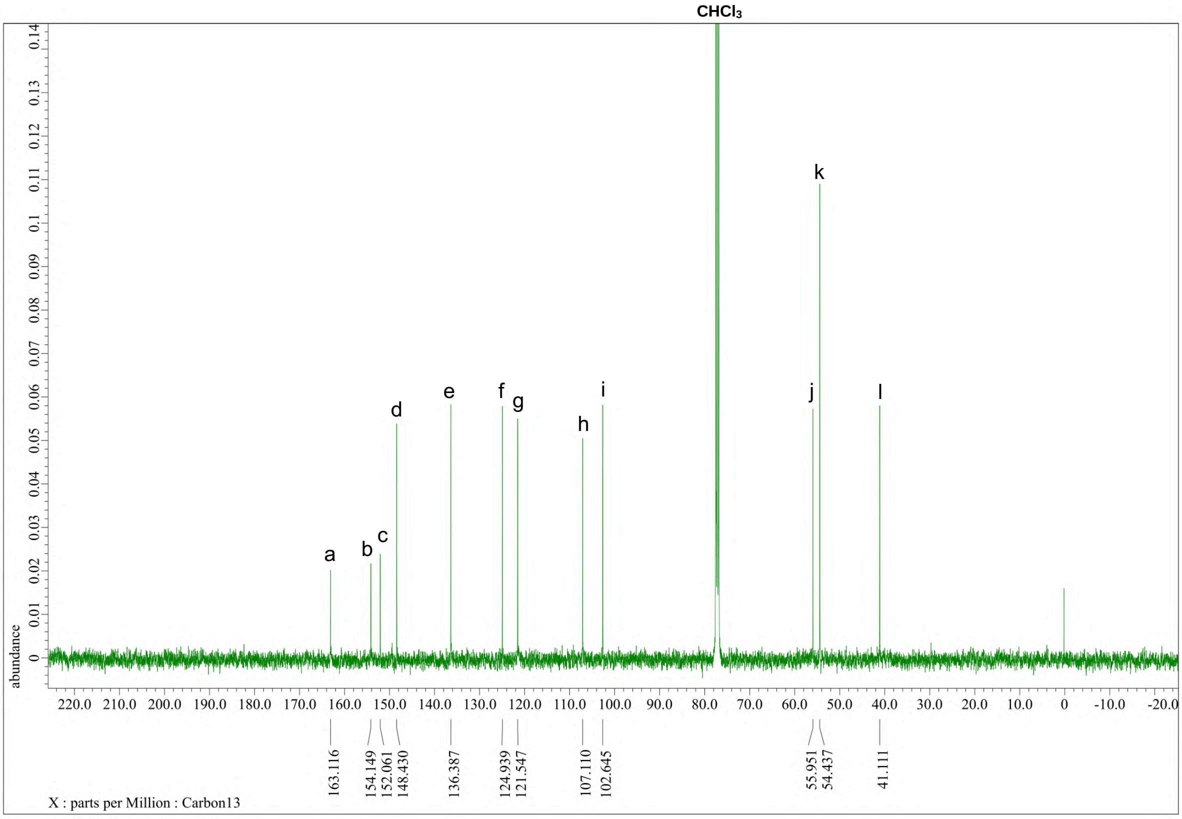
CHCl3
abundance
X : parts per Million : Carbon13
a
b
c
d
e
f
g
h
i
j
k
l
163.116
154.149
152.061
148.430
136.387
124.939
121.547
107.110
102.645
55.951
54.437
41.111

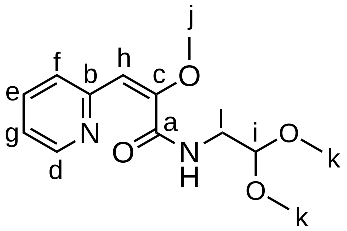

^{1}H NMR (400 MHz) and ^{13}C NMR (100 MHz) spectra of **3o** ($CDCl_3$)

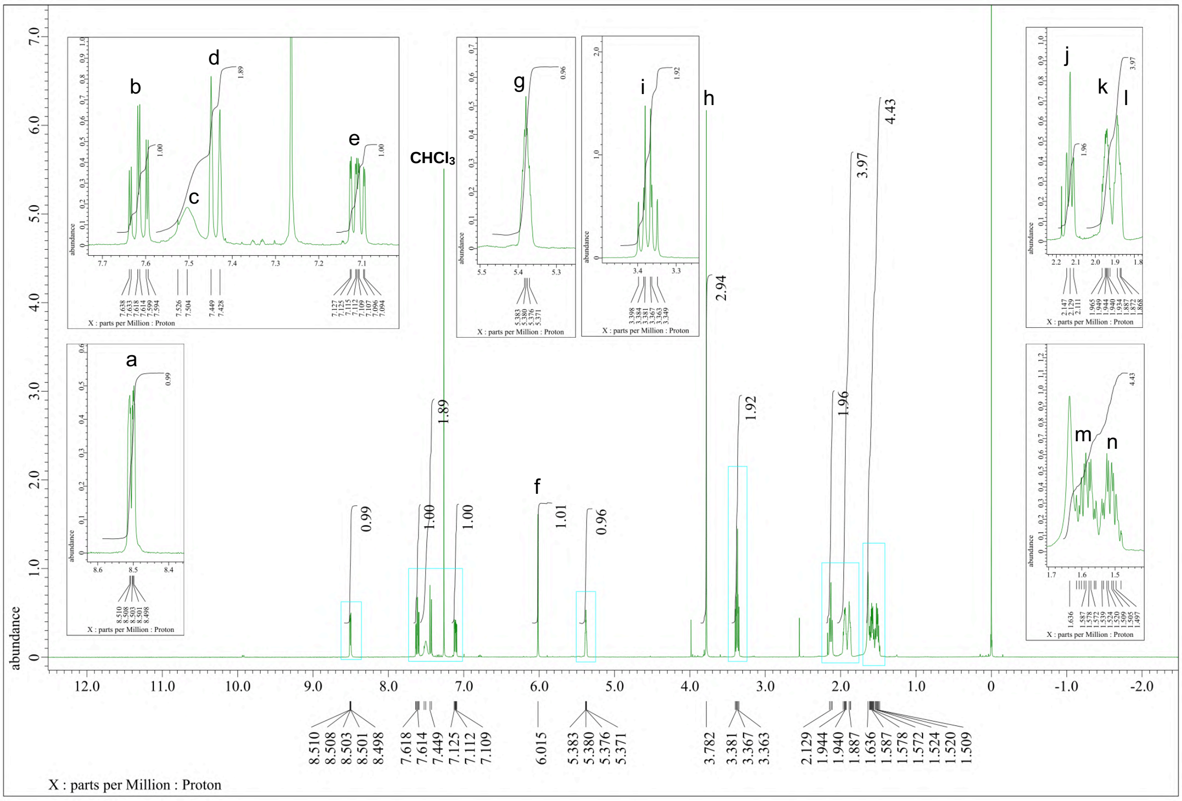

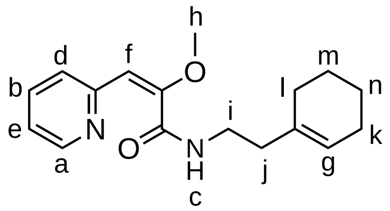

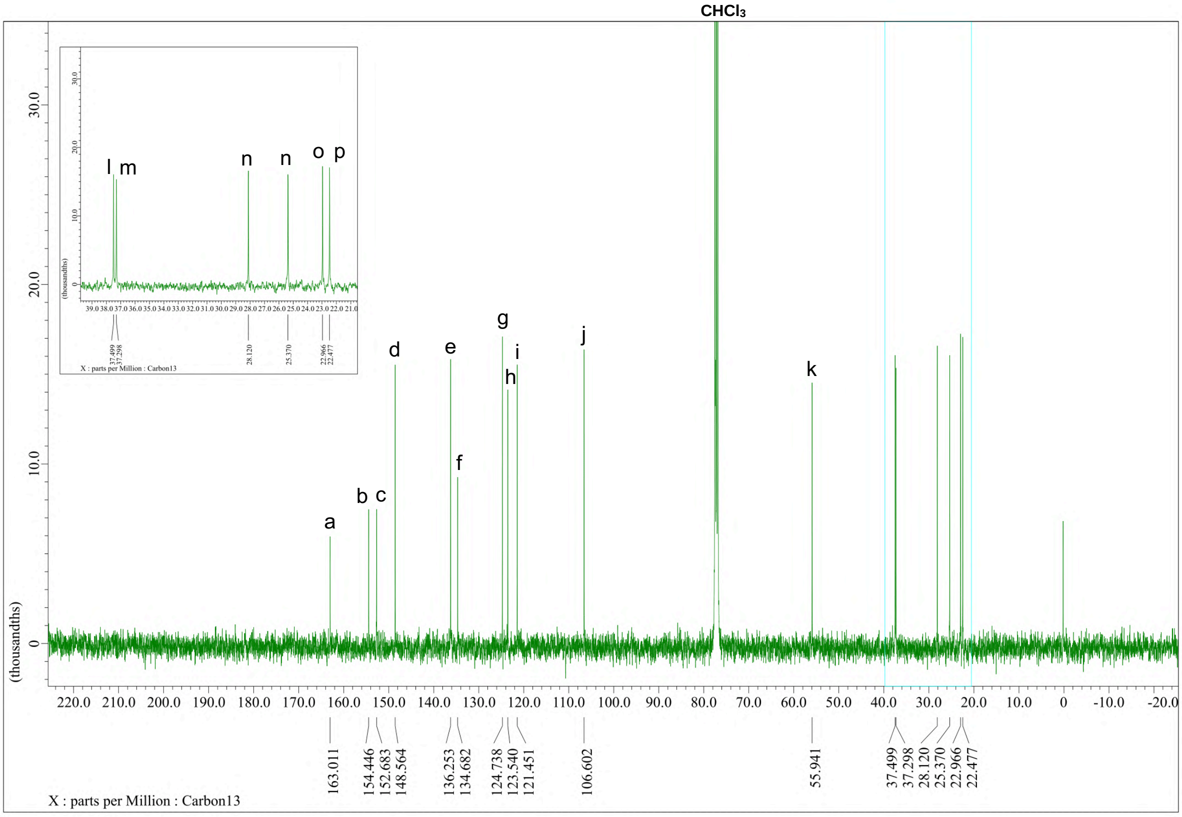
CHCl3
a
b
c
d
e
f
g
h
i
j
k
l
m
n
n
o
p
163.011
154.446
152.683
148.564
136.253
134.682
124.738
123.540
121.451
106.602
55.941
37.499
37.298
28.120
25.370
22.966
22.477
(thousandths)
X : parts per Million : Carbon13

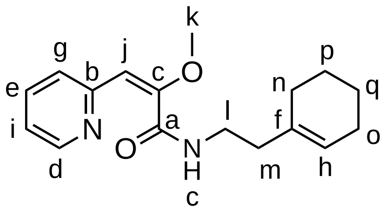
k
g
j
b
c
O
e
i
N
a
O
N
H
d
c
l
f
n
p
q
o
m
h

^1H NMR (400 MHz) and ^{13}C NMR (100 MHz) spectra of **3p** ($CDCl_3$)

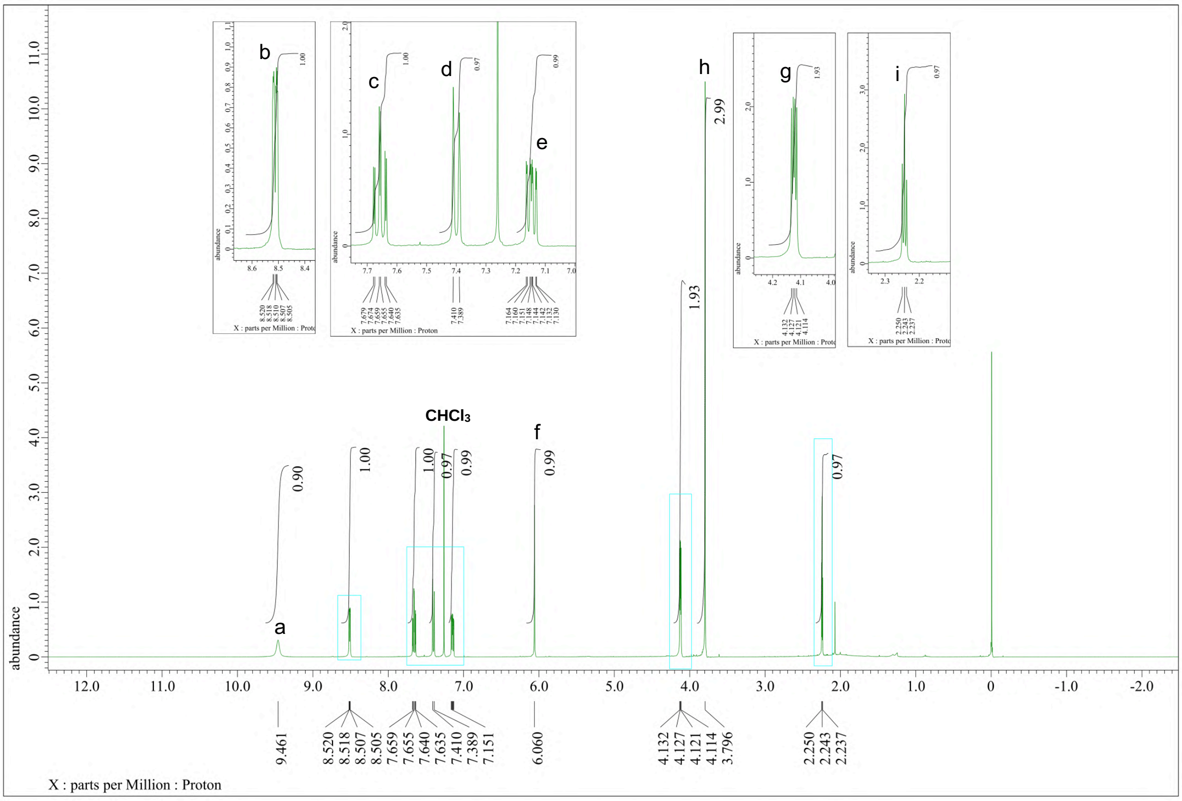

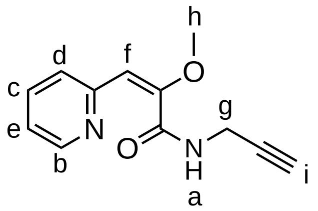

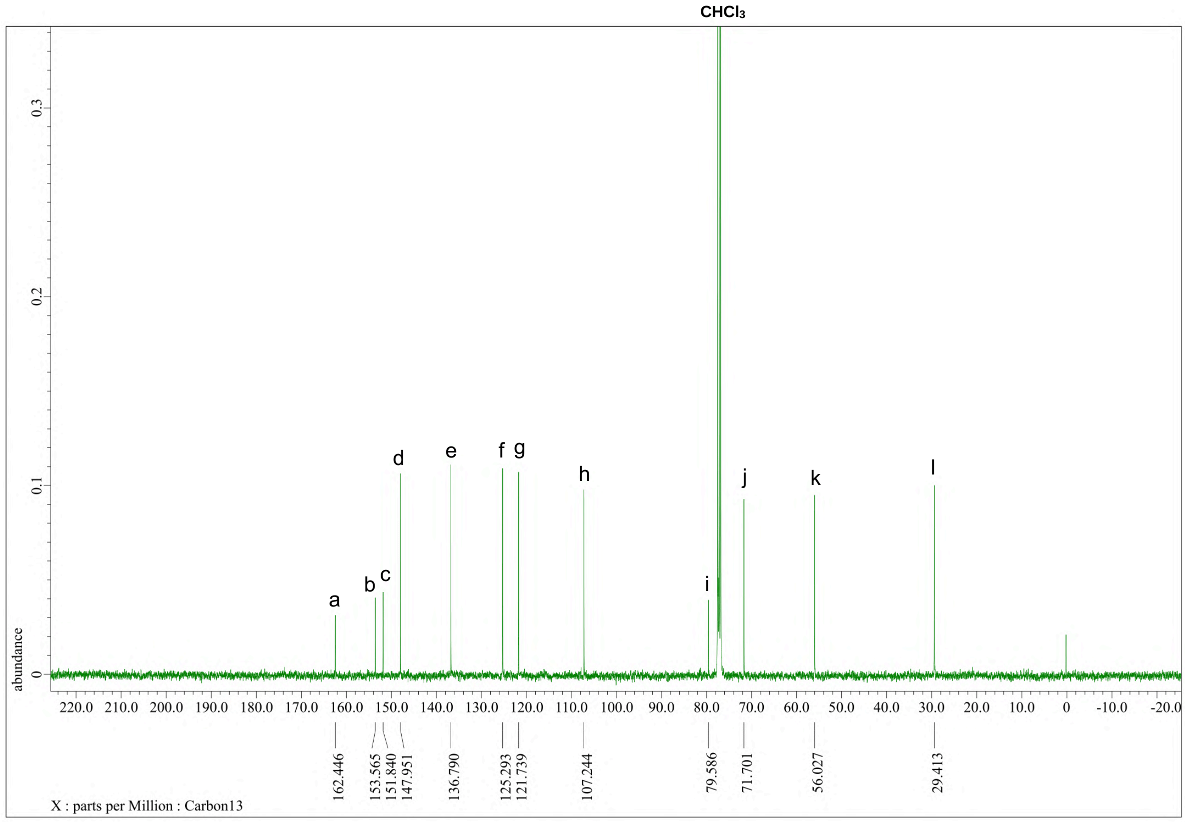
CHCl3
abundance
X : parts per Million : Carbon13
162.446
153.565
151.840
147.951
136.790
125.293
121.739
107.244
79.586
71.701
56.027
29.413

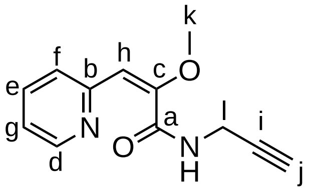

^{1}H NMR (400 MHz) and ^{13}C NMR (100 MHz) spectra of **3q** ($CDCl_3$)

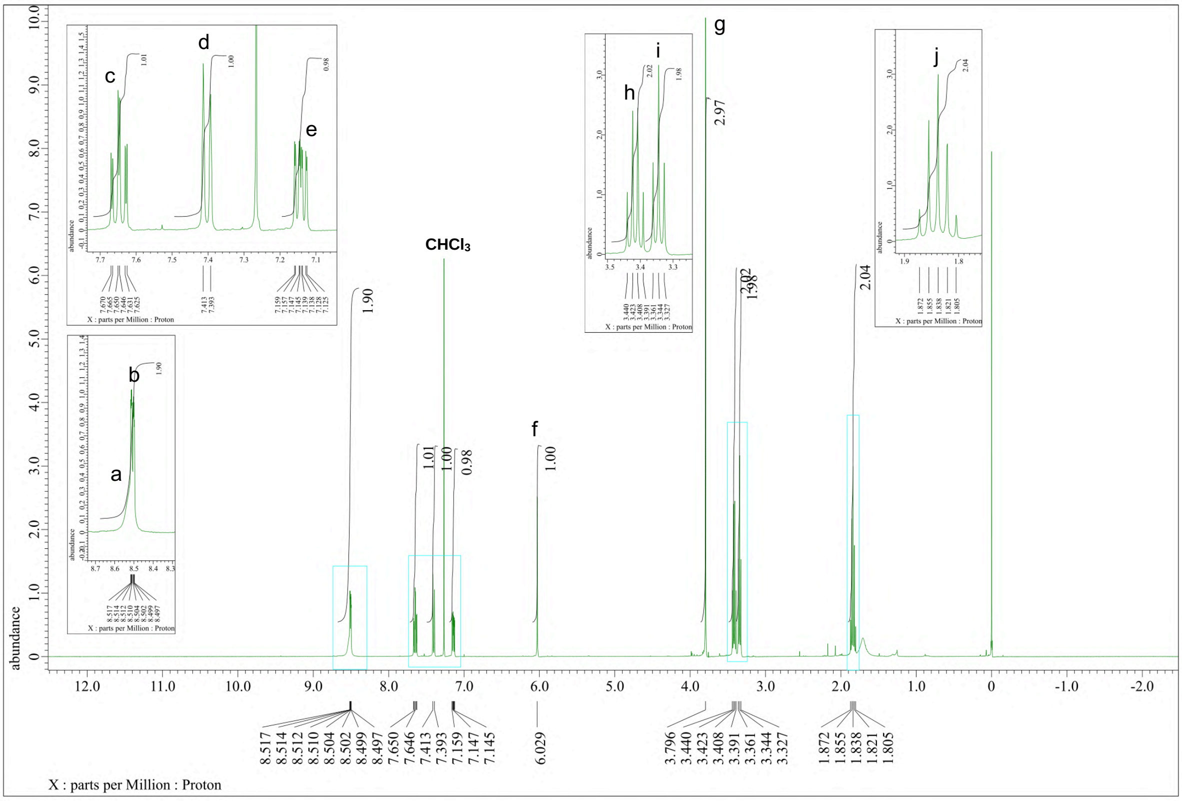

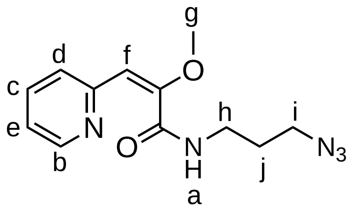

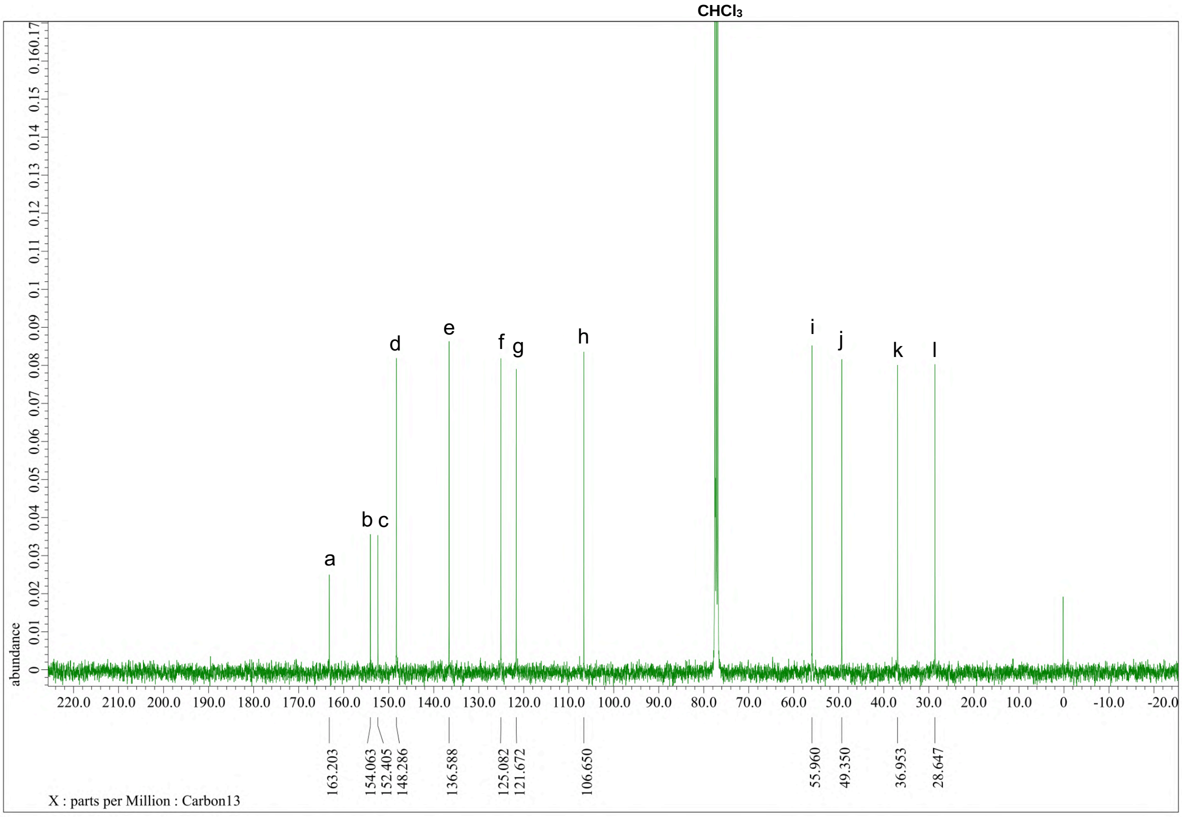
CHCl3
abundance
X : parts per Million : Carbon13
a
b c
d
e
f g
h
i
j
k
l
163.203
154.063
152.405
148.286
136.588
125.082
121.672
106.650
55.960
49.350
36.953
28.647

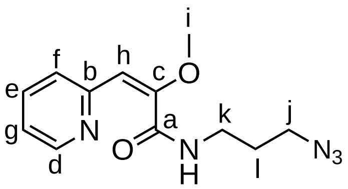
i
f
b
h
c
O
e
a
k
j
g
N
O
N
N3
d
H
l

^{1}H NMR (400 MHz) and ^{13}C NMR (100 MHz) spectra of **3r** ($CDCl_3$)

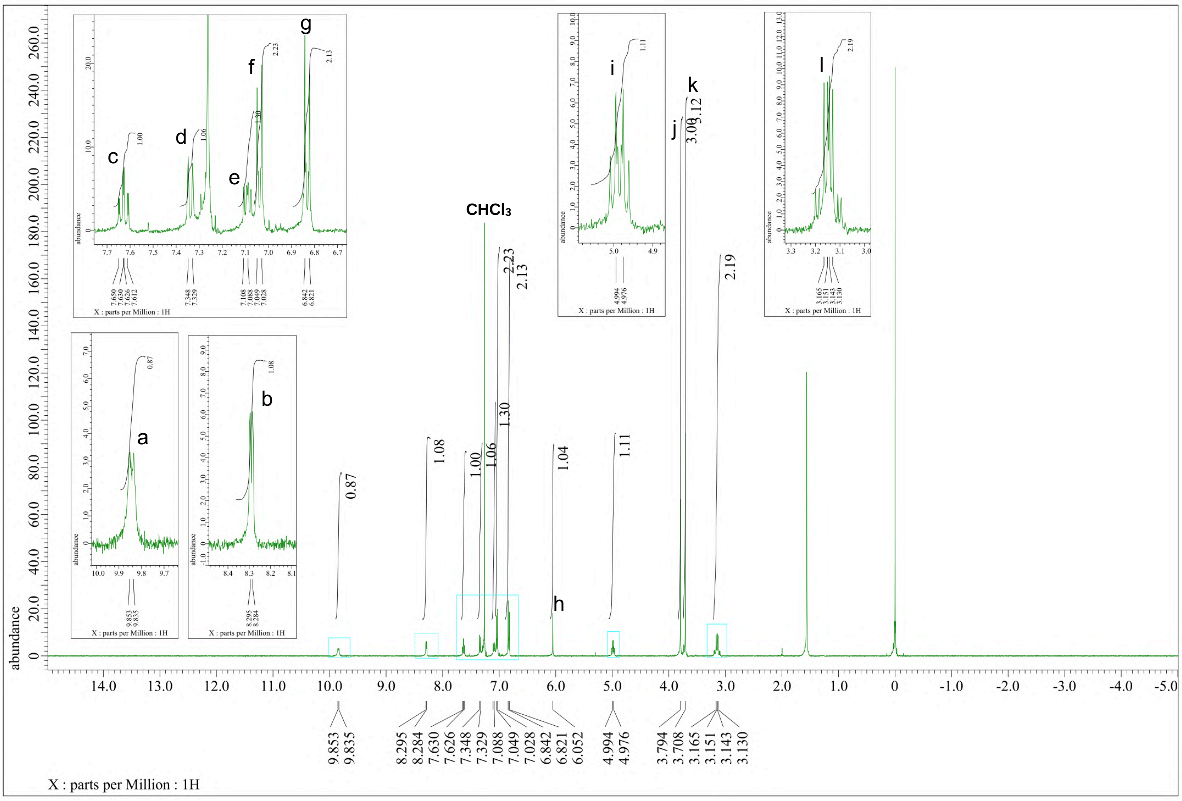

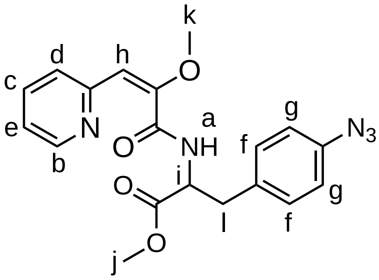

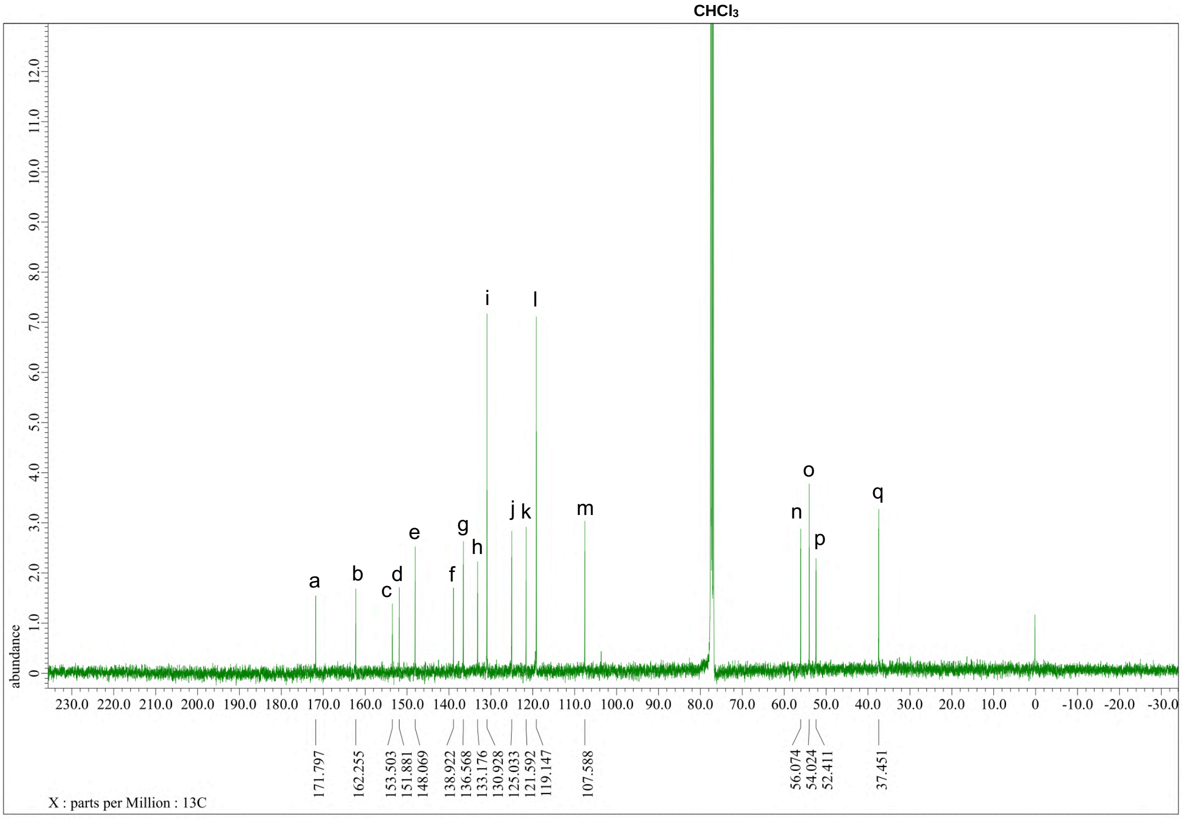
CHCl3
abundance
a
b
c
d
e
f
g
h
i
j
k
l
m
n
o
p
q
171.797
162.255
153.503
151.881
148.069
138.922
136.568
133.176
130.928
125.033
121.592
119.147
107.588
56.074
54.024
52.411
37.451
X : parts per Million : 13C

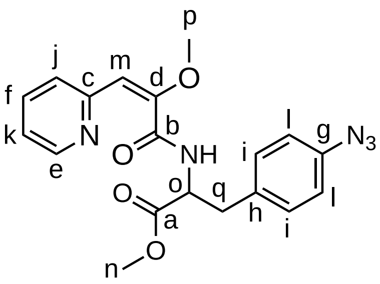
N3
NH
N
O
a
b
c
d
e
f
g
h
i
j
k
l
m
n
o
p
q

^{1}H NMR (400 MHz) and ^{13}C NMR (100 MHz) spectra of **3s** ($CDCl_3$)

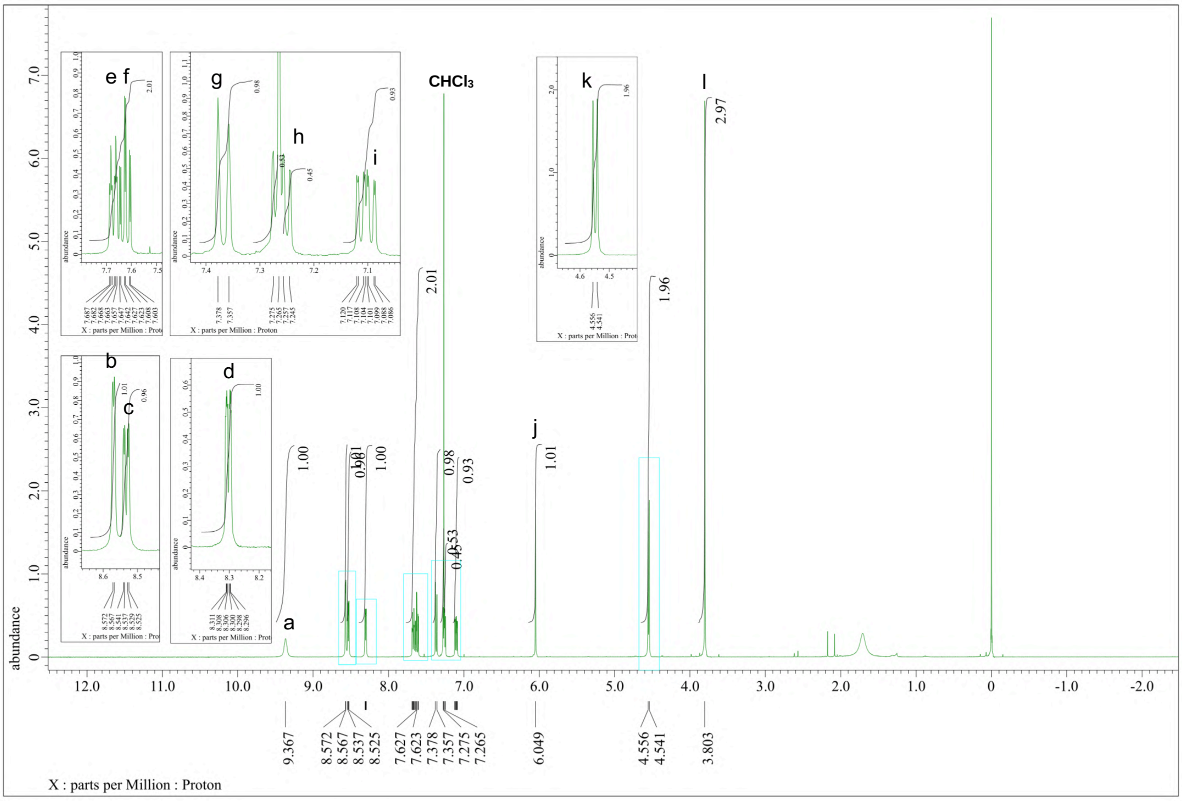

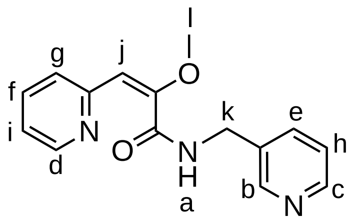

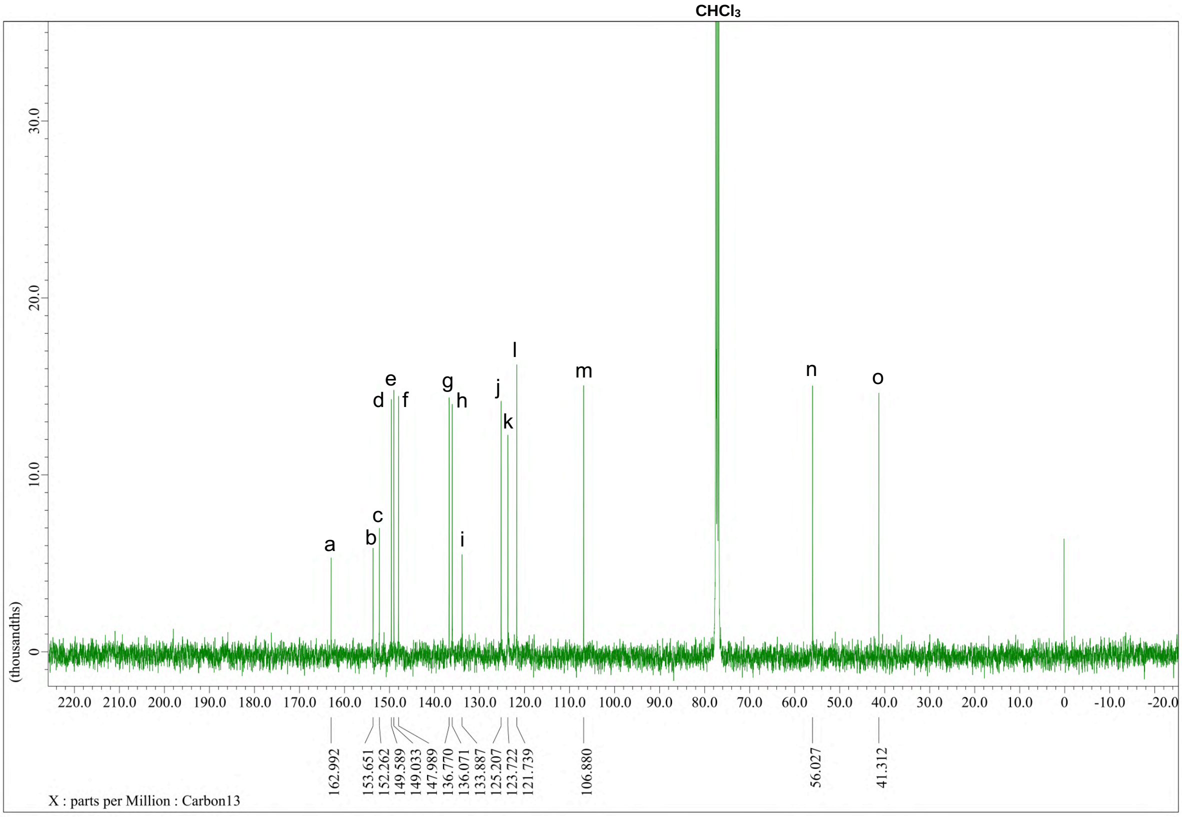
CHCl3
a
b
c
d
e
f
g
h
i
j
k
l
m
n
o
(thousandths)
30.0
20.0
10.0
0
220.0 210.0 200.0 190.0 180.0 170.0 160.0 150.0 140.0 130.0 120.0 110.0 100.0 90.0 80.0 70.0 60.0 50.0 40.0 30.0 20.0 10.0 0 -10.0 -20.0
162.992
153.651
152.262
149.589
149.033
147.989
136.770
136.071
133.887
125.207
123.722
121.739
106.880
56.027
41.312
X : parts per Million : Carbon13

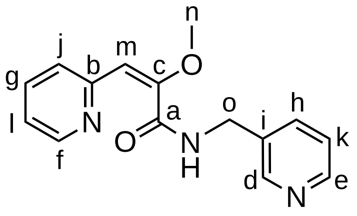
n
j
m
b
c
O
g
a
o
i
h
l
N
O
N
H
k
f
d
N
e

^{1}H NMR (400 MHz) and ^{13}C NMR (100 MHz) spectra of **3t** ($CDCl_3$)

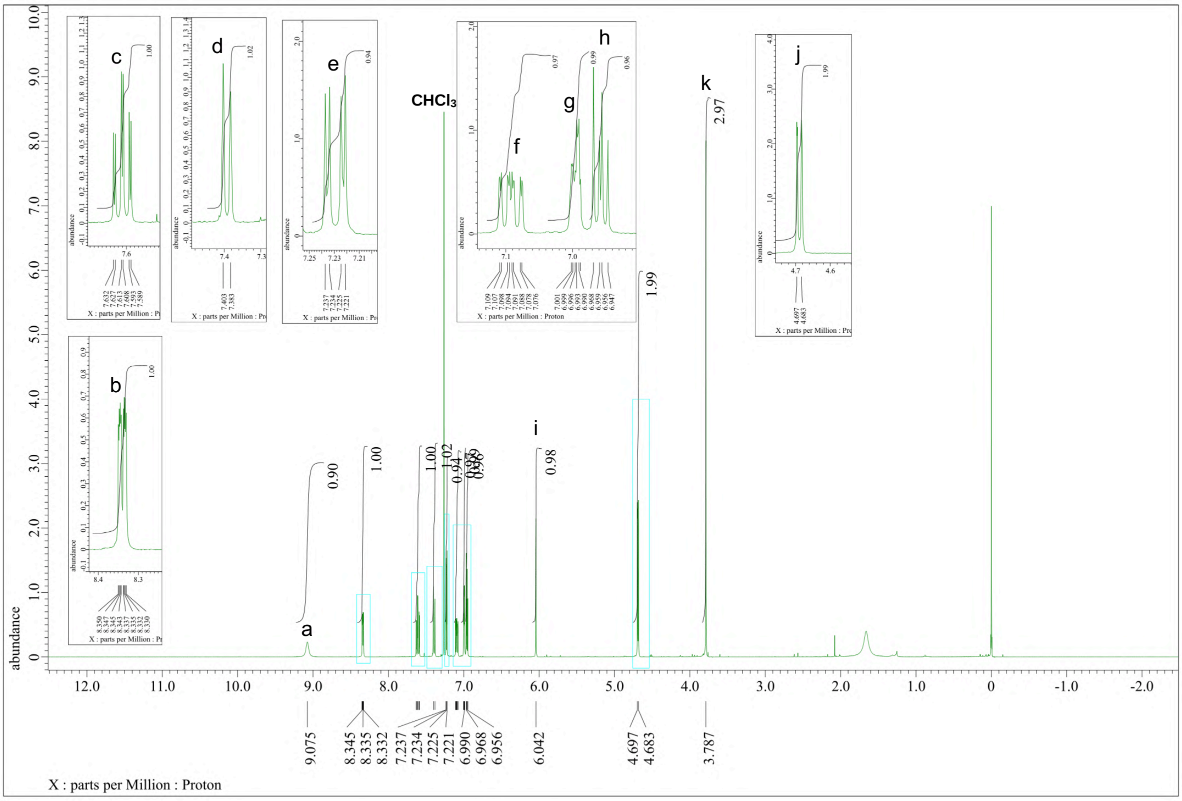

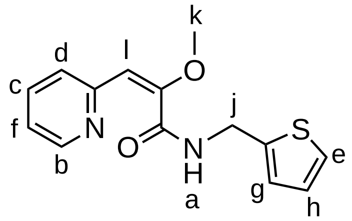

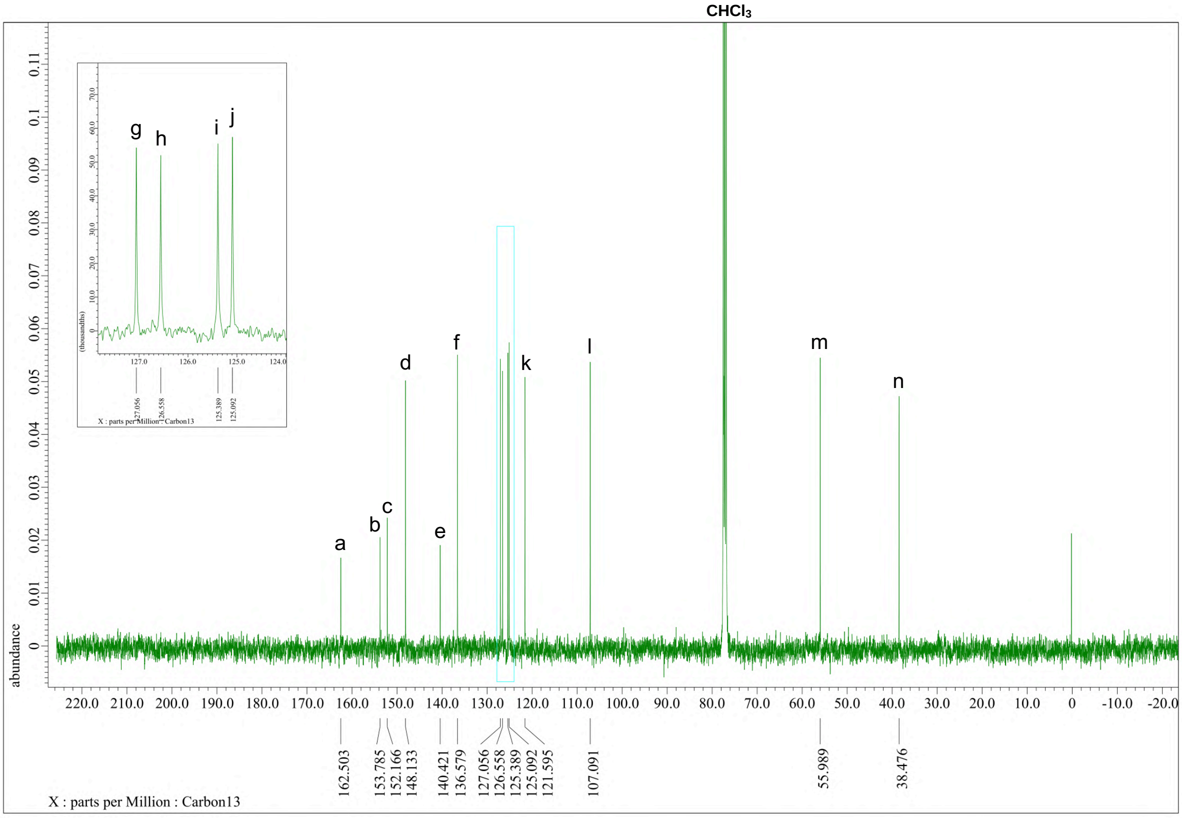
CHCl3
abundance
X : parts per Million : Carbon13
a
b
c
d
e
f
k
l
m
n
g
h
i
j
162.503
153.785
152.166
148.133
140.421
136.579
127.056
126.558
125.389
125.092
121.595
107.091
55.989
38.476

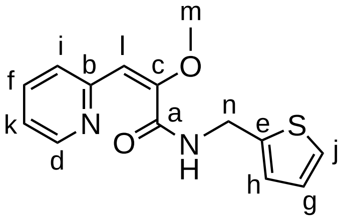

^{1}H NMR (400 MHz) and ^{13}C NMR (100 MHz) spectra of **3u** ($CDCl_3$)

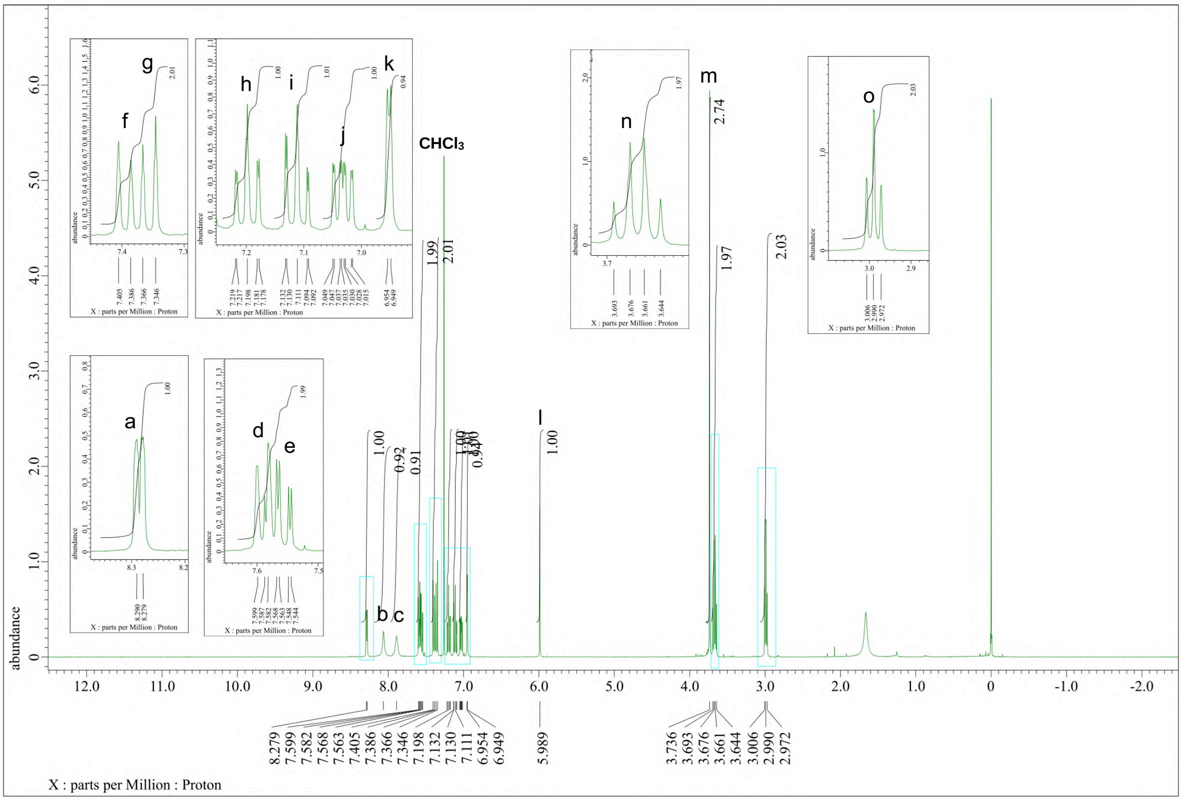

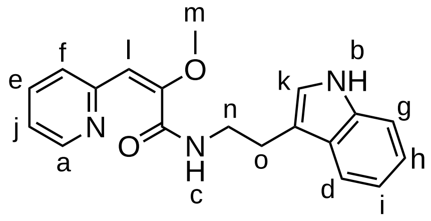

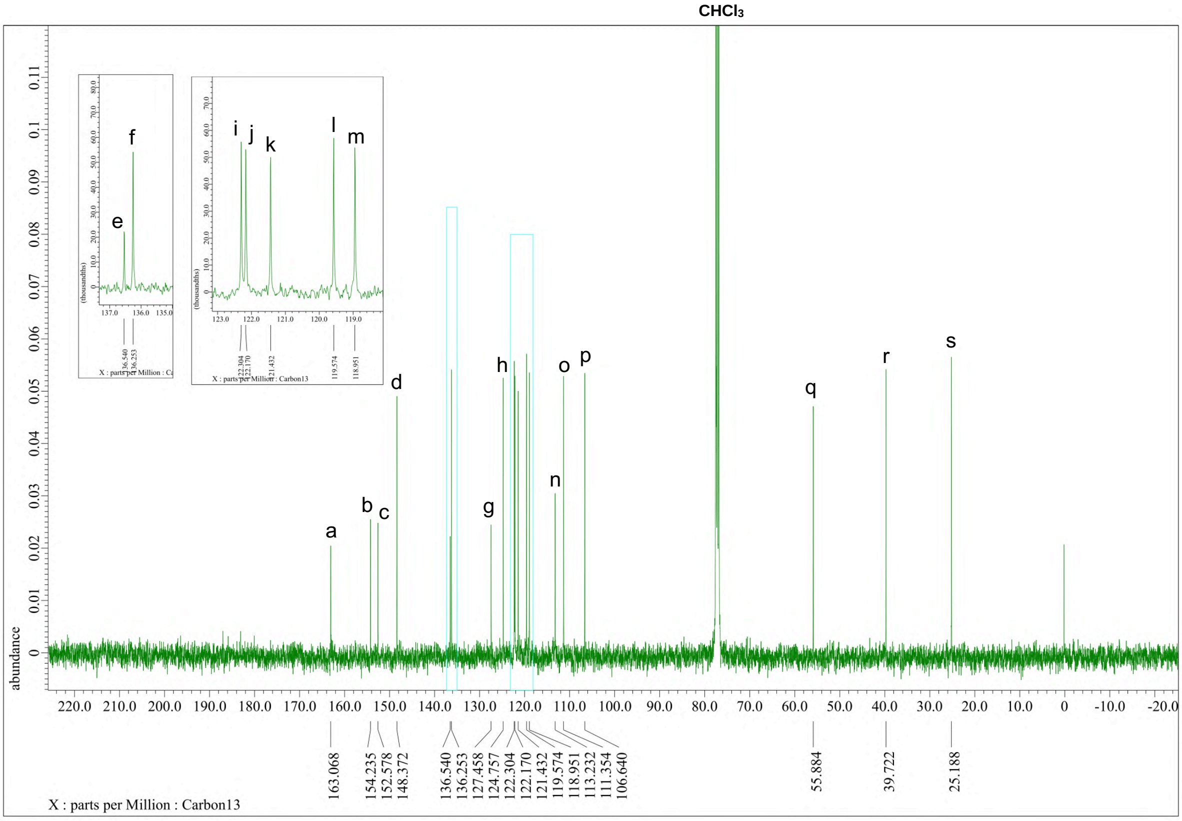
CHCl3
abundance
X : parts per Million : Carbon13
a
b
c
d
e
f
g
h
i
j
k
l
m
n
o
p
q
r
s
163.068
154.235
152.578
148.372
136.540
136.253
127.458
124.757
122.304
122.170
121.432
119.574
118.951
113.232
111.354
106.640
55.884
39.722
25.188

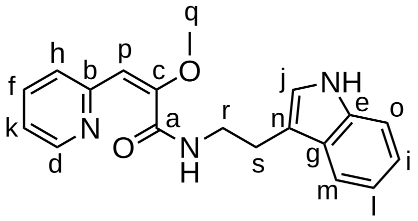
q
O
p
c
h
b
f
k
N
d
O
a
N
H
r
s
j
NH
n
e
o
g
i
m
l

^{1}H NMR (400 MHz), ^{13}C NMR (100 MHz) and ^{19}F NMR (373 MHz) spectra of **3v** ($CDCl_3$)

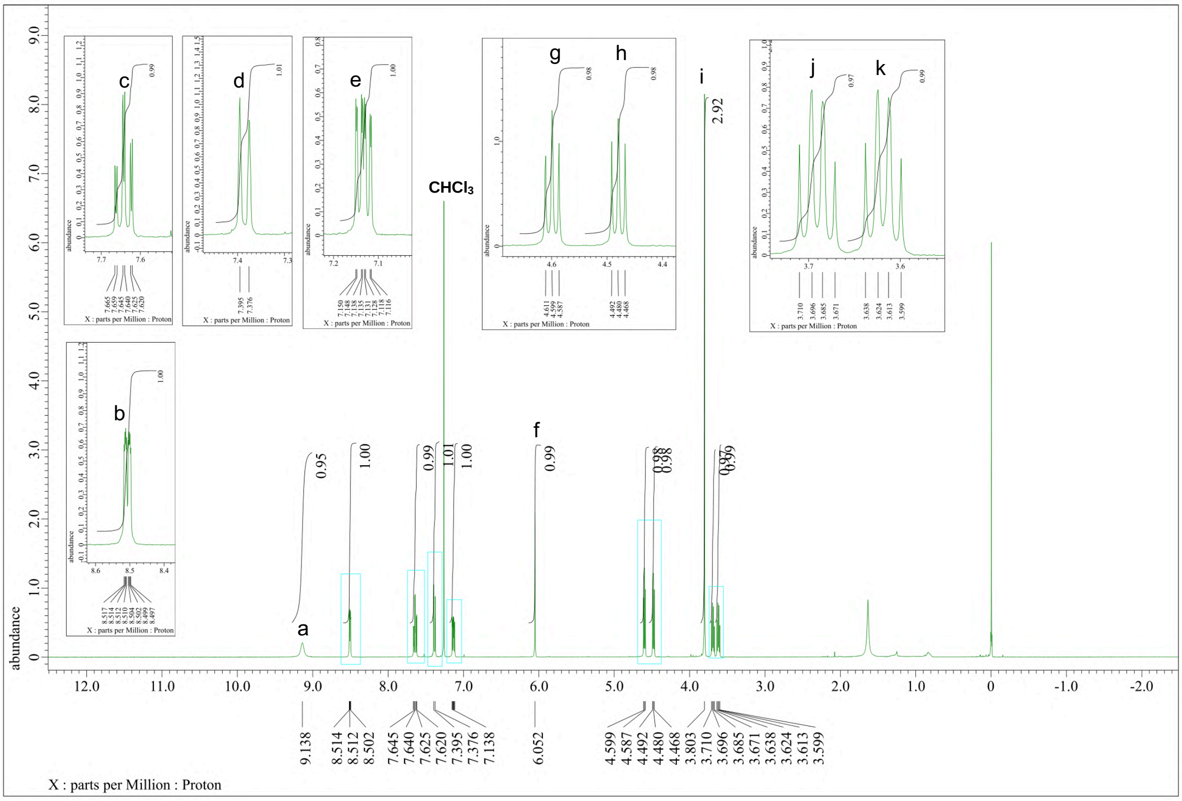

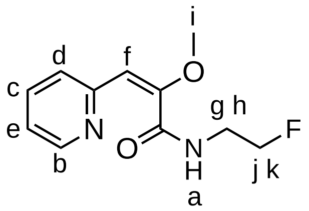

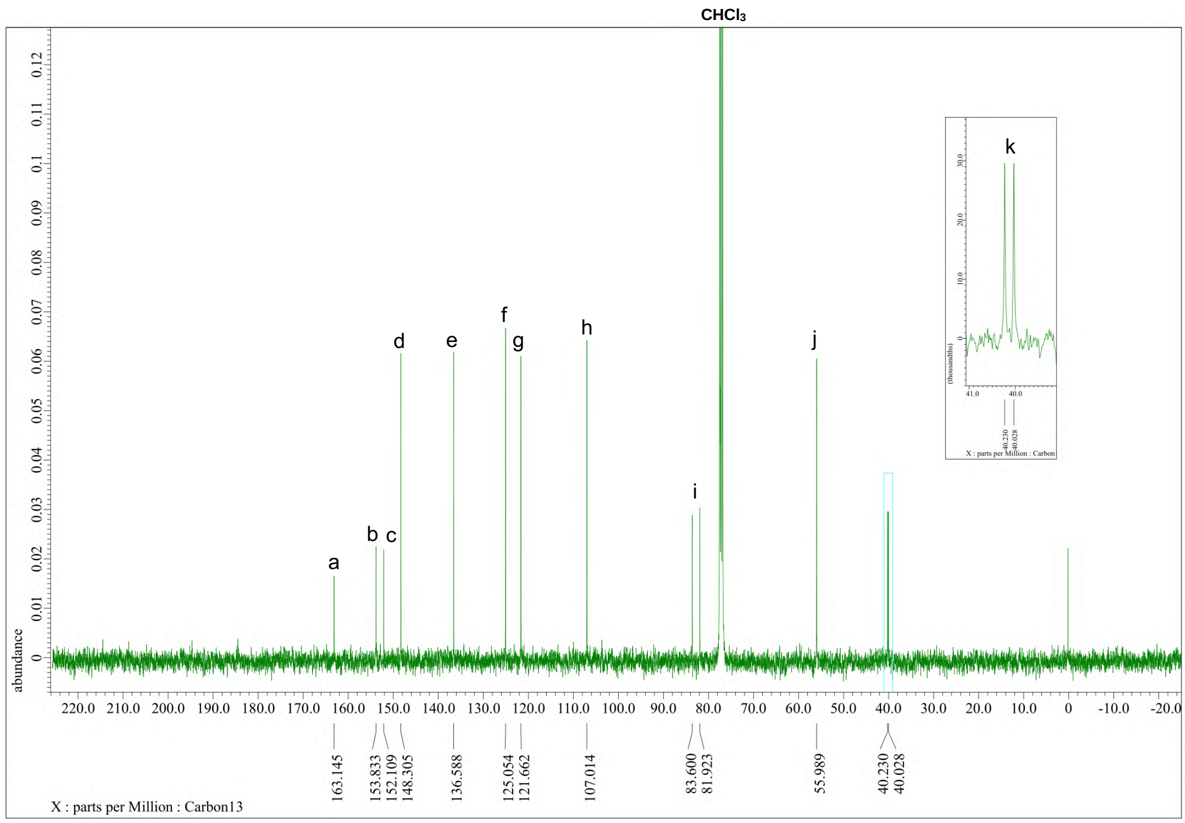
CHCl3
abundance
X : parts per Million : Carbon13
a
b
c
d
e
f
g
h
i
j
k
163.145
153.833
152.109
148.305
136.588
125.054
121.662
107.014
83.600
81.923
55.989
40.230
40.028
X : parts per Million : Carbon

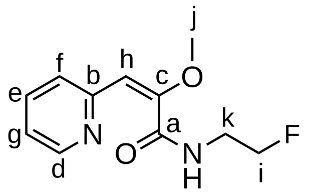
j
O
F
N
H

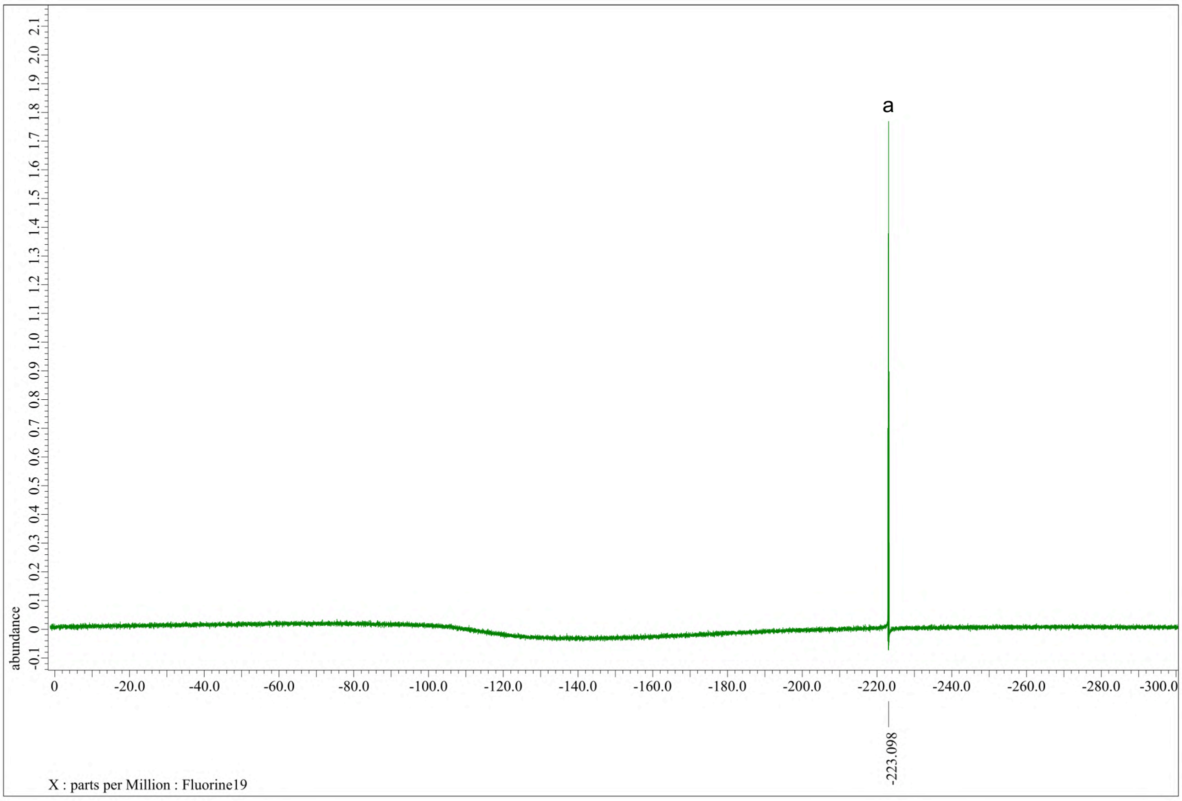
a
abundance
-0.1
0
0.1
0.2
0.3
0.4
0.5
0.6
0.7
0.8
0.9
1.0
1.1
1.2
1.3
1.4
1.5
1.6
1.7
1.8
1.9
2.0
2.1
0
-20.0
-40.0
-60.0
-80.0
-100.0
-120.0
-140.0
-160.0
-180.0
-200.0
-220.0
-240.0
-260.0
-280.0
-300.0
-223.098
X : parts per Million : Fluorine19

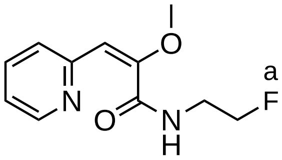
O
a
N
F
O
N
H

^{1}H NMR (400 MHz), ^{13}C NMR (100 MHz) and ^{19}F NMR (373 MHz) spectra of **3w** ($CDCl_3$)

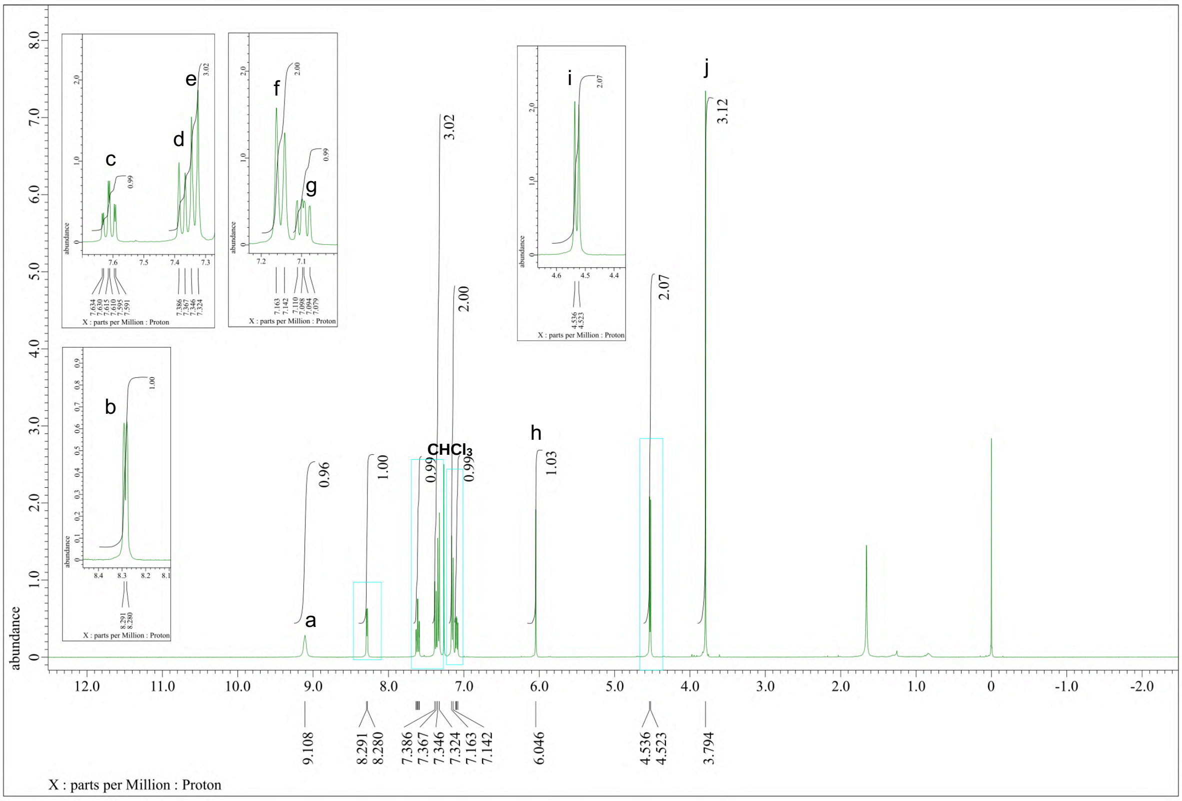

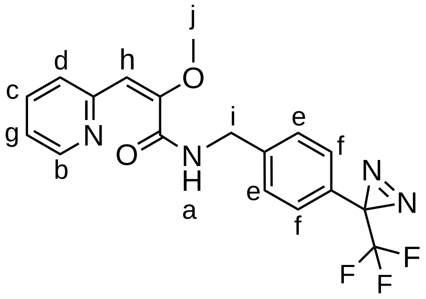

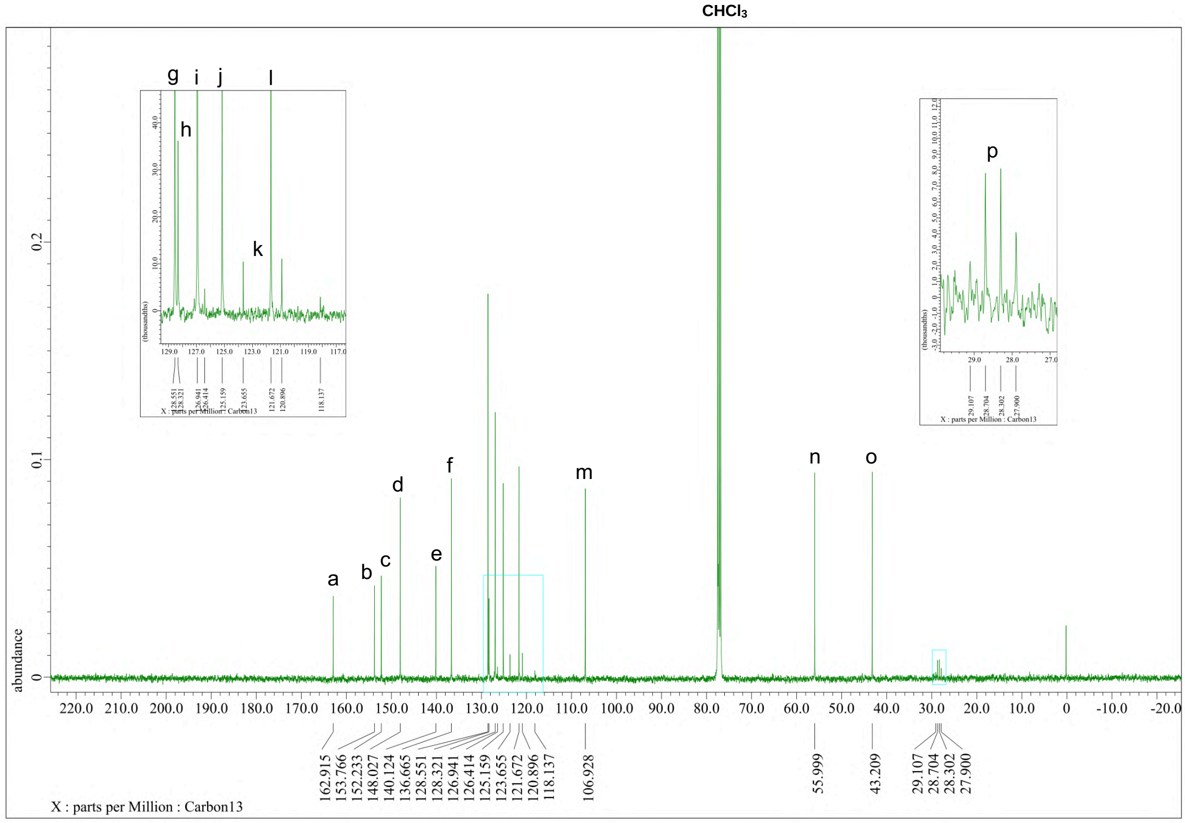
CHCl3
abundance
X : parts per Million : Carbon13
162.915
153.766
152.233
148.027
140.124
136.665
128.551
128.321
126.941
126.414
125.159
123.655
121.672
120.896
118.137
106.928
55.999
43.209
29.107
28.704
28.302
27.900

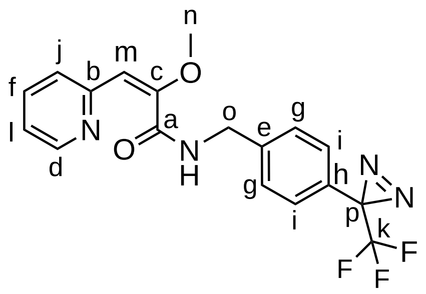

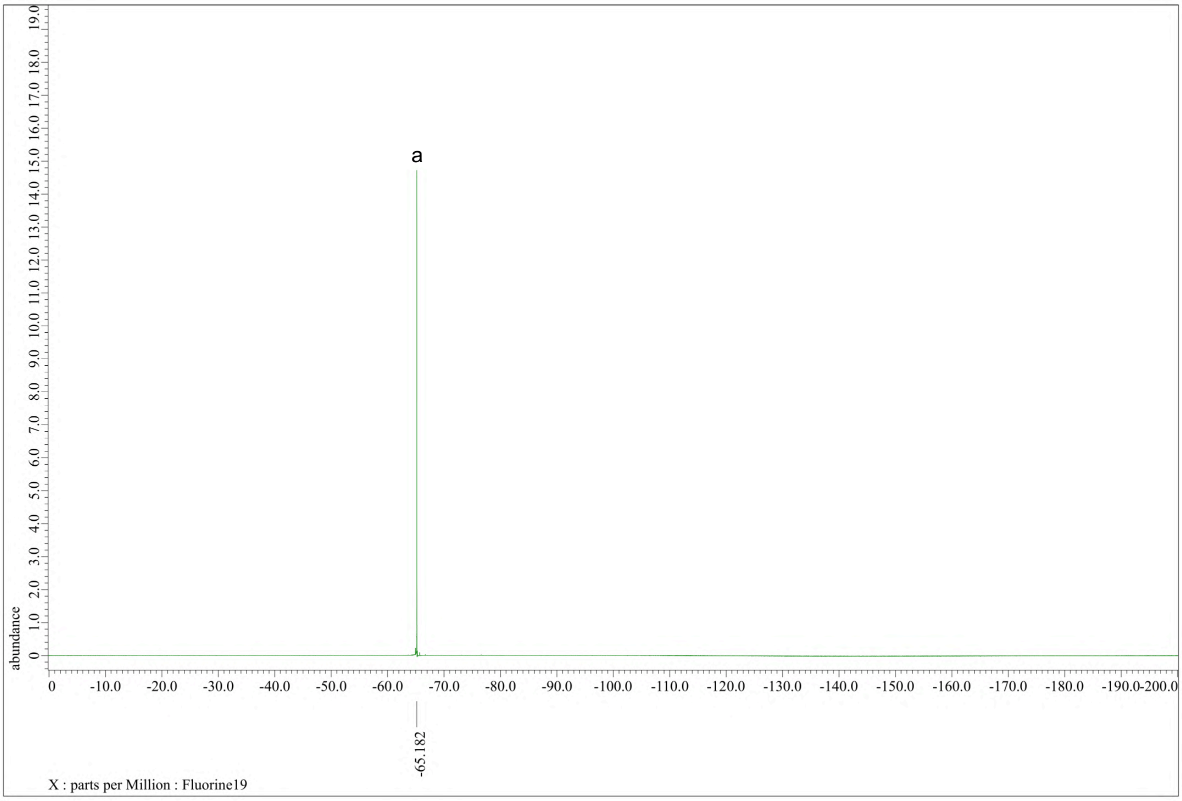
a
abundance
X : parts per Million : Fluorine19
-65.182

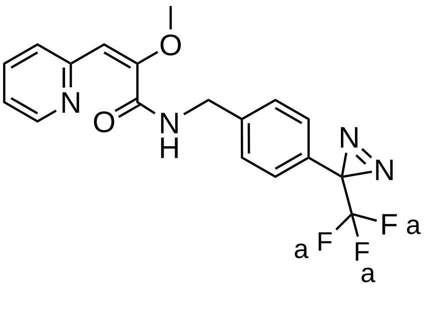

^{1}H NMR (400 MHz) and ^{13}C NMR (100 MHz) spectra of **3x** ($CDCl_3$)

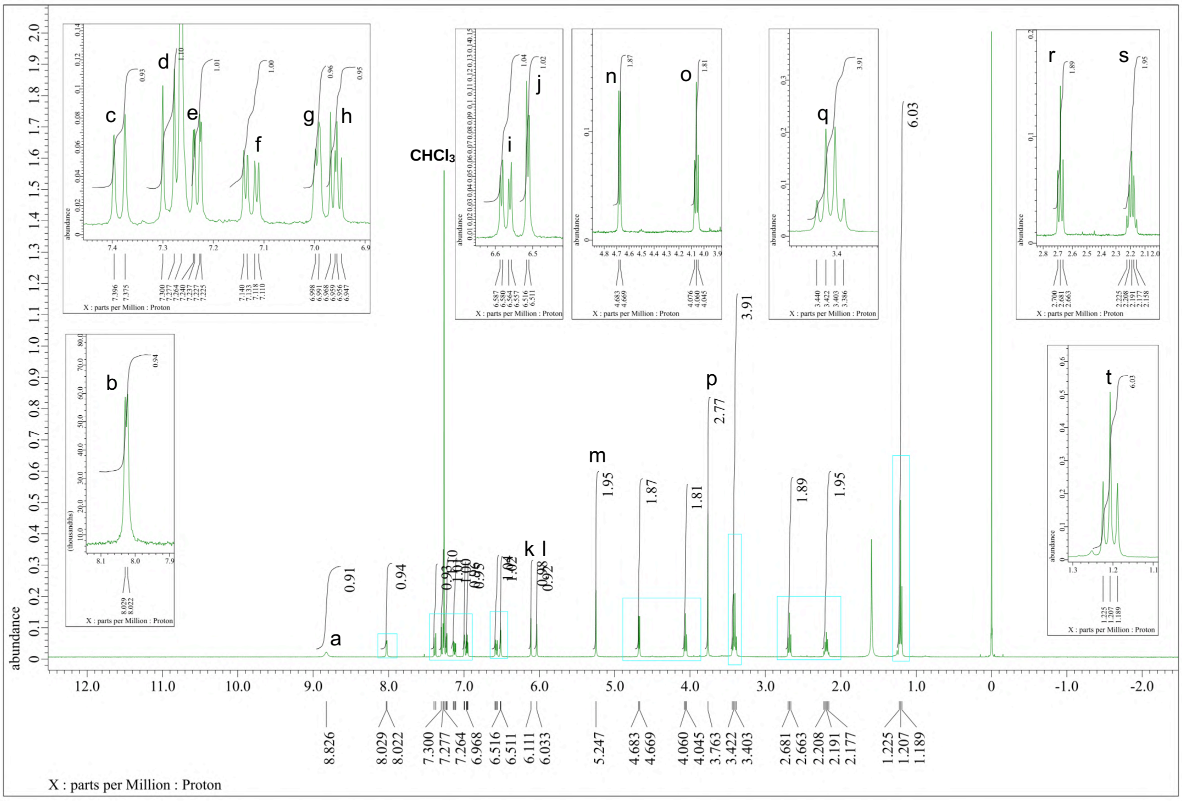

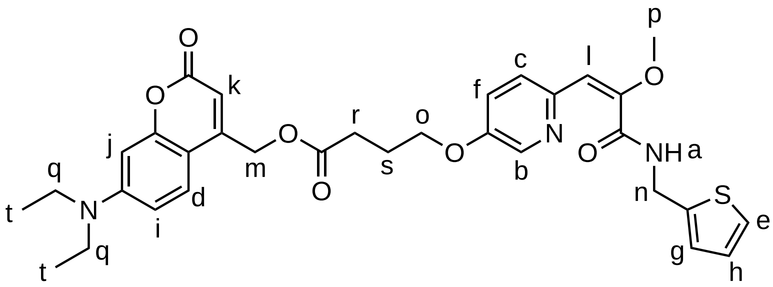

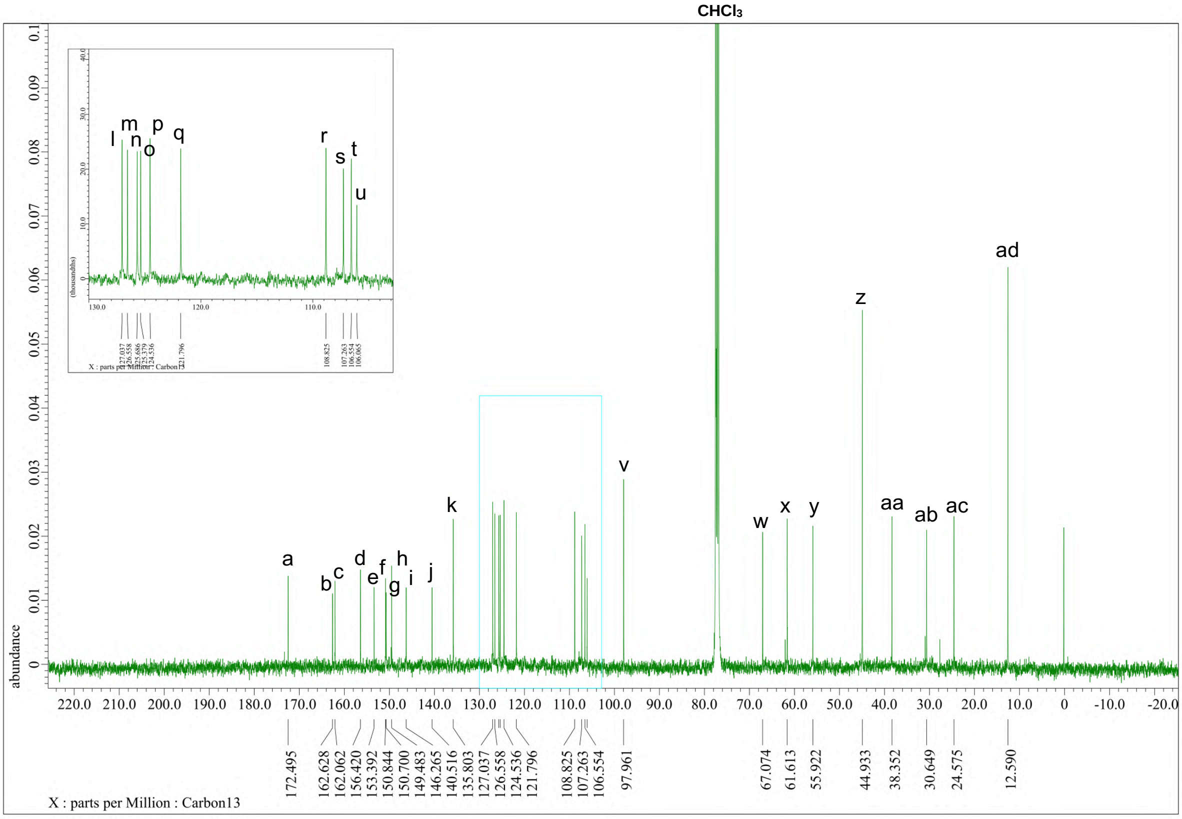
CHCl3
abundance
X : parts per Million : Carbon13
172.495
162.628
162.062
156.420
153.392
150.844
150.700
149.483
146.265
140.516
135.803
127.037
126.558
124.536
121.796
108.825
107.263
106.554
97.961
67.074
61.613
55.922
44.933
38.352
30.649
24.575
12.590
106.065
125.379

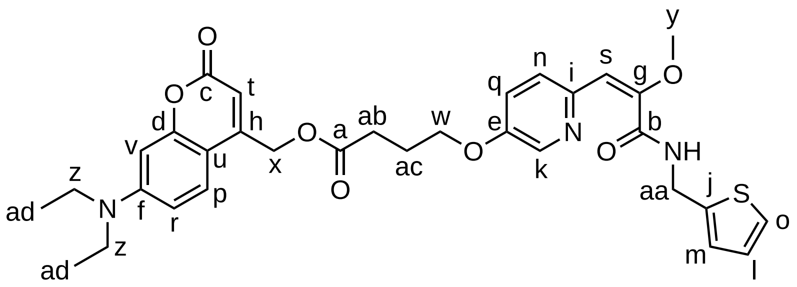

^{1}H NMR (400 MHz), ^{13}C NMR (100 MHz) and ^{19}F NMR (373 MHz) spectra of **6** ($CDCl_3$)

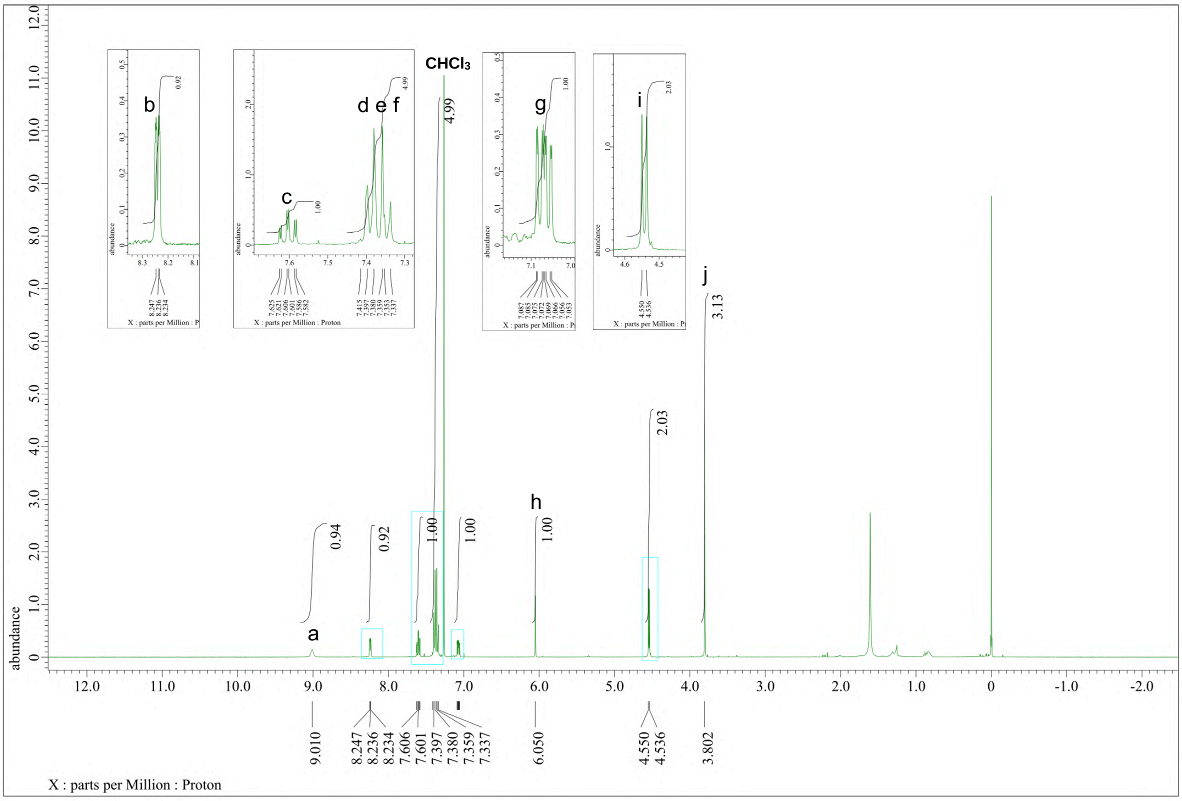

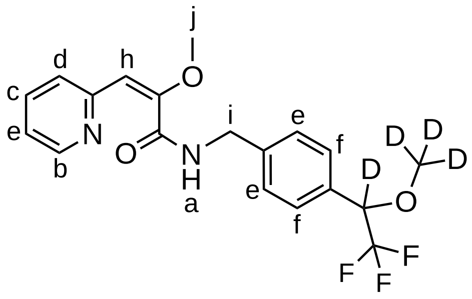

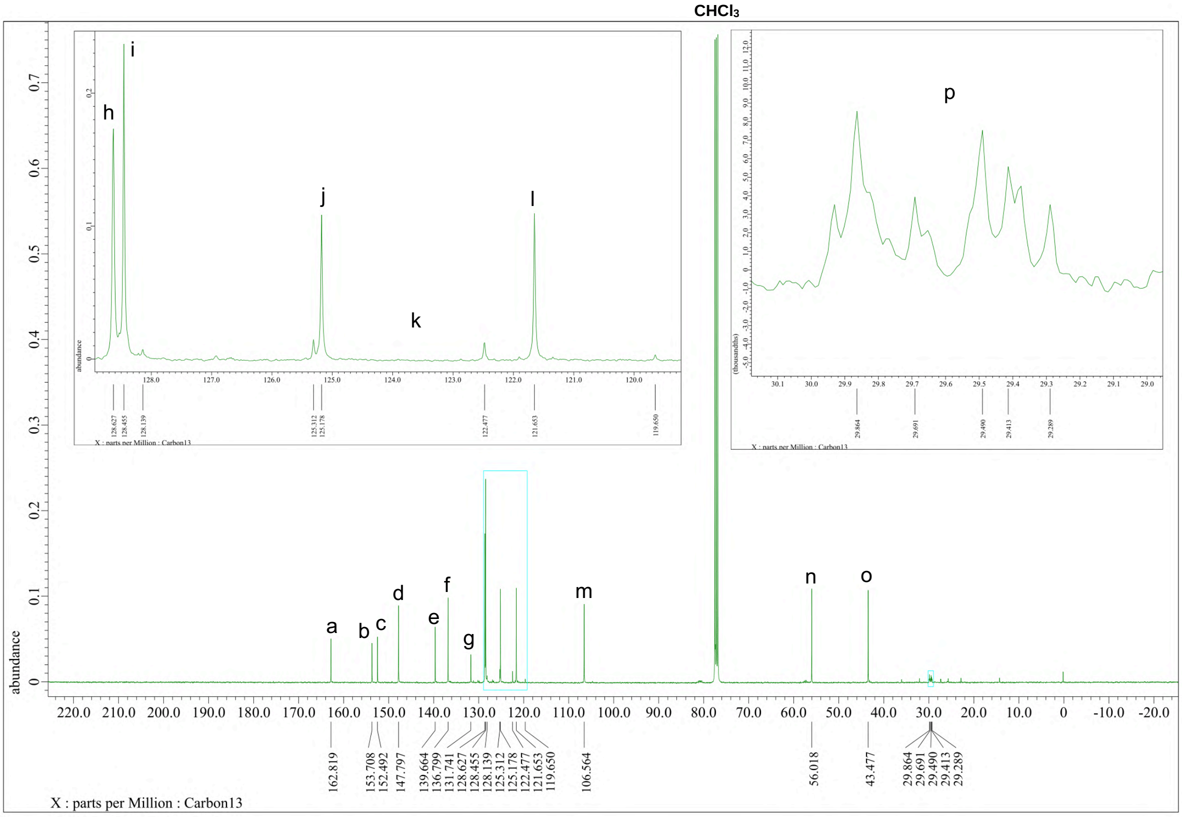
CHCl3
abundance
X : parts per Million : Carbon13
a
b
c
d
e
f
g
h
i
j
k
l
m
n
o
p
162.819
153.708
152.492
147.797
139.664
136.799
131.741
128.627
128.455
128.139
125.312
125.178
122.477
121.653
119.650
106.564
56.018
43.477
29.864
29.691
29.490
29.413
29.289

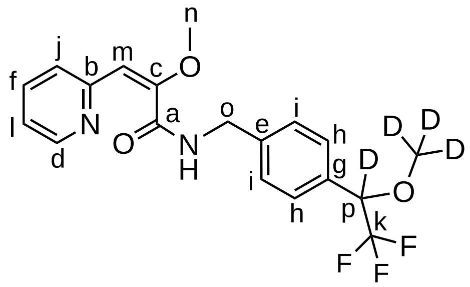
a
b
c
d
e
f
g
h
i
j
l
m
n
o
p
k
O
N
NH
D
F

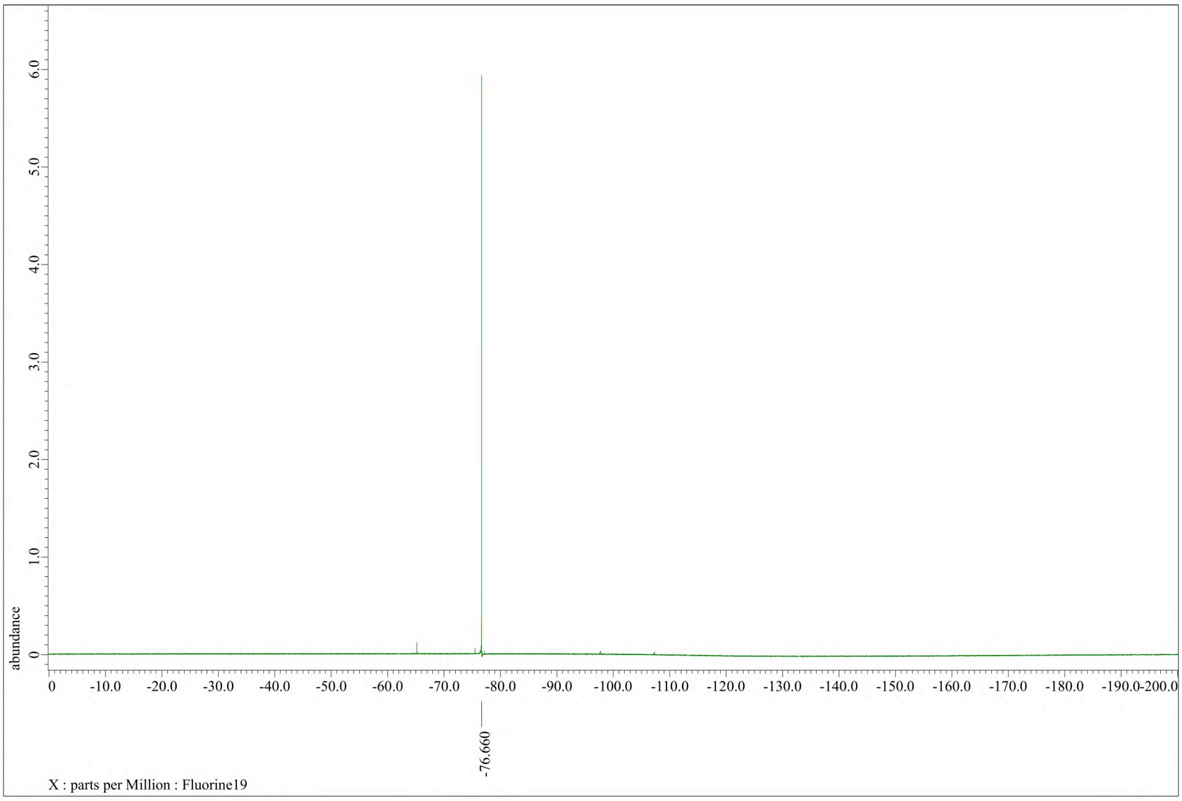
abundance
0
1.0
2.0
3.0
4.0
5.0
6.0
0
-10.0
-20.0
-30.0
-40.0
-50.0
-60.0
-70.0
-80.0
-90.0
-100.0
-110.0
-120.0
-130.0
-140.0
-150.0
-160.0
-170.0
-180.0
-190.0
-200.0
-76.660
X : parts per Million : Fluorine19

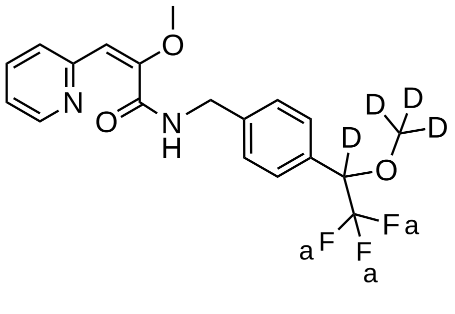
O
N
O
N
H
D
D
D
D
O
F a
a F
F
a
